# Abundant copy-number loss of CYCLOPS and STOP genes in gastric adenocarcinoma

**DOI:** 10.1007/s10120-015-0514-z

**Published:** 2015-07-24

**Authors:** Ioana Cutcutache, Alice Yingting Wu, Yuka Suzuki, John Richard McPherson, Zhengdeng Lei, Niantao Deng, Shenli Zhang, Wai Keong Wong, Khee Chee Soo, Weng Hoong Chan, London Lucien Ooi, Roy Welsch, Patrick Tan, Steven G. Rozen

**Affiliations:** Program in Cancer and Stem Cell Biology, Duke-NUS Graduate Medical School, Singapore, Singapore; Centre for Computational Biology, Duke-NUS Graduate Medical School, Singapore, Singapore; Computation and Systems Biology, Singapore-MIT Alliance, Singapore, Singapore; NUS Graduate School for Integrative Science and Engineering, National University of Singapore, Singapore, Singapore; Department of General Surgery, Singapore General Hospital, Singapore, Singapore; Division of Surgical Oncology, National Cancer Centre Singapore, Singapore, Singapore; Engineering Systems Division and Sloan School of Management, Massachusetts Institute of Technology, Cambridge, MA USA; Duke-NUS Genome Biology Facility, Duke-NUS Graduate Medical School, Singapore, Singapore; Genome Institute of Singapore, A* STAR, Singapore, Singapore

**Keywords:** DNA copy number change, Gastric cancer, Loss of heterozygosity, Tumor suppressor genes

## Abstract

**Background:**

Gastric cancer, a leading cause of cancer death worldwide, has been little studied compared with other cancers that impose similar health burdens. Our goal is to assess genomic copy-number loss and the possible functional consequences and therapeutic implications thereof across a large series of gastric adenocarcinomas.

**Methods:**

We used high-density single-nucleotide polymorphism microarrays to determine patterns of copy-number loss and allelic imbalance in 74 gastric adenocarcinomas. We investigated whether suppressor of tumorigenesis and/or proliferation (STOP) genes are associated with genomic copy-number loss. We also analyzed the extent to which copy-number loss affects Copy-number alterations Yielding Cancer Liabilities Owing to Partial losS (CYCLOPS) genes–genes that may be attractive targets for therapeutic inhibition when partially deleted.

**Results:**

The proportion of the genome subject to copy-number loss varies considerably from tumor to tumor, with a median of 5.5 %, and a mean of 12 % (range 0–58.5 %). On average, 91 STOP genes were subject to copy-number loss per tumor (median 35, range 0–452), and STOP genes tended to have lower copy-number compared with the rest of the genes. Furthermore, on average, 1.6 CYCLOPS genes per tumor were both subject to copy-number loss and downregulated, and 51.4 % of the tumors had at least one such gene.

**Conclusions:**

The enrichment of STOP genes in regions of copy-number loss indicates that their deletion may contribute to gastric carcinogenesis. Furthermore, the presence of several deleted and downregulated CYCLOPS genes in some tumors suggests potential therapeutic targets in these tumors.

**Electronic supplementary material:**

The online version of this article (doi:10.1007/s10120-015-0514-z) contains supplementary material, which is available to authorized users.

## Introduction

Gastric cancer is the fourth commonest cancer in the world and a leading cause of cancer death [1]. In 2008, it caused 738,000 deaths (10 % of all cancer-related deaths) [2]. Gastric cancer is especially prevalent in East Asia, Eastern Europe, and parts of Central America and South America [2]. Current treatments offer only slight survival benefits. Except in Japan, where endoscopic screening often detects early-stage tumors, the overall 5-year survival rate is 20–25 % [3].

Although there have been many studies of loss of heterozygosity (LOH) and copy-number loss in gastric cancer [4–9], to our knowledge none of these studies systematically surveyed copy-number loss and its effects on genes retarding proliferation or genes that, when deleted, might constitute therapeutic vulnerabilities. At present, high-density microarrays provide simultaneous assessment of single-nucleotide polymorphism (SNP) genotype and genomic copy number at hundreds of thousands of sites across the genome, and can thus delineate regions of copy-number loss [10].

It has recently emerged that copy-number loss is likely important in two distinct aspects of cancer biology. In one aspect, it appears that copy-number loss can promote proliferation by reducing expression of genes that would otherwise inhibit it; these have been termed "suppressor of tumorigenesis and/or proliferation genes" (STOP genes) [11]. These genes were previously identified in short-hairpin RNA screens for genes that tend to inhibit proliferation. In subsequent statistical analysis across more than 25 cancer types, these genes were found to be enriched in regions of recurrent deletion as determined by the Genomic Identification of Significant Targets In Cancer (GISTIC) method [12, 13].

In the second aspect, it is likely that copy-number loss often affects innocent bystander genes; the copy-number loss of these genes per se might not promote oncogenesis but instead incidentally makes cells more vulnerable to drugs targeting these genes. The model is that some of these genes already have reduced expression due to copy-number loss, and, as a consequence, would be more susceptible to inhibition by drugs. Such genes have been dubbed "Copy-number alterations Yielding Cancer Liabilities Owing to Partial losS genes" (CYCLOPS genes) [14]. These are conceptually distinct from STOP genes. The deletion of STOP genes confers a selective advantage to cancer cells, but, by contrast, the deletion of CYCLOPS genes is merely incidental, even though it presents a therapeutic opportunity. Nijhawan et al. [14] recently generated a list of probable CYCLOPS genes by associating information on cancer cell lines’ dependency on genes with information on copy-number loss of the genes in these cell lines. As determined in that previous study, a likely CYCLOPS gene was one with the property that cell lines that had copy-number loss at that gene also tended to be sensitive to the gene’s knockdown.

The criteria for CYCLOPS genes are more stringent than those for STOP genes, and this is reflected in their numbers: 55 CYCLOPS genes [14] compared with 878 STOP genes [11]. The list of CYCLOPS genes was generated on the basis of an observed association of copy-number loss with sensitivity to knockdown. By contrast, the list of STOP genes was based solely on the observation of reduced proliferation in cells in which the genes were knocked down, although subsequent analysis of STOP genes showed an aggregate statistical association with copy-number loss.

It is unknown to what extent copy-number loss of STOP genes plays a role in gastric adenocarcinoma and to what extent gastric adenocarcinomas harbor deletions of CYCLOPS genes. To investigate these questions, in the present study we used assays of approximately 906,600 SNPs in 74 tumors and matched nonmalignant tissue to delineate high-resolution, comprehensive views of copy-number loss and LOH in gastric adenocarcinomas. We then investigated the effects of copy-number loss on STOP and CYCLOPS genes in these tumors.

## Materials and methods

### Patients and samples

Primary gastric adenocarcinomas and matched nonmalignant tissue samples were obtained from Singapore Health Services with approval from the institutional review board. All samples were obtained with signed informed consent. Table S1 summarizes tumor and patient characteristics. For some of the tumors, the pathologist-estimated tumor content was very low, in some cases zero. We nevertheless analyzed these tumors because our experience has shown that pathologists, working with a portion of the surgically resected material different from that of the frozen sample from which DNA was extracted, often produce estimates of tumor content very different from those detected in DNA from the frozen portions of the tumor. Furthermore, tumors with very low tumor content can later be excluded from analysis because they have flat B-allele frequencies (BAFs) across the entire genome, as discussed in detail in "Results" and in "ASCAT profiling of allele-specific copy numbers."

### DNA extraction and hybridization

Genomic DNA from snap-frozen gastric tumors and adjacent nonmalignant gastric tissues was extracted with a Qiagen genomic DNA extraction kit. The DNA was then hybridized to Affymetrix Human Mapping SNP 6.0 arrays (Affymetrix, Santa Clara, CA, USA) according to the manufacturer’s protocol. The chips were scanned with a GeneChip scanner using the Affymetrix GeneChip Operating Software. SNP positions were represented according to the hg18 (build 36) version of the human genome reference sequence. Some of the array data were previously published in [15]. All the array data used in this work have been deposited in Gene Expression Omnibus (accession numbers GSE31168 and GSE67965).

### SNP array data preprocessing

We used Copy-number estimation using Robust Multichip Analysis version 2 (CRMA v2) [16] to extract intensity values for both alleles of each SNP from the SNP array data in the CEL files. In this process, CRMA attempts to account for (1) cross talk between alleles, (2) probe-sequence effects, and (3) the effects of the various sizes of fragments generated by restriction enzyme digestion before hybridization. We then processed each tumor and nonmalignant pair with TumorBoost [17] to increase the signal-to-noise ratio of allele-specific signals. This improved the ability of subsequent analysis to detect copy-number loss, LOH, and allelic imbalance. Matched nonmalignant samples were used as the reference to generate log_2_*R* ratios (LRRs) and BAFs for the SNPs. The LRR of a SNP is the log_2_ of the signal intensity at that SNP (summed over both alleles) in the tumor sample divided by the signal intensity in the matched nonmalignant sample. The BAF of a SNP is the proportion of the total signal in the tumor that derives from the nonreference allele [the nonreference allele is designated the B allele, whence the term “B-allele frequency” (BAF)].

### ASCAT profiling of allele-specific copy numbers

**Fig. 1:**
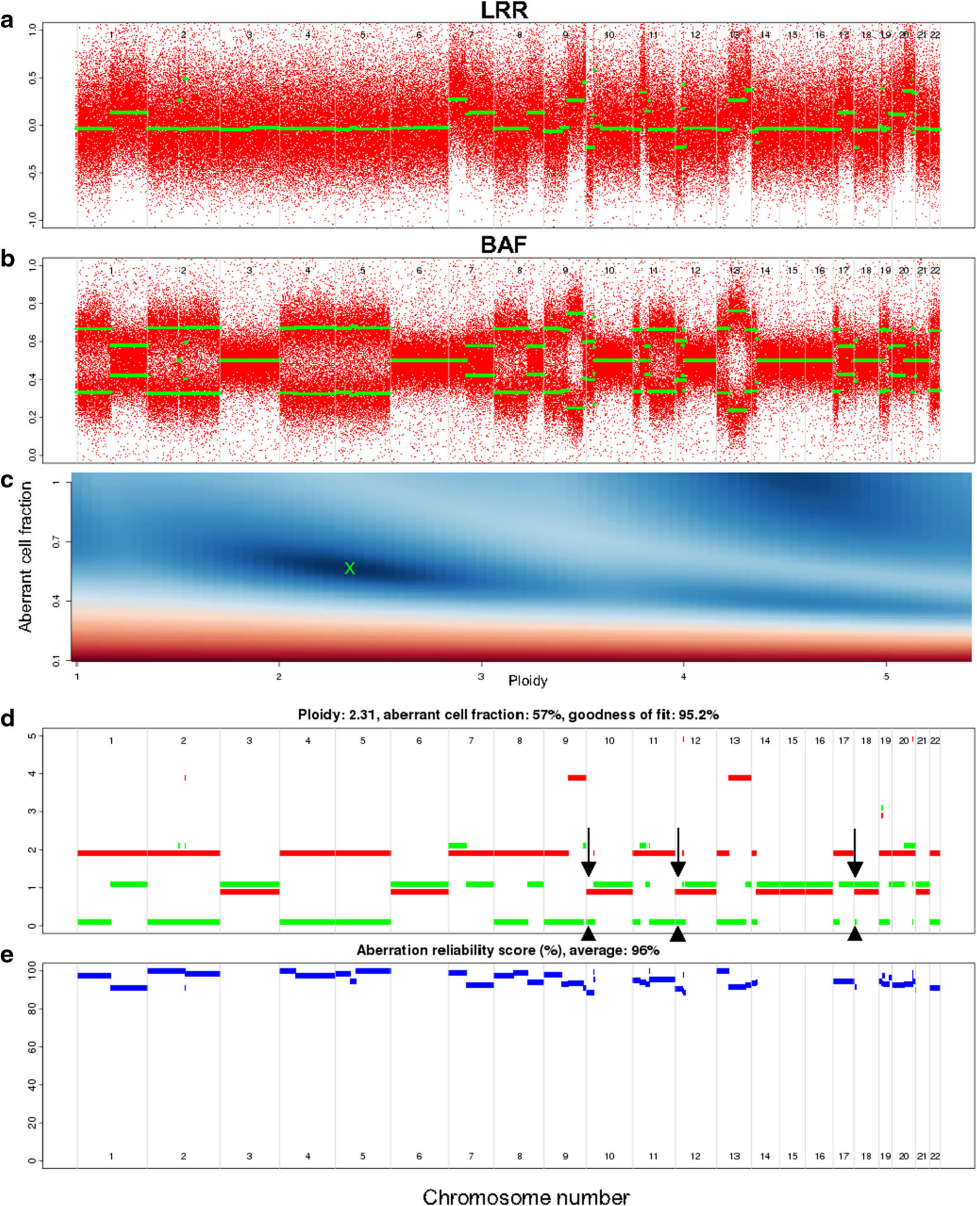
Example ASCAT profile and allele-specific copy numbers. The data are from sample 980029. **a** log_2_ *R* ratio (*LRR*). Indices of autosomal single-nucleotide polymorphisms (SNPs) that are heterozygous in the nonmalignant sample are plotted along the *x*-axis. The *y*-axis indicates LRRs of SNPs in the tumor relative to the nonmalignant sample. *Red dots* show LRRs for each informative SNP, and *green dots* show ASCAT’s segmentations. **b** B-allele frequency (*BAF*) for the SNPs plotted in **a**. *Red dots* show BAFs for each SNP and *green dots* show ASCAT’s segmentation. **c** The solution space for the two parameters “ploidy” and “aberrant cell fraction,” with the location of the chosen values marked by a *cross*. **d** ASCAT’s model of allele-specific copy numbers. The *y*-axis indicates the estimated integer chromosomal copy number. *Red lines* and *green lines* indicate the higher-copy-number and lower-copy-number chromosomal haplotypes, respectively. The lines are vertically offset slightly to avoid superimposition. **e** The ASCAT aberration reliability score, a measure of how well the model in **d** explains the segmented LRRs and BAFs. Regions of copy-number loss according to our definition (total copy number less than 0.7 times the average ploidy) can be found in **d** by looking for segments that have total copy number (sum of the two allele copy numbers given by the *green line* and the *red line*) less than 0.7 × 2.31 = 1.6. Chromosomes 10, 12, and 18 each contain a small segment with total copy number 1 (*red line* at 1 and *green line* at 0, indicated by *arrows*). The region of loss in chromosome 18 is very small, and because of the plotting it is difficult to see the gap in the *red line*. However the *green line* at copy number 0 is visible

We used Allele-Specific Copy number Analysis of Tumors (ASCAT) program [10] to estimate allele-specific copy numbers from the LRRs and BAFs while accounting for the effects of cancer-cell polyploidy and aneuploidy and the effects of the admixture of DNA from nonmalignant cells (Fig. 1). We selected ASCAT after we had evaluated several other analytical software packages, including Copy Number Analyzer for GeneChip (CNAG) [18] and Genome Alteration Print (GAP) [19]. For evaluation we used published data from a dilution series of cancer cell line DNA mixed with DNA from nonmalignant tissue from the same person [20]. We evaluated the software packages on the basis of their ability (1) to detect LOH and allelic copy numbers in tumors with a low proportion of malignant cells and (2) to be used in semiautomated fashion from the command line. Details of the evaluation are presented in [21]. We also analyzed the tumors with GAP and Global Parameter Hidden Markov Model (GPHMM) [22]. We found that GAP was often unable to detect allelic imbalance from the BAF data (Fig. S1). We believe this is because GAP is not able to use TumorBoost-processed data. GPHMM was able to use TumorBoost-processed data, but often created an implausibly large number of segments (Fig. S2). In summary, we believe that ASCAT provides the most reliable estimates of copy number, allelic imbalance, and proportion of malignant cells in the tumor DNA sample.

ASCAT was not originally designed for Affymetrix SNP array technology [10], and we made several minor modifications to it to allow it to work more effectively with Affymetrix Human Mapping SNP 6.0 arrays; patch files for modifying the original ASCAT program are available on request.

**Fig. 2:**
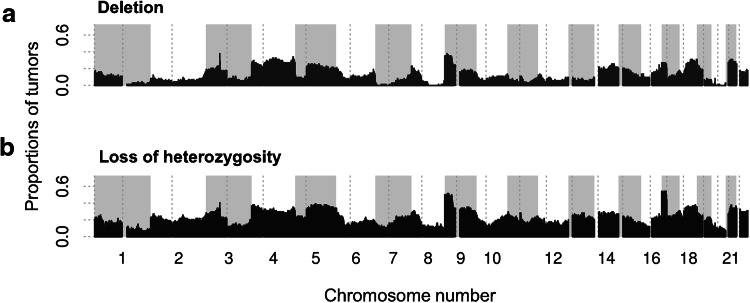
Genome-wide overview of frequencies of copy-number loss and loss of heterozygosity across 74 gastric tumors. Copy-number loss is defined as a region where the genomic copy number is less than 0.7 times the average ploidy. See Figs. S7 and S8 for detailed plots across each chromosome

The main inputs to ASCAT are LRRs and BAFs computed from a tumor and matched nonmalignant tissue as described above (Fig. 1, panels a, b). ASCAT analyzes the LRRs and BAFs for those SNPs that are heterozygous in the nonmalignant sample. These are the SNPs that are informative with respect to allelic imbalance. ASCAT segments the LRRs and BAFs to smooth random SNP-to-SNP variation. The green dots in Fig. 1a and b show the segmented LRRs and BAFs, superimposed on the original, unsegmented values, which are indicated by the red dots. After segmentation, ASCAT generates genome-wide allele-specific copy-number profiles (Fig. 1, panels d, e). The profiles (1) estimate the proportion of malignant and nonmalignant cells in the tumor sample (“aberrant cell fraction” in Fig. 1e), (2) estimate allele-specific copy numbers of chromosomal segments across the genome (Fig. 1d, red and green horizontal lines), and (3) provide reliability measures for these estimates (Fig. 1e). ASCAT also provides an average ploidy for the cancer cells in the tumor samples; this is the average of the copy numbers of informative SNPs across the genome (“ploidy” in Fig. 1d).

### List of tumor suppressor genes

We identified the tumor suppressor genes (TSGs) in Table 1 from two sources. The first was the Sanger Cancer Gene Census, an actively maintained, curated list of cancer-related genes, first described in [23], downloaded from http://cancer.sanger.ac.uk/cancergenome/assets/cancer_gene_census.tsv on May 24, 2013. The second source was the supplementary information in [24], worksheets Table S2A and Table S2B in the file http://www.sciencemag.org/content/suppl/2013/03/27/339.6127.1546.DC1/1235122TablesS1-4.xlsx. We treated a gene as a TSG if it was listed as “rec” (recessive) in the Cancer Gene Census or listed as “TSG” in [24].

**Table 1:** Copy-number alterations Yielding Cancer Liabilities Owing to Partial losS (*CYCLOPS*), suppressor of tumorigenesis and/or proliferation (*STOP*), and tumor suppressor genes in regions showing copy-number loss in at least approximately 20 % of gastric adenocarcinomas

Chromosome	Start (Mb)	End (Mb)	Size (Mb)	Maximum frequency^a^	CYCLOPS genes	Number of STOP genes	STOP genes	Tumor suppressor genes
3	21.4	31.4	10.0	0.2		3	*NR1D2*, *THRB*, *UBE2E1*	
3	42.1	61.2	19.1	0.38		7	*ARF4*, *ARIH2*, *FLNB*, *HESX1*, *HYAL2*, *PHF7*, *ZNF35*	*BAP1*, *PBRM1*, *SETD2*
4	0.03	191.2	191.2	0.34	*ETFDH*, *HPGD*, *MTHFD2L*	62	*ACSL1*, *ADH7*, *AGXT2L1*, *ALB*, *ALPK1*, *ANXA10*, *ASB5*, *BST1*, *BTC*, *CASP3*, *CASP6*, *CCNG2*, *CD38*, *CDKL2*, *CRMP1*, *CXCL6*, *DCTD*, *DDX60L*, *ELMOD2*, *ENPP6*, *EPHA5*, *FAM175A*, *FBXW7*, *FGF2*, *GAB1*, *GABRB1*, *GNPDA2*, *GPR125*, *GRK4*, *GRSF1*, *HERC3*, *HERC5*, *HTN1*, *IL8*, *LNX1*, *LRAT*, *MAPK10*, *NPY1R*, *NR3C2*, *PCGF3*, *PDHA2*, *PPAT*, *PRDM8*, *RAB33B*, *SEC31A*, *SEPSECS*, *SLC25A31*, *SPATA18*, *SPATA4*, *SPCS3*, *SRD5A3*, *TIGD2*, *TLR10*, *TLR3*, *TMEM144*, *TMEM165*, *TXK*, *UBE2K*, *UGT8*, *USP46*, *USP53*, *ZNF330*	*FBXW7*, *PHOX2B*, *TET2*
5	49.6	126.7	77.1	0.26	*ENC1*	25	*ADAMTS6*, *ANKRD55*, *AP3S1*, *BDP1*, *CARTPT*, *CENPH*, *CHD1*, *DHFR*, *ERAP2*, *FAM81B*, *FBXL17*, *FER*, *IL31RA*, *KCNN2*, *MAP3K1*, *MEF2C*, *NAIP*, *NUDT12*, *PDE4D*, *PJA2*, *RGMB*, *RNF180*, *SREK1IP1*, *SRFBP1*, *TNFAIP8*	*APC*, *MAP3K1*, *PIK3R1*
5	127.6	134.6	7.0	0.22		2	*GDF9*, *LYRM7*	
5	135.1	138.6	3.5	0.23		1	*EGR1*	
5	142.7	146.8	4.1	0.22		1	*NMHB1*	
5	155.6	173.2	17.6	0.22		2	*DOCK2*, *PTTG1*	*NPM1*
5	176.2	179.5	3.3	0.2		1	*HNRNPH1*	
8	0.1	5.0	4.9	0.23		2	*DLGAP2*, *FBXO25*	
9	0.0	46.8	46.7	0.38	*SMU1*	9	*DCAF10*, *FREM1*, *IFNA1*, *IFNA13*, *IFNW1*, *MELK*, *RLN2*, *SMU1*, *TEK*	*CDKN2A*, *DOCK8* ^b^, *FANCG*, *PAX5*, *PTPRD* ^b^
14	19.3	22.1	2.8	0.22		4	*METTL3*, *NDRG2*, *RAB2B*, *TEP1*	
14	40.6	42.2	1.6	0.22		1	*LRFN5*	
14	60.1	65.3	5.2	0.22		3	*MTHFD1*, *ZBTB1*, *ZBTB25*	
14	65.9	93.5	27.6	0.26	*EIF2B2*, *PGF*	6	*ACTN1*, *ARG2*, *ATXN3*, *FOS*, *MPP5*, *ZDHHC22*	
14	93.9	106.3	12.4	0.22		2	*CDC42BPB*, *PPP1R13B*	*DICER1*
15	18.3	21.3	3.0	0.2		0		
16	84.3	86.5	2.2	0.2		1	*FOXF1*	
17	0.0	21.3	21.3	0.27	*PAFAH1B1*	4	*ALDH3A2*, *COX10*, *CTNS*, *TP53*	*FLCN*, *MAP2K4*, *NCOR1*, *TP53*
18	24.5	26.6	2.1	0.2		0		
18	26.9	76.1	49.2	0.31		*13*	*ADNP2*, *FBXO15*, *KDSR*, *KIAA1328*, *PIK3C3*, *PMAIP1*, *RNF125*, *RNF138*, *SERPINB7*, *SMAD2*, *SMAD4*, *ST8SIA5*, *ZNF407*	*SMAD2*, *SMAD4*
19	0.2	13.3	13.1	0.24	*EEF2*, *LSM7*, *RPS15*, *SF3A2*	1	*C19orf43*	*DNM2*, *SMARCA4*, *STK11*
21	9.9	46.9	37.0	0.31		14	*ATP5J*, *BRWD1*, *BTG3*, *DNMT3L*, *GRIK1*, *HMGN1*, *IFNAR1*, *JAM2*, *LIPI*, *NRIP1*, *PTTG1IP*, *SYNJ1*, *TPTE*, *TRAPPC10*	*RUNX1*

Only regions larger than 1 Mb are shown. Figure S7 provides details. Copy-number loss is defined as a region where the genomic copy number is less than 0.7 times the average ploidy.

^a^The maximum frequency of copy-number loss within the region

^b^Possible tumor suppressor genes in gastric cancer (see the text)

### Analysis of STOP genes

STOP genes are suppressors of proliferation that were identified in a short-hairpin RNA screen for genes that retard proliferation, i.e., genes that when knocked down permit increased proliferation [11]. In our analysis, we used the most stringent criterion among several presented in [11] to select STOP genes: the genes for which at least four short-hairpin RNAs increased cell proliferation by at least fourfold. We determined the list of these genes on the basis of the data in Table S7 in [11] (878 genes). For our analysis of STOP genes, we used the Gene Set Enrichment Analysis (GSEA) Preranked software tool [25] with the “classic enrichment statistic,” i.e., the version of the enrichment statistic that uses ranks without weights. GSEAPreranked runs the analysis with a user-supplied ranked list of genes and determines if a given set of genes shows statistically significant enrichment at either end of the ranking. This is done by computation of an enrichment score for the given gene set that reflects how often members of the gene set occur at the top or bottom of the ranked list.

Our analysis examined whether, compared with other genes, STOP genes tended to have reduced copy number. We ordered the genes in increasing order of their average relative copy number across all samples, and then, to break ties, in decreasing order of the correlation coefficient between the genes’ average relative copy numbers and expression levels. In the cases of the few remaining ties, we used a random ordering. We tested this ordered list against the STOP gene set. We obtained the relative copy number of a gene in a sample by dividing the copy number of the gene in the sample by the ASCAT-determined average ploidy of the sample. We performed the analysis using several random orderings, and we report the maximum *p* value over the random orderings. Table S2 provides one such ordering.

### Gene expression data

Gene expression data were obtained from Gene Expression Omnibus (accession numbers GSE15459 and GSE34942). We used COMBAT [26] as described in [27] to remove batch effects.

### Analysis of CYCLOPS genes

CYCLOPS genes are those for which “loss correlated with a greater sensitivity to further gene suppression” [14]. For our analysis we used the list of candidate genes in Table S2 in [14] and selected the genes with a false discovery rate of less than 0.25, which was the criterion used in [14]. Fifty-five genes satisfied this criterion; the main text of [14] is apparently inconsistent in indicating 56 genes.

## Results

We initially analyzed 113 gastric tumors with their paired adjacent nonmalignant tissues using ASCAT (Table S3). For 74 of the 113 pairs, ASCAT was able to estimate allele-specific copy numbers across the genome. ASCAT was unable to estimate allele-specific copy numbers for the remaining pairs for the following reasons (Table S4): (1) excessively variable LRR data that ASCAT was unable to segment reasonably (12 tumors; Figs. S3a, S4, S5); (2) BAFs that were flat, i.e., uniformly 0.5 (25 tumors; Fig. S6a); or (3) apparently low tumor content as evidenced by very little variation in the segmented LRRs and few divergences of the BAFs from 0.5 (two tumors). We suspect that excessively variable LRRs are the result of experimental artifacts, as shown in Figs. S3, S4, and S5. We believe that very low proportions of malignant cells in the tumor samples were responsible for the BAFs that were uniformly 0.5, for the reasons described in the caption for Fig. S6. Inspection of the 74 generated ASCAT profiles revealed 12 profiles with large (more than 10 Mb) homozygous deletions, which are likely incompatible with cell survival. Therefore, these probably represent underestimates of average ploidies by ASCAT. Consequently, we adjusted these profiles by selecting the next best solution found by ASCAT at a higher average ploidy.

### Landscape of copy-number loss and LOH in gastric cancer

**Fig. 3:**
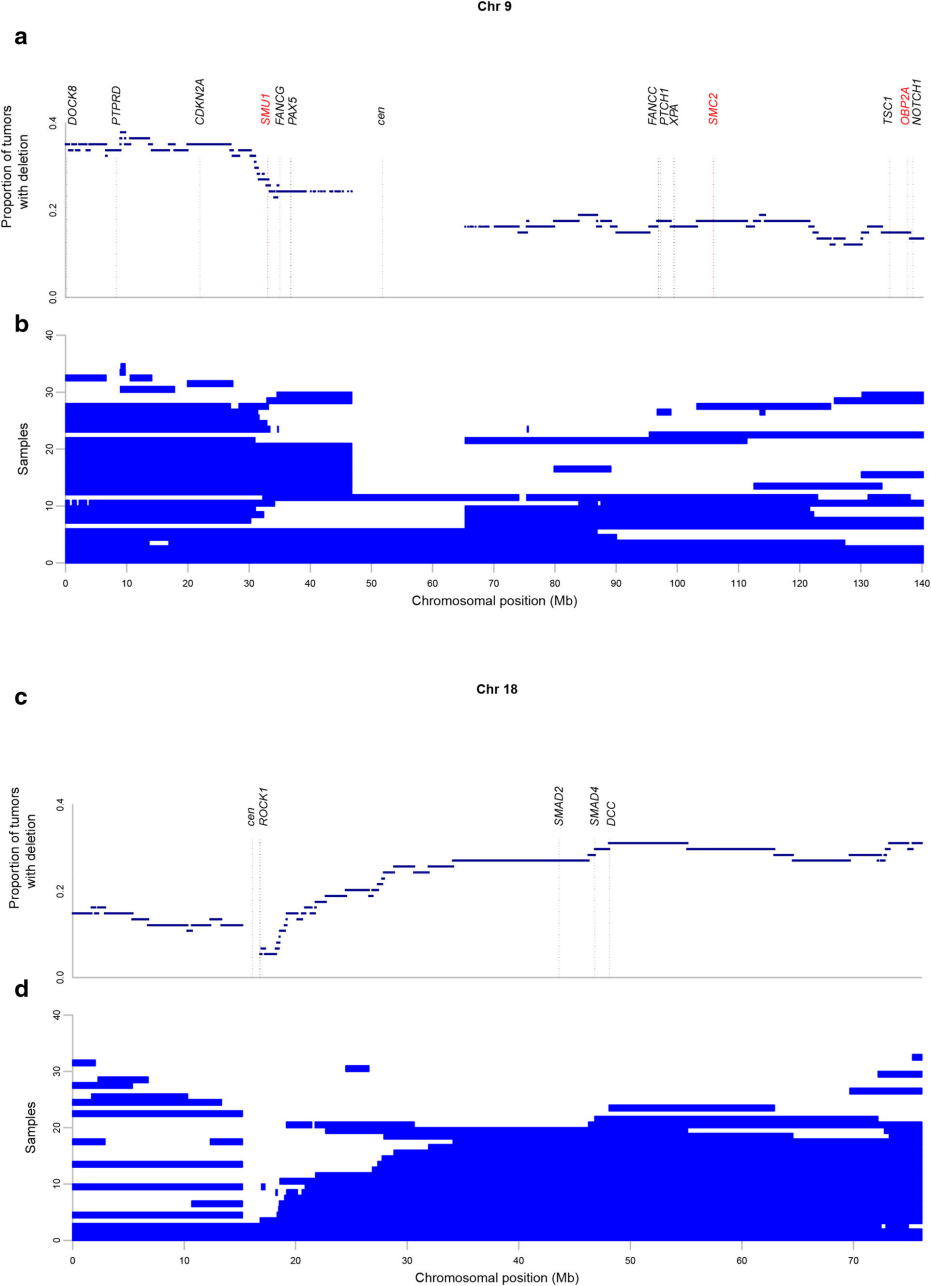
Regions of copy-number loss across chromosomes 9 and 18. **a** The proportion of tumors showing copy-number loss at each single-nucleotide polymorphism on chromosome 9, based on ASCAT’s allele-specific copy-number analysis. The locations of Copy-number alterations Yielding Cancer Liabilities Owing to Partial losS (CYCLOPS) genes (*red*) and well-established tumor suppressor genes (*black*) are indicated. **b** Regions of copy-number loss in specific tumors. **c**, **d** Analogous information for chromosome 18. Copy-number loss is defined as a region where the genomic copy number is less than 0.7 times the average ploidy. *cen* centromere

Genomic copy-number loss and LOH are pervasive in gastric cancer (Figs. 2, S7, S8, Tables 1, S5). The proportion of the genome subject to copy-number loss varies considerably from tumor to tumor, with a median of 5.5 %, and a mean of 12 % (range 0–58.5 %; Fig. S9a). In addition, an average of 22.1 % of each gastric cancer genome is subject to LOH (range 0–77.7 %). Regions of copy-number loss and LOH in individual tumors often encompass whole chromosomes, chromosome arms, or regions of tens of megabases (Figs. 2, 3, S7, S8 Table 1).

There are several large regions that are each subject to copy-number loss in at least 20 % of tumors (Table 1). One of these is a 46.7-Mb portion of 9p that contains nine STOP genes, one CYCLOPS gene, and the TSG *CDKN2A* (which encodes cyclin-dependent kinase inhibitor 2A) (Fig. 3, panels a, b). This region also contains two other genes, *PTPRD* and *DOCK8*, that have been proposed as TSGs in other cancers [28–35]. An additional large region of frequent copy-number loss affects much of the long arm of chromosome 18 in approximately 20 % of tumors and contains 13 STOP genes and two TSGs (Table 1, Fig. 3, panels c, d). Finally, much of chromosome 4 undergoes copy-number loss in many tumors, and contains 62 STOP genes and three CYCLOPS genes (Table 1, Fig. S7).

### STOP genes are enriched for copy-number loss

**Fig. 4:**
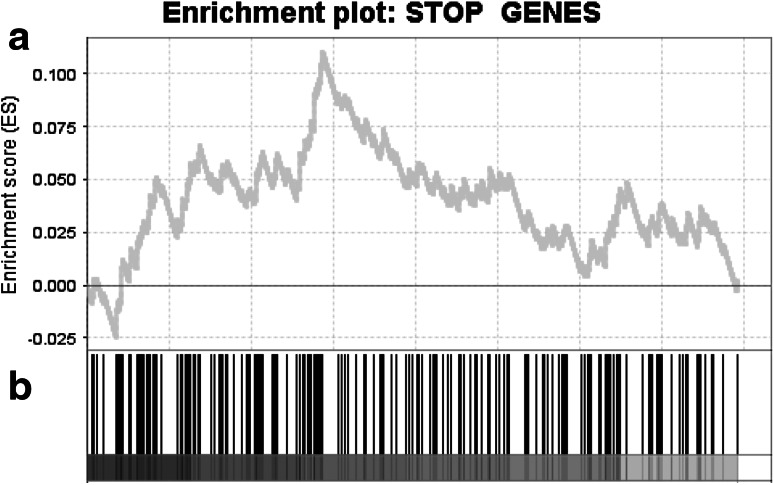
Gene Set Enrichment Analysis shows that suppressor of tumorigenesis and/or proliferation (*STOP*) genes tend to have lower average relative copy number. As discussed in the text, we restricted our attention to genes for which at least four short-hairpin RNAs increased cell proliferation by at least fourfold. **a** Running enrichment score for the STOP gene set against the list of genes ranked by their average relative copy number across all 74 samples, and then, to break ties, by the correlation coefficient between their average relative copy number and messenger RNA expression level. **b** *Vertical black lines* indicate the locations of STOP genes in the ranked list of genes

We analyzed the prevalence of deleted STOP genes in the 74 tumors and found that, on average, 91.11 STOP genes are subject to copy-number loss per tumor (median 35, range 0–452; Table S6, Fig. S9b). To test if, compared with other genes, STOP genes tend to have lower copy number in tumors, we performed a GSEAPreranked test [25] using the STOP genes as the gene set. The reasoning behind this hypothesis is that STOP genes, when reduced in copy number, would have lower expression and therefore would tend to inhibit proliferation less. Therefore, we restricted our attention to genes with significant positive correlations between average relative copy numbers and messenger RNA (mRNA) expression level. We ranked these genes on the basis of their average copy numbers relative to their tumor’s average ploidy across the 74 tumors, and then, to break ties, on the basis of the Spearman correlation coefficient between average relative copy number and expression. In this analysis, the STOP genes indeed tended to have reduced copy number (GSEA *p* < 0.02; Fig. 4). As a sanity check, we also performed an analysis based on resampling. For this, instead of using the STOP gene set (which consists of 878 genes), we randomly selected 878 genes from the genome and ran GSEAPreranked with the list of ranked genes described above. We repeated this 1000 times and then determined how many times the normalized enrichment score was higher than the one obtained when we used the STOP gene set. In our analysis this happened four times out of 1000. Therefore, the empirical *p* value is 0.004, indicating that STOP genes indeed have reduced copy number compared with the other genes in the genome.

### CYCLOPS genes are affected by copy-number loss in many tumors

CYCLOPS genes are an additional class of genes of interest in regions of copy-number loss; these are genes for which copy-number loss indicates a potential vulnerability to therapeutic inhibition [14]. Unlike the copy-number loss of a STOP gene, which is thought to promote proliferation, the copy-number loss of a CYCLOPS gene is thought to confer no advantage to the cancer cell, but rather to accidentally make the cancer more sensitive to inhibition of that gene. We found that from the total of 55 CYCLOPS genes, on average, 6.81 CYCLOPS genes were subject to copy-number loss in each tumor (median 2, range 0–39; Table S7, Fig. S9c). Forty-seven tumors had at least one CYCLOPS gene subject to copy-number loss, and 51 of the 55 CYCLOPS genes underwent copy-number loss in at least one gastric adenocarcinoma (Table S8). However, for only nine of these was the copy-number loss associated with lower mRNA levels (Table S8). On average, 1.6 of these nine genes were subject to copy-number loss per tumor (median 1, range 0–9), and 38 tumors (51.4 %) had at least one of these nine CYCLOPS genes with reduced copy number. The genes that were both subject to copy-number loss in at least 10 % of the tumors and also substantially downregulated when deleted (Table S8) are *EEF2* (which encodes eukaryotic translation elongation factor 2), *ETFDH* (which encodes electron-transferring-flavoprotein dehydrogenase), and *ENC1* (which encodes ectodermal-neural cortex 1). Visual examination of the LRRs and BAFs of these genes in several tumors strongly supports the copy-number loss assessed by ASCAT (Fig. S10).

### Correlation of copy-number loss patterns with clinical characteristics

We explored whether there were any significant correlations between the detected copy-number loss patterns and the clinical information associated with our samples. In multivariate survival analysis (Cox proportional hazards models) we found several frequent regions of copy-number loss (17p, 3p, and 5q) that were correlated with survival (Table S9). However, analysis of 212 gastric tumors from The Cancer Genome Atlas (http://cancergenome.nih.gov/) did not show a significant association between copy-number loss of these regions and survival. Possibly the biology of the tumors was different between the two patient populations, or possibly this was a chance result in our data.

We also examined associations between copy-number-loss in the regions shown in Table 1 and several other covariates. These covariates were gender, tumor stage, tumor grade, Lauren classification, and adjuvant treatment. None were significant in univariate analysis after correction for multiple hypothesis testing (Table S10). With respect to lack of association of any particular copy-number alteration with the Lauren classification, previous studies also did not detect systematic differences in copy-number alterations between the Lauren subtypes [36, 37]. We also examined association of copy-number alterations with the genomic intestinal (G-INT)/genomic diffuse (G-DIF) classification [38], and again observed no significant association after correction for multiple hypothesis testing. The G-INT/G-DIF classification is a gene-expression (mRNA)-based classification that was developed on gastric cancer cell lines and then applied to primary gastric cancer tumors. By way of background, we note that, although the G-INT subtype is enriched for Lauren intestinal-subtype tumors and the G-DIF subtype is enriched for Lauren diffuse-subtype tumors, the association is not absolute. There are diffuse-subtype tumors in the G-INT subtype and intestinal-subtype tumors in the G-DIF subtype.

## Discussion

### Limitations

Genome-wide analyses of copy-number loss and LOH are challenging owing to the mixture of malignant and nonmalignant cells in tumor samples. No standard analytical approach has emerged as the most appropriate in tumors with low proportions of malignant cells. As noted earlier, we evaluated ASCAT on a dilution series of mixed malignant and nonmalignant DNA [20, 21]. ASCAT performed well, even when analyzing tumors with low proportions of malignant cells. Nevertheless, in the current study, ASCAT was unable to analyze 39 tumors. Among these, 25 had flat BAFs. For these we believe the main issue was a very low proportion of malignant cells, for the reasons described in the caption for Fig. S6. Supporting this view, examination of the BAFs of 34 gastric cancer cell lines in the Cancer Cell Line Encyclopedia [39] revealed none with completely flat BAFs, suggesting that most gastric adenocarcinomas have at least some regions of allelic imbalance. We also note that our estimates of the proportions of tumors with LOH at each chromosome arm are statistically indistinguishable from previous estimates based on microsatellite assays (Table S11) [7], suggesting that the current analysis is correct. In addition to the 25 tumors with flat BAFs, ASCAT was unable to complete analysis of 12 tumors for which the LRRs were excessively variable. As described in Figs. S3, S4, and S5, we believe these were due to experimental artifacts.

### Candidate TSGs subject to frequent copy-number loss

We found that much of the short arm of chromosome 9 is a hot spot for copy-number loss and LOH in gastric cancer (Table 1, Figs. 3 panels a, b,  S7, S8). The TSG *CDKN2A* is located in this region and is mutated in numerous tumor types [40–42]. This gene is frequently deleted or hypermethylated in gastric cancer [43–46]. However, it is nevertheless possible that this region contains other TSGs that contribute to gastric carcinogenesis. Two genes that are promising in this regard are *PTPRD* (which encodes protein tyrosine phosphatase, receptor type, D) and *DOCK8* (which encodes dedicator of cytokinesis 8). *PTPRD* is inactivated by gene deletion or mutation in various cancers [28–33], and was previously noted to undergo LOH in gastric cancer [47]. A recent study also showed homozygous deletion of this gene in gastric cancer cell lines [48]. In our study, *PTPRD* was subject to LOH in 36 of 74 tumors and subject to copy-number loss in 25 tumors. *DOCK8* is a guanine nucleotide exchange factor that activates Rho GTPases. Homozygous deletion and reduced expression of *DOCK8* were observed in lung cancer [34, 35]. In this study, *DOCK8* was subject to LOH in 37 tumors and had reduced copy number in 26 tumors. Thus, *PTPRD* and *DOCK8* deserve more scrutiny as potential TSGs in gastric adenocarcinoma.

### Comparison with copy-number loss patterns in other cancer types

The regions most frequently subject to copy-number loss in the gastric adenocarcinomas we studied are 3p, 4, 9p, 17p, and 18q. Several other cancer types also have frequent losses in all of these regions [49]. These types include non-small-cell lung carcinoma, pancreatic adenocarcinoma, renal cell carcinoma, and esophageal carcinoma. In addition, losses of 3p and 9p are shared with head and neck cancers, malignant melanocytic neoplasia, and small cell lung and squamous cell carcinomas. Losses of 4, 17p, and 18q are also found frequently in ovarian, hepatocellular, cervical, and bladder cancers [49]. This suggests that some of the STOP gene contribution to tumorigenesis is shared across cancers. It also suggests that therapies based on CYCLOPS genes might be applicable to multiple cancer types.

### Implications of STOP genes subject to copy-number loss

We found that a substantial number of antiproliferative STOP genes were subject to copy-number loss in each tumor, and GSEAPreranked showed that STOP genes tend to have a lower copy-number compared with the other genes. The initial study of STOP genes [11] analyzed their relationship to the recurrent deletions that were originally reported in [13]. Although a large number (3131) of cancers were studied, these included only 23 gastric cancers (Supplementary Table 1 in [13]). This previous study [11] also concluded that GO genes–genes whose depletion limits proliferation—were impoverished in regions of recurrent copy-number loss. We also examined this question, but found no evidence that GO genes are impoverished in lower copy-number regions in gastric adenocarcinoma (Fig. S11).

### Implications of CYCLOPS genes subject to copy-number loss

We found that 51 of the candidate CYCLOPS genes identified in [14] were subject to copy-number loss in at least one gastric adenocarcinoma. However, for only nine of these genes was the copy-number loss in fact associated with reduced mRNA levels (Table S8), suggesting that only 16 % of the candidate CYCLOPS genes actually constitute potential therapeutic opportunities in gastric cancer. Indeed, Nijhawan et al. [14] did not examine the extent to which the candidate CYCLOPS genes were in fact downregulated when deleted. Thus, the therapeutic opportunities presented by CYCLOPS genes may be more limited than they would seem on the basis of deletions of the full set of CYCLOPS genes. Nevertheless, 38 of the tumors in the current study showed copy-number loss of at least one of the nine CYCLOPS genes for which reduced copy number was associated with reduced expression (Table S8).

Comparison of the findings of the current study with those of the previous study of CYCLOPS genes [14] suggests considerable heterogeneity in the patterns of CYCLOPS gene loss across cancer types. In the gastric tumors we studied, on average, each CYCLOPS gene was subject to copy-number loss in 12.4 % of tumors (range 0–29.7 %), which was lower than the average of 18 % (range 8–33 %) reported for 3131 tumors in [14]. These differences are reflected on a gene-by-gene basis. We take as an example the *SNRPB* gene (which encodes small nuclear ribonucleoprotein polypeptides B and B1), which was a high-ranking CYCLOPS candidate that was studied experimentally in [14]. This gene was subject to copy-number loss in 13 % of the 3131 cancers studied in [14], but had reduced copy number only once among the 74 gastric tumors we studied, a significantly lower proportion (*p* = 0.001, Fisher’s exact test). Indeed, many top-ranked CYCLOPS genes in [14] were significantly less often deleted in the gastric adenocarcinomas than in the 3131 tumors studied previously (Table S12).

## Summary

This analysis of copy-number loss in gastric adenocarcinomas showed that STOP genes tend to have a lower copy number compared with other genes, suggesting that the copy-number loss of these genes may contribute to gastric carcinogenesis. In addition, the presence of deleted and downregulated CYCLOPS genes in 51 % of the tumors suggests potential therapeutic targets in these tumors.

## Electronic supplementary material

Below is the link to the electronic supplementary material.

Supplementary material 1 (PDF 16.7 mb)

Supplementary material 2 (PDF 725 kb)

Supplementary material 3 (PDF 816 kb)

## References

[1] Ferlay J, Shin HR, Bray F, Forman D, Mathers C, Parkin DM (2010). Estimates of worldwide burden of cancer in 2008: GLOBOCAN 2008. Int J Cancer.

[2] Jemal A, Bray F, Center MM, Ferlay J, Ward E, Forman D (2011). Global cancer statistics. CA Cancer J Clin.

[3] Hartgrink HH, Jansen EP, van Grieken NC, van de Velde CJ (2009). Gastric cancer. Lancet.

[4] Uchino S, Tsuda H, Noguchi M, Yokota J, Terada M, Saito T (1992). Frequent loss of heterozygosity at the *DCC* locus in gastric cancer. Cancer Res.

[5] Sano T, Tsujino T, Yoshida K, Nakayama H, Haruma K, Ito H (1991). Frequent loss of heterozygosity on chromosomes 1q, 5q, and 17p in human gastric carcinomas. Cancer Res.

[6] Rhyu MG, Park WS, Jung YJ, Choi SW, Meltzer SJ (1994). Allelic deletions of MCC/APC and p53 are frequent late events in human gastric carcinogenesis. Gastroenterology.

[7] Yustein AS, Harper JC, Petroni GR, Cummings OW, Moskaluk CA, Powell SM (1999). Allelotype of gastric adenocarcinoma. Cancer Res.

[8] Tamura G, Sakata K, Nishizuka S, Maesawa C, Suzuki Y, Terashima M (1996). Allelotype of adenoma and differentiated adenocarcinoma of the stomach. J Pathol.

[9] Deng N, Goh LK, Wang H, Das K, Tao J, Tan IB (2012). A comprehensive survey of genomic alterations in gastric cancer reveals systematic patterns of molecular exclusivity and co-occurrence among distinct therapeutic targets. Gut.

[10] Van Loo P, Nordgard SH, Lingjaerde OC, Russnes HG, Rye IH, Sun W (2010). Allele-specific copy number analysis of tumors. Proc Natl Acad Sci U S A.

[11] Solimini NL, Xu Q, Mermel CH, Liang AC, Schlabach MR, Luo J (2012). Recurrent hemizygous deletions in cancers may optimize proliferative potential. Science.

[12] Beroukhim R, Getz G, Nghiemphu L, Barretina J, Hsueh T, Linhart D (2007). Assessing the significance of chromosomal aberrations in cancer: methodology and application to glioma. Proc Natl Acad Sci U S A.

[13] Beroukhim R, Mermel CH, Porter D, Wei G, Raychaudhuri S, Donovan J (2010). The landscape of somatic copy-number alteration across human cancers. Nature.

[14] Nijhawan D, Zack TI, Ren Y, Strickland MR, Lamothe R, Schumacher SE (2012). Cancer vulnerabilities unveiled by genomic loss. Cell.

[15] Deng N, Goh LK, Wang H, Das K, Tao J, Tan IB (2012). A comprehensive survey of genomic alterations in gastric cancer reveals systematic patterns of molecular exclusivity and co-occurrence among distinct therapeutic targets. Gut.

[16] Bengtsson H, Wirapati P, Speed TP (2009). A single-array preprocessing method for estimating full-resolution raw copy numbers from all Affymetrix genotyping arrays including GenomeWideSNP 5 & 6. Bioinformatics.

[17] Bengtsson H, Neuvial P, Speed TP (2010). TumorBoost: normalization of allele-specific tumor copy numbers from a single pair of tumor-normal genotyping microarrays. BMC Bioinformatics.

[18] Nannya Y, Sanada M, Nakazaki K, Hosoya N, Wang L, Hangaishi A (2005). A robust algorithm for copy number detection using high-density oligonucleotide single nucleotide polymorphism genotyping arrays. Cancer Res.

[19] Popova T, Manie E, Stoppa-Lyonnet D, Rigaill G, Barillot E, Stern MH (2009). Genome Alteration Print (GAP): a tool to visualize and mine complex cancer genomic profiles obtained by SNP arrays. Genome Biol.

[20] Rasmussen M, Sundstrom M, Kultima HG, Botling J, Micke P, Birgisson H (2011). Allele-specific copy number analysis of tumor samples with aneuploidy and tumor heterogeneity. Genome Biol.

[21] Wu Y (2013). Genome-wide analysis of loss of heterozygosity and discovery of novel tumor suppressor genes in gastric cancer.

[22] Li A, Liu Z, Lezon-Geyda K, Sarkar S, Lannin D, Schulz V (2011). GPHMM: an integrated hidden Markov model for identification of copy number alteration and loss of heterozygosity in complex tumor samples using whole genome SNP arrays. Nucleic Acids Res.

[23] Futreal PA, Coin L, Marshall M, Down T, Hubbard T, Wooster R (2004). A census of human cancer genes. Nat Rev Cancer.

[24] Vogelstein B, Papadopoulos N, Velculescu VE, Zhou S, Diaz LA, Kinzler KW (2013). Cancer genome landscapes. Science.

[25] Subramanian A, Tamayo P, Mootha VK, Mukherjee S, Ebert BL, Gillette MA (2005). Gene set enrichment analysis: a knowledge-based approach for interpreting genome-wide expression profiles. Proc Natl Acad Sci U S A.

[26] Johnson WE, Li C, Rabinovic A (2007). Adjusting batch effects in microarray expression data using empirical Bayes methods. Biostatistics.

[27] Lei Z, Tan IB, Das K, Deng N, Zouridis H, Pattison S (2013). Identification of molecular subtypes of gastric cancer with different responses to PI3-kinase inhibitors and 5-fluorouracil. Gastroenterology.

[28] Purdie KJ, Lambert SR, Teh MT, Chaplin T, Molloy G, Raghavan M (2007). Allelic imbalances and microdeletions affecting the *PTPRD* gene in cutaneous squamous cell carcinomas detected using single nucleotide polymorphism microarray analysis. Genes Chromosomes Cancer.

[29] Kohno T, Otsuka A, Girard L, Sato M, Iwakawa R, Ogiwara H (2010). A catalog of genes homozygously deleted in human lung cancer and the candidacy of *PTPRD* as a tumor suppressor gene. Genes Chromosomes Cancer.

[30] Solomon DA, Kim JS, Cronin JC, Sibenaller Z, Ryken T, Rosenberg SA (2008). Mutational inactivation of *PTPRD* in glioblastoma multiforme and malignant melanoma. Cancer Res.

[31] Veeriah S, Brennan C, Meng S, Singh B, Fagin JA, Solit DB (2009). The tyrosine phosphatase *PTPRD* is a tumor suppressor that is frequently inactivated and mutated in glioblastoma and other human cancers. Proc Natl Acad Sci U S A.

[32] Giefing M, Zemke N, Brauze D, Kostrzewska-Poczekaj M, Luczak M, Szaumkessel M (2011). High resolution ArrayCGH and expression profiling identifies *PTPRD* and *PCDH17*/*PCH68* as tumor suppressor gene candidates in laryngeal squamous cell carcinoma. Genes Chromosomes Cancer.

[33] Nair P, De Preter K, Vandesompele J, Speleman F, Stallings RL (2008). Aberrant splicing of the *PTPRD* gene mimics microdeletions identified at this locus in neuroblastomas. Genes Chromosomes Cancer.

[34] Takahashi K, Kohno T, Ajima R, Sasaki H, Minna JD, Fujiwara T (2006). Homozygous deletion and reduced expression of the *DOCK8* gene in human lung cancer. Int J Oncol.

[35] Kang JU, Koo SH, Kwon KC, Park JW (2010). Frequent silence of chromosome 9p, homozygous *DOCK8*, *DMRT1* and *DMRT3* deletion at 9p24.3 in squamous cell carcinoma of the lung. Int J Oncol.

[36] Noguchi T, Wirtz HC, Michaelis S, Gabbert HE, Mueller W (2001). Chromosomal imbalances in gastric cancer. Correlation with histologic subtypes and tumor progression. Am J Clin Pathol.

[37] Panani AD, Ferti AD, Avgerinos A, Raptis SA (2004). Numerical aberrations of chromosome 8 in gastric cancer detected by fluorescence in situ hybridization. Anticancer Res.

[38] Tan IB, Ivanova T, Lim KH, Ong CW, Deng N, Lee J (2011). Intrinsic subtypes of gastric cancer, based on gene expression pattern, predict survival and respond differently to chemotherapy. Gastroenterology.

[39] Barretina J, Caponigro G, Stransky N, Venkatesan K, Margolin AA, Kim S (2012). The cancer cell line encyclopedia enables predictive modelling of anticancer drug sensitivity. Nature.

[40] Cachia AR, Indsto JO, McLaren KM, Mann GJ, Arends MJ (2000). *CDKN2A* mutation and deletion status in thin and thick primary melanoma. Clin Cancer Res.

[41] Foulkes WD, Flanders TY, Pollock PM, Hayward NK (1997). The *CDKN2A* (p16) gene and human cancer. Mol Med.

[42] Lang JC, Tobin EJ, Knobloch TJ, Schuller DE, Bartynski KJ, Mountain RE (1998). Frequent mutation of p16 in squamous cell carcinoma of the head and neck. Laryngoscope.

[43] Muscarella P, Melvin WS, Fisher WE, Foor J, Ellison EC, Herman JG (1998). Genetic alterations in gastrinomas and nonfunctioning pancreatic neuroendocrine tumors: an analysis of p16/MTS1 tumor suppressor gene inactivation. Cancer Res.

[44] Wu MS, Lin YW, Sheu JC, Wang HP, Wang JT, Shun CT (1996). Intragenic homozygous deletions of MTS1 gene in gastric cancer in Taiwan. Jpn J Cancer Res.

[45] Zhao GH, Li TC, Shi LH, Xia YB, Lu LM, Huang WB (2003). Relationship between inactivation of p16 gene and gastric carcinoma. World J Gastroenterol.

[46] Tang S, Luo H, Yu J, Yang D, Shu J (2003). Relationship between alterations of p16INK4a and p14ARF genes of *CDKN2A* locus and gastric carcinogenesis. Chin Med J (Engl)..

[47] Tada M, Kanai F, Tanaka Y, Sanada M, Nannya Y, Tateishi K (2010). Prognostic significance of genetic alterations detected by high-density single nucleotide polymorphism array in gastric cancer. Cancer Sci.

[48] Lee B, Yoon K, Lee S, Kang JM, Kim J, Shim SH (2015). Homozygous deletions at 3p22, 5p14, 6q15, and 9p21 result in aberrant expression of tumor suppressor genes in gastric cancer. Genes Chromosomes Cancer.

[49] Baudis M (2007). Genomic imbalances in 5918 malignant epithelial tumors: an explorative meta-analysis of chromosomal CGH data. BMC Cancer.

