# Supplementary material for: Abundant copy-number loss of CYCLOPS and STOP genes in gastric adenocarcinoma
Source: Gastric Cancer. 2015 Jul 24;19:453–65. doi: 10.1007/s10120-015-0514-z (PMC4824836; doi:10.1007/s10120-015-0514-z)
Supplement: Supplementary file 1 — Supplementary material 1 (PDF 16.7 mb) [file 10120_2015_514_MOESM1_ESM.pdf]

Original article

Abundant copy-number loss of CYCLOPS and STOP genes in gastric adenocarcinoma

Ioana Cutcutache*,[1,2], Alice Yingting Wu*,[1, 2,3], Yuka Suzuki[1,2], John Richard McPherson[1,2], Zhengdeng Lei[1,2], Niantao Deng[1,4], Shenli Zhang[1], Wai Keong Wong[5], Khee Chee Soo[5,6], Weng Hoong Chan[5], London Lucien Ooi[5,6], Roy Welsch[3,7], Patrick Tan[1,3,8,9], Steven G. Rozen[1, 2,3]

* These authors contributed equally to this manuscript.

[1] Program in Cancer and Stem Cell Biology, Duke-NUS Graduate Medical School, Singapore

[2] Centre for Computational Biology, Duke-NUS Graduate Medical School, Singapore

[3] Computation and Systems Biology, Singapore-MIT Alliance

[4] NUS Graduate School for Integrative Science and Engineering, National University of Singapore, Singapore

[5] Department of General Surgery, Singapore General Hospital

[6] Division of Surgical Oncology, National Cancer Centre Singapore

[7] Engineering Systems Division and Sloan School of Management, Massachusetts Institute of Technology, MA, USA

[8] Duke-NUS Genome Biology Facility, Duke-NUS Graduate Medical School, Singapore

[9] Genome Institute of Singapore, A*STAR, Singapore

Correspondence: Patrick Tan (gmstanp@duke-nus.edu.sg), Steven Rozen (steve.rozen@duke-nus.edu.sg, +65 9857 3213)

Supplementary Figures

Fig. S1. GAP was not able to detect all allelic imbalance. Data from sample 990228. (a) BAF profile generated by GAP [1]; the red rectangles indicate locations where GAP was not able to detect allelic imbalance. (b) Copy number profile generated by GAP. (c) LRR profile generated by GAP. (d) BAF profile generated by ASCAT; the allelic imbalance is correctly detected.

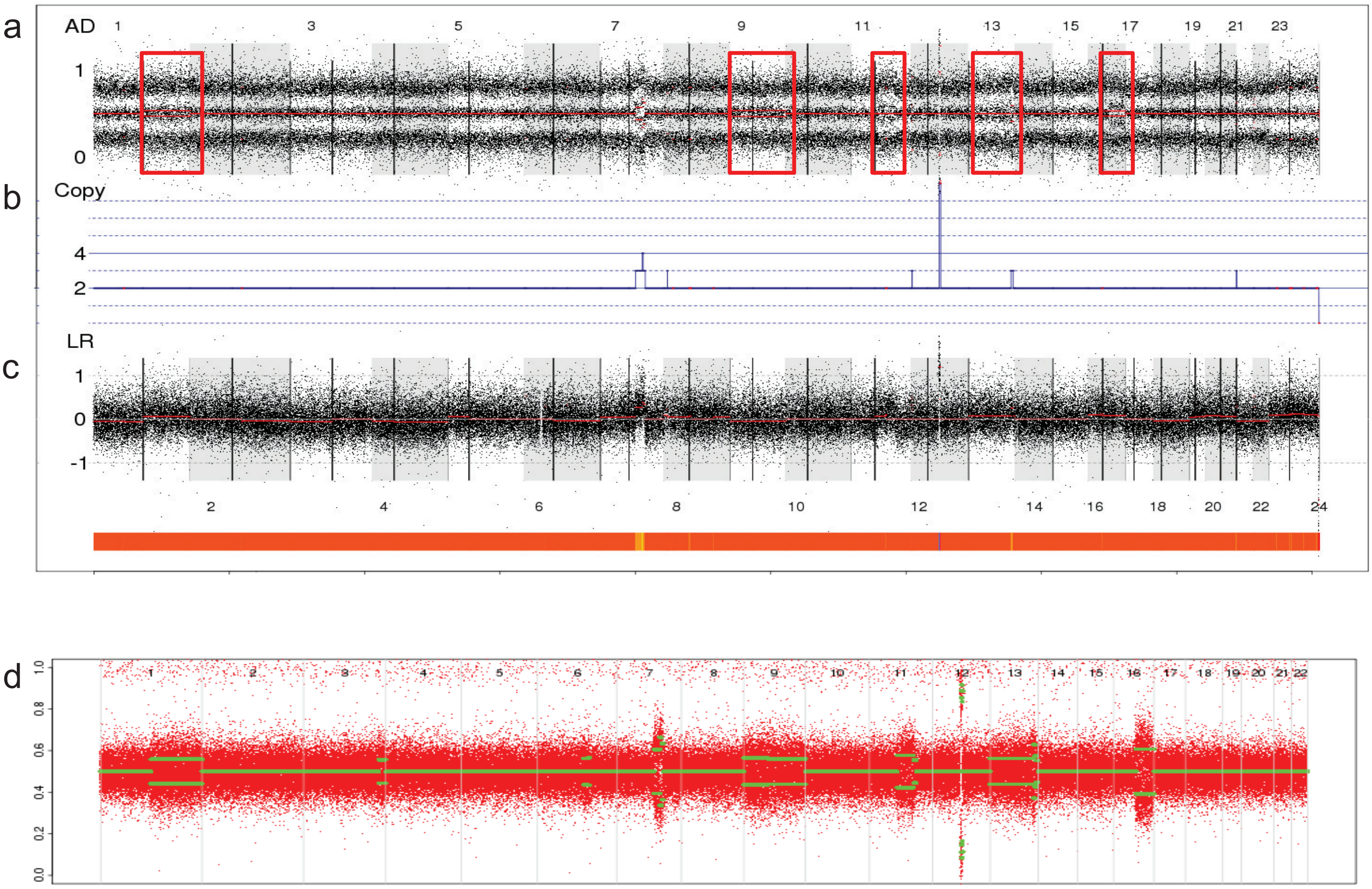

Fig. S2. GPHMM tended to create too many segments. Data from sample 57701999. (a) Copy number profile generated by GPHMM [2]. (b) LOH status profile generated by GPHMM showed implausibly frequent changes of LOH status.

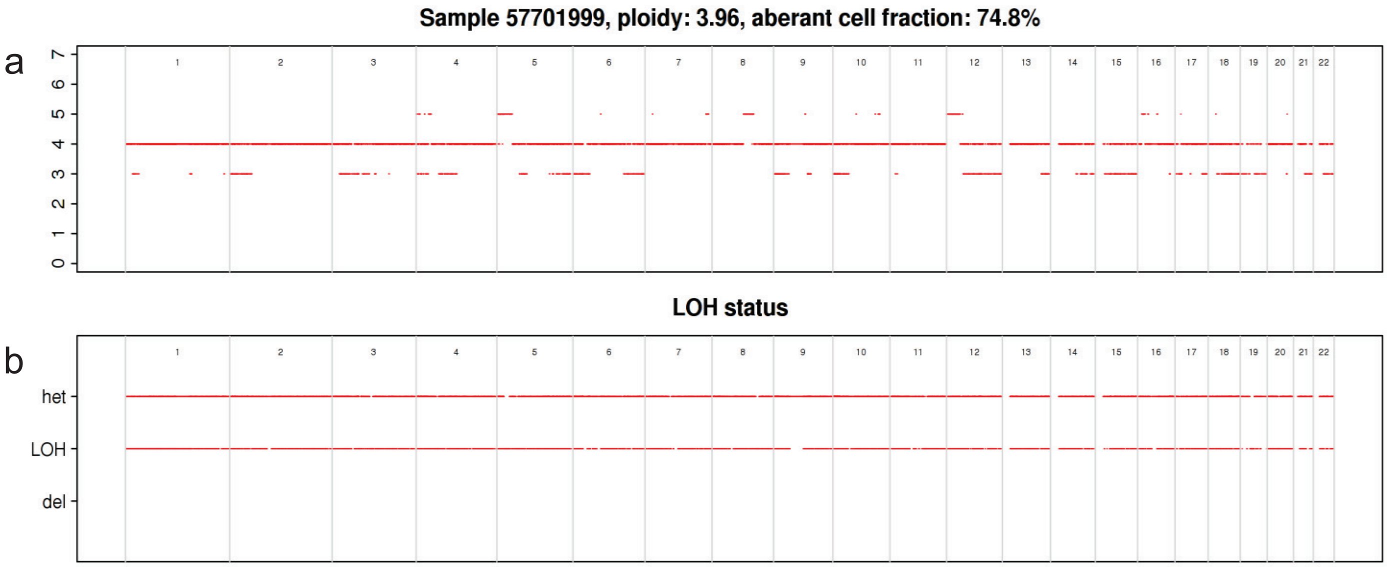

Fig. S3. Tumors that ASCAT was unable to analyze because of excessively variable LRR are likely due to experimental artifacts. (a) LRR (log R ratio) and BAF (B allele frequency) from ASCAT. (b) Relationship between standard deviations of segmented BAF and segmented LRR. In most tumors for which ASCAT was able to generate an allele-specific-copy-number profile, the standard deviations of the segmented LRRs and BAFs are tightly correlated (blue circles). For the samples with excessively variable LRRs, however, the standard deviations of the LRRs are relatively higher than those of the BAFs (red squares). This reflects the fact that, in these tumors, the LRRs change frequently along the genome without corresponding changes in the BAFs (panel a). Most true changes in copy number along the genome would also result in a change in BAF. Therefore the high variability of the LRRs across every chromosome without corresponding variability in BAF likely stems from experimental artifacts. Figs. S4 and S5 show a pair of Affymetrix SNP6 hybridizations from the same cell line, with one hybridization showing excessively variable LRR and the other not.

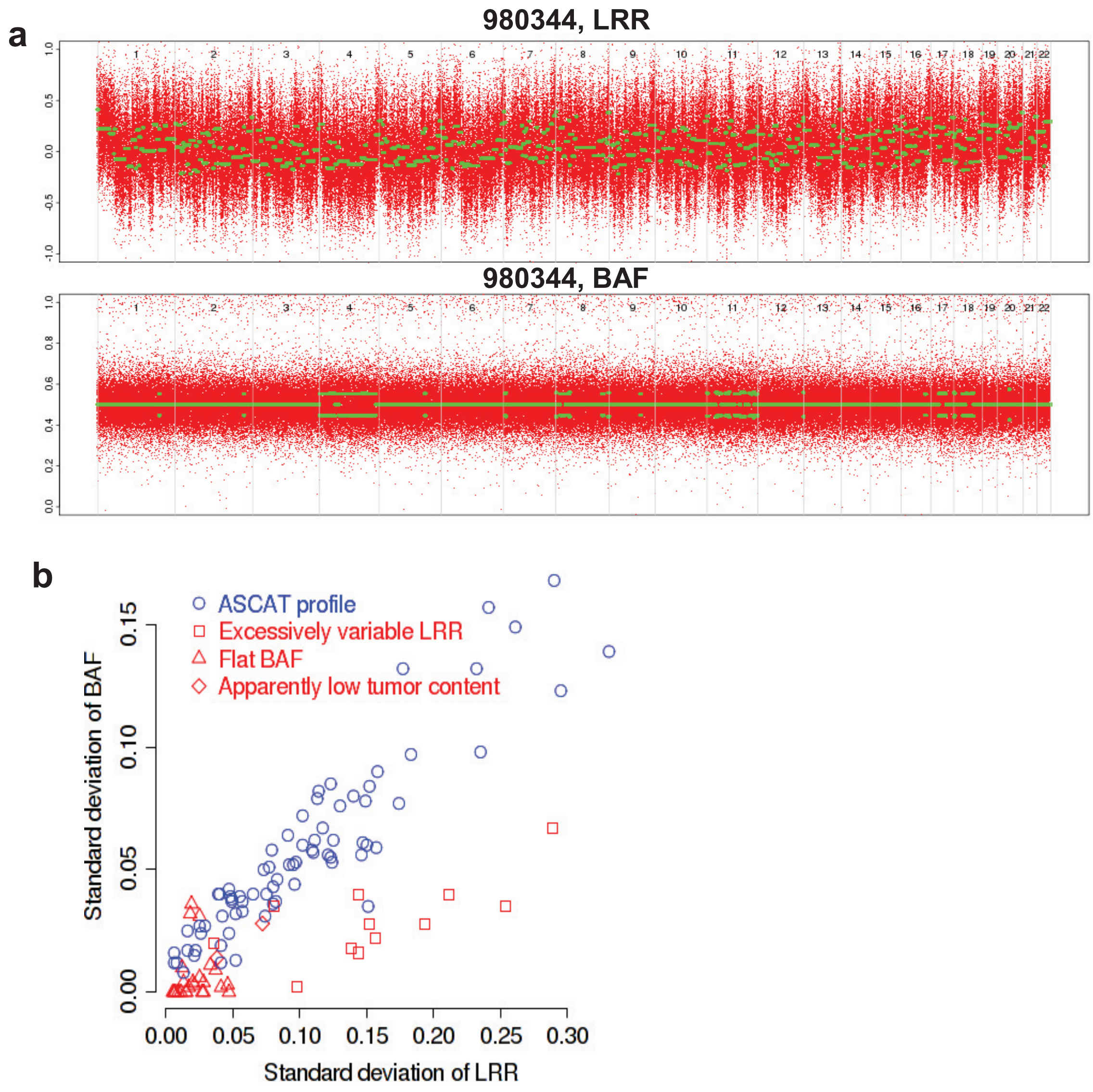

Fig. S4. Gastric cancer cell line HGC27, good hybridization;
BAF, LRR, and GAP (Genome Alteration Print) from the GAP software [1].
Data from the Cancer Cell Line Encyclopedia (http://www.broadinstitute.org/ccle/home [3]). The LRR is relatively smooth compared to Fig. S5.

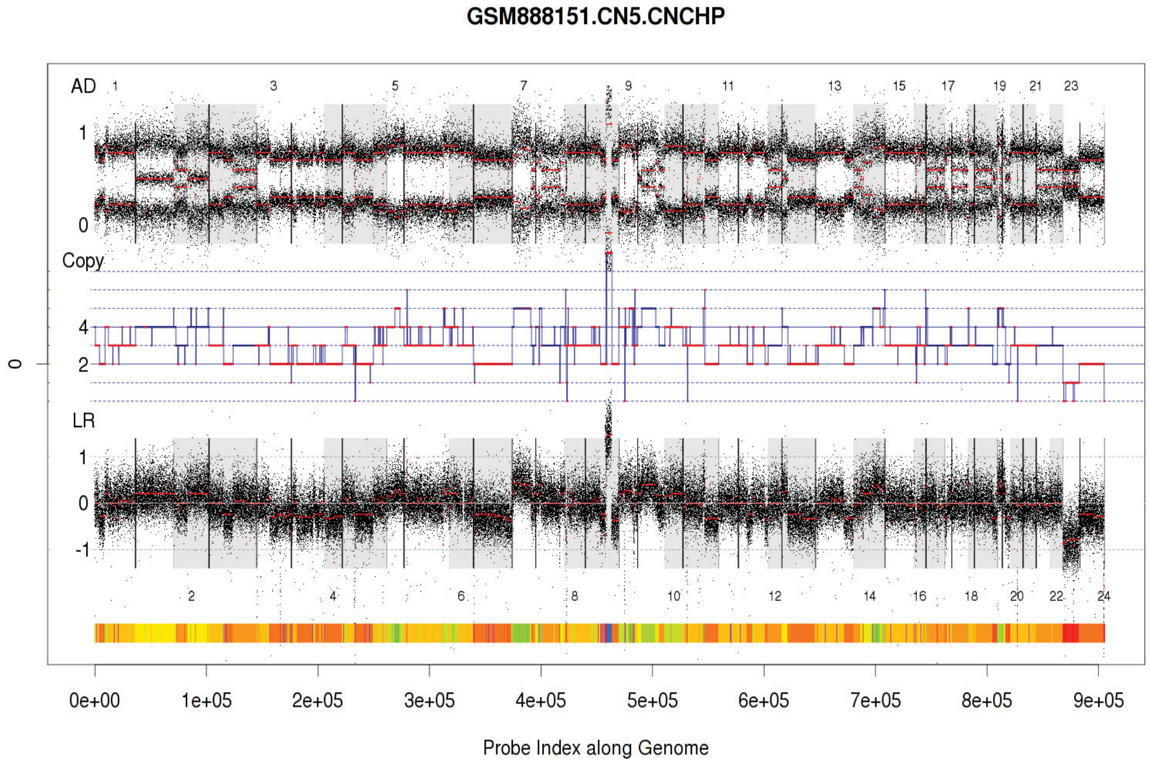

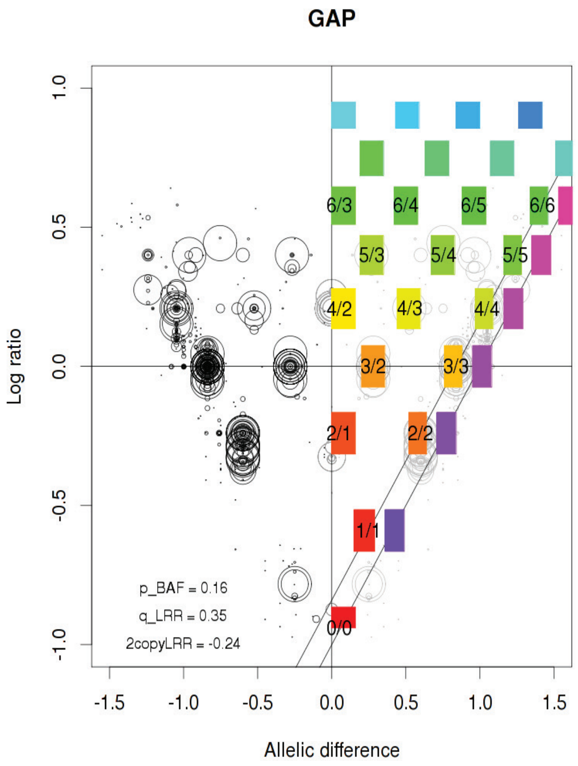

Summary and Centromeres

Break counts = 488
Chromosome counts = 72
Centromere counts = 130
DNA index = 1.5
Centromere breaks = 10

Ploidy detected = 4
% base state = 7
% '1' copy loss = 23
% accuired homo = 69

Chr	Copy	B allele	SNPs	Chr	Copy	B allele	SNPs
1 p	3	3	4636	12 p	3	2	5279
1 q	4	2	8446	12 q	5	3	449
2 p	4	2	166	13 q	3	3	1483
2 q	3	3	12429	14 q	3	2	2619
3 p	1	0	140	15 q	3	3	728
3 q	2	0	205	16 p	3	3	102
4 p	2	2	6876	16 q	3	3	94
4 q	3	3	1308	17 p	3	2	213
5 p	4	4	3757	17 q	6	5	58
5 q	3	3	2412	18 p	3	3	4773
6 p	3	3	391	18 q	3	2	775
6 q	3	0	30	19 p	4	3	233
7 p	3	2	1701	19 q	4	4	59
7 q	4	3	1370	20 p	3	3	4745
8 p	3	3	8718	20 q	3	3	5449
8 q	3	3	1943	21 q	2	1	123
9 p	4	2	27	22 q	3	2	2007
9 q	4	2	3665	23 p	2	2	791
10 p	4	4	10088	23 q	2	2	9036
10 q	3	3	2490	24 p	0	0	81
11 p	3	3	5813	24 q	0	0	176
11 q	3	3	179				

Fig. S5. Gastric cancer cell line HGC27, hybridization with experimental artifacts; BAF, LRR, and GAP (Genome Alteration Print) from the GAP software [1]. Data from our laboratory's hybridization. The LRR is excessively variable compared to Fig. S4. This suggests that excessively variable LRR is due to an experimental artifact, and does not reflect underlying variability in genomic copy number.

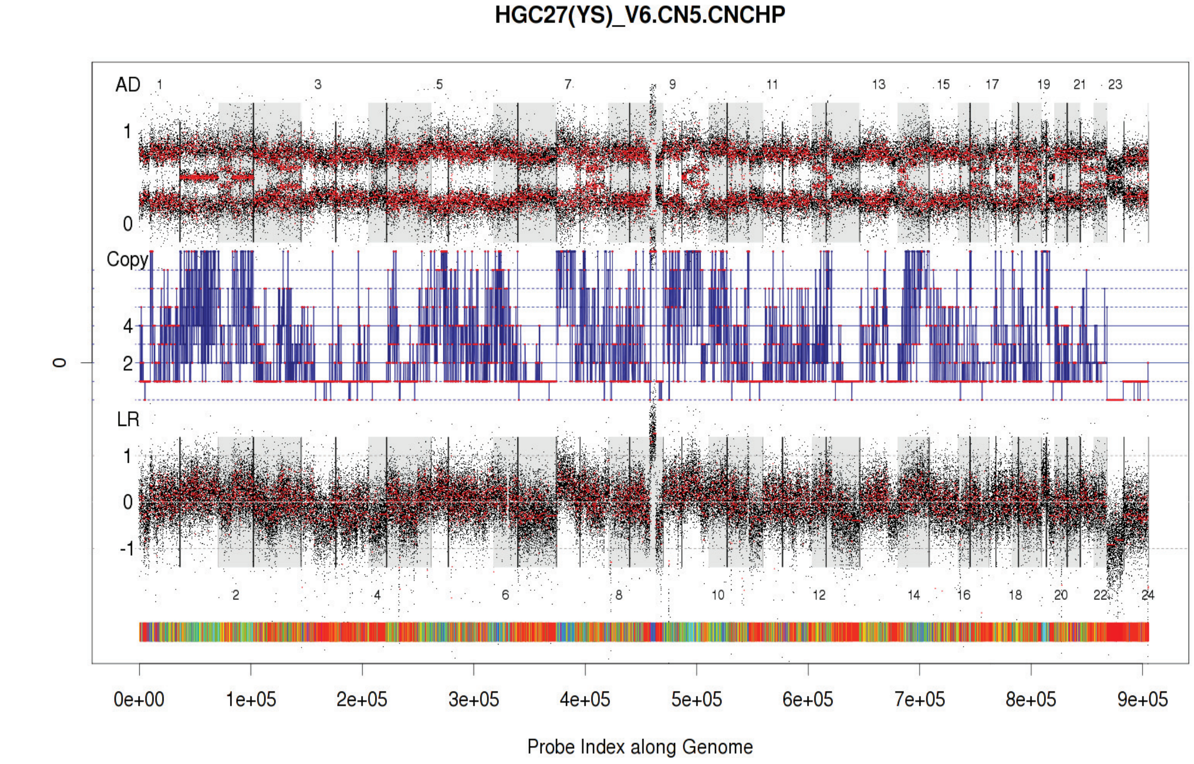

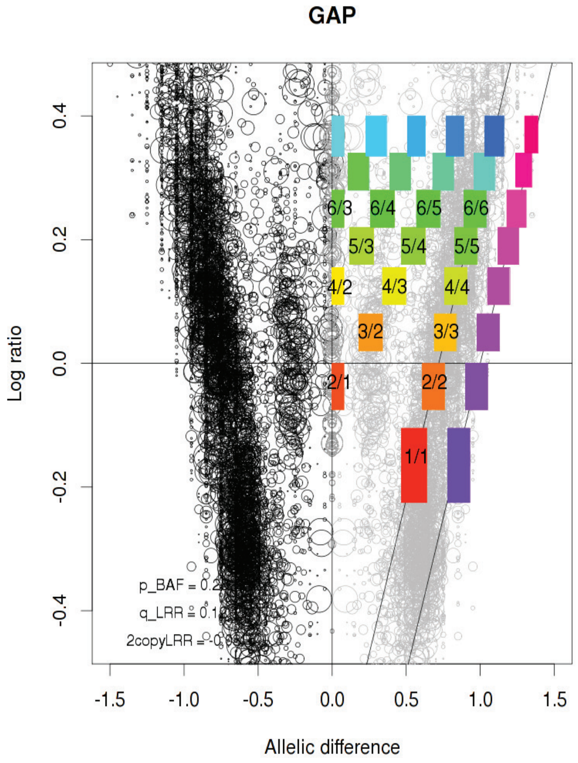

Summary and Centromeres

Break counts = 5104
Chromosome counts = 84.5
Centromere counts = 153
DNA index = 1.61
Centromere breaks = 12

Ploidy detected = 4
% base state = 1
% '1' copy loss = 6
% accuired homo = 78

Chr	Copy	B allele	SNPs	Chr	Copy	B allele	SNPs
1 p	4	4	17	12 p	1	0	17
1 q	5	4	65	12 q	8	8	194
2 p	7	5	30	13 q	5	5	56
2 q	5	5	200	14 q	3	2	751
3 p	1	1	324	15 q	4	3	49
3 q	2	2	26	16 p	4	4	91
4 p	2	2	208	16 q	5	5	205
4 q	1	1	121	17 p	3	3	134
5 p	4	4	86	17 q	8	4	88
5 q	6	6	105	18 p	6	6	112
6 p	1	1	21	18 q	3	2	731
6 q	3	3	31	19 p	4	4	16
7 p	4	3	26	19 q	6	6	80
7 q	5	4	29	20 p	4	4	439
8 p	1	1	17	20 q	4	4	291
8 q	2	0	24	21 q	2	1	421
9 p	8	4	24	22 q	2	1	392
9 q	8	4	21	23 p	1	1	791
10 p	4	4	38	23 q	1	1	1821
10 q	4	4	395	24 p	0	0	81
11 p	1	1	77	24 q	0	0	176
11 q	1	1	134				

Fig. S6. Low tumor content in unanalyzable tumors. (a) ASCAT output for an example tumor that had a flat BAF. (b) The samples that ASCAT was not able to analyze had lower pathologist-estimated tumor content compared to samples that ASCAT was able to analyze (p = 0.034, one-sided Wilcoxon rank-sum test, Table S1). (c) Furthermore, the minimum malignant cell proportion that ASCAT was able to estimate was 18%, suggesting that it is not able to analyze samples with proportions lower than this. These two observations implicate low proportions of malignant cells as a major reason that ASCAT could not estimate allele-specific copy number for some samples. Lending additional evidence to this inference, as discussed in the main text, our analysis of data from 34 gastric cancer cell lines from the Cancer Cell Line Encyclopedia [3] revealed none with completely flat BAFs.

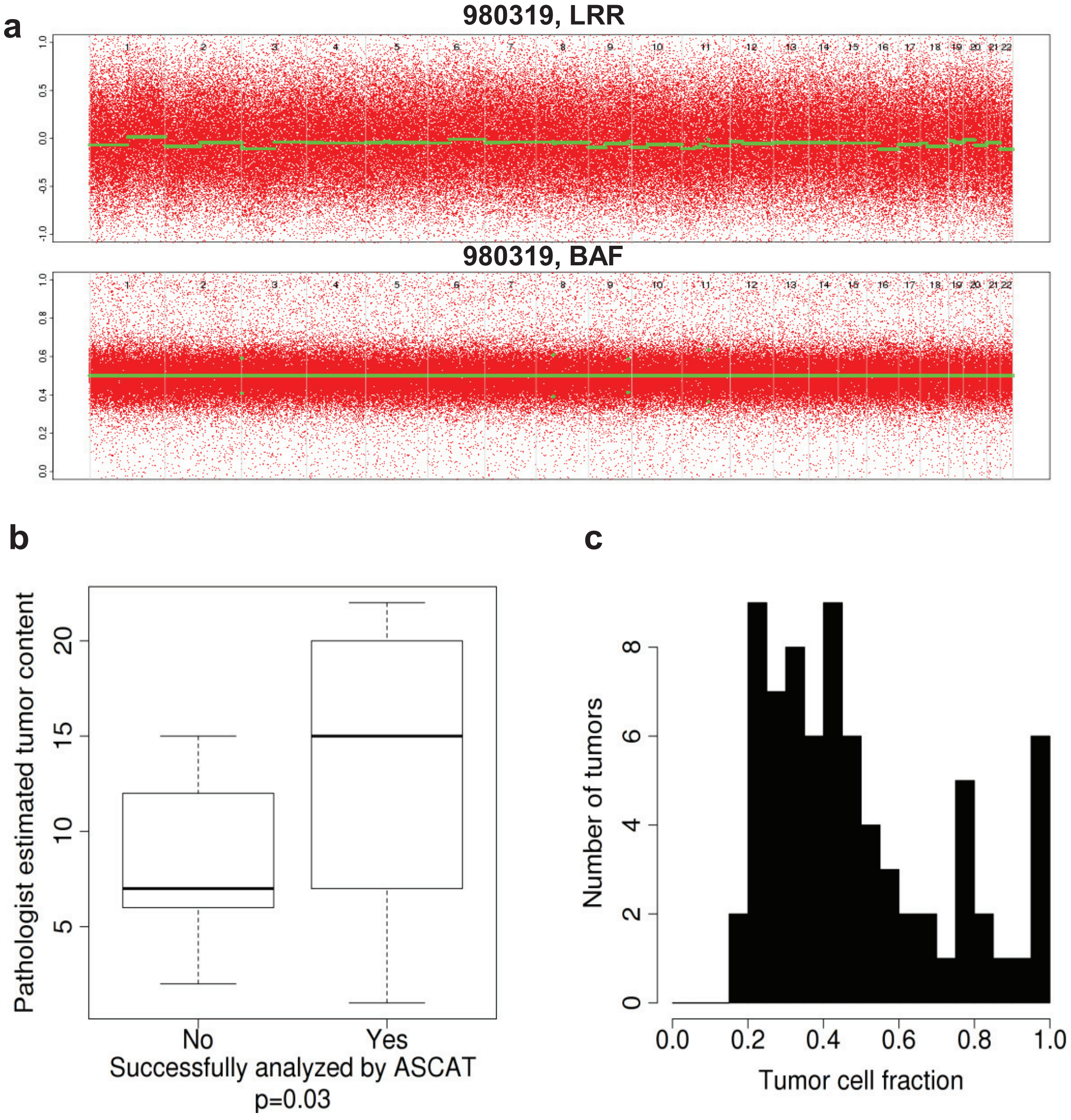

Fig. S7. Landscape of copy-number loss by chromosome.

For each chromosome, the proportion of tumors showing copy-number loss at each SNP, based on ASCAT's allele-specific copy number analysis (upper panels) and regions of copy-number loss in specific tumors (lower panels). The genes marked in black color are tumor suppressor genes, while the genes marked in red color are CYCLOPS genes. Copy-number loss is defined as a region where genomic copy number is < 0.7 times the average ploidy.

Chr 1

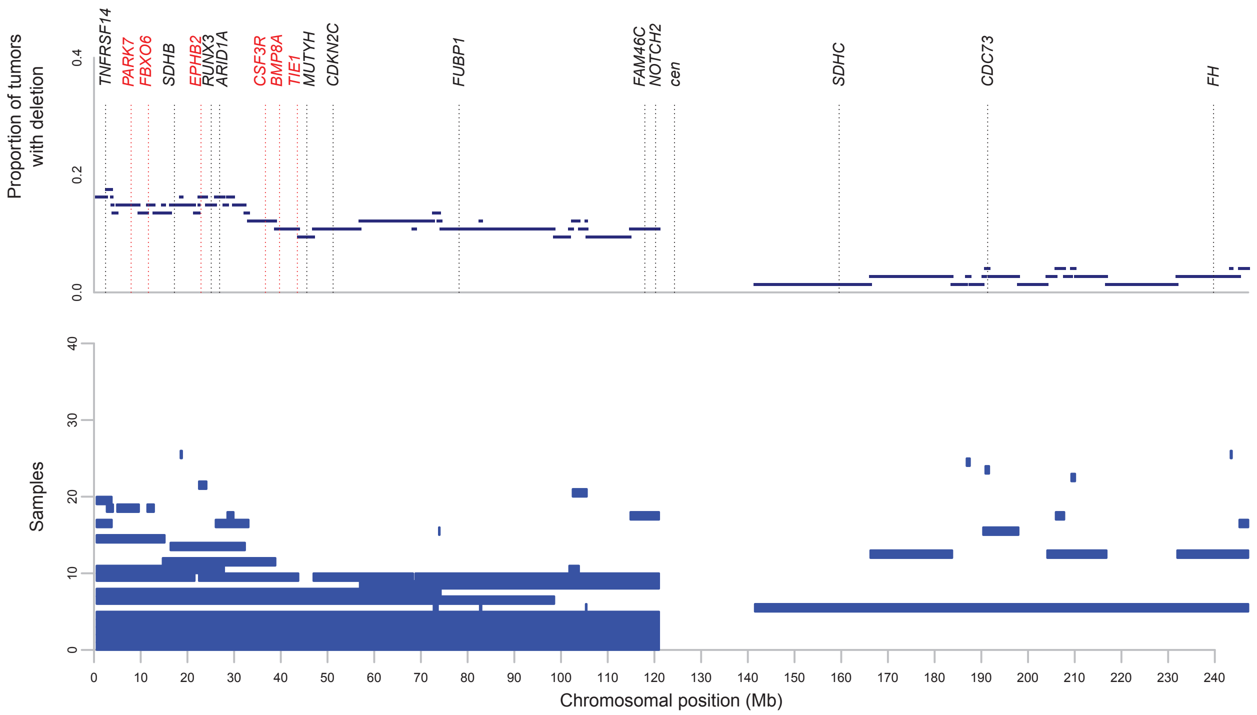
TNFRSF14
PARK7
FBXO6
SDHB
EPHB2
RUNX3
ARID1A
CSF3R
BMP8A
TIE1
MUTYH
CDKN2C
FUBP1
FAM46C
NOTCH2
cen
SDHC
CDC73
FH
Proportion of tumors with deletion
0.0
0.2
0.4
Samples
0
10
20
30
40
0 10 20 30 40 50 60 70 80 90 100 110 120 130 140 150 160 170 180 190 200 210 220 230 240
Chromosomal position (Mb)

Chr 2

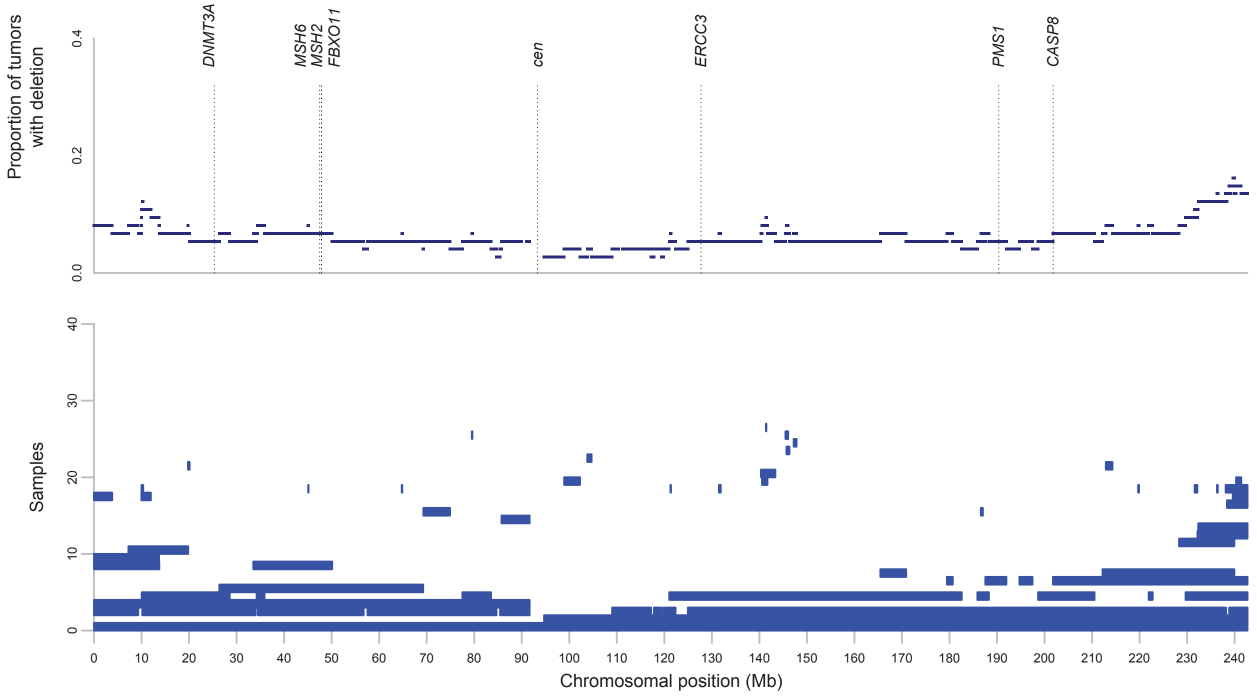
DNMT3A
MSH6
MSH2
FBXO11
cen
ERCC3
PMS1
CASP8
Proportion of tumors with deletion
0.0
0.2
0.4
Samples
0
10
20
30
40
0 10 20 30 40 50 60 70 80 90 100 110 120 130 140 150 160 170 180 190 200 210 220 230 240
Chromosomal position (Mb)

Chr 3

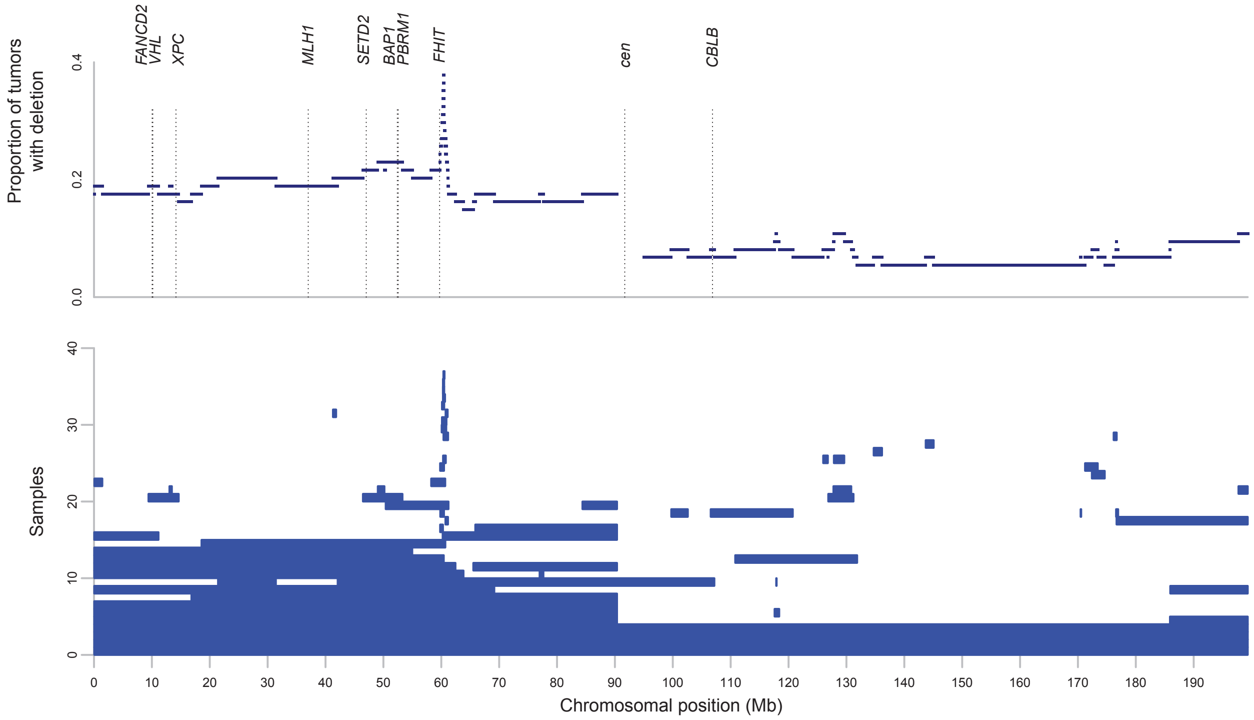

FANCD2
VHL
XPC
MLH1
SETD2
BAP1
PBRM1
FHIT
cen
CBLB
Proportion of tumors with deletion
0.0
0.2
0.4
Samples
0
10
20
30
40
0 10 20 30 40 50 60 70 80 90 100 110 120 130 140 150 160 170 180 190
Chromosomal position (Mb)

Chr 4

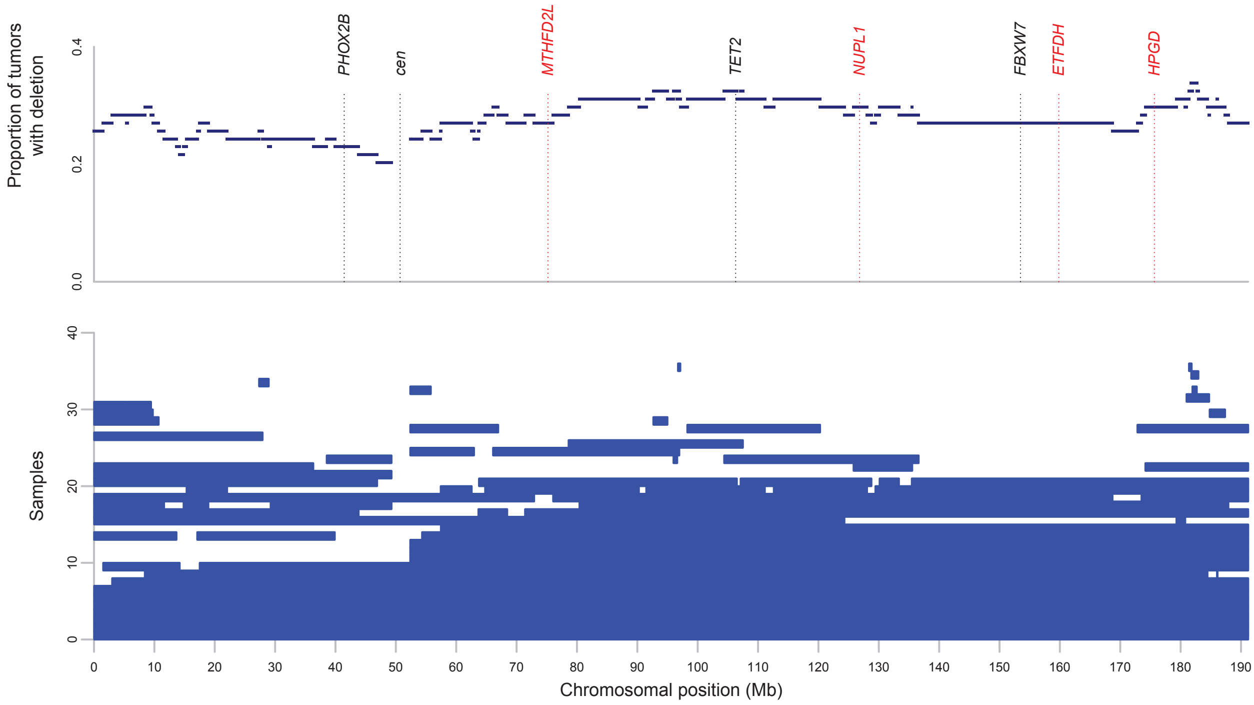

PHOX2B
cen
MTHFD2L
TET2
NUPL1
FBXW7
ETFDH
HPGD
Proportion of tumors with deletion
0.0
0.2
0.4
Samples
0
10
20
30
40
0 10 20 30 40 50 60 70 80 90 100 110 120 130 140 150 160 170 180 190
Chromosomal position (Mb)

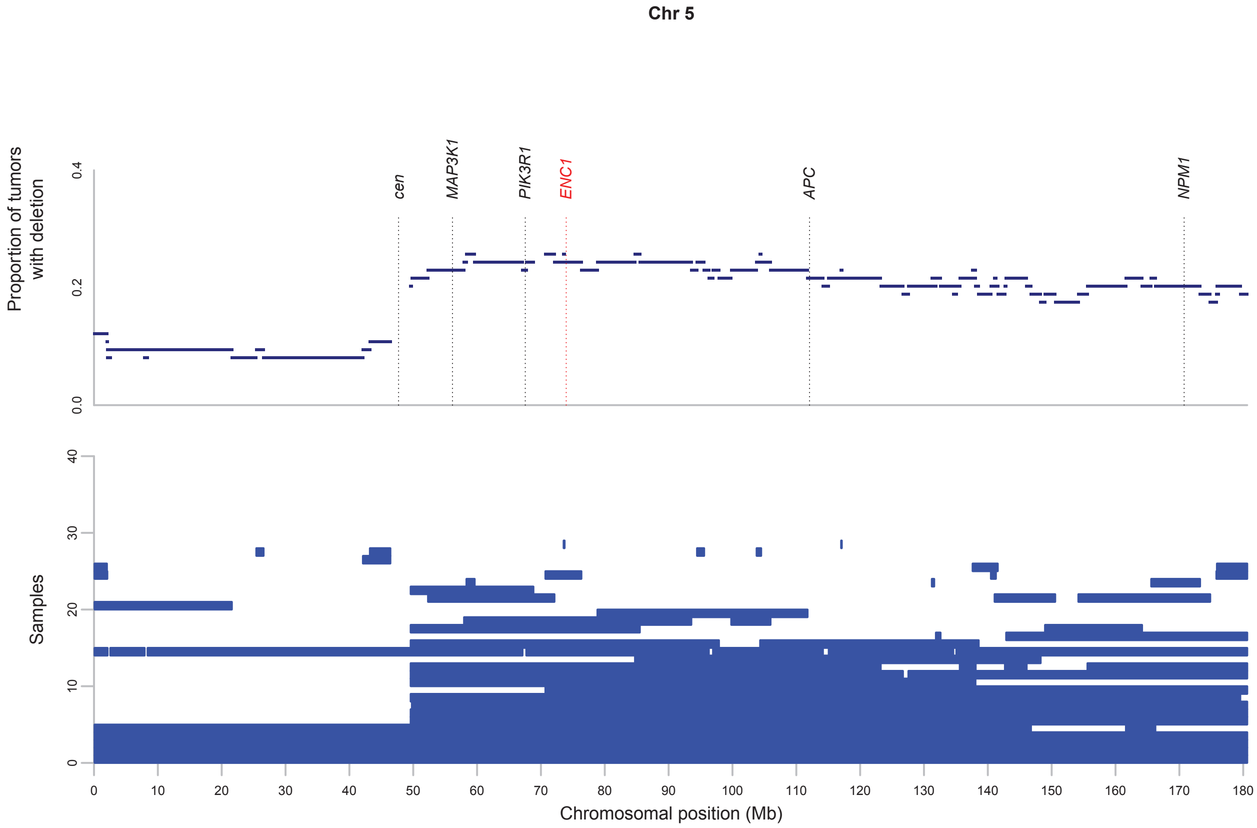
Chr 5
Proportion of tumors with deletion
0.0
0.2
0.4
cen
MAP3K1
PIK3R1
ENC1
APC
NPM1
Samples
0
10
20
30
40
0
10
20
30
40
50
60
70
80
90
100
110
120
130
140
150
160
170
180
Chromosomal position (Mb)

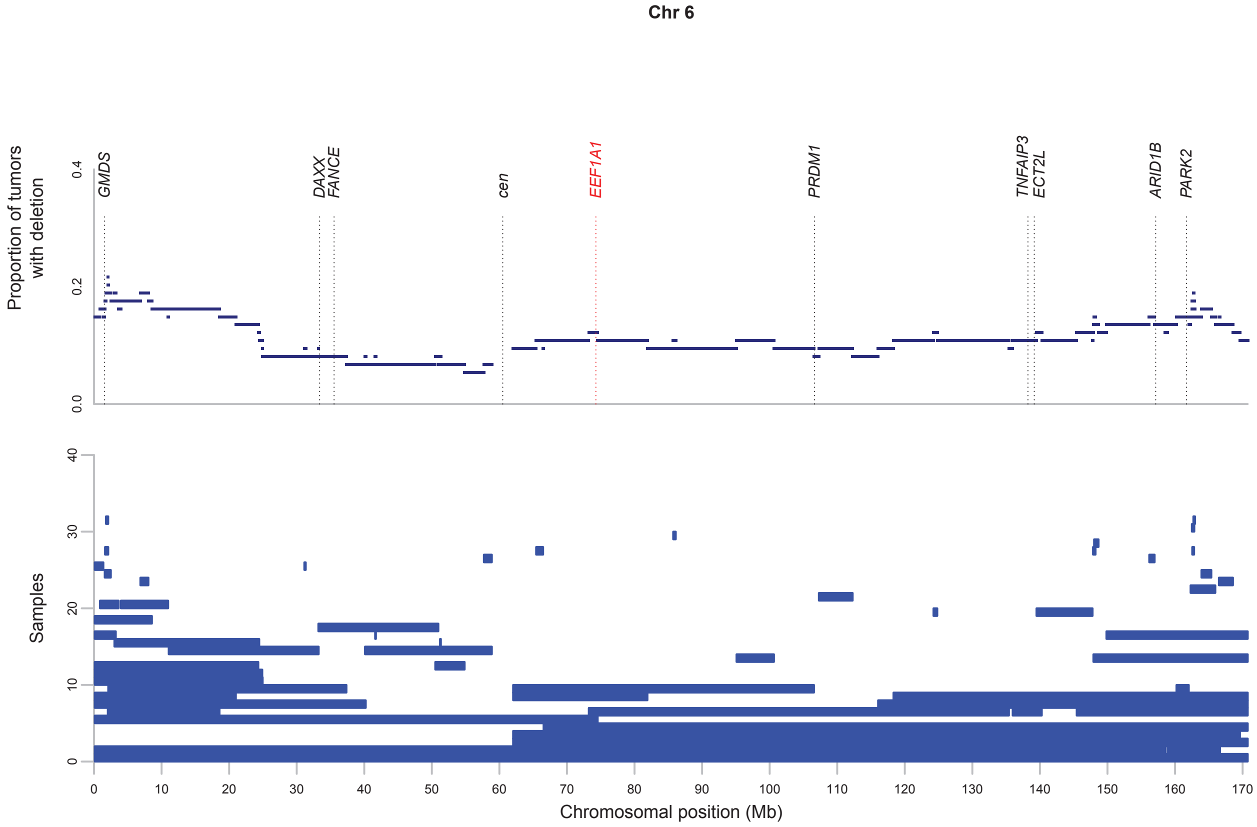
Chr 6
Proportion of tumors with deletion
0.0
0.2
0.4
GMDS
DAXX
FANCE
cen
EEF1A1
PRDM1
TNFAIP3
ECT2L
ARID1B
PARK2
Samples
0
10
20
30
40
0
10
20
30
40
50
60
70
80
90
100
110
120
130
140
150
160
170
Chromosomal position (Mb)

Chr 7

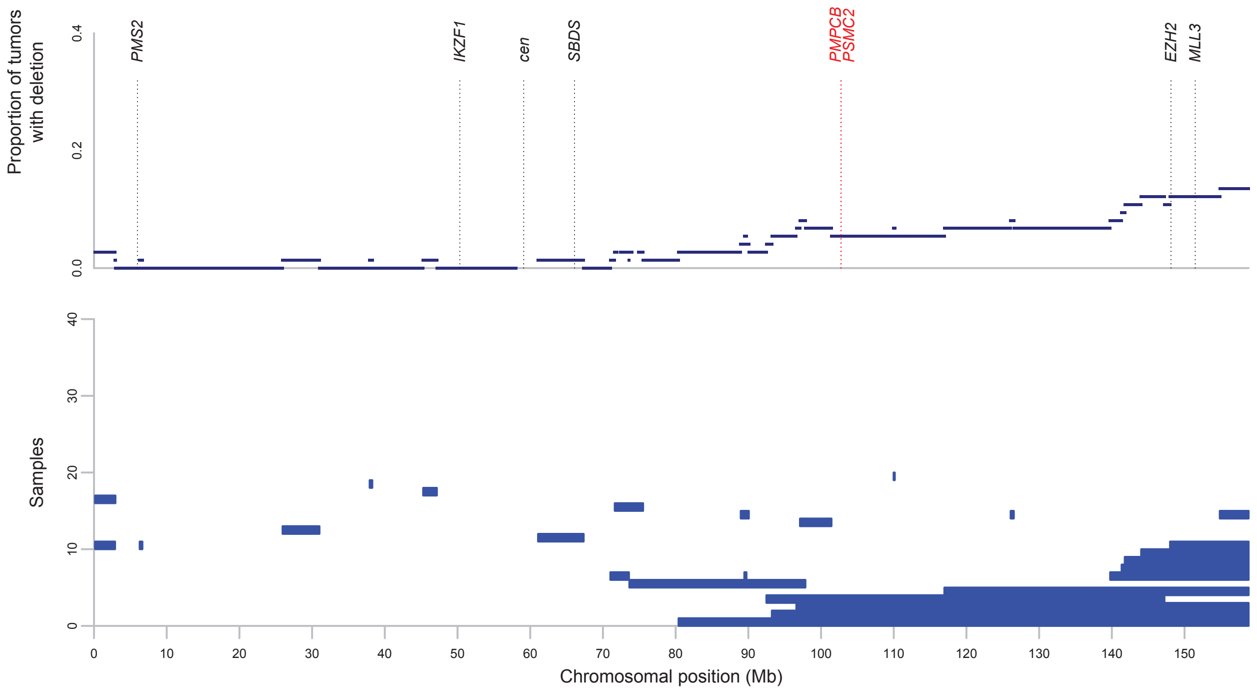
Proportion of tumors with deletion
0.0
0.2
0.4
PMS2
IKZF1
cen
SBDS
PMPCB
PSMC2
EZH2
MLL3
Samples
0
10
20
30
40
0
10
20
30
40
50
60
70
80
90
100
110
120
130
140
150
Chromosomal position (Mb)

Chr 8

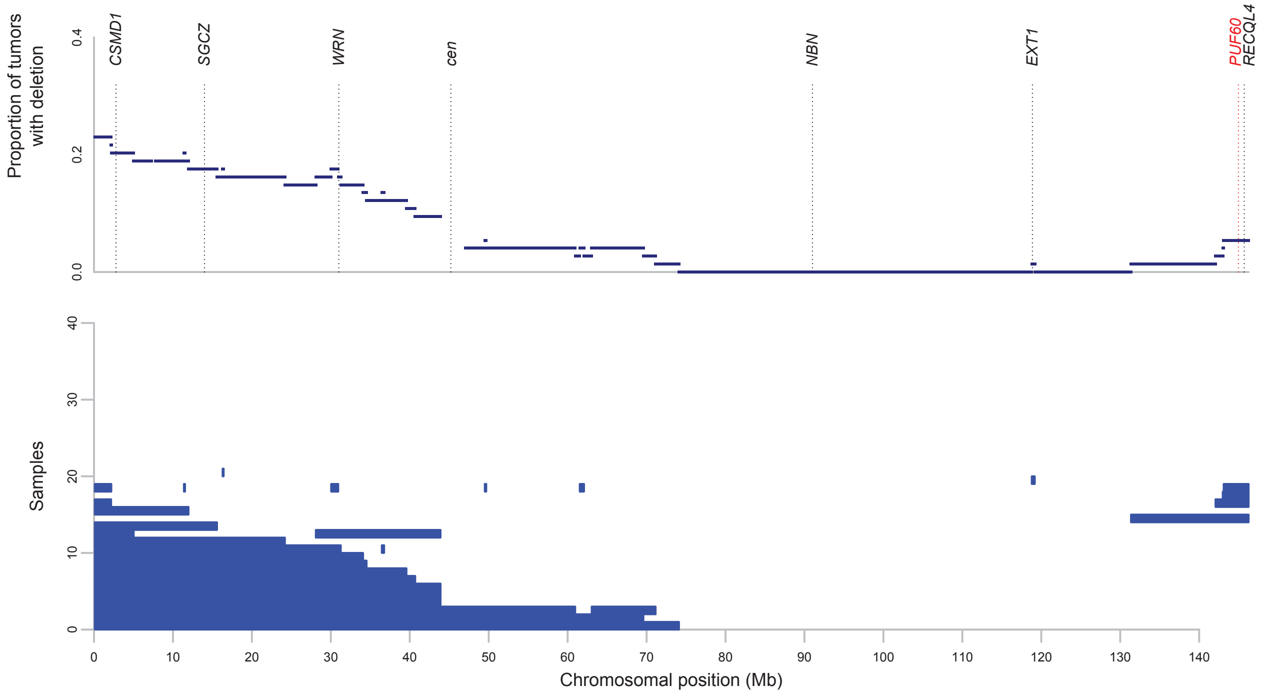
Proportion of tumors with deletion
0.0
0.2
0.4
CSMD1
SGCZ
WRN
cen
NBN
EXT1
PUF60
RECQL4
Samples
0
10
20
30
40
0
10
20
30
40
50
60
70
80
90
100
110
120
130
140
Chromosomal position (Mb)

Chr 9

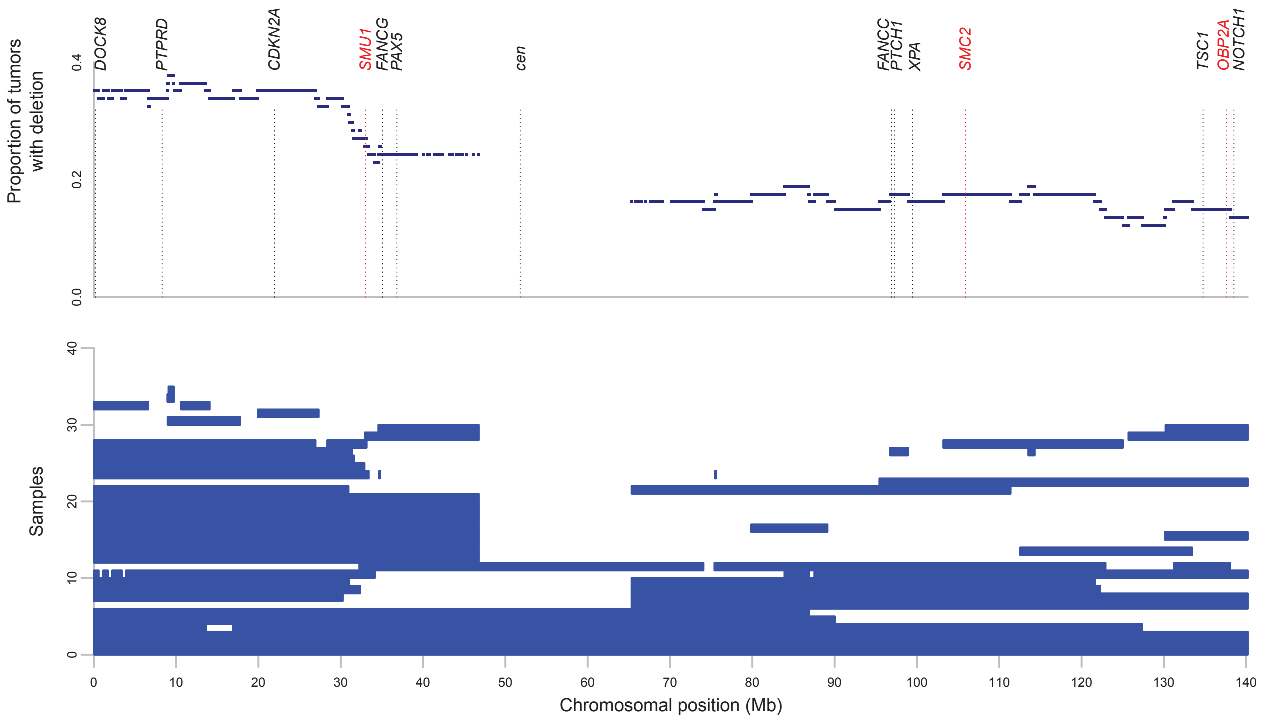
DOCK8
PTPRD
CDKN2A
SMU1
FANCG
PAX5
cen
FANCC
PTCH1
XPA
SMC2
TSC1
OBP2A
NOTCH1
Proportion of tumors with deletion
0.0
0.2
0.4
Samples
0
10
20
30
40
0
10
20
30
40
50
60
70
80
90
100
110
120
130
140
Chromosomal position (Mb)

Chr 10

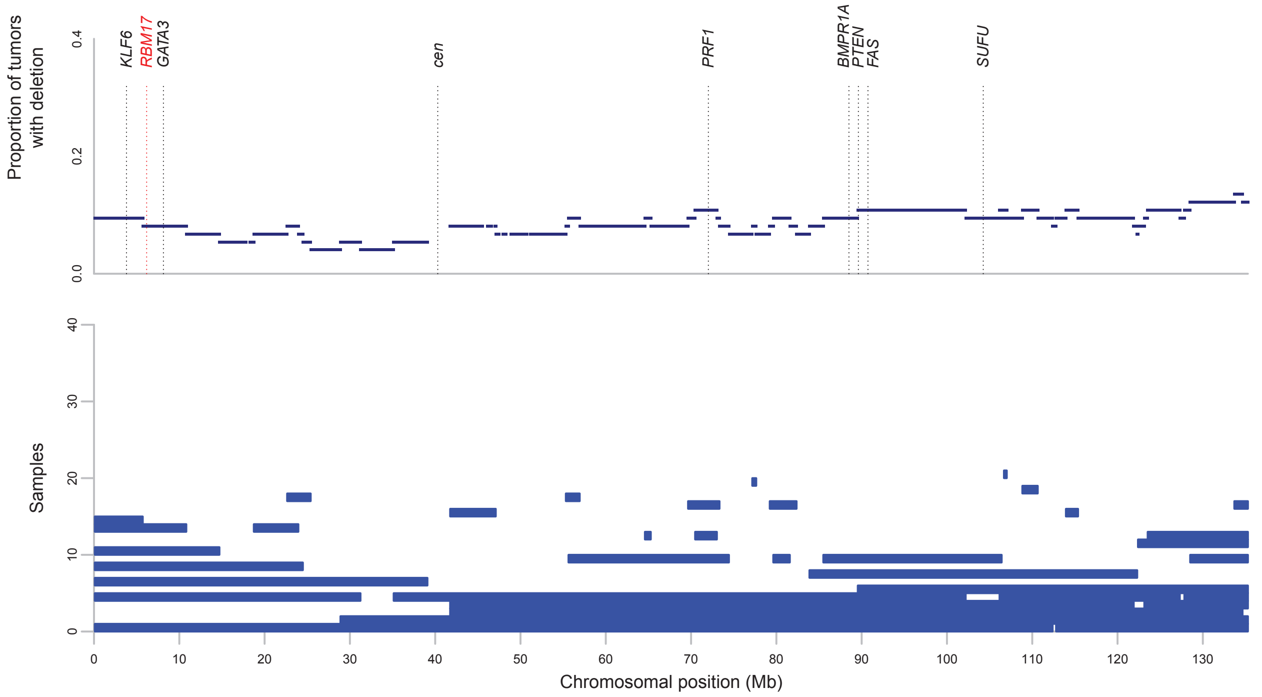
KLF6
RBM17
GATA3
cen
PRF1
BMPR1A
PTEN
FAS
SUFU
Proportion of tumors with deletion
0.0
0.2
0.4
Samples
0
10
20
30
40
0
10
20
30
40
50
60
70
80
90
100
110
120
130
Chromosomal position (Mb)

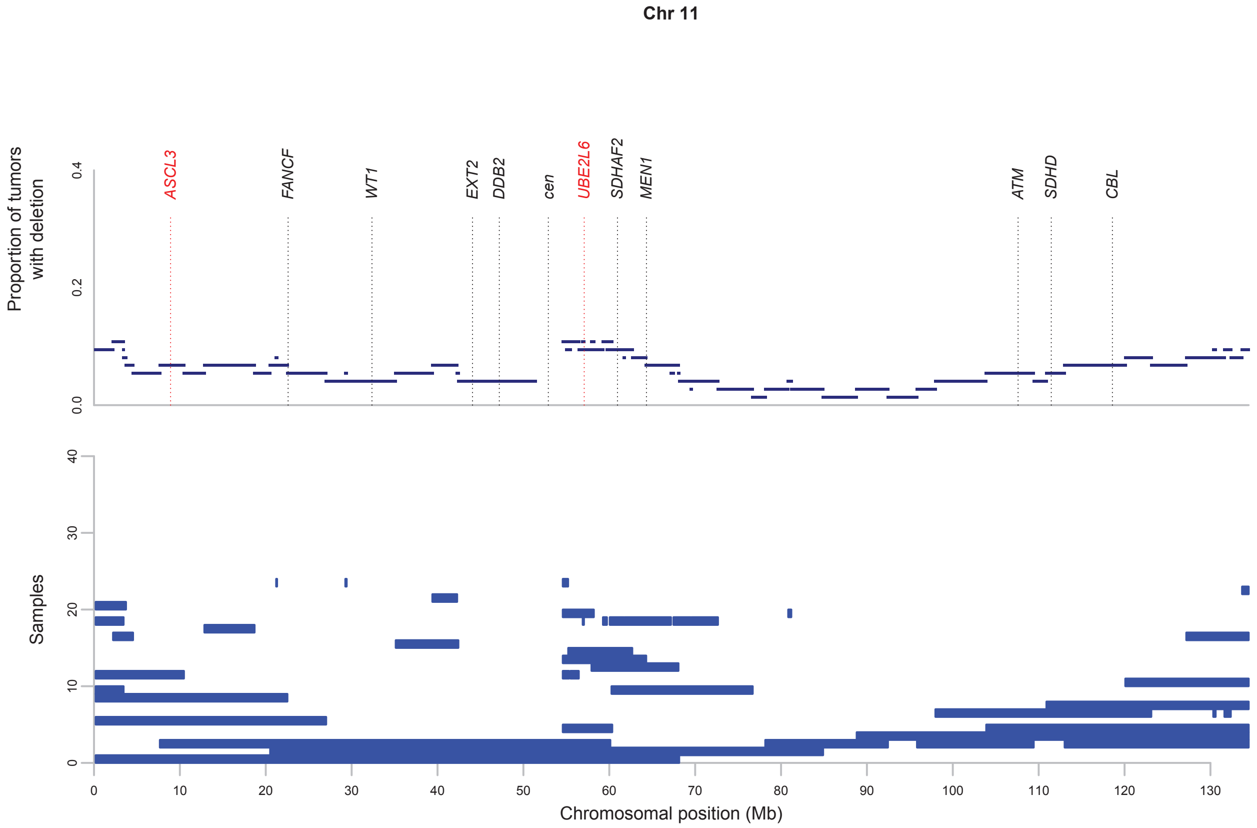
Chr 11
Proportion of tumors with deletion
ASCL3
FANCF
WT1
EXT2
DDB2
cen
UBE2L6
SDHAF2
MEN1
ATM
SDHD
CBL
Samples
Chromosomal position (Mb)

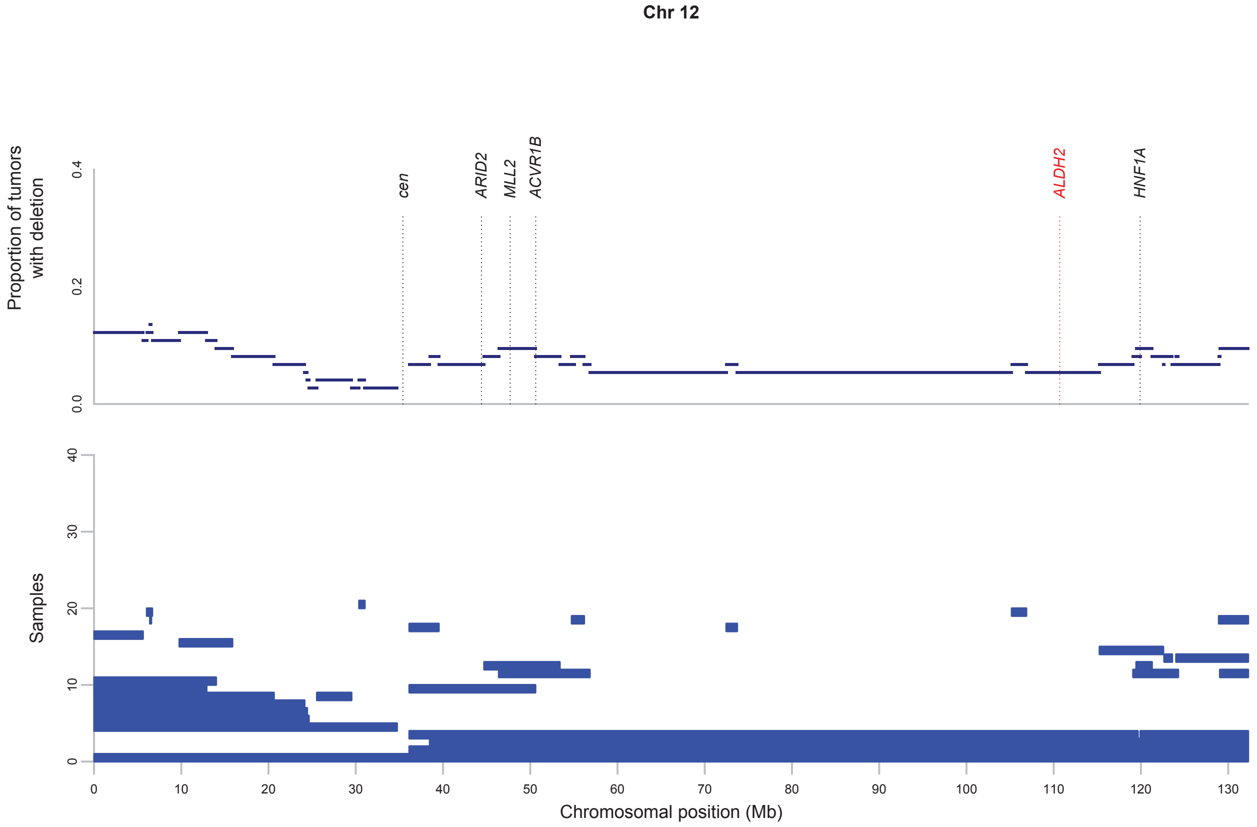
Chr 12
Proportion of tumors with deletion
cen
ARID2
MLL2
ACVR1B
ALDH2
HNF1A
Samples
Chromosomal position (Mb)

Chr 13

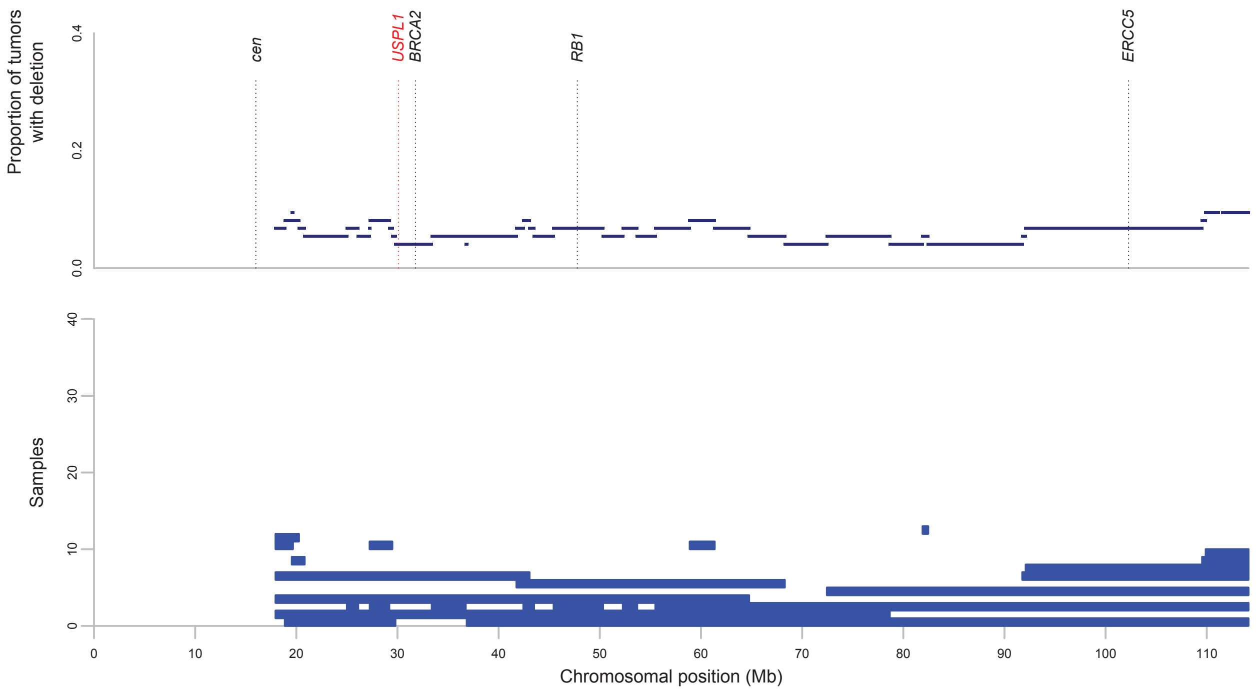

Proportion of tumors with deletion
0.0
0.2
0.4
cen
USPL1
BRCA2
RB1
ERCC5
Samples
0
10
20
30
40
0
10
20
30
40
50
60
70
80
90
100
110
Chromosomal position (Mb)

Chr 14

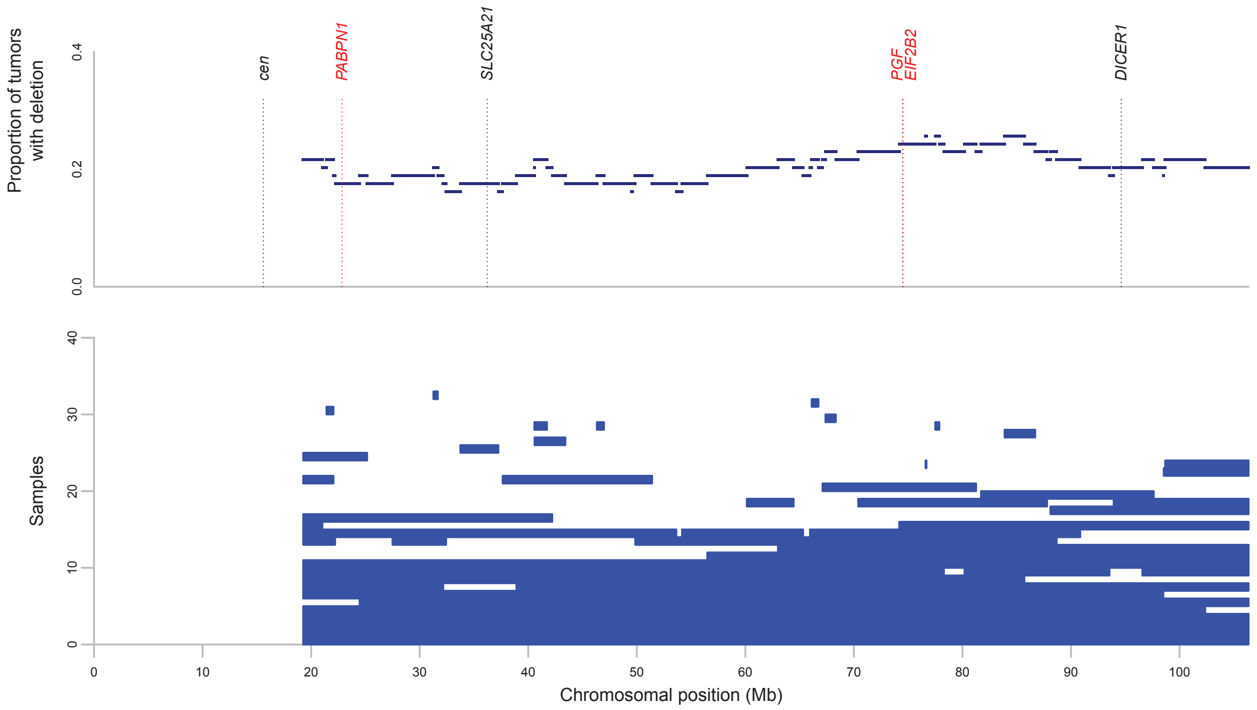

Proportion of tumors with deletion
0.0
0.2
0.4
cen
PABPN1
SLC25A21
PGF
EIF2B2
DICER1
Samples
0
10
20
30
40
0
10
20
30
40
50
60
70
80
90
100
Chromosomal position (Mb)

Chr 15

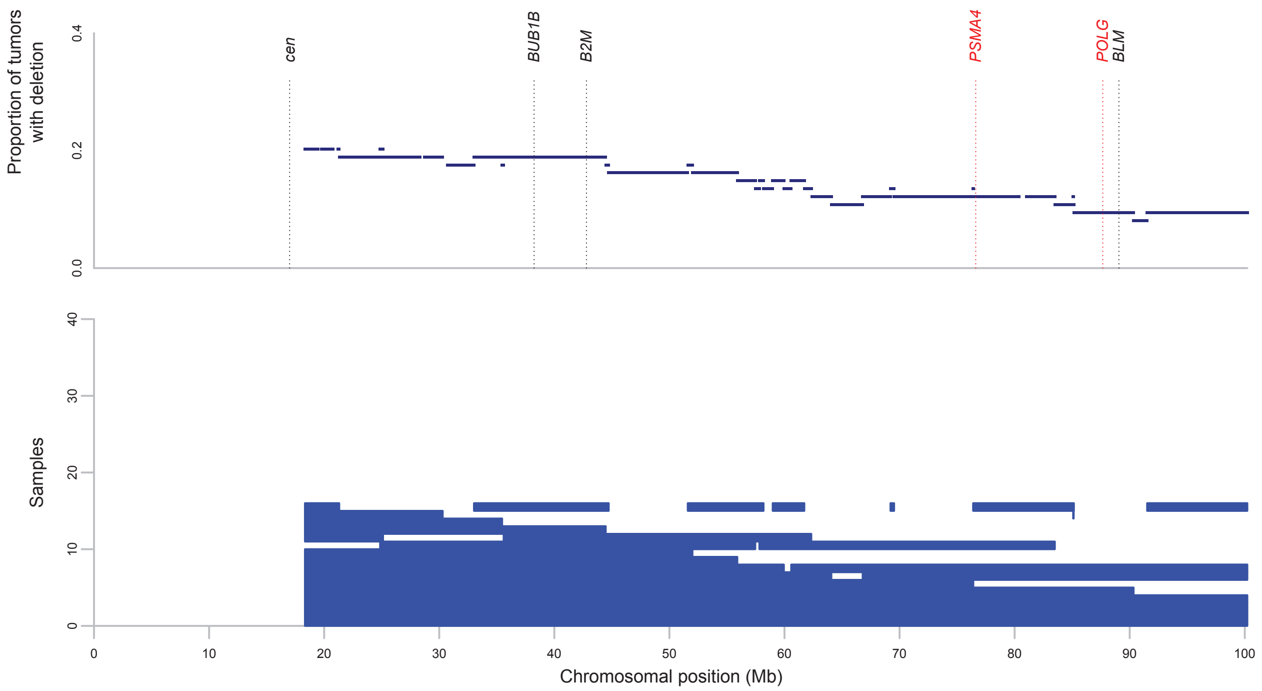
Proportion of tumors with deletion
Samples
Chromosomal position (Mb)
cen
BUB1B
B2M
PSMA4
POLG
BLM
0.0
0.2
0.4
0
10
20
30
40
50
60
70
80
90
100

Chr 16

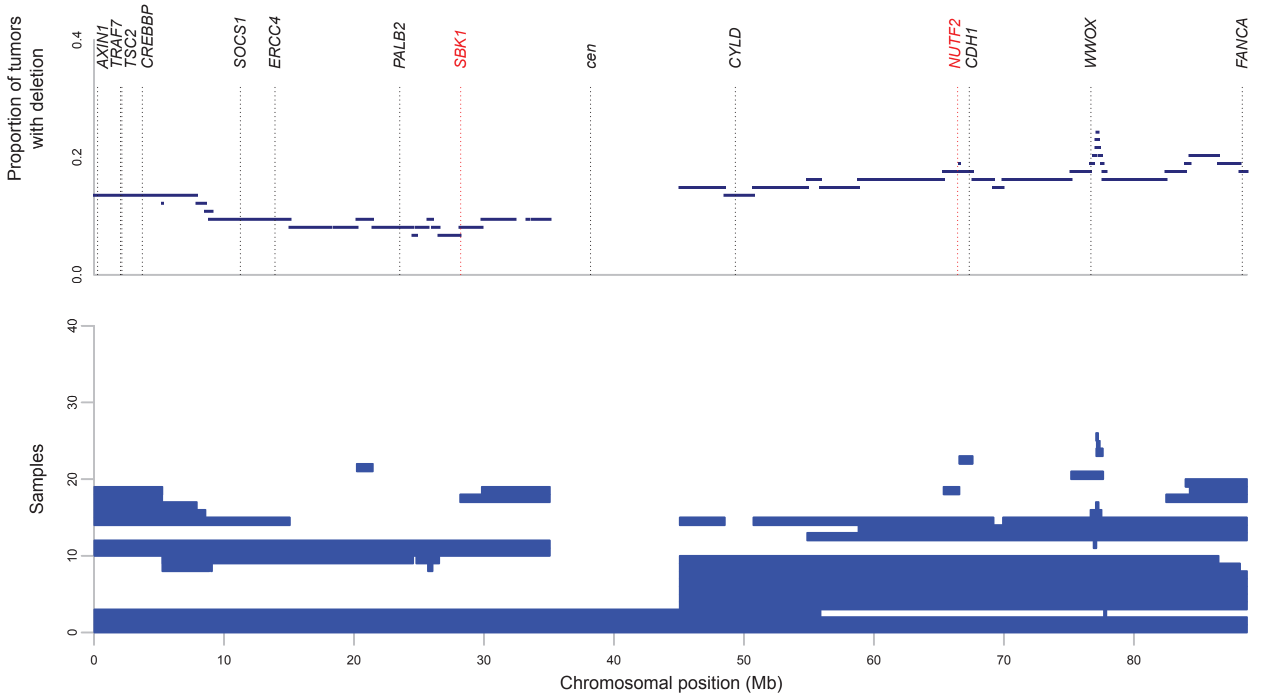
Proportion of tumors with deletion
Samples
Chromosomal position (Mb)
AXIN1
TRAF7
TSC2
CREBBP
SOCS1
ERCC4
PALB2
SBK1
cen
CYLD
NUTF2
CDH1
WWOX
FANCA
0.0
0.2
0.4
0
10
20
30
40
50
60
70
80

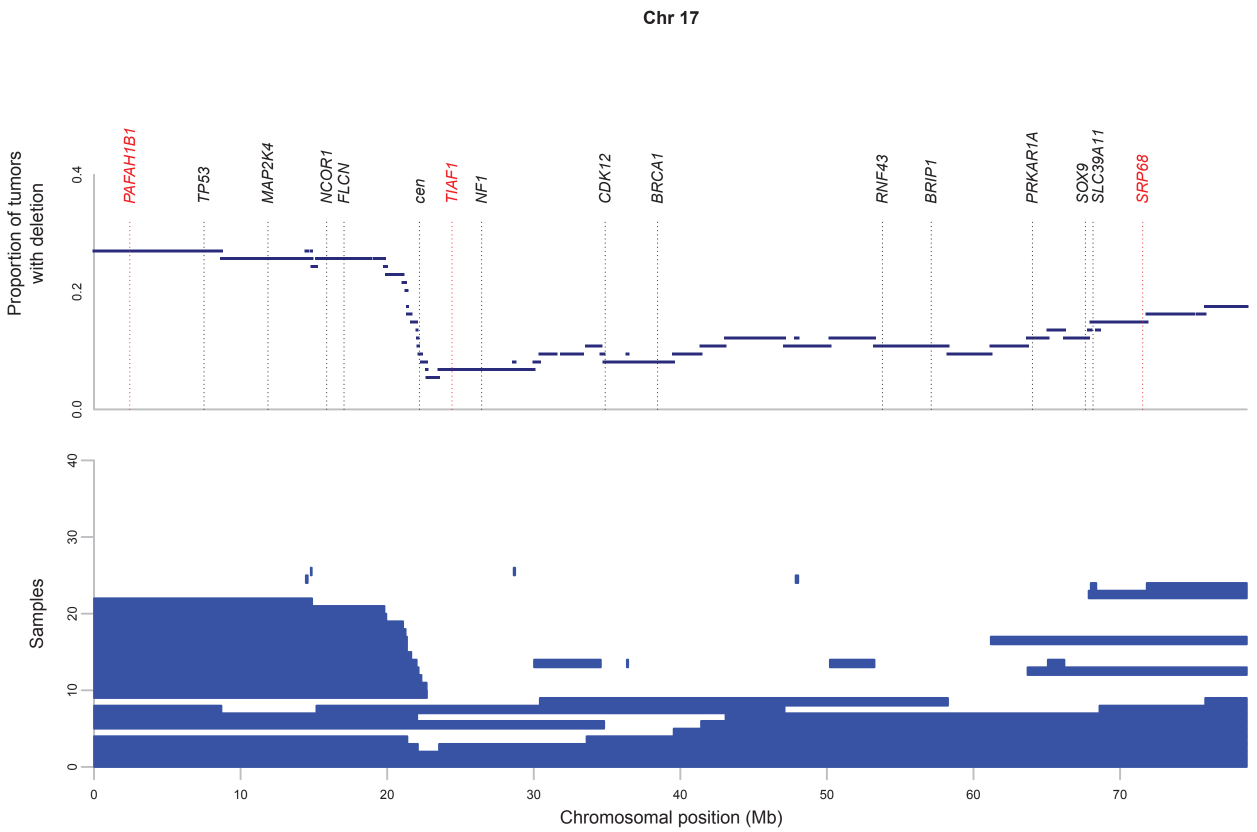
Chr 17
PAFAH1B1
TP53
MAP2K4
NCOR1
FLCN
cen
TIAF1
NF1
CDK12
BRCA1
RNF43
BRIP1
PRKAR1A
SOX9
SLC39A11
SRP68
Proportion of tumors with deletion
0.0
0.2
0.4
Samples
0
10
20
30
40
0
10
20
30
40
50
60
70
Chromosomal position (Mb)

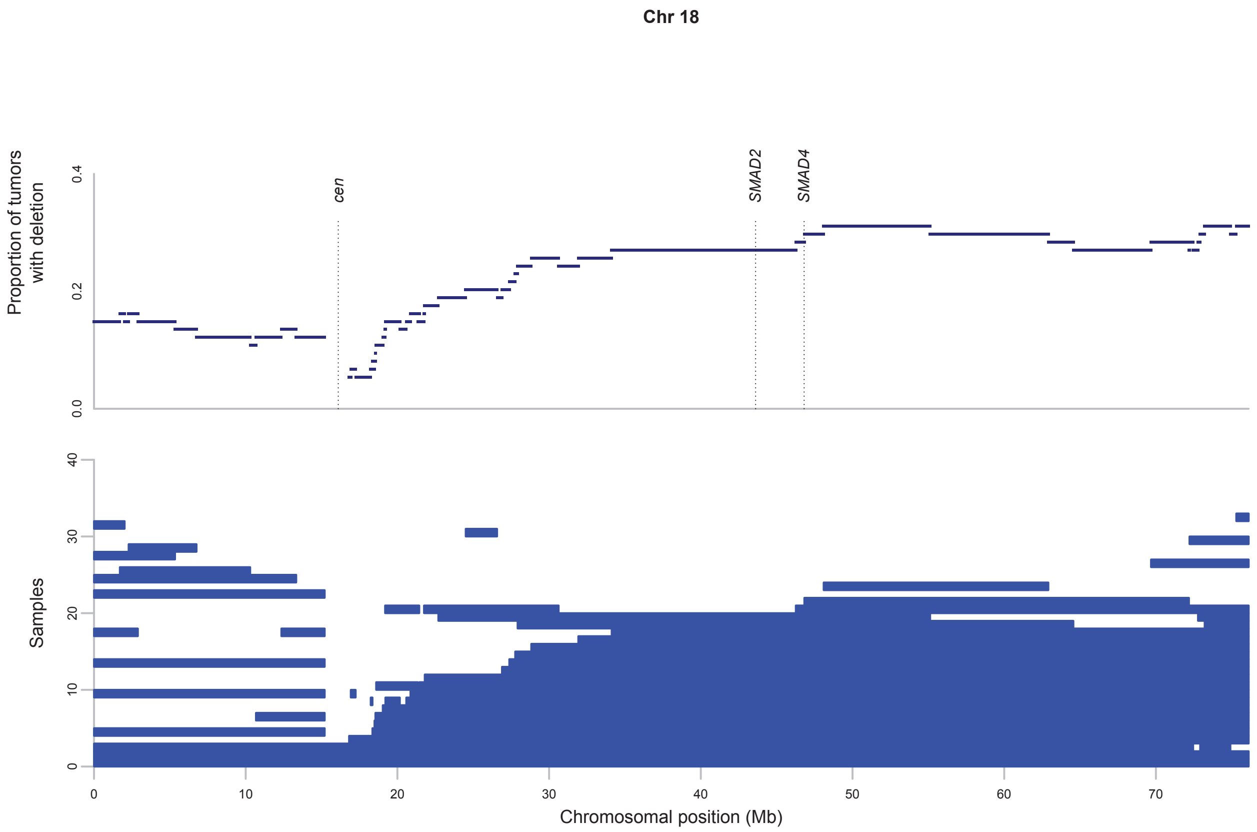
Chr 18
cen
SMAD2
SMAD4
Proportion of tumors with deletion
0.0
0.2
0.4
Samples
0
10
20
30
40
0
10
20
30
40
50
60
70
Chromosomal position (Mb)

Chr 19

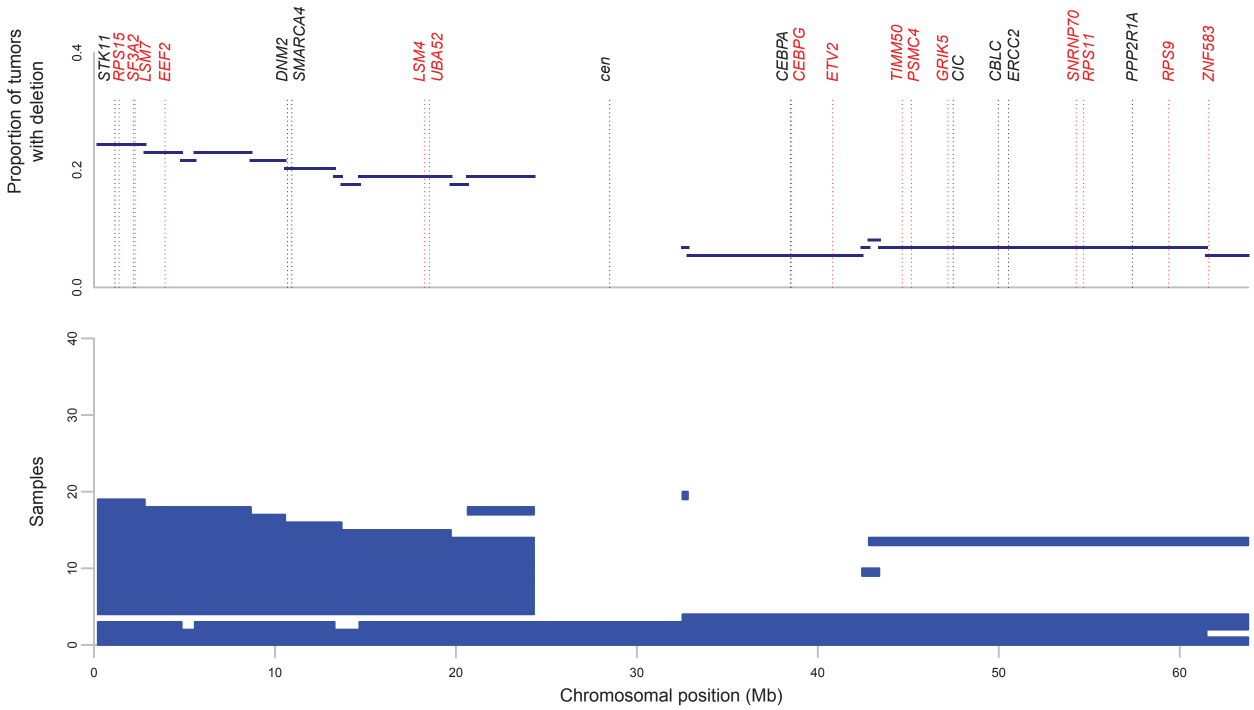
Proportion of tumors with deletion
0.0
0.2
0.4
STK11
RPS15
SF3A2
LSM7
EEF2
DNM2
SMARCA4
LSM4
UBA52
cen
CEBPA
CEBPG
ETV2
TIMM50
PSMC4
GRIK5
CIC
CBLC
ERCC2
SNRNP70
RPS11
PPP2R1A
RPS9
ZNF583
Samples
0
10
20
30
40
0
10
20
30
40
50
60
Chromosomal position (Mb)

Chr 20

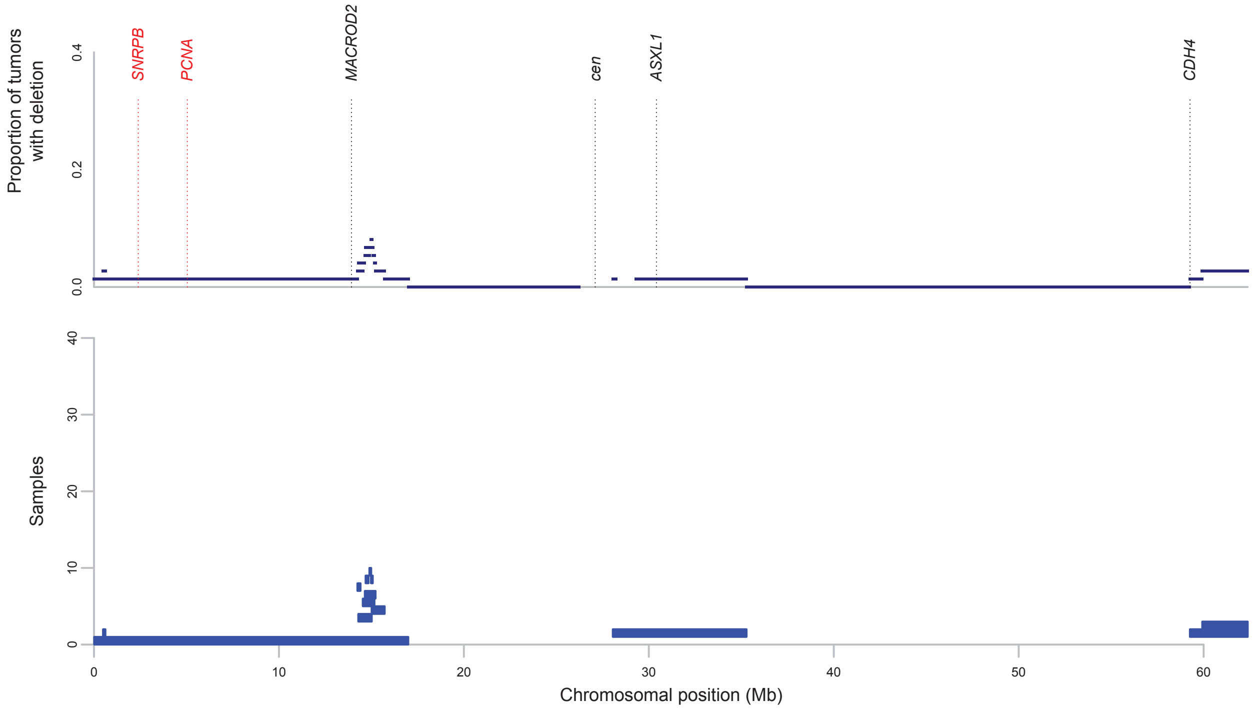
Proportion of tumors with deletion
0.0
0.2
0.4
SNRPB
PCNA
MACROD2
cen
ASXL1
CDH4
Samples
0
10
20
30
40
0
10
20
30
40
50
60
Chromosomal position (Mb)

Chr 21

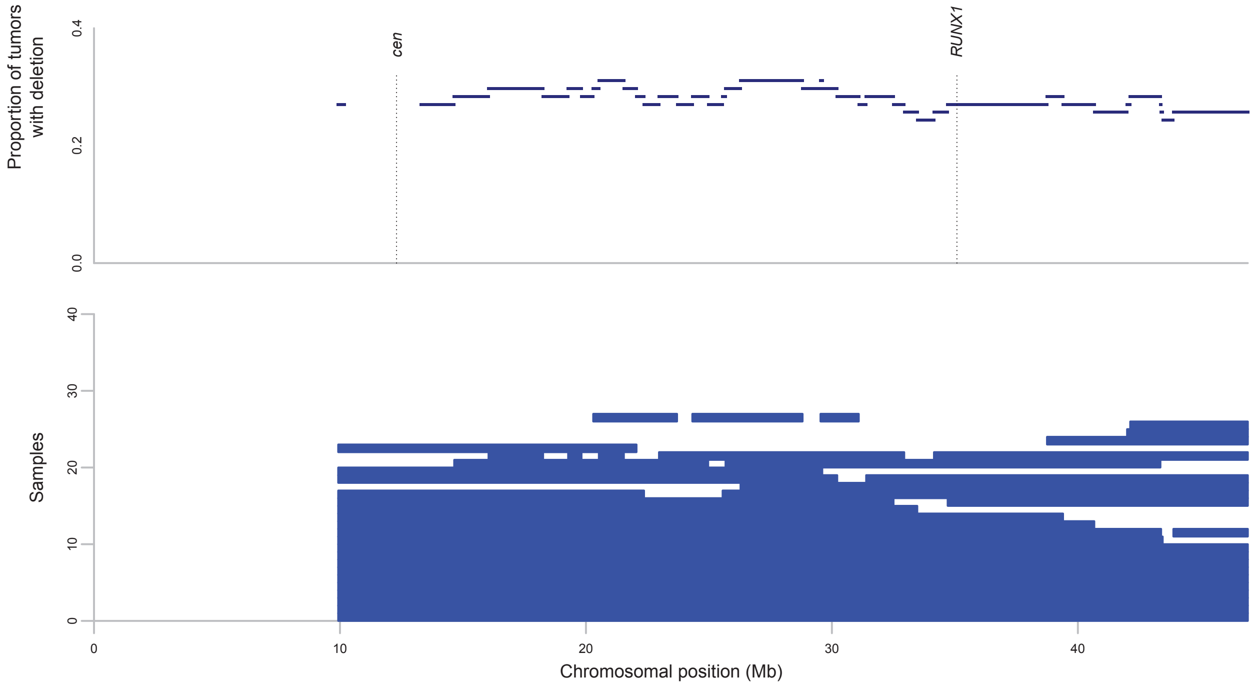
Proportion of tumors with deletion
0.0
0.2
0.4
cen
RUNX1
Samples
0
10
20
30
40
0
10
20
30
40
Chromosomal position (Mb)

Chr 22

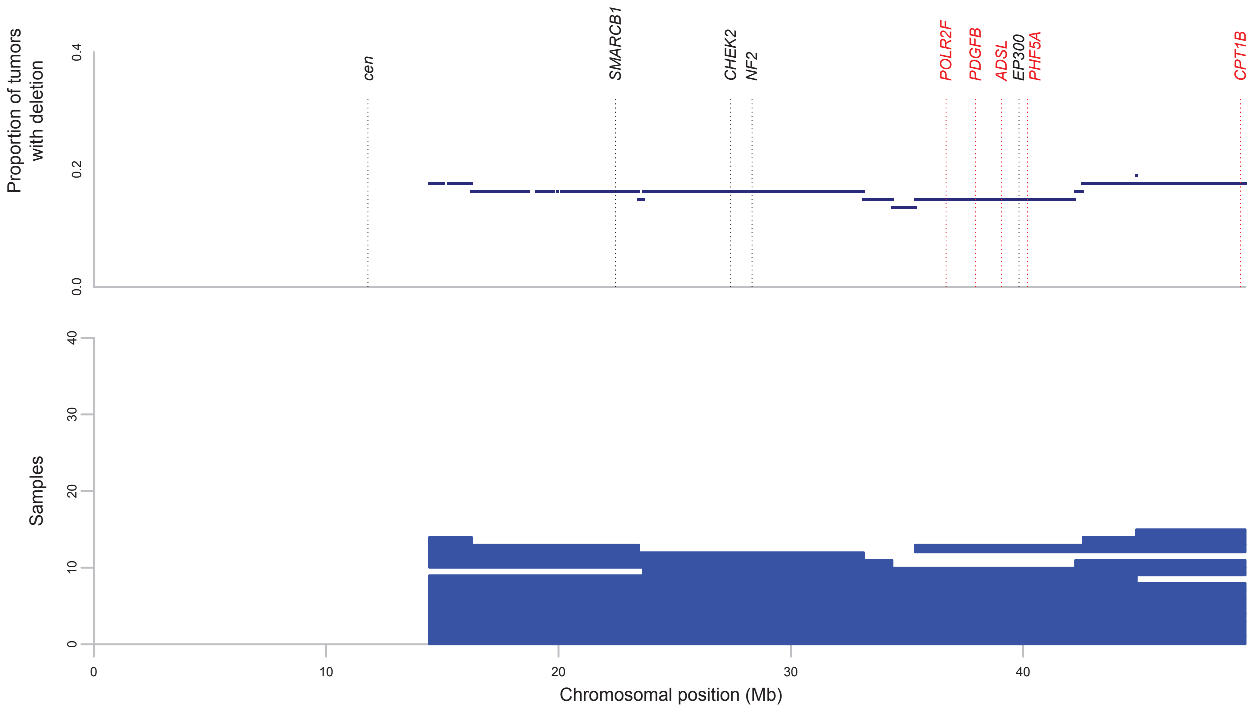
Proportion of tumors with deletion
0.0
0.2
0.4
cen
SMARCB1
CHEK2
NF2
POLR2F
PDGFB
ADSL
EP300
PHF5A
CPT1B
Samples
0
10
20
30
40
0
10
20
30
40
Chromosomal position (Mb)

Fig. S8. Landscape of LOH by chromosome.
For each chromosome, the proportion of tumors showing LOH at each SNP, based on ASCAT's allele-specific copy number analysis (upper panels) and regions of LOH in specific tumors (lower panels). The genes marked in black color are tumor suppressor genes.

Chr 1

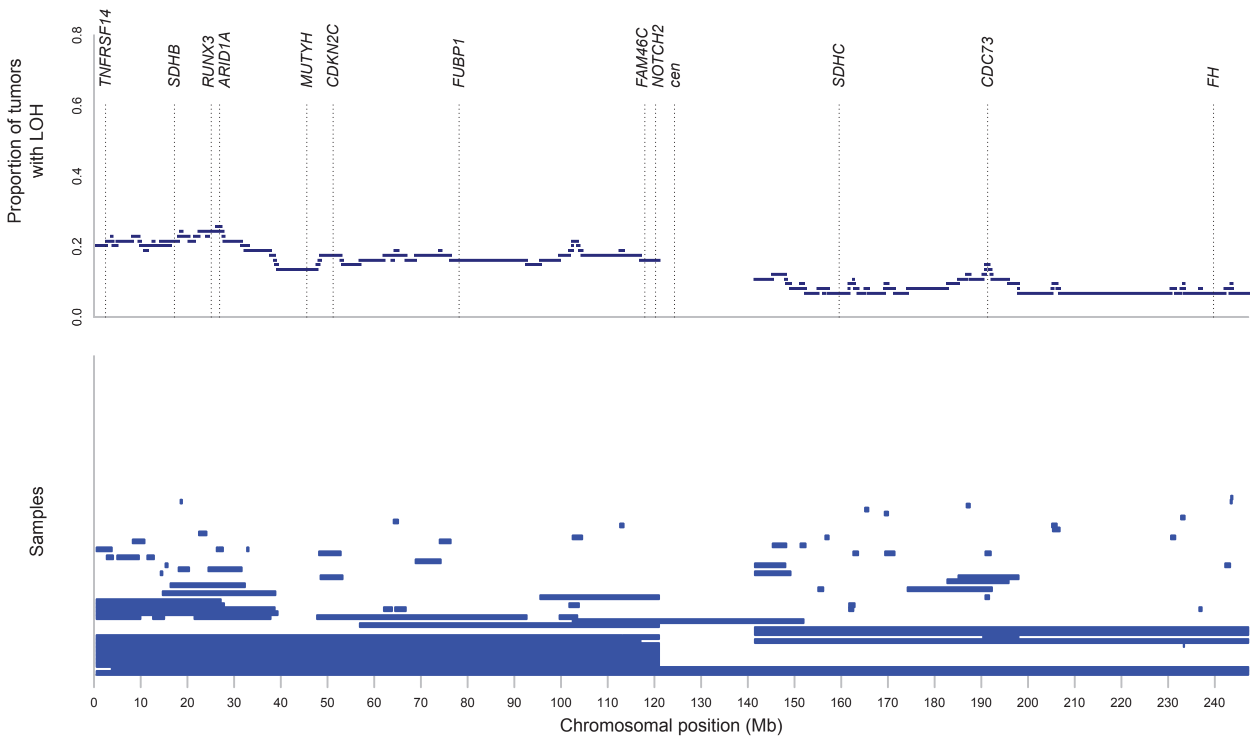
Proportion of tumors with LOH
0.0
0.2
0.4
0.6
0.8
TNFRSF14
SDHB
RUNX3
ARID1A
MUTYH
CDKN2C
FUBP1
FAM46C
NOTCH2
cen
SDHC
CDC73
FH
Samples
0 10 20 30 40 50 60 70 80 90 100 110 120 130 140 150 160 170 180 190 200 210 220 230 240
Chromosomal position (Mb)

Chr 2

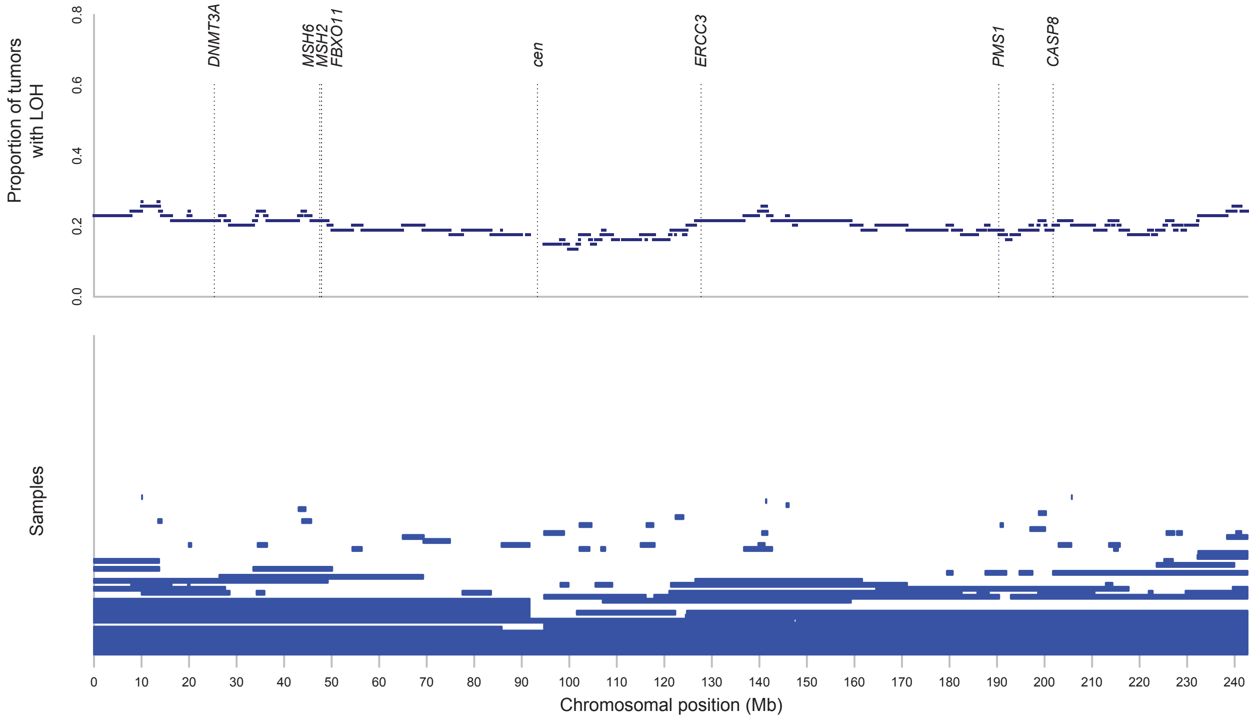
Proportion of tumors with LOH
0.0
0.2
0.4
0.6
0.8
DNMT3A
MSH6
MSH2
FBXO11
cen
ERCC3
PMS1
CASP8
Samples
0 10 20 30 40 50 60 70 80 90 100 110 120 130 140 150 160 170 180 190 200 210 220 230 240
Chromosomal position (Mb)

Chr 3

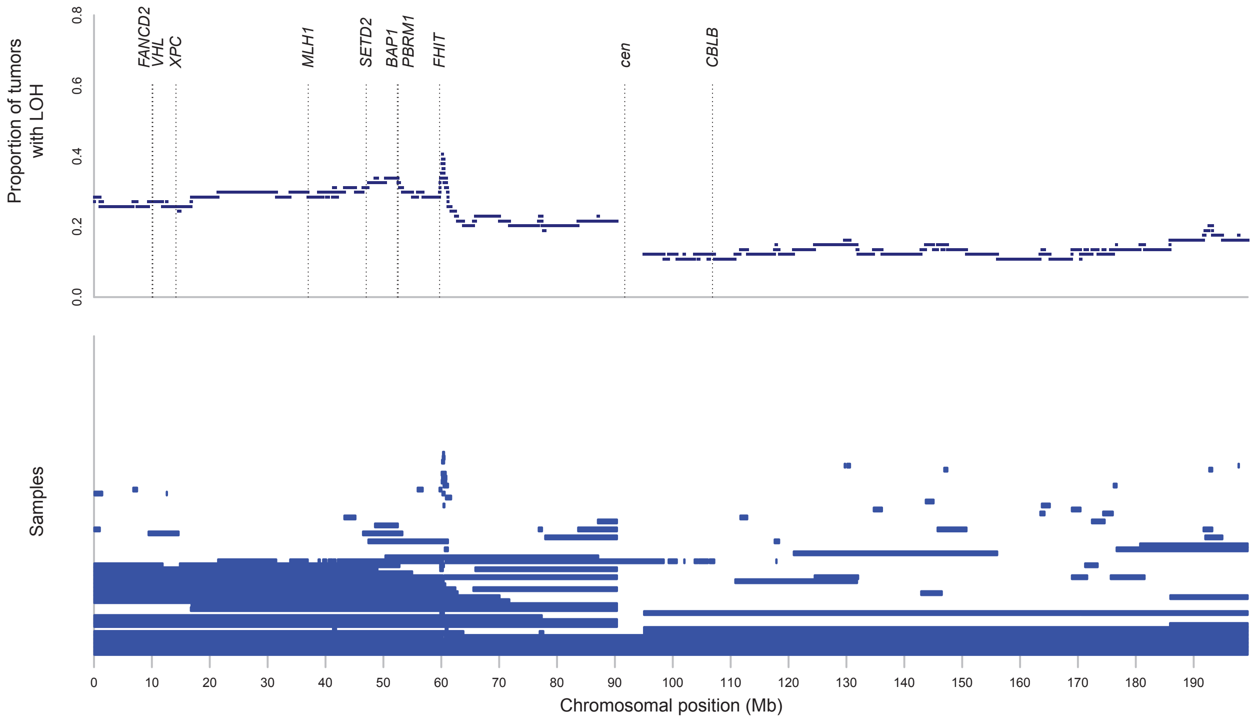

Proportion of tumors with LOH
0.0
0.2
0.4
0.6
0.8
FANCD2
VHL
XPC
MLH1
SETD2
BAP1
PBRM1
FHIT
cen
CBLB
Samples
0
10
20
30
40
50
60
70
80
90
100
110
120
130
140
150
160
170
180
190
Chromosomal position (Mb)

Chr 4

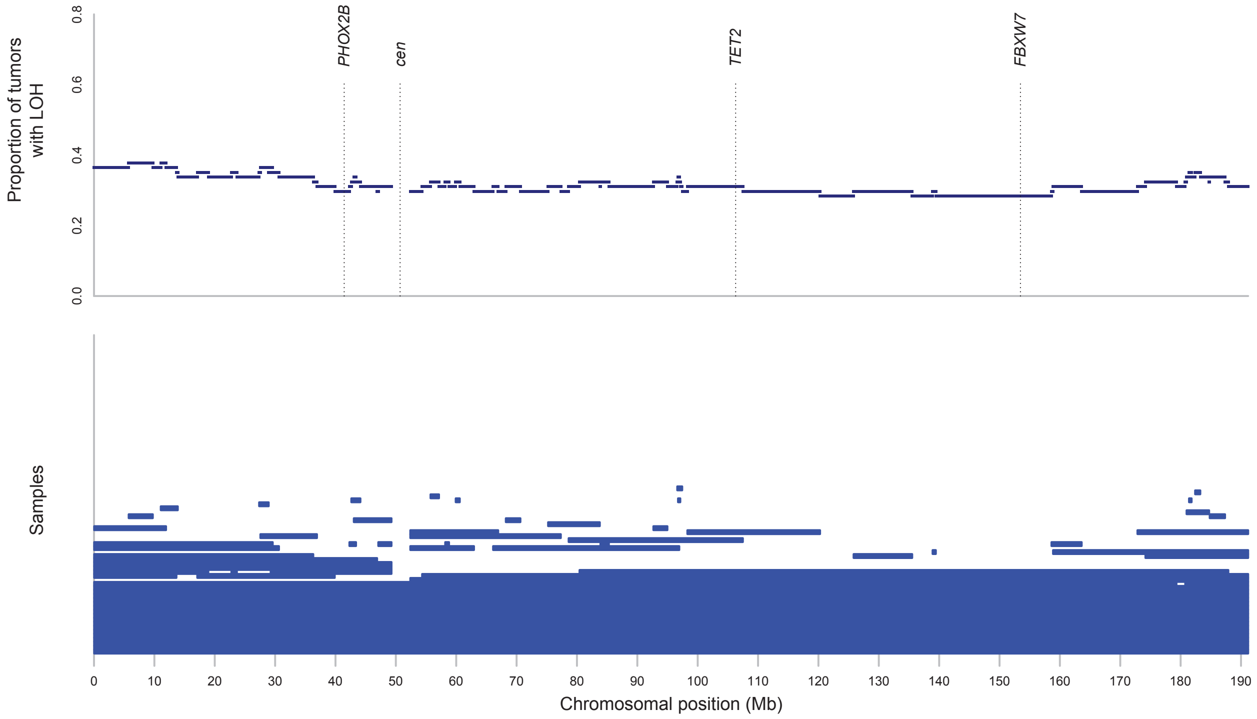

Proportion of tumors with LOH
0.0
0.2
0.4
0.6
0.8
PHOX2B
cen
TET2
FBXW7
Samples
0
10
20
30
40
50
60
70
80
90
100
110
120
130
140
150
160
170
180
190
Chromosomal position (Mb)

Chr 5

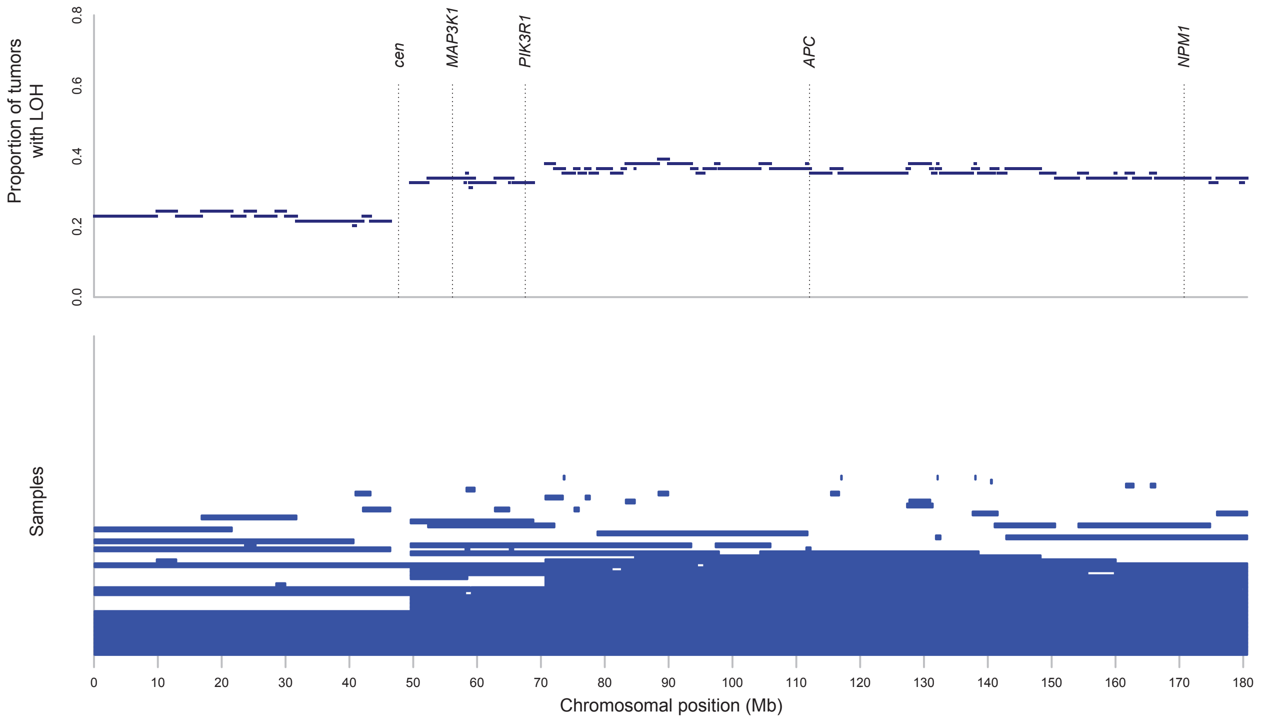

Proportion of tumors with LOH
0.0
0.2
0.4
0.6
0.8
cen
MAP3K1
PIK3R1
APC
NPM1
Samples
0
10
20
30
40
50
60
70
80
90
100
110
120
130
140
150
160
170
180
Chromosomal position (Mb)

Chr 6

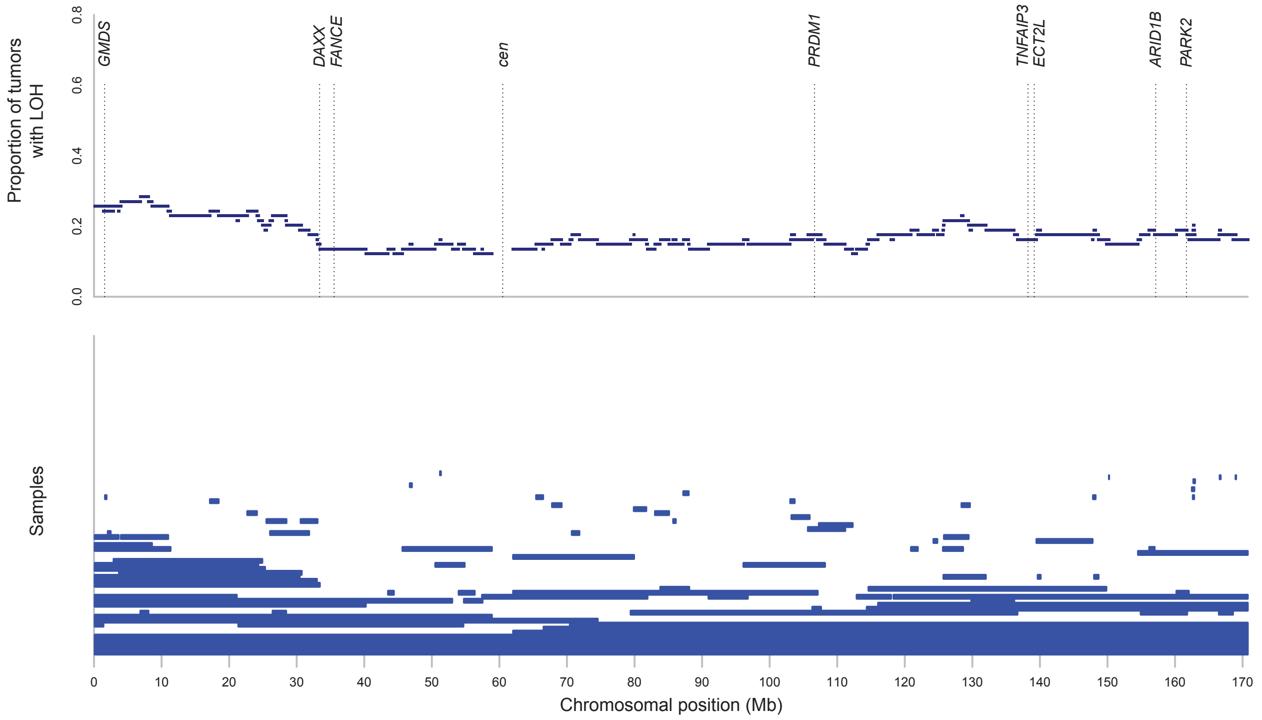

Proportion of tumors with LOH
0.0
0.2
0.4
0.6
0.8
GMDS
DAXX
FANCE
cen
PRDM1
TNFAIP3
ECT2L
ARID1B
PARK2
Samples
0
10
20
30
40
50
60
70
80
90
100
110
120
130
140
150
160
170
Chromosomal position (Mb)

Chr 7

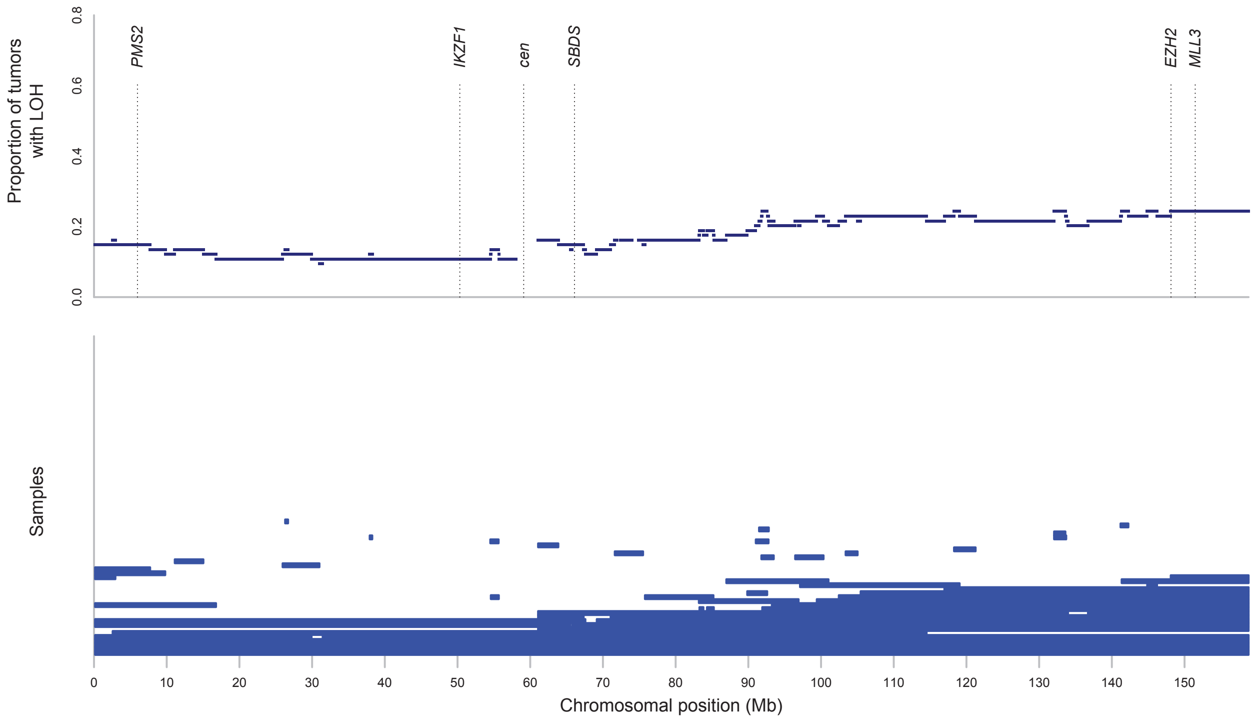
Proportion of tumors with LOH
0.0
0.2
0.4
0.6
0.8
PMS2
IKZF1
cen
SBDS
EZH2
MLL3
Samples
0
10
20
30
40
50
60
70
80
90
100
110
120
130
140
150
Chromosomal position (Mb)

Chr 8

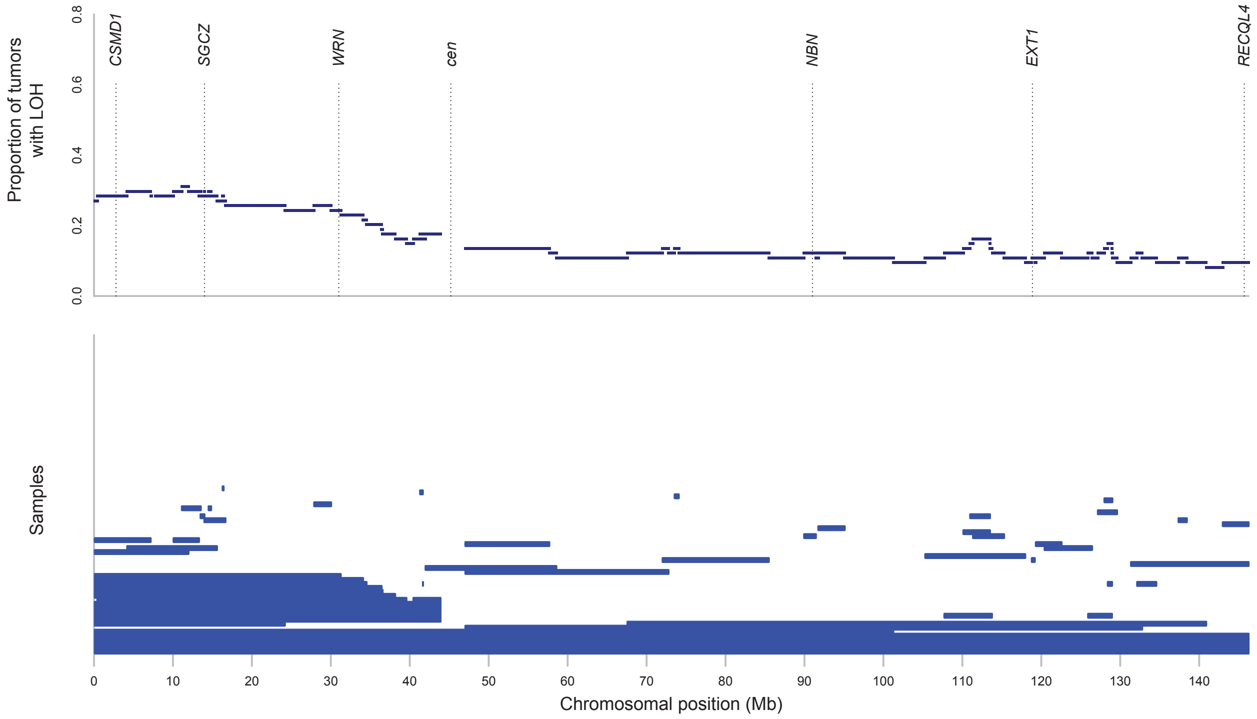
Proportion of tumors with LOH
0.0
0.2
0.4
0.6
0.8
CSMD1
SGCZ
WRN
cen
NBN
EXT1
RECQL4
Samples
0
10
20
30
40
50
60
70
80
90
100
110
120
130
140
Chromosomal position (Mb)

Chr 9

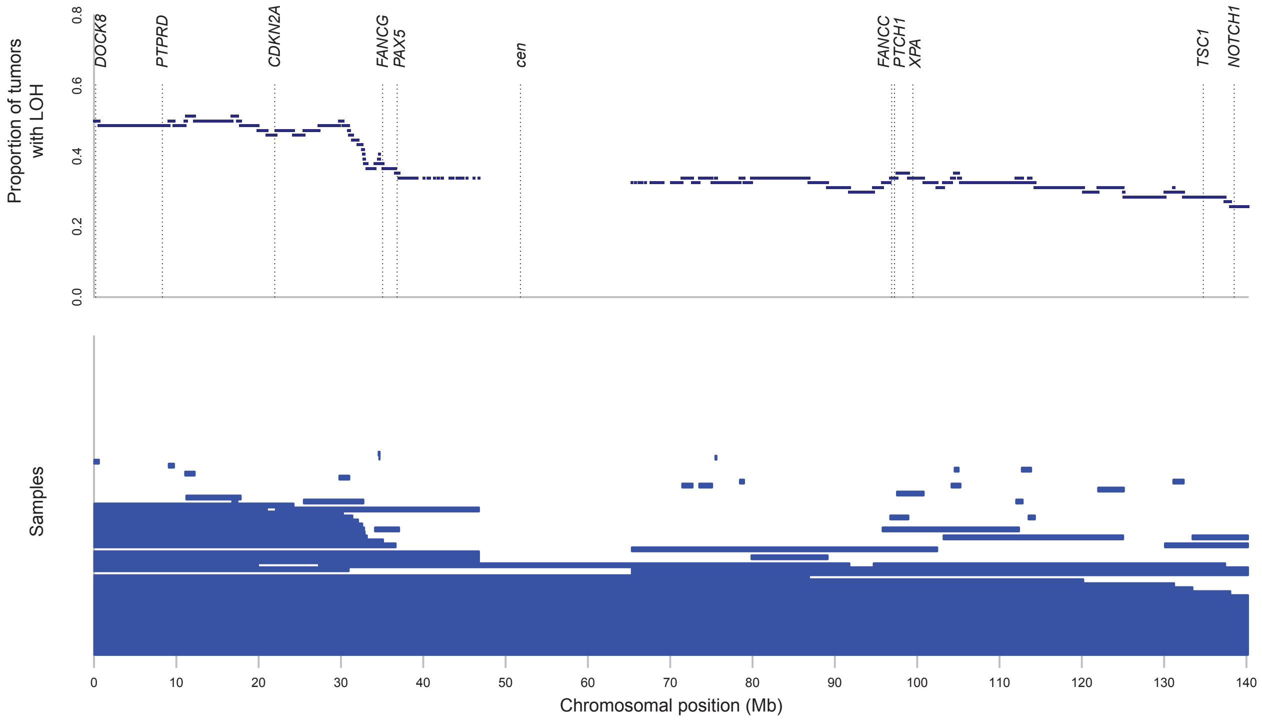
Proportion of tumors with LOH
0.0
0.2
0.4
0.6
0.8
DOCK8
PTPRD
CDKN2A
FANCG
PAX5
cen
FANCC
PTCH1
XPA
TSC1
NOTCH1
Samples
0
10
20
30
40
50
60
70
80
90
100
110
120
130
140
Chromosomal position (Mb)

Chr 10

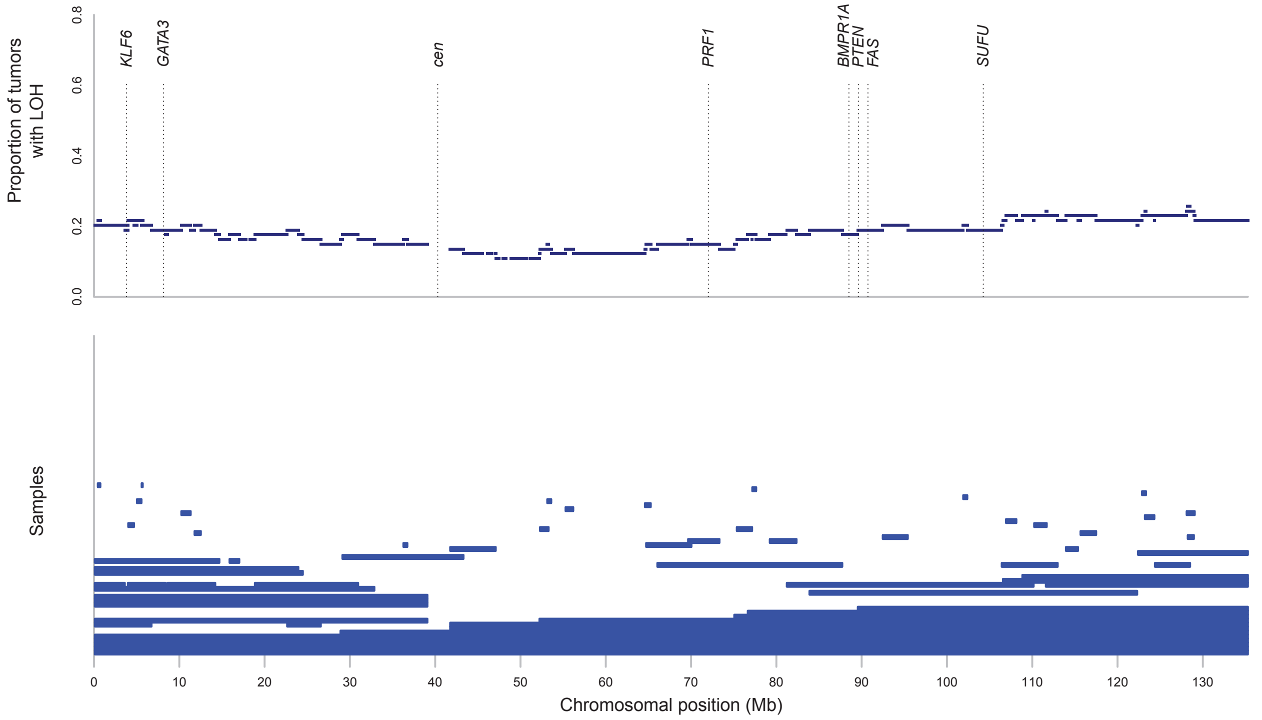
Proportion of tumors with LOH
0.0
0.2
0.4
0.6
0.8
KLF6
GATA3
cen
PRF1
BMPR1A
PTEN
FAS
SUFU
Samples
0
10
20
30
40
50
60
70
80
90
100
110
120
130
Chromosomal position (Mb)

Chr 11

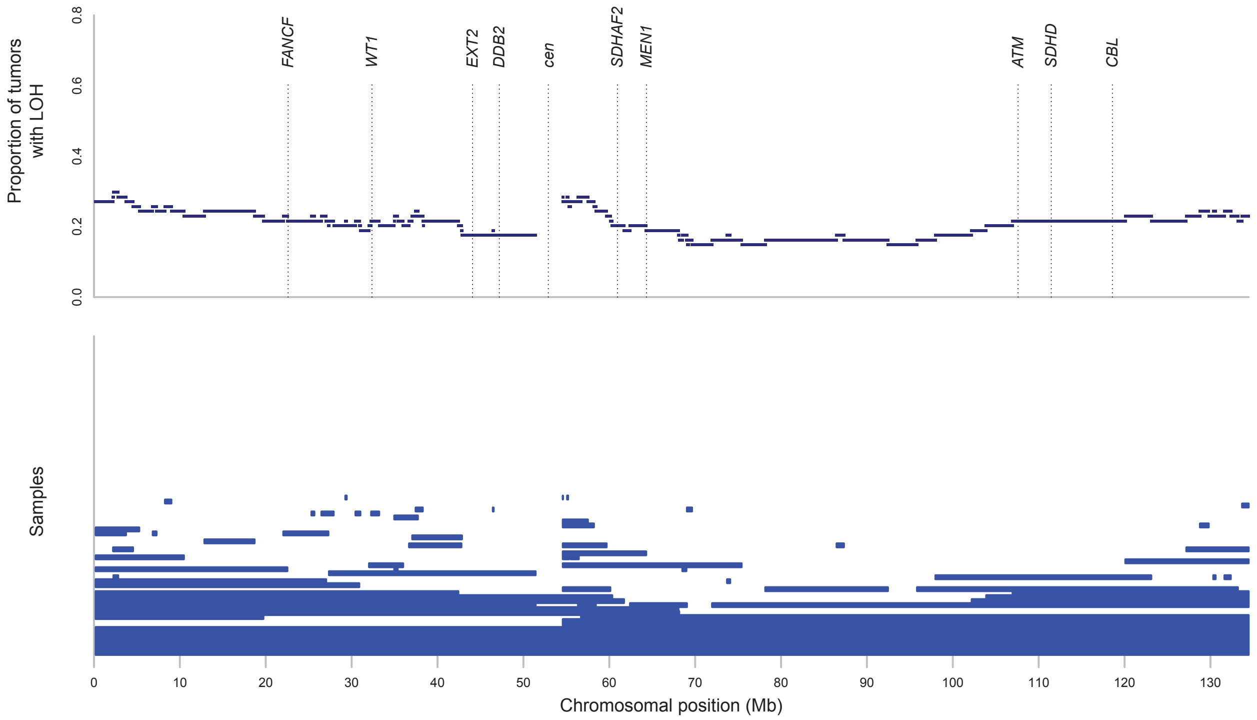

Proportion of tumors with LOH
0.0
0.2
0.4
0.6
0.8
FANCF
WT1
EXT2
DDB2
cen
SDHAF2
MEN1
ATM
SDHD
CBL
Samples
0
10
20
30
40
50
60
70
80
90
100
110
120
130
Chromosomal position (Mb)

Chr 12

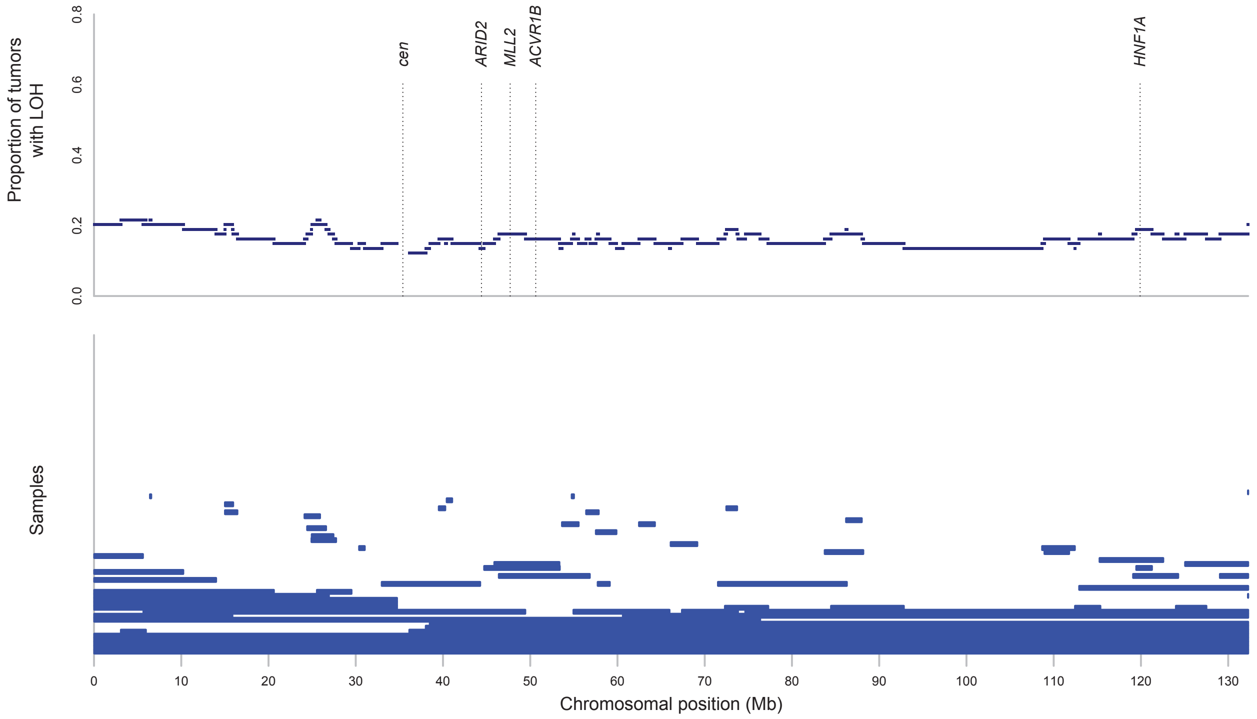

Proportion of tumors with LOH
0.0
0.2
0.4
0.6
0.8
cen
ARID2
MLL2
ACVR1B
HNF1A
Samples
0
10
20
30
40
50
60
70
80
90
100
110
120
130
Chromosomal position (Mb)

Chr 13

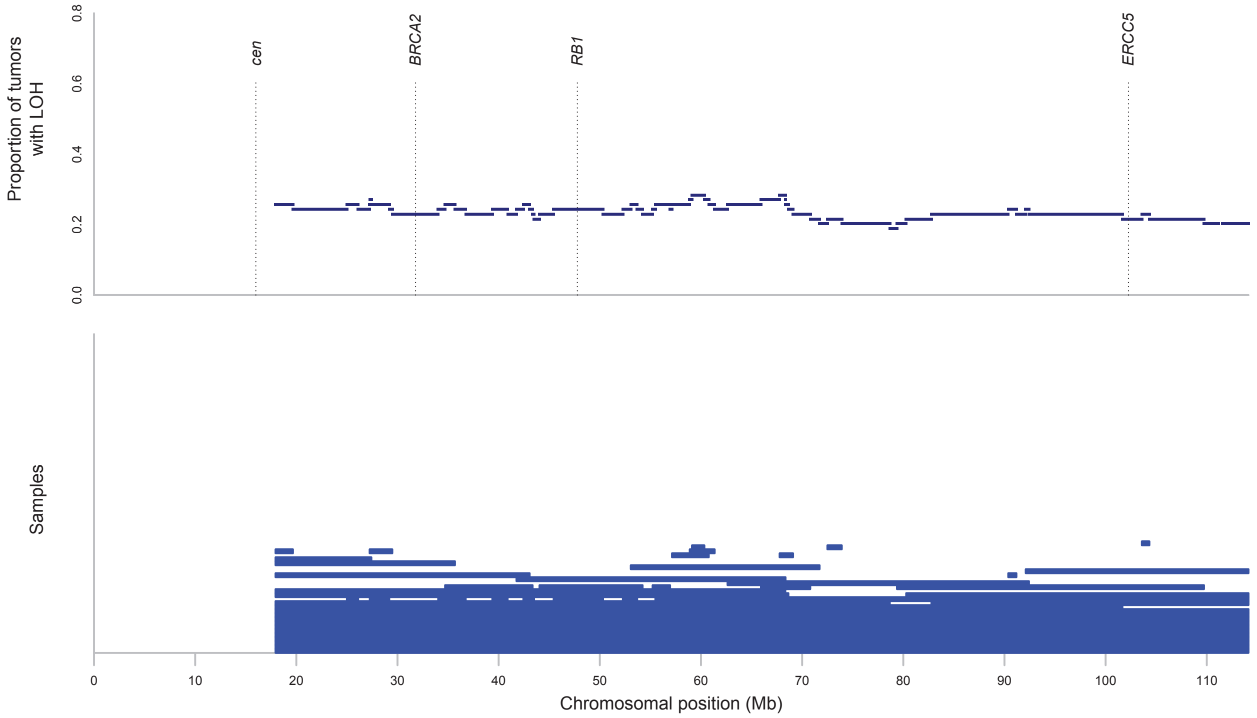

Proportion of tumors with LOH
0.0
0.2
0.4
0.6
0.8
cen
BRCA2
RB1
ERCC5
Samples
0
10
20
30
40
50
60
70
80
90
100
110
Chromosomal position (Mb)

Chr 14

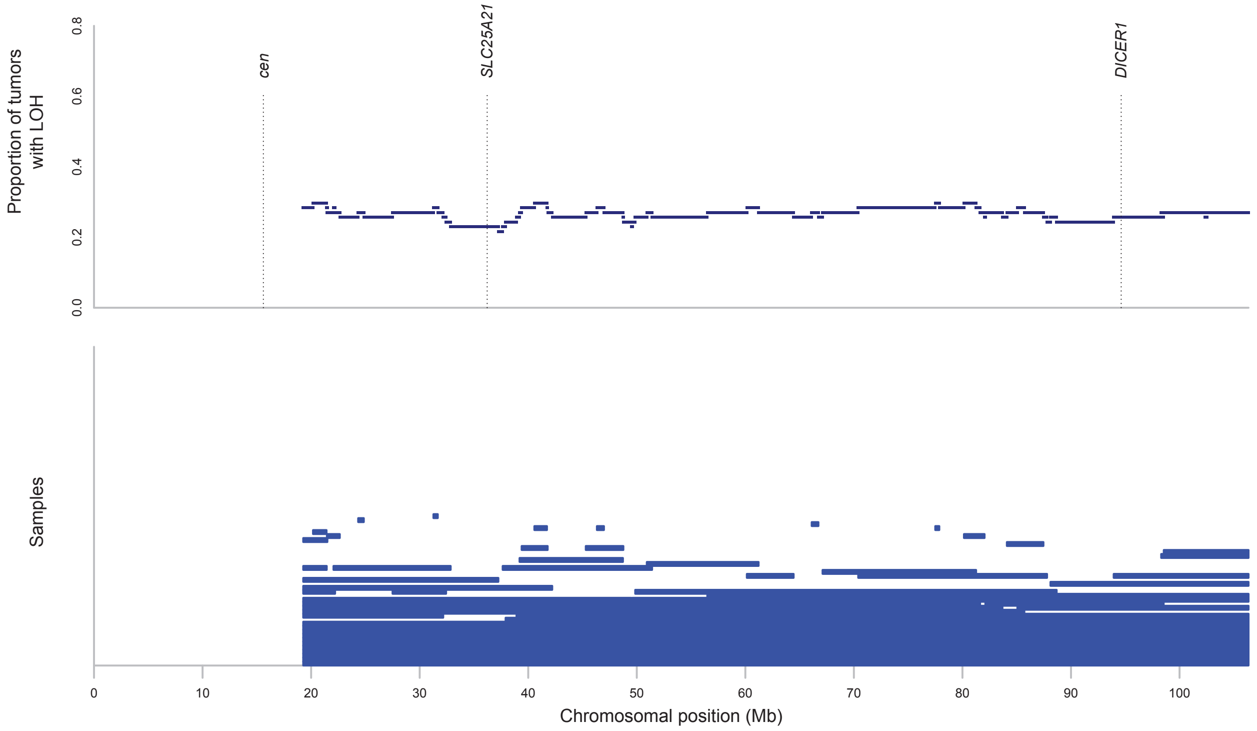

Proportion of tumors with LOH
0.0
0.2
0.4
0.6
0.8
cen
SLC25A21
DICER1
Samples
0
10
20
30
40
50
60
70
80
90
100
Chromosomal position (Mb)

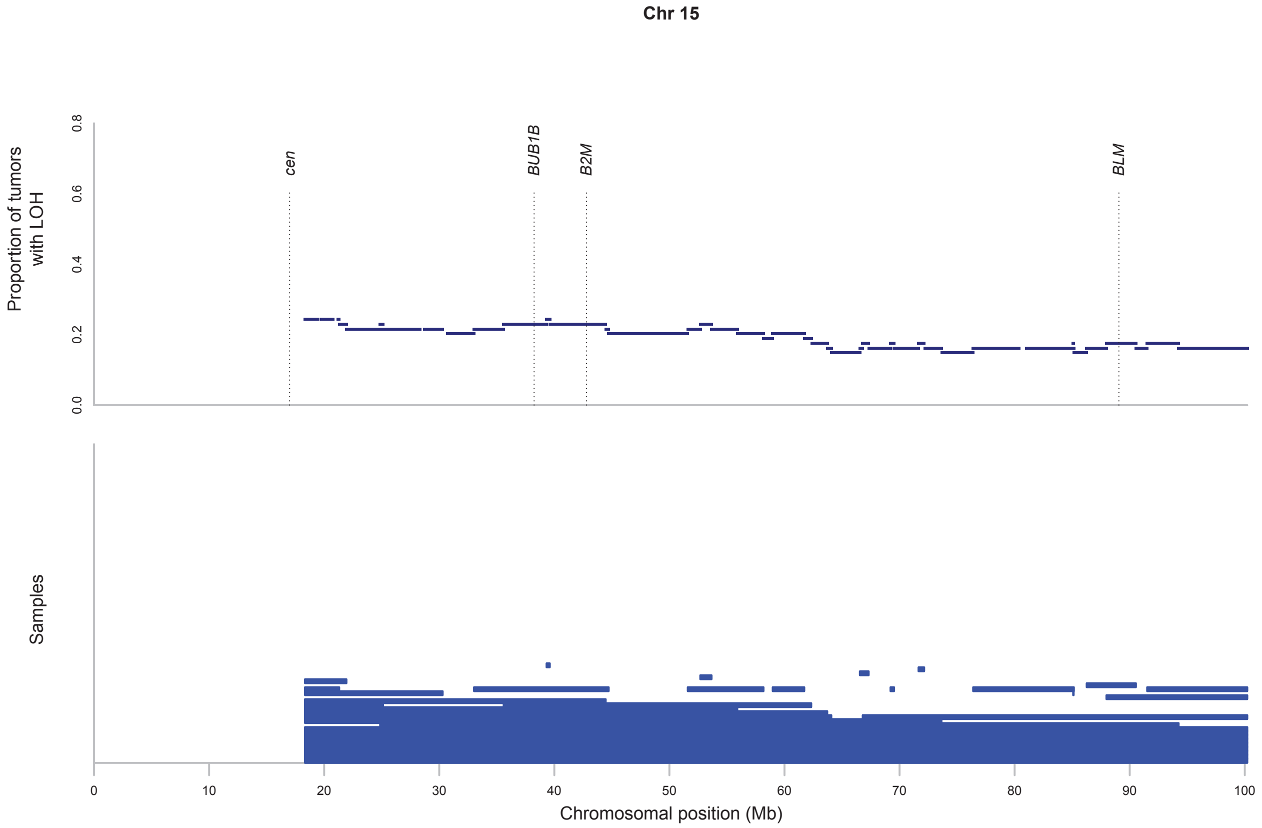
Chr 15
Proportion of tumors with LOH
0.0
0.2
0.4
0.6
0.8
cen
BUB1B
B2M
BLM
Samples
0
10
20
30
40
50
60
70
80
90
100
Chromosomal position (Mb)

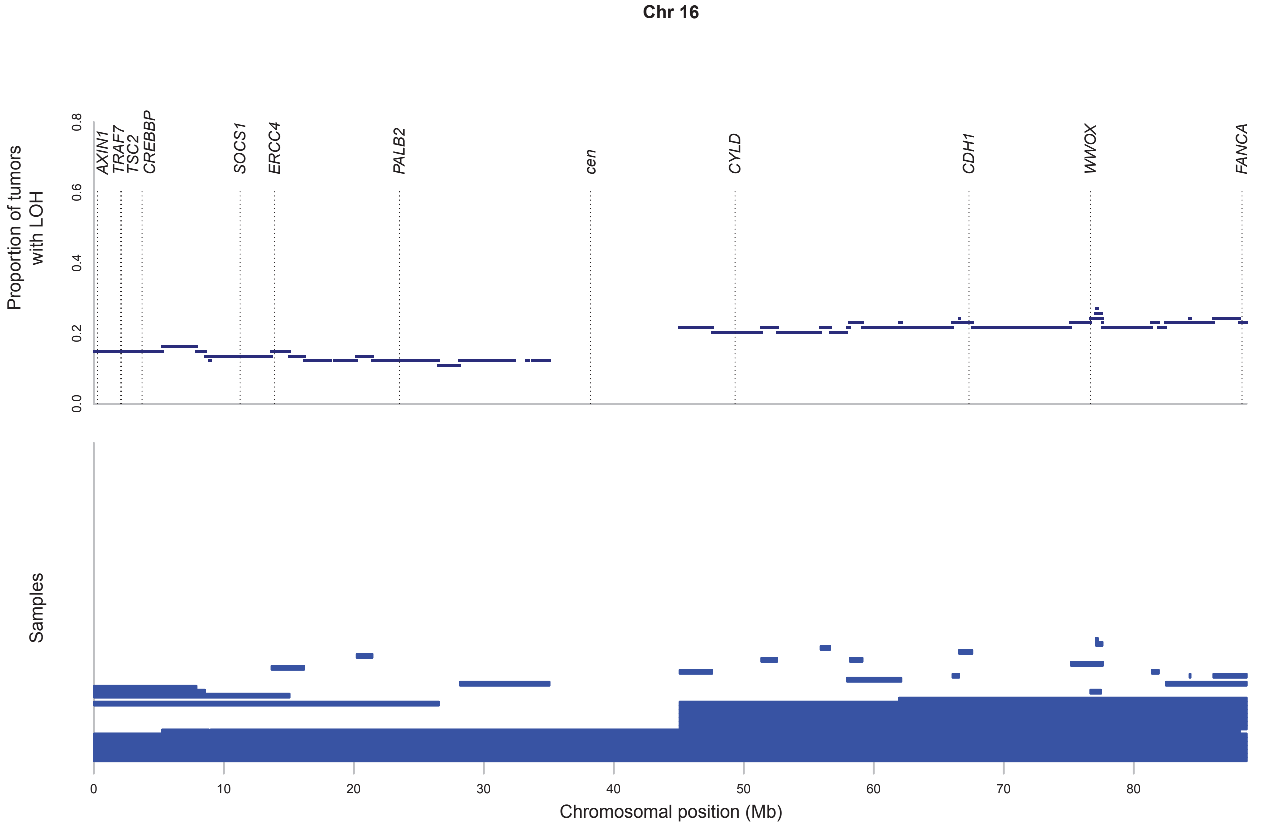
Chr 16
Proportion of tumors with LOH
0.0
0.2
0.4
0.6
0.8
AXIN1
TRAF7
TSC2
CREBBP
SOCS1
ERCC4
PALB2
cen
CYLD
CDH1
WWOX
FANCA
Samples
0
10
20
30
40
50
60
70
80
Chromosomal position (Mb)

Chr 17

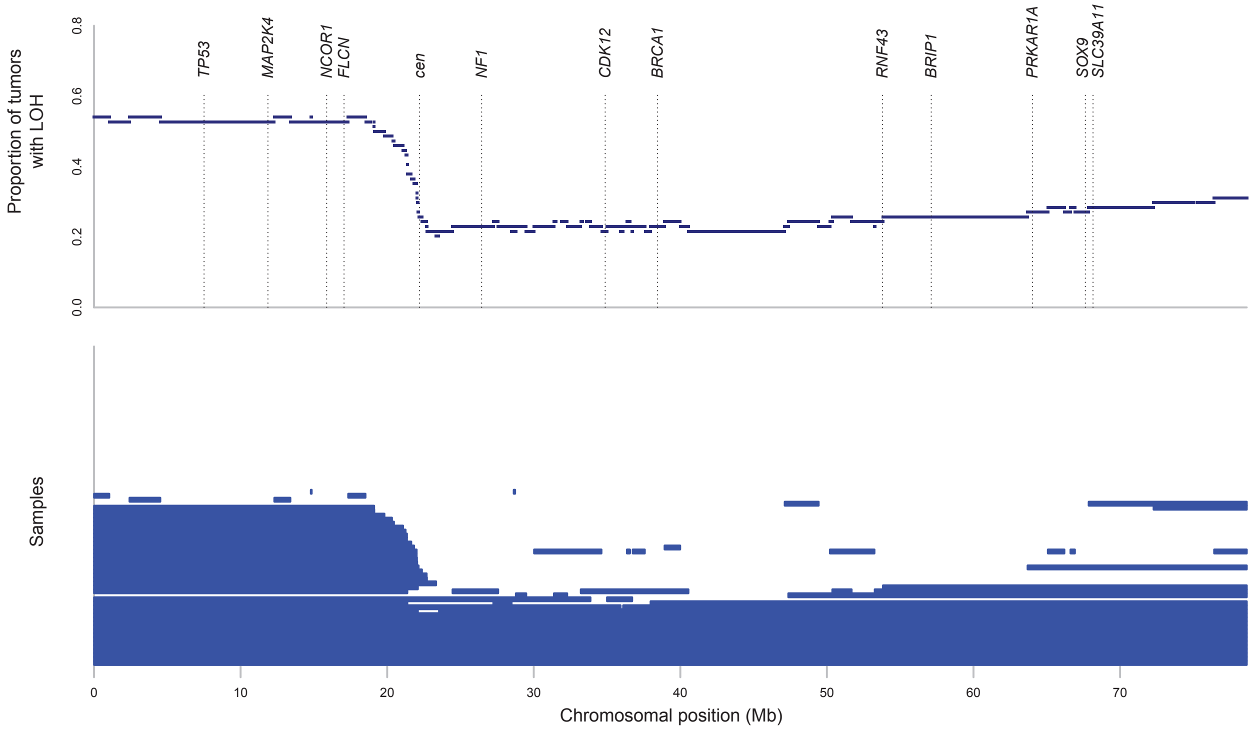

Proportion of tumors with LOH
0.0
0.2
0.4
0.6
0.8
TP53
MAP2K4
NCOR1
FLCN
cen
NF1
CDK12
BRCA1
RNF43
BRIP1
PRKAR1A
SOX9
SLC39A11
Samples
0
10
20
30
40
50
60
70
Chromosomal position (Mb)

Chr 18

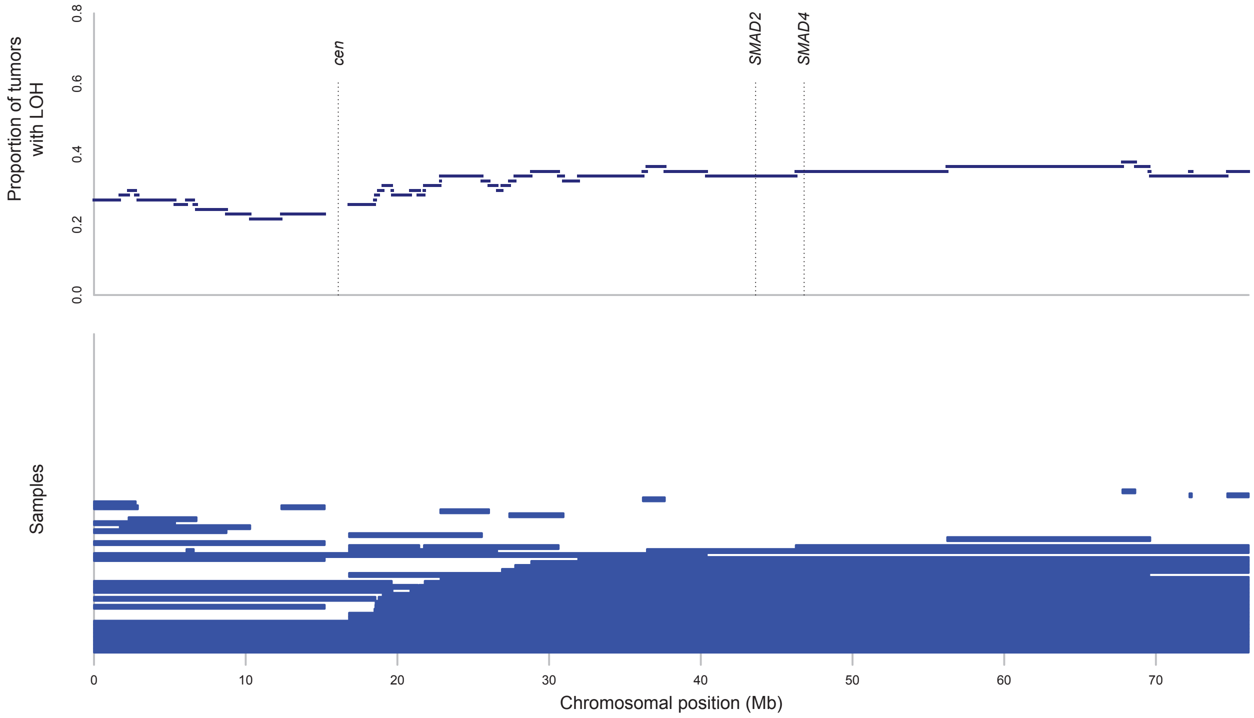

Proportion of tumors with LOH
0.0
0.2
0.4
0.6
0.8
cen
SMAD2
SMAD4
Samples
0
10
20
30
40
50
60
70
Chromosomal position (Mb)

Chr 19

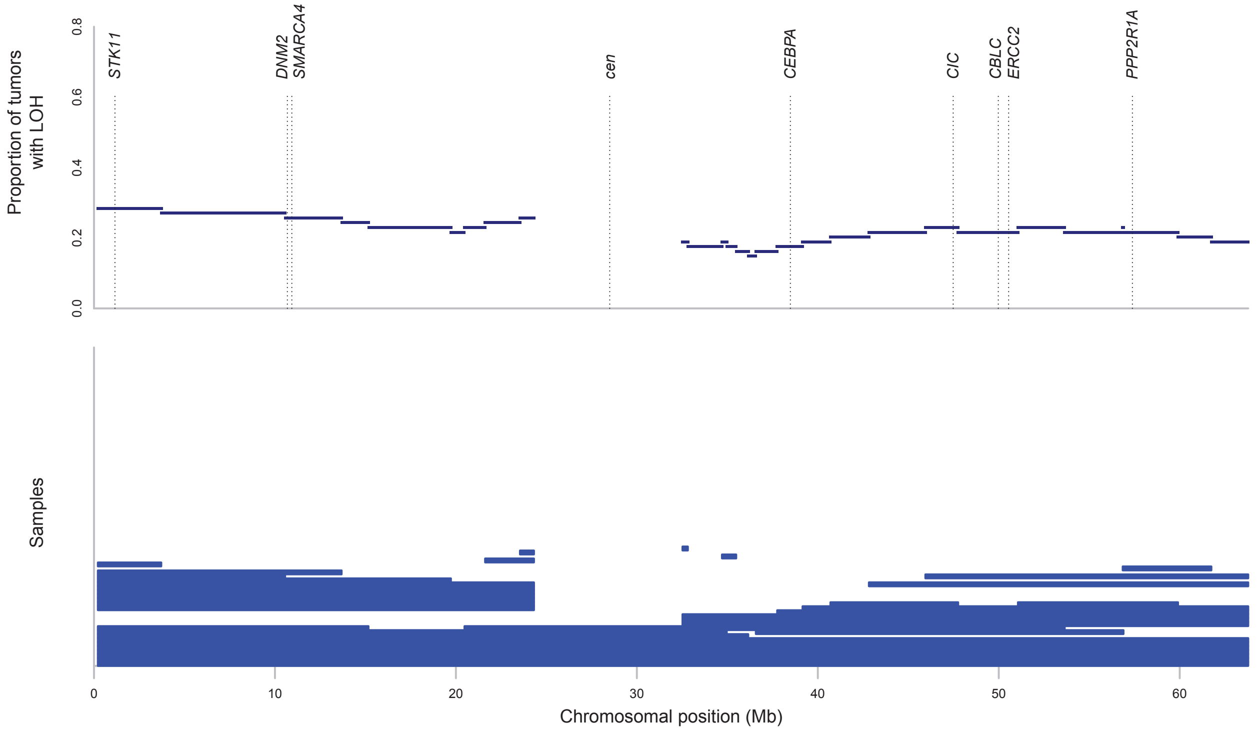

Proportion of tumors with LOH
0.0
0.2
0.4
0.6
0.8
STK11
DNM2
SMARCA4
cen
CEBPA
CIC
CBLC
ERCC2
PPP2R1A
Samples
0
10
20
30
40
50
60
Chromosomal position (Mb)

Chr 20

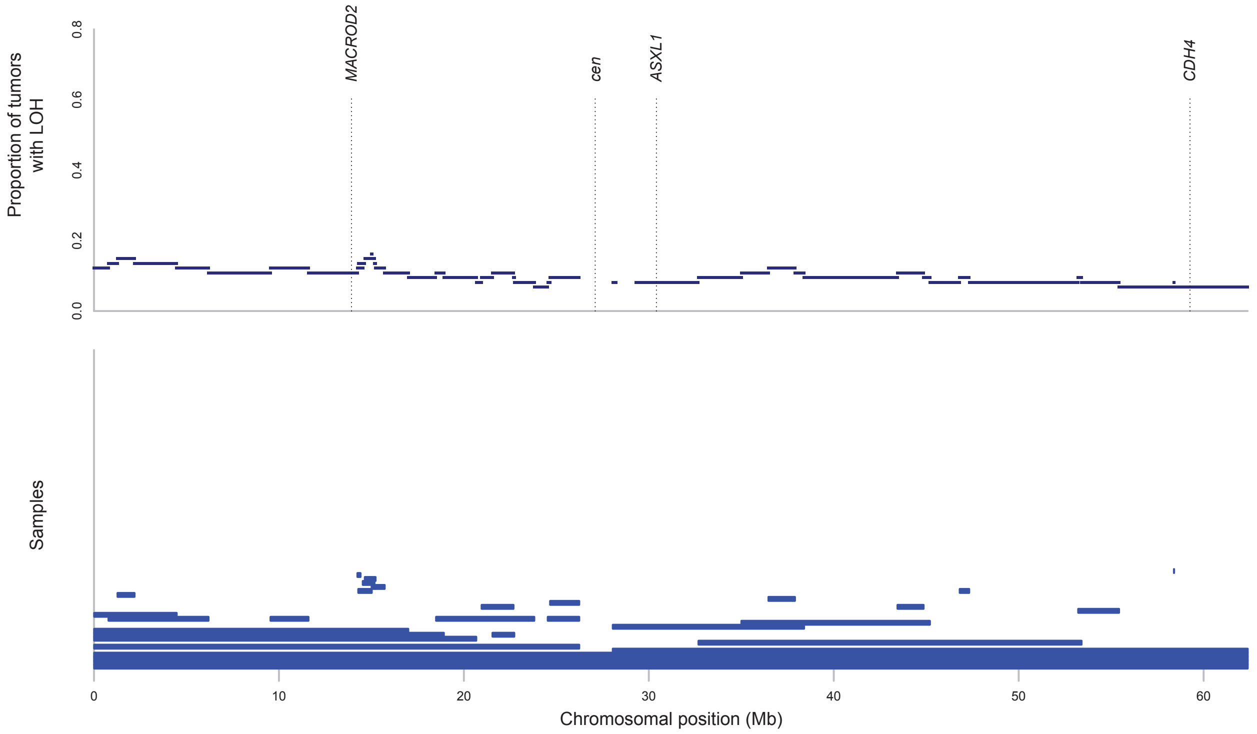

Proportion of tumors with LOH
0.0
0.2
0.4
0.6
0.8
MACROD2
cen
ASXL1
CDH4
Samples
0
10
20
30
40
50
60
Chromosomal position (Mb)

Chr 21

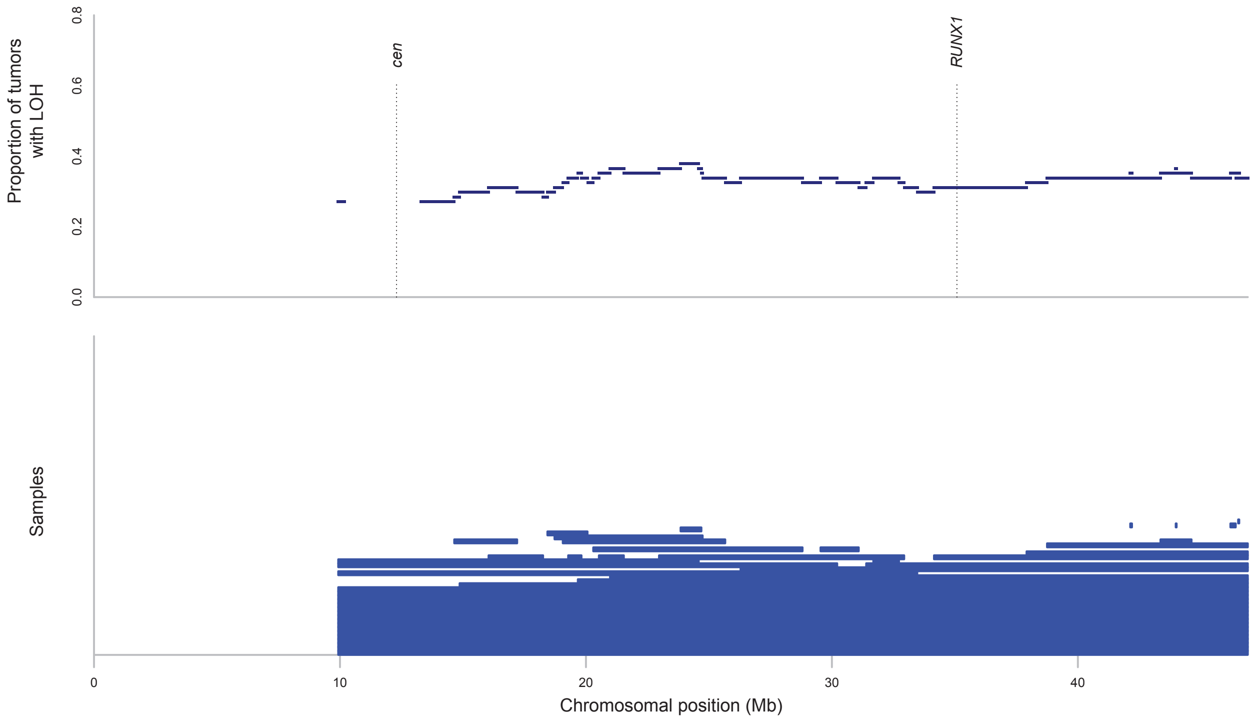

Proportion of tumors with LOH
0.0
0.2
0.4
0.6
0.8
cen
RUNX1
Samples
0
10
20
30
40
Chromosomal position (Mb)

Chr 22

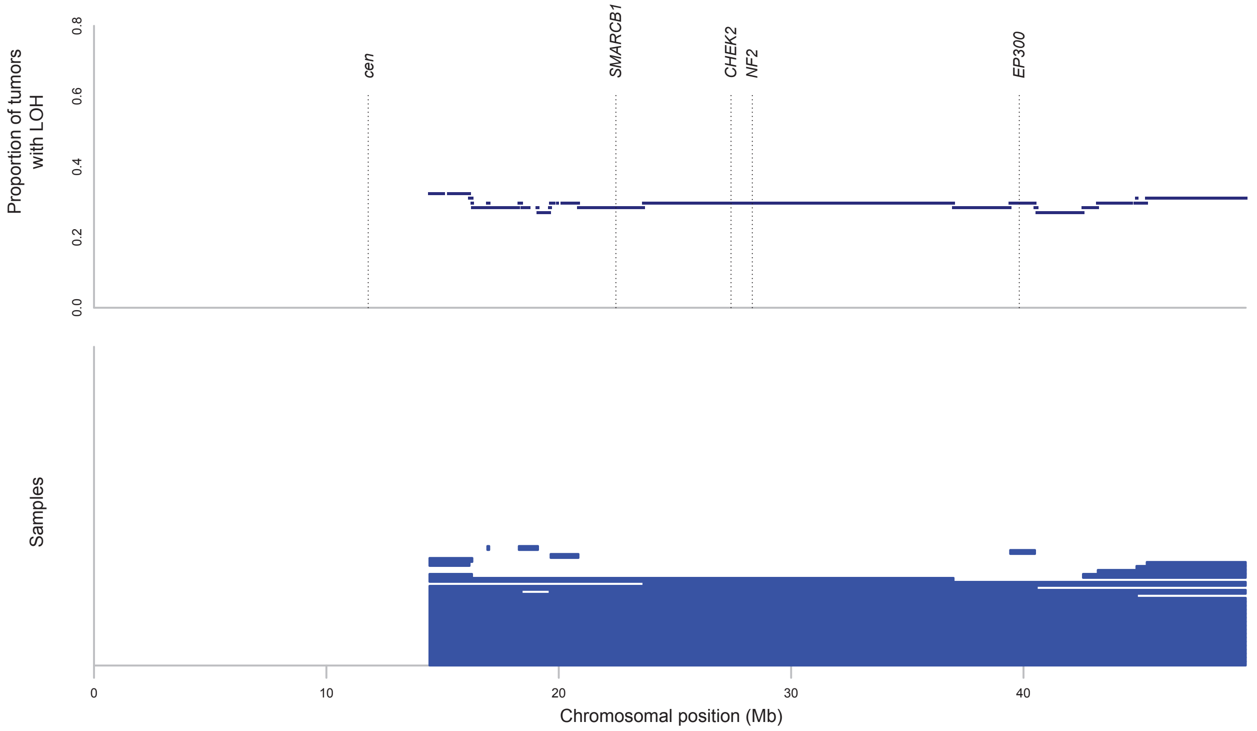

Proportion of tumors with LOH
0.0
0.2
0.4
0.6
0.8
cen
SMARCB1
CHEK2
NF2
EP300
Samples
0
10
20
30
40
Chromosomal position (Mb)

Fig. S9. (a) The proportion of copy-number loss per tumor. (b) The number of STOP genes subject to copy-number loss per tumor. (c) The number or CYCLOPS genes subject to subject to copy-number loss per tumor. The blue lines mark the medians. Copy-number loss is defined as a region where genomic copy number is < 0.7 times the average ploidy.

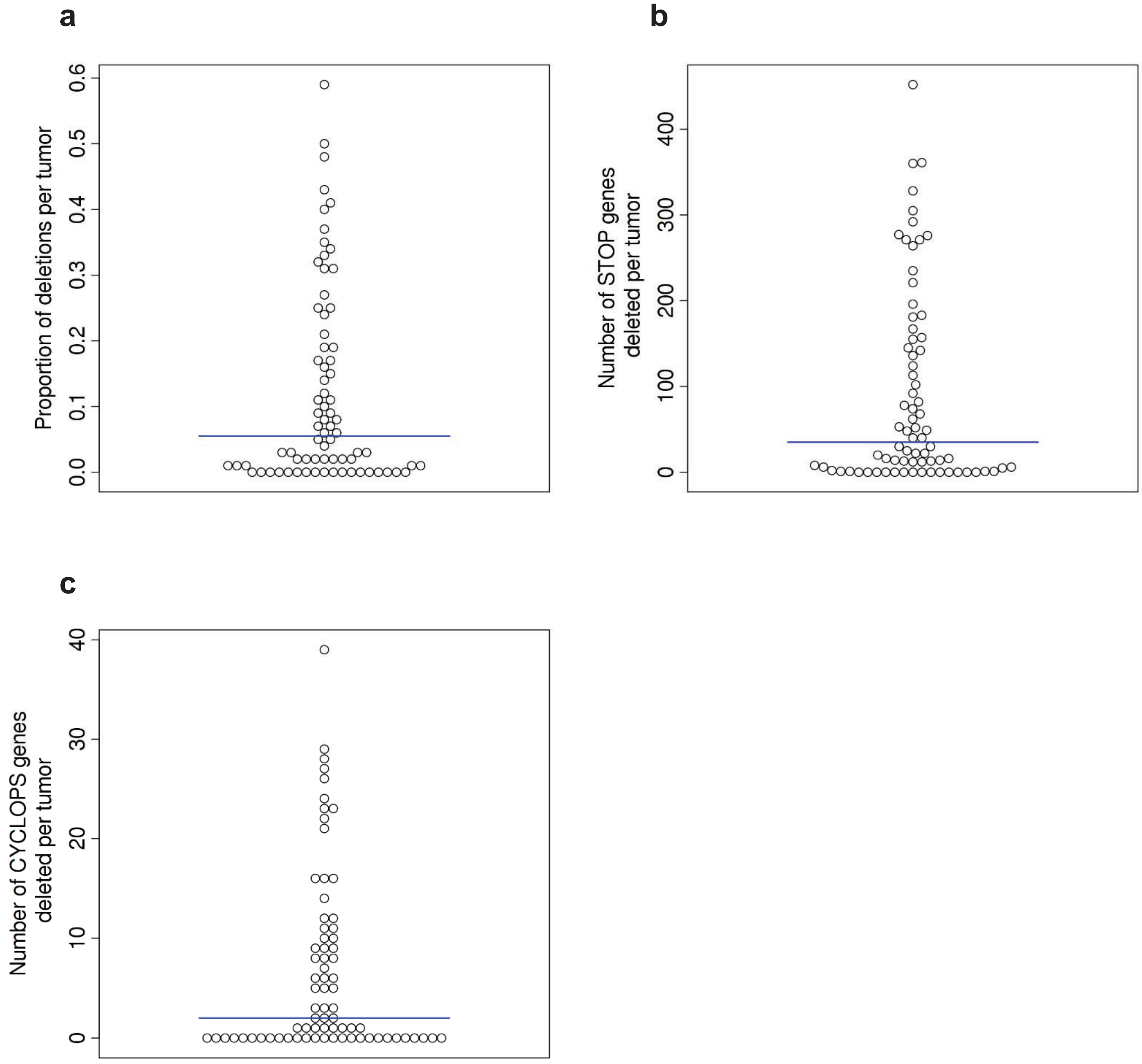

Fig. S10. Copy-number loss of CYCLOPS genes *EEF2, ETFDH* and *ENC1*.
LRR, BAF and allele-specific copy number as inferred by ASCAT in tumors in which one or two of these genes show copy-number loss. Only the chromosomes that have these genes are shown. In each instance except possibly one, examination of the LRR and BAF plots strongly supports ASCATs assessment of copy-number loss. The exception is *EEF2* in tumor 990412, where the decrease in LRR is rather slight. Recall that in most tumors there is an admixture of DNA from non-malignant cells, which reduces the amplitude of changes in LRRs (and BAFs).

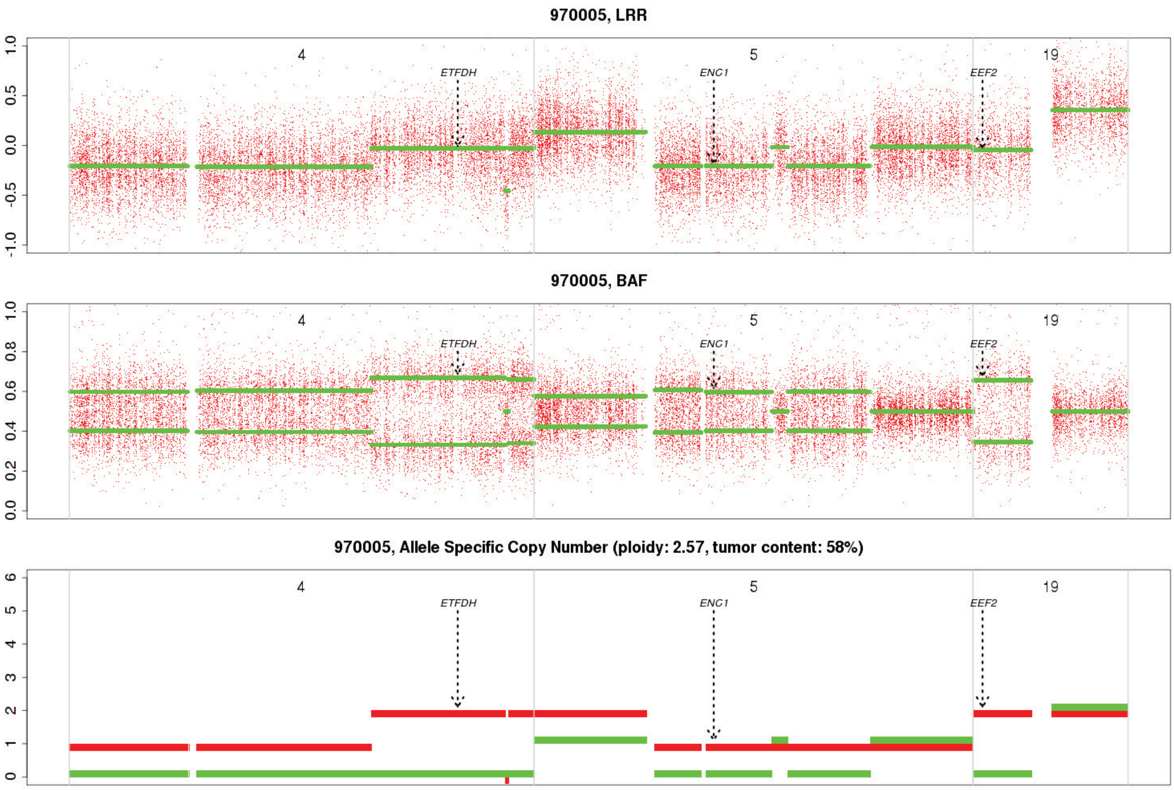

Sample 970005, *ENC1* gene shows copy-number loss.

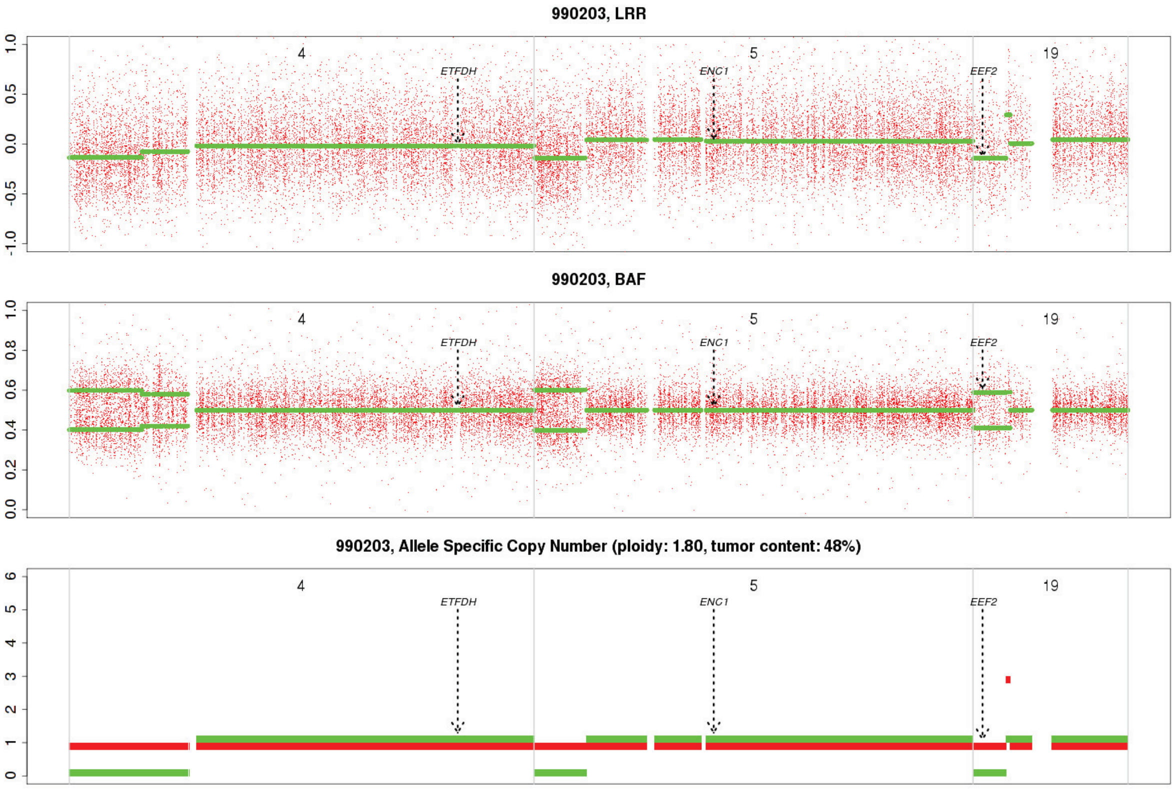

Sample 990203, *EEF2* gene shows copy-number loss.

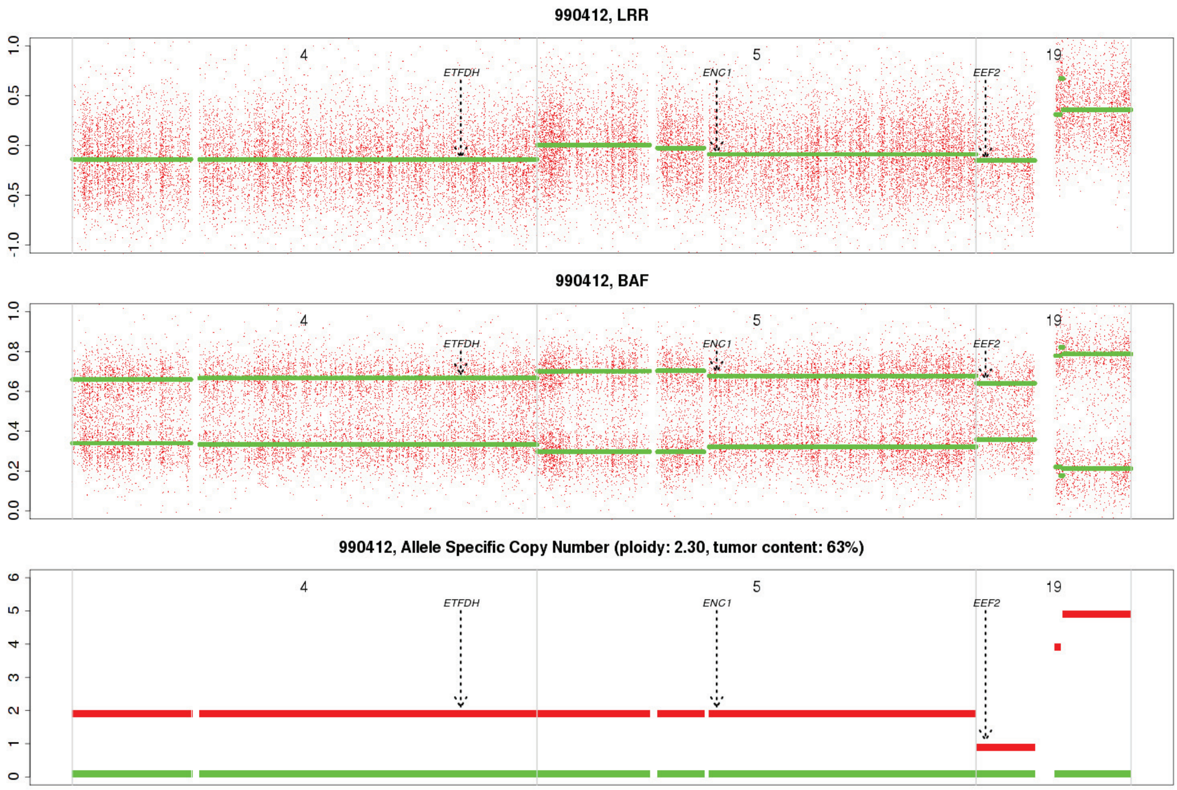

Sample 990412, *EEF2* gene shows copy-number loss.

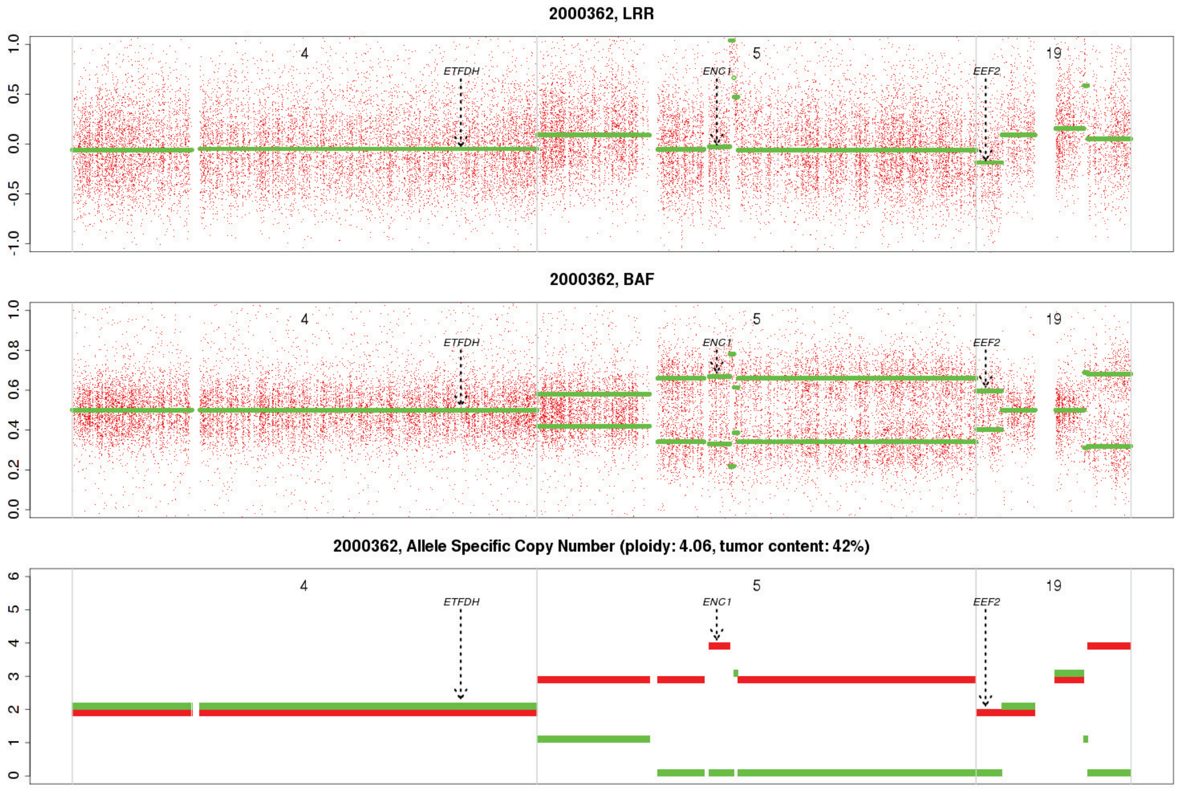

Sample 2000362, *EEF2* gene shows copy-number loss.

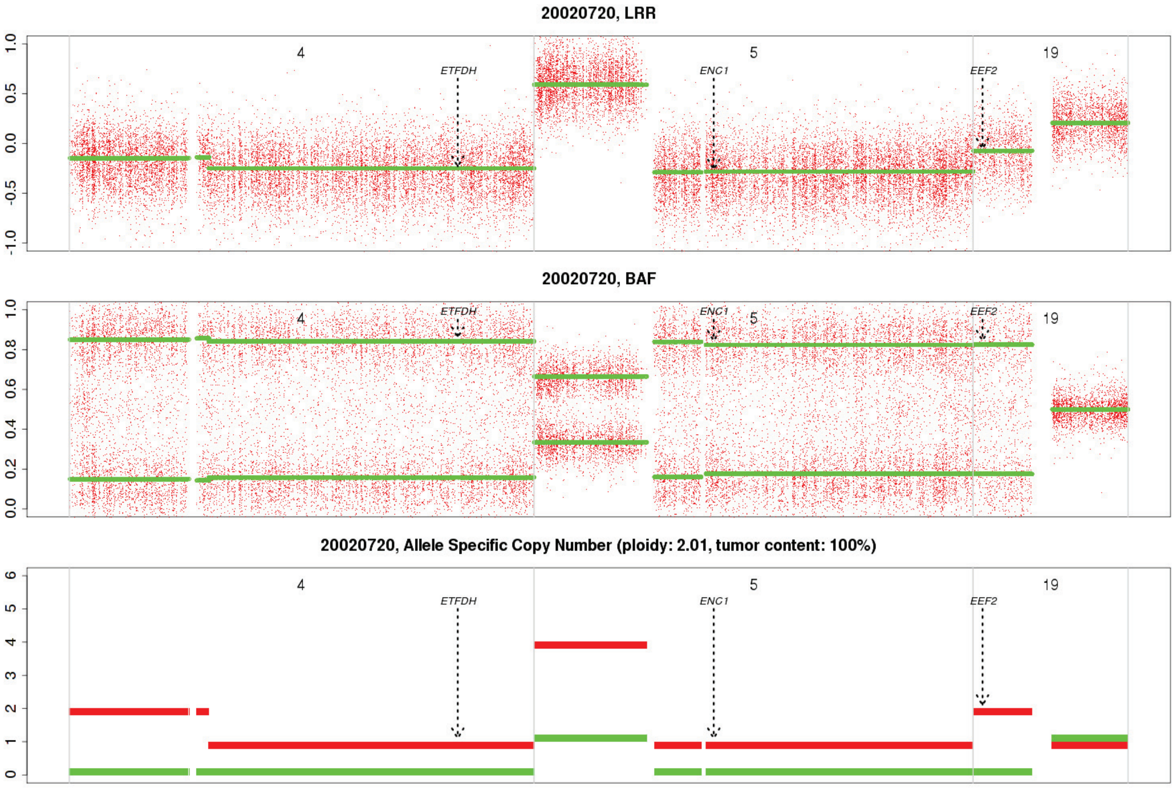

Sample 20020720, *ETFDH* and *ENC1* genes show copy-number loss.

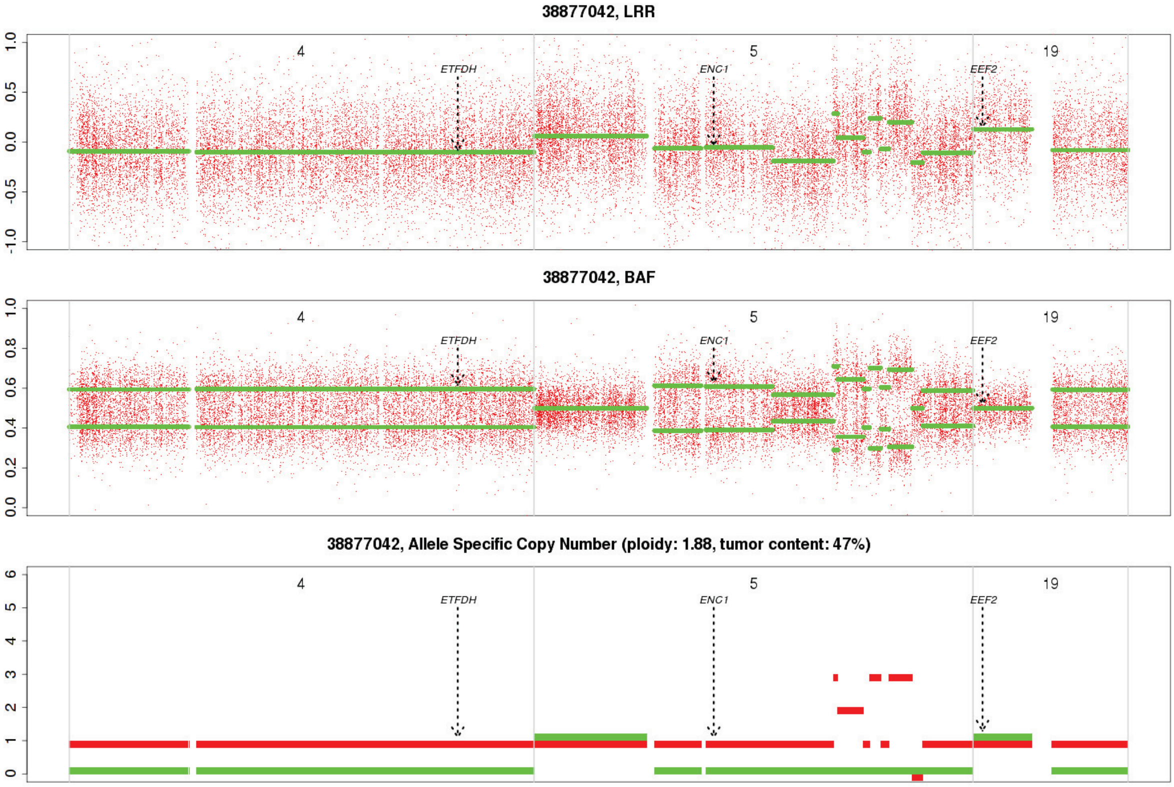

Sample 38877042, *ETFDH* and *ENC1* genes show copy-number loss.

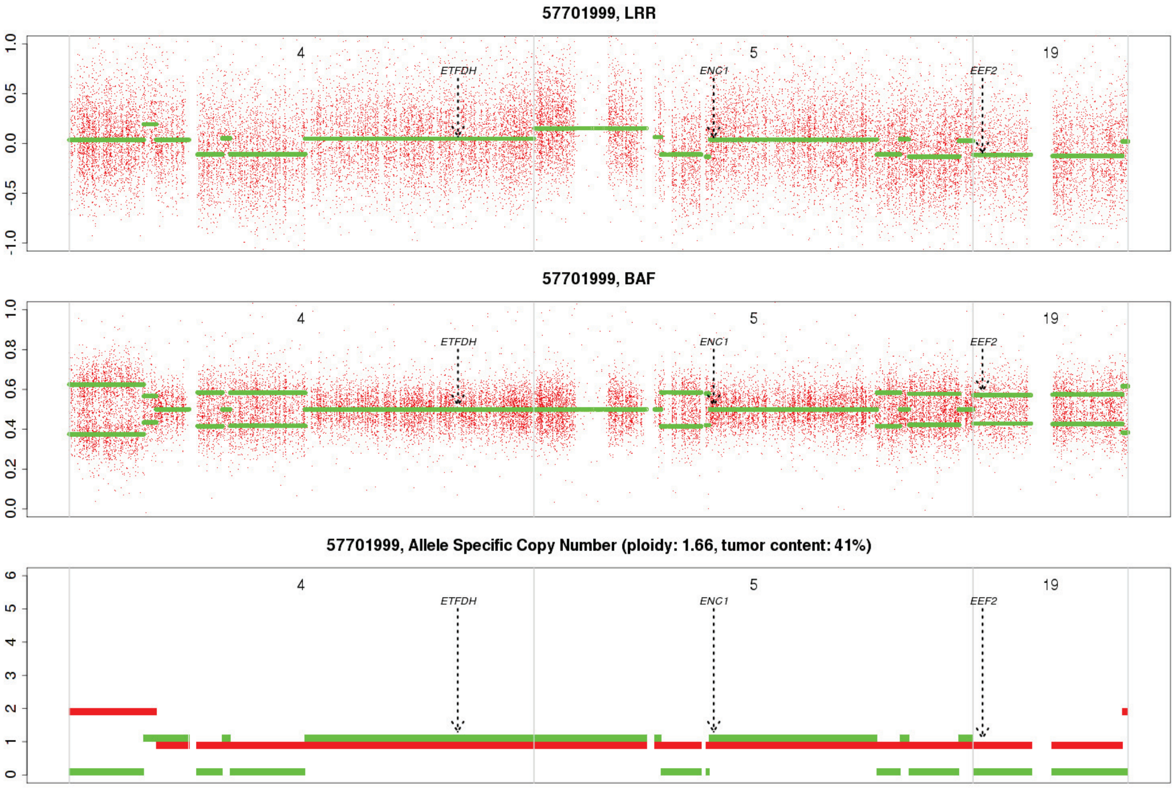

Sample 57701999, *EEF2* gene shows copy-number loss

Fig. S11. GSEAs of GO genes in regions of lower copy-number in gastric adenocarcinoma. Reference [4] experimentally identified a list of "GO" genes—genes whose depletion limits proliferation—and determined that GO genes were underrepresented in regions of recurrent deletion. We carried out GSEAs for the GO genes analogous to the GSEAs for STOP genes presented in the main text. We used the stringent set of GO genes from reference [4]: namely the genes that reduced cell proliferation and viability by a factor ≥ 1.5 in five out of nine cell lines (from Supplementary Table S11 in reference [4]).
(a) Running enrichment score for the GO gene set against the list of genes that have significant positive correlation between their average relative copy number and expression, ranked by the genes' average relative copy number across all 74 samples, and then, to break ties, by the correlation coefficient between their average relative copy number and expression.
(b) Vertical black lines mark locations of members of the GO gene set in the ranked list of genes. We found no enrichment or impoverishment (p = 0.191).

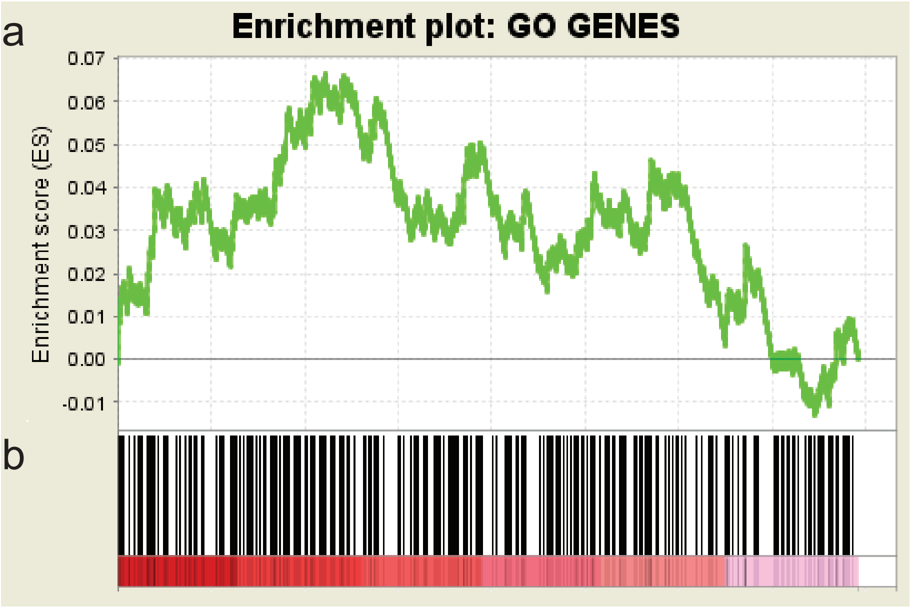

Supplementary References

1. Popova T, Manie E, Stoppa-Lyonnet D, Rigaill G, Barillot E, Stern MH. Genome Alteration Print (GAP): a tool to visualize and mine complex cancer genomic profiles obtained by SNP arrays. Genome Biol. 2009;10:R128
2. Li A, Liu Z, Lezon-Geyda K, Sarkar S, Lannin D, Schulz V, et al. GPHMM: an integrated hidden Markov model for identification of copy number alteration and loss of heterozygosity in complex tumor samples using whole genome SNP arrays. Nucleic Acids Res. 2011;39:4928-41
3. Barretina J, Caponigro G, Stransky N, Venkatesan K, Margolin AA, Kim S, et al. The Cancer Cell Line Encyclopedia enables predictive modelling of anticancer drug sensitivity. Nature. 2012;483:603-7
4. Solimini NL, Xu Q, Mermel CH, Liang AC, Schlabach MR, Luo J, et al. Recurrent hemizygous deletions in cancers may optimize proliferative potential. Science. 2012;337:104-9
