# Supplementary material for: Abundant copy-number loss of CYCLOPS and STOP genes in gastric adenocarcinoma
Source: Gastric Cancer. 2015 Jul 24;19:453–65. doi: 10.1007/s10120-015-0514-z (PMC4824836; doi:10.1007/s10120-015-0514-z)
Supplement: Supplementary file 2 — Supplementary material 2 (PDF 725 kb) [file 10120_2015_514_MOESM2_ESM.pdf]

Original article

# Abundant copy-number loss of CYCLOPS and STOP genes in gastric adenocarcinoma

Ioana Cutcutache*[,1,2], Alice Yingting Wu*[,1, 2,3], Yuka Suzuki[1,2], John Richard McPherson[1,2], Zhengdeng Lei[1,2], Niantao Deng[1,4], Shenli Zhang[1], Wai Keong Wong[5], Khee Chee Soo[5,6], Weng Hoong Chan[5], London Lucien Ooi[5,6], Roy Welsch[3,7], Patrick Tan[1,3,8,9], Steven G. Rozen[1, 2,3]

* These authors contributed equally to this manuscript.

[1] Program in Cancer and Stem Cell Biology, Duke-NUS Graduate Medical School, Singapore

[2] Centre for Computational Biology, Duke-NUS Graduate Medical School, Singapore

[3] Computation and Systems Biology, Singapore-MIT Alliance

[4] NUS Graduate School for Integrative Science and Engineering, National University of Singapore, Singapore

[5] Department of General Surgery, Singapore General Hospital

[6] Division of Surgical Oncology, National Cancer Centre Singapore

[7] Engineering Systems Division and Sloan School of Management, Massachusetts Institute of Technology, MA, USA

[8] Duke-NUS Genome Biology Facility, Duke-NUS Graduate Medical School, Singapore

[9] Genome Institute of Singapore, A*STAR, Singapore

Correspondence: Patrick Tan (gmstanp@duke-nus.edu.sg), Steven Rozen (steve.rozen@duke-nus.edu.sg, +65 9857 3213)

## Supplementary Tables

**Table S1. Clinical and pathological information on tumors studied.**

| Category | Subcategory | All tumors | Tumors with ASCAT estimate | Tumors without ASCAT estimate | P-value, with vs. without ASCAT estimate | Test |
|---|---|---|---|---|---|---|
| Age (years) | Range | 25-92 | 25-92 | 32-88 | 0.238 | t |
| | Median | 67 | 65 | 68 | | |
| Pathologist-estimated tumor content (%) | Range | 0-90 | 0-90 | 5-60 | 0.034 | One-sided Wilcoxon rank-sum |
| | Median | 35 | 60 | 20 | | |
| Gender | Female | 41 | 23 | 18 | 0.15 | Fisher's exact |
| | Male | 72 | 51 | 21 | | |
| Laurén class-ification | Diffuse | 44 | 25 | 19 | 0.152 | Fisher's exact (excluding “Mixed”) |
| | Intestinal | 62 | 44 | 18 | | |
| | Mixed | 7 | 5 | 2 | | |
| TNM Stage | I | 14 | 10 | 4 | 0.961 | Chisq |
| | II | 20 | 13 | 7 | | |
| | III | 61 | 39 | 22 | | |
| | IV | 18 | 12 | 6 | | |
| G-INT / G-DIF subtype | G-INT | 57 | 42 | 15 | 0.062 | Fisher's exact (excluding NC) |
| | G-DIF | 45 | 25 | 20 | | |
| | NC | 11 | 7 | 4 | | |
| Overall survival time (months) | Range | 0.2-178.1 | 0.2-167.0 | 2.27-178.1 | 0.151 | Kaplan-Meier log-rank [a] |
| | Median [a] | 30.0 | 24.6 | 56.9 | | |
| Tumor grade | Poorly differentiated | 69 | 44 | 25 | 0.935 | Fisher’s exact |
| | Moderately differentiated | 40 | 27 | 13 | | |
| | Well differentiated | 4 | 3 | 1 | | |

t: t-test; G-INT: genomic intestinal [1]; G-DIF: genomic diffuse [1]; NC: not classifiable; Chisq: chi-squared test.

[a] Median survival and Kaplan-Meier log-rank tests calculated by the survfit and survdiff functions in the R package “survival” (http://cran.r-project.org/web/packages/survival/index.html) [2].

**Table S3. Values of parameters used in ASCAT analysis.** See notes at end of table for explanations of the parameters.

| Sample ID | ASCAT version | μ factor[a] | Germline BAF limits[b] | Min goodness of fit[c] | Segment length[d] |
|---|---|---|---|---|---|
| 970005 | 2.0 | 1.5 | 0.35, 0.65 | 85 | 100 |
| 980011 | 2.1 | 1.5 | 0.35, 0.65 | 85 | as determined by ASCAT 2.1 |
| 980021 | 2.0 | 1.5 | 0.35, 0.65 | 85 | 100 |
| 980029 | 2.0 | 1.5 | 0.35, 0.65 | 85 | 100 |
| 980097 | 2.0 | 1.5 | 0.35, 0.65 | 85 | 100 |
| 980156 | 2.0 | 1.5 | 0.35, 0.65 | 85 | 100 |
| 980369 | 2.0 | 1.5 | 0.35, 0.65 | 65 | 100 |
| 980390 | 2.0 | 1.5 | 0.35, 0.65 | 65 | 800 |
| 980401 | 2.0 | 1.5 | 0.35, 0.65 | 85 | 100 |
| 980417 | 2.0 | 1.3 | 0.35, 0.65 | 85 | 100 |
| 980437 | 2.0 | 1.5 | 0.35, 0.65 | 85 | 100 |
| 980447 | 2.0 | 1.5 | 0.35, 0.65 | 65 | 100 |
| 990005 | 2.1 | 1.5 | 0.35, 0.65 | 85 | as determined by ASCAT 2.1 |
| 990010 | 2.0 | 1.9 | 0.35, 0.65 | 85 | 100 |
| 990041 | 2.1 | 1.5 | 0.35, 0.65 | 85 | as determined by ASCAT 2.1 |
| 990044 | 2.0 | 1.5 | 0.35, 0.65 | 85 | 100 |
| 990046 | 2.0 | 1.5 | 0.35, 0.65 | 65 | 100 |
| 990060 | 2.0 | 1.5 | 0.35, 0.65 | 85 | 100 |
| 990069 | 2.0 | 1.5 | 0.35, 0.65 | 85 | 100 |
| 990071 | 2.0 | 1.5 | 0.35, 0.65 | 85 | 100 |
| 990090 | 2.0 | 1.5 | 0.35, 0.65 | 85 | 100 |
| 990097 | 2.0 | 1.3 | 0.35, 0.65 | 85 | 100 |
| 990098 | 2.0 | 1.5 | 0.35, 0.65 | 65 | 800 |
| 990108 | 2.0 | 1.3 | 0.35, 0.65 | 65 | 100 |
| 990111 | 2.1 | 1.5 | 0.35, 0.65 | 85 | as determined by ASCAT 2.1 |
| 990170 | 2.1 | 1.5 | 0.35, 0.65 | 85 | as determined by ASCAT 2.1 |
| 990172 | 2.0 | 1.9 | 0.35, 0.65 | 75 | 100 |
| 990195 | 2.0 | 1.5 | 0.35, 0.65 | 85 | 100 |
| 990203 | 2.0 | 1.5 | 0.35, 0.65 | 85 | 100 |
| 990228 | 2.0 | 1.5 | 0.35, 0.65 | 85 | 100 |
| 990275 | 2.1 | 1.5 | 0.35, 0.65 | 85 | as determined by ASCAT 2.1 |
| 990300 | 2.0 | 1.3 | 0.35, 0.65 | 85 | 100 |
| 990339 | 2.1 | 1.5 | 0.35, 0.65 | 85 | as determined by ASCAT 2.1 |
| 990355 | 2.0 | 1.5 | 0.35, 0.65 | 85 | 100 |
| 990396 | 2.0 | 1.5 | 0.35, 0.65 | 85 | 100 |
| 990412 | 2.0 | 1.5 | 0.35, 0.65 | 65 | 100 |
| 990474 | 2.0 | 1.5 | 0.35, 0.65 | 85 | 100 |
| 990475 | 2.0 | 1.9 | 0.35, 0.65 | 85 | 100 |
| 990489 | 2.1 | 1.5 | 0.35, 0.65 | 85 | as determined by ASCAT 2.1 |
| 990515 | 2.0 | 1.5 | 0.35, 0.65 | 85 | 100 |
| 2000040 | 2.1 | 1.5 | 0.35, 0.65 | 85 | as determined by ASCAT 2.1 |
| 2000068 | 2.0 | 1.5 | 0.35, 0.65 | 85 | 100 |
| 2000085 | 2.1 | 1.5 | 0.35, 0.65 | 85 | as determined by ASCAT 2.1 |
| 2000088 | 2.0 | 1.5 | 0.35, 0.65 | 85 | 100 |
| 2000169 | 2.0 | 1.5 | 0.35, 0.65 | 65 | 100 |
| 2000175 | 2.0 | 1.5 | 0.35, 0.65 | 85 | 100 |
| 2000201 | 2.0 | 1.5 | 0.35, 0.65 | 85 | 100 |
| 2000242 | 2.1 | 1.5 | 0.35, 0.65 | 85 | as determined by ASCAT 2.1 |

| | | | | | |
|---|---|---|---|---|---|
| 2000286 | 2.1 | 1.5 | 0.35, 0.65 | 85 | as determined by ASCAT 2.1 |
| 2000303 | 2.0 | 1.5 | 0.3, 0.7 | 85 | 100 |
| 2000362 | 2.0 | 1.5 | 0.35, 0.65 | 85 | 100 |
| 2000403 | 2.0 | 1.5 | 0.35, 0.65 | 65 | 100 |
| 2000433 | 2.0 | 1.5 | 0.35, 0.65 | 85 | 100 |
| 2000441 | 2.0 | 1.3 | 0.35, 0.65 | 85 | 100 |
| 2000877 | 2.0 | 1.5 | 0.35, 0.65 | 85 | 100 |
| 2000892 | 2.0 | 1.5 | 0.35, 0.65 | 85 | 100 |
| 20020011 | 2.0 | 1.9 | 0.35, 0.65 | 85 | 100 |
| 20020448 | 2.0 | 1.5 | 0.35, 0.65 | 65 | 800 |
| 20020720 | 2.0 | 1.5 | 0.35, 0.65 | 85 | 100 |
| 20263644 | 2.1 | 1.5 | 0.35, 0.65 | 85 | as determined by ASCAT 2.1 |
| 32226415 | 2.1 | 1.5 | 0.35, 0.65 | 85 | as determined by ASCAT 2.1 |
| 38877042 | 2.0 | 1.5 | 0.35, 0.65 | 85 | 100 |
| 46404174 | 2.1 | 1.5 | 0.35, 0.65 | 85 | as determined by ASCAT 2.1 |
| 47492137 | 2.0 | 1.9 | 0.35, 0.65 | 85 | 100 |
| 57689477 | 2.0 | 1.5 | 0.35, 0.65 | 85 | 100 |
| 57701999 | 2.0 | 1.5 | 0.35, 0.65 | 85 | 100 |
| 58947266 | 2.0 | 1.5 | 0.35, 0.65 | 85 | 100 |
| 61669256 | 2.0 | 1.5 | 0.35, 0.65 | 85 | 100 |
| 66811693 | 2.0 | 1.5 | 0.35, 0.65 | 85 | 100 |
| 73291145 | 2.0 | 1.5 | 0.35, 0.65 | 85 | 100 |
| 76629543 | 2.0 | 1.5 | 0.35, 0.65 | 80 | 100 |
| 87622942 | 2.0 | 1.3 | 0.35, 0.65 | 85 | 100 |
| 91228050 | 2.0 | 1.9 | 0.35, 0.65 | 85 | 100 |
| 96141474 | 2.1 | 1.5 | 0.35, 0.65 | 85 | as determined by ASCAT 2.1 |

[a] μ factor is used by the segmentation algorithm to determine if the BAF is 0.5 or if there is allelic imbalance at that segment.

[b] Germline BAF limits are used to remove from analysis those SNPs that are not heterozygous in the non-malignant samples; any probes with BAF values above or below these limits are considered homozygous.

[c] Min goodness of fit is the minimum goodness of fit allowed for a sample; ASCAT evaluates the goodness of fit for a grid of possible values of average ploidy and tumor content, then picks the optimal solution which should have a goodness of fit value higher than this parameter.

[d] Segment length is used by the segmentation algorithm in ASCAT 2.0, and refers to the number of informative probes in a segment.

**Table S4. Tumors for which ASCAT was unable to estimate allele-specific copy numbers.**

| Sample ID | Problem |
|---|---|
| 970003 | Excessively variable LRR |
| 970017 | Flat BAF |
| 980035 | Flat BAF |
| 980251 | Flat BAF |
| 980319 | Flat BAF |
| 980327 | Flat BAF |
| 980344 | Excessively variable LRR |
| 980386 | Flat BAF |
| 980418 | Flat BAF |
| 980436 | Flat BAF |
| 980442 | Flat BAF |
| 990015 | Flat BAF |
| 990024 | Flat BAF |
| 990068 | Excessively variable LRR |
| 990070 | Apparently low tumor content |
| 990089 | Apparently low tumor content |
| 990119 | Flat BAF |
| 990129 | Excessively variable LRR |
| 990136 | Excessively variable LRR |
| 990205 | Excessively variable LRR |
| 990247 | Flat BAF |
| 990413 | Flat BAF |
| 990424 | Flat BAF |
| 2000114 | Flat BAF |
| 2000159 | Flat BAF |
| 2000178 | Flat BAF |
| 2000238 | Flat BAF |
| 2000291 | Flat BAF |
| 2000346 | Flat BAF |
| 2000479 | Flat BAF |
| 2000617 | Excessively variable LRR |
| 2000920 | Excessively variable LRR |
| 2001159 | Flat BAF |
| 2001226 | Excessively variable LRR |
| 2001229 | Flat BAF |
| 2001241 | Excessively variable LRR |
| 37262942 | Excessively variable LRR |
| 43658255 | Excessively variable LRR |
| 65256293 | Flat BAF |

Table S5. LOH and copy number alterations in 74 gastric adenocarcinomas.

| Sample ID | Average ploidy | Tumor content | Proportion of probes with LOH | CN loss | CN gain |
|---|---|---|---|---|---|
| 970005 | 2.57 | 0.58 | 0.58 | 0.11 | 0.00 |
| 980011 | 1.84 | 0.32 | 0.37 | 0.32 | 0.00 |
| 980021 | 2.81 | 0.77 | 0.33 | 0.00 | 0.03 |
| 980029 | 2.31 | 0.57 | 0.47 | 0.02 | 0.09 |
| 980097 | 2.09 | 0.54 | 0.02 | 0.00 | 0.00 |
| 980156 | 4.18 | 1 | 0.36 | 0.24 | 0.10 |
| 980369 | 4.08 | 0.25 | 0.11 | 0.01 | 0.00 |
| 980390 | 3.05 | 0.76 | 0.40 | 0.25 | 0.01 |
| 980401 | 2.08 | 0.32 | 0.15 | 0.00 | 0.02 |
| 980417 | 1.64 | 0.35 | 0.30 | 0.17 | 0.01 |
| 980437 | 4.19 | 0.6 | 0.00 | 0.01 | 0.00 |
| 980447 | 4.23 | 0.37 | 0.22 | 0.02 | 0.02 |
| 990005 | 1.97 | 0.39 | 0.08 | 0.03 | 0.00 |
| 990010 | 2.33 | 0.34 | 0.31 | 0.00 | 0.14 |
| 990041 | 2 | 0.27 | 0.22 | 0.07 | 0.00 |
| 990044 | 2.65 | 0.45 | 0.37 | 0.05 | 0.03 |
| 990046 | 3.91 | 0.36 | 0.15 | 0.16 | 0.01 |
| 990060 | 2.31 | 0.5 | 0.04 | 0.02 | 0.06 |
| 990069 | 2.98 | 1 | 0.47 | 0.35 | 0.02 |
| 990071 | 2.2 | 0.75 | 0.17 | 0.09 | 0.04 |
| 990090 | 3.41 | 0.48 | 0.23 | 0.31 | 0.07 |
| 990097 | 2.19 | 0.27 | 0.02 | 0.00 | 0.10 |
| 990098 | 3.09 | 0.88 | 0.33 | 0.37 | 0.02 |
| 990108 | 4.26 | 0.2 | 0.29 | 0.09 | 0.01 |
| 990111 | 2.47 | 1 | 0.23 | 0.00 | 0.06 |
| 990170 | 2 | 0.32 | 0.08 | 0.00 | 0.00 |
| 990172 | 2.94 | 0.45 | 0.35 | 0.50 | 0.06 |
| 990195 | 1.64 | 0.42 | 0.43 | 0.40 | 0.02 |
| 990203 | 1.81 | 0.48 | 0.37 | 0.27 | 0.03 |
| 990228 | 2.2 | 0.36 | 0.06 | 0.05 | 0.04 |
| 990275 | 3.56 | 0.53 | 0.34 | 0.11 | 0.10 |
| 990300 | 3.83 | 0.81 | 0.00 | 0.01 | 0.00 |
| 990339 | 2 | 0.29 | 0.15 | 0.06 | 0.00 |
| 990355 | 2 | 0.35 | 0.16 | 0.08 | 0.01 |
| 990396 | 2.14 | 0.41 | 0.51 | 0.15 | 0.09 |
| 990412 | 2.32 | 0.63 | 0.53 | 0.07 | 0.07 |
| 990474 | 2.3 | 0.18 | 0.00 | 0.00 | 0.08 |
| 990475 | 1.96 | 0.67 | 0.15 | 0.14 | 0.04 |
| 990489 | 2.01 | 0.25 | 0.07 | 0.02 | 0.00 |
| 990515 | 4.34 | 0.98 | 0.31 | 0.43 | 0.08 |
| 2000040 | 2 | 0.4 | 0.19 | 0.03 | 0.00 |
| 2000068 | 4.04 | 0.42 | 0.43 | 0.08 | 0.02 |
| 2000085 | 2 | 0.28 | 0.03 | 0.00 | 0.00 |
| 2000088 | 3.61 | 0.62 | 0.00 | 0.03 | 0.03 |
| 2000169 | 4.41 | 0.79 | 0.01 | 0.00 | 0.04 |
| 2000175 | 2.16 | 0.48 | 0.44 | 0.19 | 0.11 |

**Table S5. LOH and copy number alterations in 74 gastric adenocarcinomas.**

| Sample ID | Average ploidy | Tumor content | Proportion of probes with LOH | CN loss | CN gain |
|---|---|---|---|---|---|
| 2000201 | 4.06 | 0.39 | 0.18 | 0.10 | 0.03 |
| 2000242 | 2 | 0.23 | 0.14 | 0.00 | 0.00 |
| 2000286 | 2 | 0.22 | 0.08 | 0.00 | 0.00 |
| 2000303 | 3.87 | 0.54 | 0.00 | 0.02 | 0.01 |
| 2000362 | 4.07 | 0.42 | 0.25 | 0.04 | 0.01 |
| 2000403 | 3.67 | 0.25 | 0.44 | 0.21 | 0.01 |
| 2000433 | 1.73 | 0.35 | 0.38 | 0.31 | 0.02 |
| 2000441 | 3.98 | 0.93 | 0.00 | 0.01 | 0.01 |
| 2000877 | 2.79 | 1 | 0.35 | 0.02 | 0.07 |
| 2000892 | 1.94 | 0.77 | 0.25 | 0.17 | 0.02 |
| 20020011 | 3.93 | 0.35 | 0.00 | 0.03 | 0.00 |
| 20020448 | 4.57 | 0.69 | 0.01 | 0.12 | 0.02 |
| 20020720 | 2.01 | 1 | 0.56 | 0.33 | 0.09 |
| 20263644 | 2 | 0.26 | 0.03 | 0.00 | 0.00 |
| 32226415 | 2 | 0.25 | 0.02 | 0.00 | 0.00 |
| 38877042 | 1.88 | 0.47 | 0.27 | 0.19 | 0.01 |
| 46404174 | 2 | 0.28 | 0.04 | 0.00 | 0.00 |
| 47492137 | 4 | 0.48 | 0.00 | 0.01 | 0.01 |
| 57689477 | 2.9 | 0.83 | 0.35 | 0.48 | 0.10 |
| 57701999 | 1.65 | 0.41 | 0.36 | 0.34 | 0.00 |
| 58947266 | 2.77 | 0.25 | 0.78 | 0.00 | 0.02 |
| 61669256 | 2.1 | 0.25 | 0.18 | 0.00 | 0.03 |
| 66811693 | 2.85 | 0.41 | 0.28 | 0.06 | 0.06 |
| 73291145 | 2.96 | 0.41 | 0.21 | 0.41 | 0.07 |
| 76629543 | 3.61 | 0.8 | 0.27 | 0.59 | 0.18 |
| 87622942 | 3.73 | 0.51 | 0.00 | 0.02 | 0.00 |
| 91228050 | 2.03 | 0.26 | 0.02 | 0.00 | 0.01 |
| 96141474 | 4.36 | 0.22 | 0.10 | 0.25 | 0.00 |
| | **Average** | 0.50 | 0.22 | 0.12 | 0.03 |

**NOTES:**

LOH = loss of heterozygosity

CN loss = proportion of probes that show copy number < average ploidy of sample * 0.7

CN gain = proportion of probes that show copy number > average ploidy of the sample * 1.65

**Table S6. STOP genes that are subject to copy-number loss (copy number < 0.7 times the average plody) in each tumor.**

| Sample | Number of STOP genes subject to copy-number loss | STOP genes subject to copy-number loss |
|---|---|---|
| 76629543 | 452 | *ABCD3,ACACB,ACSL1,ACTN1,ACVR2A,ACYP2,ADAMTS6,ADH7,ADORA3,ADRBK2,AGXT2L1,AHRR,AKAP12,AKAP7,ALB,ALDH3A2,ALG6,ALPK1,AMD1,ANGPTL3,ANKRD44,ANKRD55,ANXA10,AP3M2,AP3S1,APAF1,ARG2,ARHGAP11A,ARL6IP6,ASB15,ASB17,ASB5,ATG5,ATP5J,ATP6V1E1,ATRNL1,AUH,AVPR1A,B3GALT1,BAHD1,BAI2,BAZ1A,BCL11A,BCL6,BDP1,BMI1,BPIL2,BPTF,BRAF,BRDT,BRWD1,BST1,BTBD8,BTC,BTG3,C10orf28,C10orf96,C12orf29,C14orf28,C14orf37,C14orf45,C15orf24,C15orf42,C17orf68,C19orf42,C19orf43,C2orf47,C4orf14,C5orf41,C6orf170,C8orf4,C9orf6,C9orf64,CALD1,CAPZA2,CARTPT,CASD1,CASP3,CASP6,CCDC28A,CCDC74A,CCDC74B,CCDC99,CCNB2,CCNG2,CD109,CD302,CD38,CDC123,CDC14A,CDH10,CDH9,CDKL2,CENPH,CGA,CH25H,CHCHD7,CHD1,CHI3L2,CHKA,CHUK,CLCC1,CLTC,CNOT4,COBLL1,COCH,COIL,COPS8,COQ10B,COX10,CPT2,CSF1,CTBS,CTLA4,CTNS,CTSL1,CXCL12,CXCL6,CYP26B1,CYP2C19,CYP4X1,DAAM1,DAP,DCN,DCTD,DDX1,DFFA,DIABLO,DIS3L,DLG2,DNAH10,DNAH5,DNMT3L,DOCK2,DUXA,DYRK2,ECM2,EFCAB6,EGR1,EIF2AK2,EIF4A2,EIF4E2,ELMOD2,ENPP1,ENPP6,EPB41L2,EPHA5,ERAP2,ERI1,ERRFI1,ESR1,FAM175A,FAM81B,FAM84A,FAM8A1,FBXO22,FBXO25,FBXO3,FBXO34,FBXW7,FER,FGF19,FGF2,FGF7,FOS,FOXD2,FRK,FRZB,FSHR,G3BP1,GAB1,GAD1,GCA,GCNT2,GDF9,GEN1,GJA1,GMCL1,GNG5,GPD2,GPR125,GPR158,GPR21,GPR22,GRB14,GRIK1,GTDC1,GTF3C6,HAT1,HCFC2,HDAC1,HERC3,HERC5,HES1,HMGN1,HMHB1,HNRNPH1,HNRNPUL1,HNRPLL,HTN1,HTR7,IBTK,IFNA1,IFNA13,IFNAR1,IFNW1,IFRD1,IFT81,IGF1,IL31RA,IL8,IMPAD1,INPP1,INVS,IQGAP1,IRAK3,IRAK4,ISL2,ITGA6,JAM2,JUB,KCNJ3,KCNN2,KCTD18,KIF20B,KLHDC1,KLHL6,KNG1,LAIR1,LIFR,LIPI,LIPJ,LMBRD2,LMO4,LPXN,LRAT,LRFN5,LRRC32,LRRIQ3,LUZP1,LYRM7,MAFG,MAP2K6,MAP3K1,MAPK10,MARCH6,MARS2,MCFD2,MEGF11,MEIS2,METT5D1,METTL3,METTL7A,MFF,MIA2,MIPOL1,MKX,MMADHC,MPP5,MS4A5,MSH6,MTF1,MTF2,MTFR1,MTHFD1,MTUS1,MUDENG,MYEOV,MYLIP,MYO1B,NAIP,NDRG2,NDUFA5,NF2,NHLRC2,NLRP9,NPY1R,NR0B2,NR2F2,NR3C2,NRIP1,NSUN6,NT5DC3,NTRK2,NUDT12,NUDT5,OAT,OGN,OLFML3,ORC4L,PAPOLG,PDE7A,PDHA2,PDZRN4,PENK,PGAP1,PHACTR4,PIGK,PJA2,PKP4,PLCE1,PLN,PLXNC1,PNRC1,PNRC2,POLR2D,PON2,PPP2CB,PPP3CB,PRDM1,PRDM8,PRKACB,PRKAR1A,PRKCA,PRKD1,PRTG,PTGER2,PTGR1,PTN,PTPLA,PTPRK,PTRH2,PTTG1,PTTG1IP,RAB2B,RAB33B,RABGGTB,RAD51C,RAD51L1,RAG2,RAI14,RARS2,RBP7,RCAN3,REV3L,RLN2,RNF111,RNF133,RNF170,RNF180,RNF44,RNFT1,RSBN1,RTN1,S1PR1,SAMD13,SCN1A,SDCBP,SEC31A,SEC63,SECISBP2,SENP2,SERAC1,SERINC1,SLC12A2,SLC19A3,SLC20A1,SLC20A2,SLC25A18,SLC38A2,SLC41A2,SLC46A2,SMU1,SNAI2,SNRNP48,SNX6,SNX9,SOCS2,SOCS5,SORCS1,SOS2,SOX7,SPATA4,SPG11,SPTLC1,SQRDL,SRFBP1,SSR1,SSX2IP,ST6GALNAC1,ST7L,STAMBPL1,STK17B,STK39,STON1-GTF2A1L,STRADB,STRN3,STXBP4,SUPT7L,SYNJ1,TANK,TARDBP,TAS1R1,TAS2R1,TEK,TEP1,TGFBR1,THAP3,THPO,TIGD2,TLR3,TLR4,TM9SF3,TMEM144,TMEM56,TMEM59,TMPO,TNKS2,TP53,TPTE,TRAF3IP2,TRAPPC10,TRIM24,TSGA14,TTC9C,TTPA,TUSC3,UBE2U,UGT8,UHRF1BP1L,USP32,USP33,USP34,USP45,USP53,VIP,VPS26A,VRK2,WHSC1L1,YIPF1,ZBTB1,ZBTB2,ZBTB25,ZBTB43,ZBTB6,ZNF253,ZNF254,ZNF326,ZNF330,ZNF417,ZNF43,ZNF549,ZNF571,ZNF587,ZNF626,ZNF644,ZNF664,ZNF675,ZNF800,ZNF804A,ZNF85,ZRANB2* |

**Table S6. STOP genes that are subject to copy-number loss (copy number < 0.7 times the average plody) in each tumor.**

| Sample | Number of STOP genes subject to copy-number loss | STOP genes subject to copy-number loss |
|---|---|---|
| 990172 | 361 | *AADACL2,ACACB,ACSL1,ACTN1,ADAMTS6,ADH7,AGTR1,AGXT2L1,ALDH3A2,ALG6,ALPK1,ANGPTL3,ANKRD55,AP3S1,APAF1,ARF4,ARG2,ARHGAP11A,ARIH2,ARL2BP,ARL6,ASB5,ASTE1,ATG3,ATP5J,ATRNL1,ATXN3,AUH,AVPR1A,AXIN1,BACE1,BAHD1,BAI2,BAZ1A,BCL6,BDP1,BPTF,BRWD1,BST1,BTG3,C10orf28,C10orf79,C10orf96,C11orf58,C12orf29,C14orf109,C14orf28,C14orf37,C14orf45,C15orf24,C15orf42,C16orf63,C16orf75,C17orf68,C19orf42,C19orf43,C3orf14,C3orf15,C3orf23,C4orf14,C4orf23,C5orf41,C9orf6,C9orf64,CADM2,CARTPT,CASP3,CASP6,CCDC99,CCNB2,CCNG2,CCRL1,CD109,CD38,CDC42BPB,CDKL2,CDKN1A,CDKN3,CENPH,CEP70,CEP97,CGA,CH25H,CHD1,CHUK,COCH,COPS8,COX10,CP,CPT2,CRMP1,CSTA,CTDSPL,CTNS,CTSL1,CX3CR1,CXCL12,CYP2C19,CYP4X1,DAAM1,DCN,DCTD,DEPDC1,DFFA,DIABLO,DIS3L,DLG2,DNAH10,DNMT3L,DOCK2,DYRK2,ECM2,EGR1,EIF4A2,EIF4E2,ELMOD2,ENPP6,EPHA5,ERAP2,ERRFI1,FAM175A,FAM55A,FAM81B,FAM8A1,FBXO22,FBXO3,FBXO34,FBXW7,FER,FEZ1,FGF2,FGF7,FLNB,FOS,FOXD2,FXYD2,G3BP1,GAB1,GABRB1,GCNT2,GDF9,GNPDA2,GPR125,GPR15,GPR21,GRIK1,GRK4,GSK3B,GTF2H1,GTPBP8,HCFC2,HDAC1,HERC3,HERC5,HES1,HESX1,HMGN1,HMHB1,HNRNPH1,HTN1,HTR1F,HTR7,HYAL2,IBTK,IFNA1,IFNA13,IFNAR1,IFNW1,IFT81,IGF1,IL31RA,INA,INVS,IPP,IQCK,IQGAP1,IRAK3,IRAK4,ISL2,JAM2,JUB,KAT2B,KBTBD3,KCNN2,KIF20B,KLHDC1,KLHL6,KNG1,KPNA4,LEKR1,LIPI,LIPJ,LNX1,LPXN,LRAT,LRFN5,LRRFIP2,LUZP1,LYRM7,MAFG,MAP2K6,MAP3K1,MAPK10,MEGF11,MEIS2,MELK,METT5D1,METTL3,METTL7A,MIA2,MICAL2,MIPOL1,MITF,MLH1,MME,MMP20,MPP5,MS4A5,MTF1,MTHFD1,MUDENG,MYLIP,NAIP,NDRG2,NDUFB4,NHLRC2,NMNAT3,NPY1R,NR0B2,NR1D2,NR3C2,NRIP1,NRIP3,NRSN1,NT5DC3,NTRK2,NUDT12,OAT,OGN,ORC6L,P2RY13,PARP15,PCGF3,PDHA2,PDZRN4,PFN2,PHACTR4,PHF7,PIK3R4,PJA2,PLCE1,PLCL2,PLSCR4,PLXNC1,PNRC1,PNRC2,PPARG,PPAT,PPP1R13B,PPP3CB,PRDM8,PRKAR1A,PRKCA,PRKD1,PRTG,PTGER2,PTGR1,PTP4A1,PTTG1,PTTG1IP,RAB2B,RAB33B,RAD18,RAD51L1,RAF1,RAG2,RARS2,RBP7,RCAN3,RLN2,RNF111,RNF13,RNF180,RNF44,RNF7,RPE65,RSRC1,RTN1,SEC31A,SECISBP2,SENP2,SEPSECS,SKIL,SLC25A31,SLC37A2,SLC38A2,SLC41A2,SLC46A2,SLC6A6,SLCO2A1,SMU1,SNRNP48,SNX6,SOCS2,SORCS1,SOS2,SPATA18,SPATA4,SPG11,SPTLC1,SQRDL,SRD5A3,SRFBP1,SSR1,ST6GALNAC1,STAMBPL1,STRN3,SYNJ1,TARDBP,TAS1R1,TEK,TEP1,TESK2,TGFBR1,THAP3,THPO,THRB,TIGD2,TLR10,TLR3,TLR4,TM9SF3,TMEM144,TMEM165,TMEM59,TMPO,TNKS2,TP53,TPTE,TRAPPC10,TRIM39,TXK,UBE2E1,UBE2K,UBE2U,UGT8,UHRF1BP1L,USP45,USP53,VPS13A,VPS26A,YIPF1,ZBTB1,ZBTB25,ZBTB6,ZBTB9,ZKSCAN2,ZNF330,ZNF35,ZNF664,ZRANB2* |

Table S6. STOP genes that are subject to copy-number loss (copy number < 0.7 times the average plody) in each tumor.

| Sample | Number of STOP genes subject to copy-number loss | STOP genes subject to copy-number loss |
|---|---|---|
| 57689477 | 360 | *ACACB,ACSL1,ACTN1,ACVR2A,ACYP2,ADAMTS6,ADH7,ADRBK2,AFF3,AGXT2L1,AHRR,ALB,ALDH3A2,ALG10,ALPK1,ANKRD44,ANKRD55,ANXA10,AP3S1,APAF1,ARG2,ARHGAP11A,ARL2BP,ARL6IP6,ASB15,ASB5,ATP5J,ATP6V1E1,ATXN3,AUH,AVPR1A,B3GALT1,BAHD1,BAZ1A,BCL11A,BDP1,BPIL2,BPTF,BRAF,BRWD1,BST1,BTC,BTG3,C12orf29,C12orf60,C13orf27,C13orf34,C14orf109,C14orf28,C14orf37,C14orf45,C15orf24,C17orf68,C2orf29,C2orf47,C4orf14,C4orf23,C5orf41,C9orf6,C9orf64,CALD1,CAPZA2,CARTPT,CASP3,CASP6,CCDC74A,CCDC74B,CCDC99,CCL22,CCNG2,CD302,CD38,CDC42BPB,CDH10,CDH9,CDKL2,CDKN1B,CDKN3,CENPH,CHD1,CLEC12A,CLEC2B,CLEC4A,CLTC,CNOT4,COBLL1,COCH,COIL,COPS8,COQ10B,COX10,CPB2,CREBL2,CRMP1,CTLA4,CTNS,CTSL1,CXCL6,CYP26B1,DAAM1,DAP,DCN,DCTD,DDX1,DIABLO,DNAH10,DNAH5,DNMT3L,DOCK2,DYRK2,ECM2,EFCAB6,EGR1,EIF2AK2,EIF4E2,ELMOD2,EMP1,ENPP6,ENTHD1,EP300,EPHA5,ERAP2,ERGIC2,FA2H,FAM175A,FAM81B,FAM84A,FBXL14,FBXL3,FBXO34,FBXW7,FER,FGF2,FGF7,FGFR1OP2,FKBP4,FOS,FOXF1,FRZB,FSHR,G3BP1,GAB1,GABRB1,GAD1,GCA,GCSH,GDF9,GEN1,GIMAP4,GMCL1,GNPDA2,GPC5,GPD2,GPR125,GPR180,GPR21,GPR22,GRB14,GRIK1,GRK4,GTDC1,HAT1,HCFC2,HERC3,HERC5,HMGN1,HMHB1,HNRNPH1,HNRPLL,HS6ST3,HTN1,IFNA1,IFNA13,IFNW1,IFRD1,IFT81,IGF1,IL1R1,IL31RA,IL8,INPP1,INVS,IRAK3,IRAK4,ITGA6,JAM2,JUB,KCNJ3,KCNN2,KCTD18,KLHDC1,KLHL36,KLRB1,KLRF1,KPNA3,KRAS,LIFR,LIG4,LIPT1,LMBRD2,LNX1,LRAT,LRFN5,LYRM7,MAB21L1,MAFG,MAP2K6,MAP3K1,MAPK10,MARCH6,MARS2,MCFD2,MCHR1,MEIS2,MELK,METTL3,METTL7A,MFAP5,MFF,MGP,MIA2,MIPOL1,MMADHC,MPP5,MSH6,MTHFD1,MUDENG,MYCBP2,MYO1B,NAE1,NAIP,NDRG2,NDUFA5,NF2,NPY1R,NR3C2,NT5DC3,NTRK2,NUDT12,OGN,OLFM4,ORC4L,ORC6L,PACSIN2,PAPOLG,PCDH9,PCGF3,PDHA2,PDZRN4,PGAP1,PJA2,PKP4,PLXNC1,POLR2D,PPAT,PPFIBP1,PPP1R13B,PRDM8,PRKAR1A,PRKCA,PRKD1,PRTG,PTGER2,PTGR1,PTN,PTRH2,PTTG1,PTTG1IP,RAB2B,RAB33B,RAD51C,RAD51L1,RAI14,RAP2A,RB1,RCBTB2,RLN2,RNF133,RNF180,RNF44,RNFT1,RTN1,SCN1A,SEC31A,SECISBP2,SEPSECS,SLC12A2,SLC19A3,SLC20A1,SLC25A17,SLC25A18,SLC25A31,SLC38A2,SLC41A2,SLC46A2,SMU1,SNX6,SOCS2,SOCS5,SOS2,SOX1,SPATA18,SPATA4,SPG11,SPTLC1,SQRDL,SRD5A3,SRFBP1,ST6GALNAC1,STK17B,STK39,STON1-GTF2A1L,STRADB,STRAP,STRN3,STXBP4,SUPT7L,SYNJ1,TANK,TAS2R1,TBC1D4,TEK,TEP1,TGFBR1,TIGD2,TLR10,TLR3,TLR4,TMEM144,TMEM165,TMPO,TP53,TRAPPC10,TRIM24,TSGA14,TXK,UBE2K,UCHL3,UGT8,UHRF1BP1L,USP32,USP34,USP53,VAPA,VPS13A,VRK2,ZBTB1,ZBTB25,ZBTB43,ZBTB6,ZFP1,ZIC2,ZNF254,ZNF330,ZNF43,ZNF664,ZNF675,ZNF800,ZNF804A,ZNF85* |

**Table S6. STOP genes that are subject to copy-number loss (copy number < 0.7 times the average plody) in each tumor.**

| Sample | Number of STOP genes subject to copy-number loss | STOP genes subject to copy-number loss |
|---|---|---|
| 990515 | 328 | *AADACL2,ABCD3,ACSL1,ACTN1,ADAMTS6,ADH7,ADNP2,ADRBK2,AGTR1,AGXT2L1,AKAP12,ALB,ALG6,ALPK1,ANGPTL3,ANKRD55,ANXA10,AP3S1,ARF4,ARG2,ARIH2,ARL6,ASB15,ASB17,ASB5,ASTE1,ATG3,ATP5J,ATP6V1E1,ATRNL1,ATXN3,AXIN1,B3GALT1,BAI2,BCL6,BDP1,BPIL2,BRAF,BRDT,BRWD1,BST1,BTBD8,BTC,BTG3,C10orf28,C10orf79,C10orf96,C14orf109,C14orf45,C16orf63,C16orf75,C19orf42,C19orf43,C3orf14,C3orf15,C3orf23,C4orf23,C5orf41,C9orf64,CADM2,CALD1,CARTPT,CASP3,CASP6,CCDC99,CCNG2,CCRL1,CD38,CDC42BPB,CDKL2,CDKN1B,CENPH,CEP70,CEP97,CH25H,CHD1,CHUK,CLEC12A,CLEC2B,CLEC4A,CNOT4,COPS8,CP,CPT2,CREBL2,CRMP1,CSTA,CTBS,CTDSPL,CTSL1,CX3CR1,CXCL12,CXCL6,CYP2C19,CYP4X1,DCTD,DEPDC1,DFFA,DNMT3L,DOCK2,EFCAB6,EGR1,EIF4A2,EIF4E2,ELMOD2,ENPP6,ENTHD1,EP300,EPHA5,ERAP2,ERI1,ERRFI1,ESR1,FAM175A,FAM81B,FBXL14,FBXO15,FBXO25,FBXW7,FER,FGF2,FKBP4,FLNB,FOS,FOXD2,G3BP1,GAB1,GDF9,GIMAP4,GNG5,GPR125,GPR15,GRIK1,GRK4,GSK3B,GTPBP8,HDAC1,HERC3,HERC5,HES1,HESX1,HMGN1,HMHB1,HNRNPH1,HTR1F,HTR7,HYAL2,IFNA1,IFNA13,IFNAR1,IFNW1,IL31RA,IL8,INA,IPP,IQCK,IRAK4,JAM2,KAT2B,KCNN2,KDSR,KIF20B,KLHL6,KLRB1,KLRF1,KNG1,KPNA4,LEKR1,LIPI,LIPJ,LMO4,LPXN,LRAT,LRRFIP2,LRRIQ3,LUZP1,LYRM7,MAP3K1,MAPK10,MCHR1,MELK,METTL7A,MFAP5,MFF,MITF,MLH1,MME,MPP5,MS4A5,MTF1,MTF2,MTHFD1,MTUS1,NAIP,NDUFA5,NDUFB4,NF2,NHLRC2,NMNAT3,NPY1R,NR0B2,NR1D2,NR3C2,NRIP1,NTRK2,NUDT12,OAT,P2RY13,PACSIN2,PARP15,PCGF3,PDHA2,PDZRN4,PFN2,PHACTR4,PHF7,PIGK,PIK3C3,PIK3R4,PJA2,PLCE1,PLCL2,PLSCR4,PMAIP1,PNRC2,PPARG,PPP1R13B,PPP2CB,PPP3CB,PRDM8,PRKACB,PTN,PTTG1,PTTG1IP,RAB33B,RABGGTB,RAD18,RAD51L1,RAF1,RBP7,RCAN3,RLN2,RNF13,RNF133,RNF180,RNF44,RNF7,ROCK1,RPE65,RSRC1,SAMD13,SCN1A,SEC31A,SENP2,SEPSECS,SERAC1,SERPINB7,SKIL,SLC12A2,SLC19A3,SLC25A17,SLC25A18,SLC25A31,SLC38A2,SLC6A6,SLCO2A1,SMAD2,SMAD4,SMU1,SNX9,SORCS1,SOX7,SPATA4,SRFBP1,SSX2IP,ST8SIA5,STAMBPL1,STK39,SYNJ1,TARDBP,TAS1R1,TEK,TESK2,THAP3,THPO,THRB,TIGD2,TLR10,TLR3,TM9SF3,TMEM144,TMEM56,TMEM59,TNKS2,TPTE,TRAPPC10,TRIM24,TSGA14,TTC9C,TUSC3,UBE2E1,UBE2K,UBE2U,UGT8,USP33,USP45,USP53,VIP,VPS13A,VPS26A,WHSC1L1,YIPF1,ZBTB1,ZBTB2,ZBTB25,ZKSCAN2,ZNF253,ZNF254,ZNF326,ZNF330,ZNF35,ZNF43,ZNF626,ZNF644,ZNF675,ZNF800,ZNF85,ZRANB2* |
| 73291145 | 305 | *ABCB1,ACSL1,ACTN1,ADAMTS6,ADH7,ADNP2,ADRBK2,AGXT2L1,AHRR,AIG1,ALB,ALPK1,ANKRD55,ANXA10,AP3M2,AP3S1,ARF4,ARG2,ARHGAP11A,ARIH2,ASB5,ATP12A,ATP5J,ATP6V1E1,BAHD1,BAZ1A,BDP1,BPIL2,BPTF,BRWD1,BST1,BTC,BTG3,C13orf27,C13orf34,C14orf28,C14orf37,C14orf45,C15orf24,C15orf42,C19orf42,C19orf43,C3orf14,C3orf23,C4orf14,C4orf23,C5orf41,C8orf4,CADM2,CARTPT,CASD1,CASP3,CASP6,CCDC99,CCNB2,CCNG2,CD38,CDH10,CDH2,CDH9,CDKL2,CDKN3,CENPH,CHCHD7,CHD1,CLTC,COCH,COIL,CPB2,CRMP1,CROT,CTDSPL,CX3CR1,CXCL6,DAAM1,DAP,DCTD,DIS3L,DNAH5,DNMT3L,DOCK2,EFCAB6,EGR1,ELMOD2,ENPP6,ENTHD1,EP300,EPHA5,ERAP2,ERI1,ESCO1,FA2H,FAM175A,FAM81B,FBXL3,FBXO15,FBXO22,FBXO25,FBXO34,FBXW7,FER,FGF2,FGF7,FGL2,FLNB,FOS,FOXF1,G3BP1,GAB1,GABRB1,GCSH,GDF9,GNPDA2,GPC5,GPR125,GPR180,GRIK1,GRK4,HERC3,HERC5,HESX1,HMGN1,HMHB1,HNRNPH1,HS6ST3,HTN1,HTR1F,HYAL2,IFNA1,IFNA13,IFNAR1,IFNW1,IL31RA,IL8,IMPAD1,IQGAP1,ISL2,JAM2,JUB,KAT2B,KCNN2,KDSR,KIAA1324L,KLHDC1,KLHL36,KPNA3,LIFR,LIG4,LIPI,LMBRD2,LNX1,LRAT,LRFN5,LRRFIP2,LYRM7,MAFG,MAP2K6,MAP3K1,MAPK10,MARCH6,MCHR1,MEGF11,MEIS2,MELK,METTL3,MIA2,MIPOL1,MITF,MLH1,MPP5,MTFR1,MTHFD1,MTUS1,MUDENG,MYCBP2,NAE1,NAIP,NDRG2,NF2,NPY1R,NR1D2,NR2F2,NR3C2,NRIP1,NUDT12,OLFM4,PACSIN2,PCDH9,PCGF3,PDE7A,PDHA2,PENK,PHF7,PIK3C3,PJA2,PLCL2,PMAIP1,PON2,PPARG,PPAT,PPP2CB,PRDM8,PRKAR1A,PRKCA,PRKD1,PRTG,PTGER2,PTRH2,PTTG1,PTTG1IP,RAB2B,RAB33B,RAD18,RAD51C,RAD51L1,RAF1,RAI14,RAP2A,RB1,RCBTB2,RIOK3,RLN2,RNF111,RNF125,RNF138,RNF170,RNF180,RNF44,RNFT1,ROCK1,RSBN1L,RTN1,SACS,SDCBP,SEC31A,SEPSECS,SERPINB7,SLC12A2,SLC20A2,SLC25A17,SLC25A18,SLC25A31,SLC6A6,SMAD2,SMAD4,SMU1,SNAI2,SNX6,SOS2,SOX1,SOX7,SPATA18,SPATA4,SPG11,SQRDL,SRD5A3,SRFBP1,ST6GALNAC1,ST8SIA5,STOML3,STRN3,STXBP4,SUGT1,SYNJ1,TAS2R1,TBC1D4,TEK,TEP1,THRB,TIGD2,TLR10,TLR3,TMEM144,TMEM165,TMEM60,TPT1,TPTE,TPTE2,TRAM1,TRAPPC10,TTPA,TUSC3,TXK,UBE2E1,UBE2K,UCHL3,UGT8,USP12,USP32,USP53,UTRN,VAPA,WHSC1L1,ZBTB1,ZBTB25,ZFP1,ZIC2,ZNF253,ZNF254,ZNF330,ZNF35,ZNF43,ZNF626,ZNF675,ZNF85* |

Table S6. STOP genes that are subject to copy-number loss (copy number < 0.7 times the average plody) in each tumor.

| Sample | Number of STOP genes subject to copy-number loss | STOP genes subject to copy-number loss |
|---|---|---|
| 990195 | 292 | *ACSL1,ACTN1,ACYP2,ADAMTS6,ADH7,ADNP2,ADRBK2,AGXT2L1,AHRR,ALB,ALPK1,ANKRD55,ANXA10,AP3S1,ARG2,ARL2BP,ASB5,ATP5J,ATP6V1E1,ATRNL1,ATXN3,AUH,AXIN1,BAZ1A,BCL11A,BDP1,BMI1,BPIL2,BRWD1,BTC,BTG3,C10orf28,C10orf79,C10orf96,C11orf58,C14orf109,C14orf28,C14orf37,C14orf45,C16orf63,C16orf75,C19orf42,C19orf43,C4orf14,C4orf23,C5orf41,C9orf6,C9orf64,CARTPT,CASP3,CASP6,CCDC99,CCL22,CCNG2,CDC123,CDC42BPB,CDH10,CDH2,CDH9,CDKL2,CDKN1B,CDKN3,CENPH,CH25H,CHD1,CHUK,CLEC12A,CLEC2B,CLEC4A,COCH,CREBL2,CRMP1,CTSL1,CXCL12,CXCL6,CYP2C19,DAAM1,DAP,DCTD,DNAH5,DNMT3L,DOCK2,DUSP8,DUXA,ECM2,EFCAB6,EGR1,EIF2AK2,ELMOD2,EMP1,ENPP6,ENTHD1,EP300,EPHA5,ERAP2,ERI1,FA2H,FAM175A,FAM81B,FBXL14,FBXO15,FBXO25,FBXO34,FBXW7,FER,FGF2,FKBP4,FOS,FOXF1,FSHR,G3BP1,GAB1,GABRB1,GCSH,GDF9,GNPDA2,GPR125,GPR158,GPR21,GRIK1,GRK4,GTF2H1,HERC3,HERC5,HMGN1,HMHB1,HNRNPH1,HNRNPUL1,HNRPLL,HTN1,HTR7,IFNA1,IFNA13,IFNAR1,IFNW1,IL31RA,IL8,INA,INVS,IQCK,JAM2,JUB,KCNN2,KDSR,KIF20B,KLHDC1,KLHL36,KLRB1,KLRF1,LAIR1,LIFR,LIPI,LIPJ,LMBRD2,LNX1,LRAT,LRFN5,LYRM7,MAFG,MAP3K1,MAPK10,MARCH6,MCFD2,MCHR1,MELK,METTL3,MFAP5,MIA2,MICAL2,MIPOL1,MKX,MPP5,MSH6,MTHFD1,MUDENG,NAE1,NAIP,NDRG2,NF2,NHLRC2,NLRP9,NPY1R,NR3C2,NRIP1,NRIP3,NSUN6,NTRK2,NUDT12,NUDT5,OAT,OGN,OLFML1,ORC6L,PACSIN2,PAPOLG,PDHA2,PIK3C3,PJA2,PLCE1,PMAIP1,PPAT,PPP1R13B,PPP3CB,PRDM8,PRKD1,PTGER2,PTGR1,PTPLA,PTTG1,PTTG1IP,RAB2B,RAB33B,RAD51L1,RAI14,RIOK3,RLN2,RNF125,RNF138,RNF180,RNF44,RTN1,SEC31A,SECISBP2,SEPSECS,SERPINB7,SLC12A2,SLC25A17,SLC25A18,SLC25A31,SLC46A2,SMAD2,SMAD4,SMU1,SNRNP48,SNX6,SOCS5,SORCS1,SOS2,SOX7,SPATA18,SPATA4,SPTLC1,SRD5A3,SRFBP1,SSR1,ST6GALNAC1,ST8SIA5,STAMBPL1,STON1-GTF2A1L,STRN3,SUPT7L,SYNJ1,TAS2R1,TEK,TEP1,TGFBR1,TIGD2,TLR10,TLR3,TLR4,TM9SF3,TMEM144,TMEM165,TNKS2,TPTE,TRAPPC10,TXK,UBE2K,UBQLNL,UGT8,USP34,USP53,VPS13A,VPS26A,VRK2,ZBTB1,ZBTB25,ZBTB43,ZBTB6,ZFP1,ZKSCAN2,ZNF214,ZNF253,ZNF254,ZNF330,ZNF417,ZNF43,ZNF549,ZNF587,ZNF626,ZNF675,ZNF85* |
| 990098 | 277 | *ACTN1,ADCY10,ADH7,AGXT2L1,AIG1,AKAP12,AKAP7,ALB,ALPK1,AMD1,ANXA10,ARF4,ARG2,ARHGAP11A,ARIH2,ARL2BP,ARL6,ASB5,ATG5,ATP5J,ATXN3,AUH,BAZ1A,BMI1,BRWD1,BST1,BTC,BTG3,C12orf60,C14orf109,C14orf28,C14orf37,C14orf45,C16orf63,C16orf75,C1orf49,C3orf14,C3orf23,C4orf14,C4orf23,C6orf170,C9orf6,C9orf64,CADM2,CASP6,CCDC28A,CCL22,CCNG2,CD109,CD1D,CD2AP,CD38,CD55,CDC123,CDC42BPB,CDH2,CDKL2,CDKN1A,CDKN1B,CDKN3,CEP97,CGA,CLEC12A,CLEC2B,CLEC4A,COCH,CREBL2,CTSK,CTSL1,CXCL6,DAAM1,DCTD,DISP1,ECM2,ELMOD2,EMP1,ENPP1,ENPP5,EPB41L2,EPHA5,ERI1,ESCO1,ESR1,FA2H,FAM175A,FAM177B,FAM8A1,FBXL14,FBXO15,FBXO25,FBXO28,FBXO34,FBXW7,FCGR3A,FCGR3B,FCRL2,FGF2,FKBP4,FLNB,FMO5,FOS,FOXF1,FRK,GAB1,GABRB1,GCNT2,GCSH,GJA1,GNPDA2,GPR110,GPR125,GPR15,GRIK1,GSTA3,GTF3C6,HERC3,HERC5,HESX1,HMGN1,HORMAD1,HTN1,HTR1F,HYAL2,IBTK,IER5,IFNA1,IFNA13,IFNAR1,IFNW1,IL8,INVS,IQCK,JAM2,JUB,KCNK1,KDSR,KIF6,KIFAP3,KLHDC1,KLHL36,KLRB1,KLRF1,LEMD1,LIPI,LNX1,LRAT,LRFN5,LYST,MAPK10,METTL3,MFAP5,MGP,MIA2,MIPOL1,MITF,MPP5,MTHFD1,MTUS1,MUDENG,MYB,MYLIP,NAE1,NDRG2,NPY1R,NR1D2,NR3C2,NR5A2,NRIP1,NRSN1,NSUN6,NTRK2,NUDT5,OGN,OPN3,ORC6L,PDHA2,PHF7,PIK3C3,PLN,PMAIP1,PNRC1,PPAT,PPP1R13B,PRDM1,PRDM8,PRKD1,PSEN2,PTGER2,PTGR1,PTP4A1,PTPLA,PTPRK,RAB2B,RAB33B,RAD51L1,RARS2,REV3L,RGS2,RGS5,RHOU,RIOK3,RLN2,RNF125,RNF138,ROCK1,RTN1,RUSC1,SCYL3,SEC31A,SEC63,SECISBP2,SEPSECS,SERAC1,SERINC1,SERPINB7,SERPINC1,SLC19A2,SLC25A31,SLC46A2,SMAD2,SMAD4,SNRNP48,SNX10,SNX6,SNX9,SOS2,SOX7,SPATA18,SPATA4,SPTLC1,SRD5A3,SSR1,ST8SIA5,STRAP,STRN3,SYNJ1,SYT14,TAF1A,TEK,TEP1,TGFBR1,THRB,TIGD2,TLR10,TLR4,TMEM144,TMEM165,TNFSF4,TOR1AIP1,TPTE,TRAF3IP2,TRIM39,TRIM58,TUSC3,TXK,UBE2E1,UBE2K,UBR2,UGT8,URB2,USP45,USP53,UTRN,VAPA,VIP,VPS13A,YOD1,ZBTB1,ZBTB2,ZBTB25,ZBTB9,ZFP1,ZKSCAN2,ZNF281,ZNF330,ZNF35,ZNRF2* |

**Table S6. STOP genes that are subject to copy-number loss (copy number < 0.7 times the average plody) in each tumor.**

| Sample | Number of STOP genes subject to copy-number loss | STOP genes subject to copy-number loss |
|---|---|---|
| 20020720 | 276 | *AADACL2,ABCB1,ABCD3,ACSL1,ACTN1,ADAMTS6,ADH7,ADORA3,AGTR1,AGXT2L1,ALB,ALG6,ALPK1,ANGPTL3,ANKRD55,ANXA10,AP3S1,ARF4,ARG2,ARIH2,ARL6,ASB15,ASB17,ASB5,ASTE1,ATG3,ATP5J,ATXN3,BAHD1,BAI2,BAZ1A,BCL6,BDP1,BRAF,BRDT,BRWD1,BTBD8,BTC,BTG3,C14orf109,C14orf28,C14orf37,C14orf45,C3orf14,C3orf15,C3orf23,C4orf14,C5orf41,CADM2,CALD1,CAPZA2,CARTPT,CASD1,CASP3,CASP6,CCDC99,CCNB2,CCNG2,CCRL1,CD2AP,CDC14A,CDC42BPB,CDKL2,CDKN3,CENPH,CEP70,CEP97,CHD1,CHI3L2,CLCC1,CNOT4,COCH,CP,CPT2,CROT,CSF1,CSTA,CTBS,CTDSPL,CX3CR1,CXCL6,CYP4X1,DAAM1,DCTD,DEPDC1,DFFA,DIABLO,DNMT3L,DOCK2,EGR1,EIF4A2,ELMOD2,ENPP5,ENPP6,EPHA5,ERAP2,ERRFI1,FAM175A,FAM81B,FAM8A1,FBXO34,FBXW7,FER,FGF2,FGF7,FLNB,FOS,FOXD2,G3BP1,GAB1,GDF9,GIMAP4,GNG5,GPR110,GPR15,GPR22,GRIK1,GSK3B,GSTA3,GTPBP8,HDAC1,HERC3,HERC5,HES1,HESX1,HMGN1,HMHB1,HNRNPH1,HTN1,HTR1F,HYAL2,IFNA1,IFNA13,IFNAR1,IFNW1,IFRD1,IL31RA,IL8,IPP,JAM2,KAT2B,KCNN2,KIAA1324L,KLHDC1,KLHL6,KNG1,KPNA4,LEKR1,LIPI,LMO4,LRAT,LRFN5,LRRFIP2,LRRIQ3,LUZP1,LYRM7,MAP3K1,MAPK10,MELK,MIA2,MIPOL1,MITF,MLH1,MME,MPP5,MTF1,MTF2,MTHFD1,MUDENG,MYLIP,NAIP,NDUFA5,NDUFB4,NMNAT3,NPY1R,NR0B2,NR1D2,NR3C2,NRIP1,NRSN1,NUDT12,OLFML3,P2RY13,PARP15,PDHA2,PFN2,PHACTR4,PHF7,PIGK,PIK3R4,PJA2,PLCL2,PLSCR4,PNRC2,PON2,PPARG,PPP1R13B,PRDM8,PRKACB,PRKD1,PRTG,PTGER2,PTN,PTTG1,PTTG1IP,RAB33B,RABGGTB,RAD18,RAD51L1,RAF1,RBP7,RCAN3,RLN2,RNF111,RNF13,RNF133,RNF180,RNF44,RNF7,RPE65,RSBN1,RSRC1,RTN1,S1PR1,SAMD13,SEC31A,SENP2,SKIL,SLC12A2,SLC25A31,SLC6A6,SLCO2A1,SMU1,SNX6,SOS2,SPATA4,SPG11,SQRDL,SRFBP1,SSX2IP,ST7L,STRN3,SYNJ1,TARDBP,TAS1R1,TEK,TESK2,THAP3,THPO,THRB,TIGD2,TLR3,TMEM144,TMEM56,TMEM59,TPTE,TRAPPC10,TRIM24,TRIM39,TSGA14,UBE2E1,UBE2U,UBR2,UGT8,USP33,USP53,VAPA,YIPF1,ZBTB1,ZBTB25,ZNF326,ZNF330,ZNF35,ZNF644,ZNF800,ZRANB2* |
| 980011 | 271 | *ABCD3,ACSL1,ACVR2A,ADH7,ADNP2,ADORA3,AGXT2L1,AIG1,AKAP12,AKAP7,ALB,ALDH3A2,ALG6,ALPK1,AMD1,ANGPTL3,ANXA10,ARF4,ARIH2,ARL6IP6,ASB17,ASB5,ATG5,ATP5J,AUH,B3GALT1,BAI2,BAZ1A,BRDT,BRWD1,BST1,BTBD8,BTC,BTG3,C17orf68,C2orf47,C3orf14,C3orf23,C4orf14,C4orf23,C6orf170,C9orf6,C9orf64,CASP3,CASP6,CCDC28A,CCDC74A,CCDC74B,CCNG2,CD109,CD2AP,CD302,CD38,CDC14A,CDKL2,CDKN1A,CGA,CHI3L2,CLCC1,COBLL1,COCH,COPS8,COX10,CPT2,CRMP1,CSF1,CTBS,CTDSPL,CTLA4,CTNS,CTSL1,CX3CR1,CXCL6,CYP4X1,DCTD,DDX1,DEPDC1,DFFA,ECM2,EIF4E2,ELMOD2,ENPP1,ENPP5,ENPP6,EPB41L2,EPHA5,ERRFI1,ESR1,FAM175A,FAM84A,FAM8A1,FBXO15,FBXW7,FGF2,FLNB,FOXD2,FRK,GAB1,GABRB1,GAD1,GCA,GCNT2,GEN1,GJA1,GNG5,GNPDA2,GPD2,GPR110,GPR125,GRB14,GRIK1,GRK4,GSTA3,GTDC1,GTF3C6,HAT1,HDAC1,HERC3,HERC5,HESX1,HMGN1,HTN1,HYAL2,IBTK,IFNA1,IFNA13,IFNAR1,IFNW1,IL8,INVS,IPP,ITGA6,JAM2,JUB,KAT2B,KCNJ3,KCTD18,KDSR,KIF6,LIPI,LMO4,LNX1,LRAT,LRFN5,LRRFIP2,LRRIQ3,LUZP1,MAPK10,METTL3,MIA2,MIPOL1,MLH1,MMADHC,MTF1,MTF2,MYB,MYLIP,NDRG2,NPY1R,NR0B2,NR1D2,NR3C2,NRIP1,NRSN1,NTRK2,OGN,OLFML3,ORC4L,PCGF3,PDHA2,PHACTR4,PHF7,PIGK,PIK3C3,PKP4,PLCL2,PLN,PMAIP1,PNRC1,PNRC2,POLR2D,PPARG,PPAT,PRDM1,PRDM8,PRKACB,PRKD1,PTP4A1,PTPRK,RAB2B,RAB33B,RABGGTB,RAD18,RAF1,RARS2,RBP7,RCAN3,REV3L,RLN2,RPE65,RSBN1,S1PR1,SAMD13,SCN1A,SEC31A,SEC63,SECISBP2,SEPSECS,SERAC1,SERINC1,SERPINB7,SLC25A31,SLC6A6,SMAD2,SMAD4,SNRNP48,SNX6,SNX9,SPATA18,SPATA4,SPTLC1,SRD5A3,SSR1,SSX2IP,ST7L,ST8SIA5,STK39,STRADB,STRN3,SUPT7L,SYNJ1,TANK,TARDBP,TAS1R1,TEK,TEP1,TESK2,TGFBR1,THAP3,THRB,TIGD2,TLR10,TLR3,TMEM144,TMEM165,TMEM56,TMEM59,TP53,TPTE,TRAF3IP2,TRIM39,TXK,UBE2E1,UBE2K,UBE2U,UBR2,UGT8,USP33,USP45,USP53,UTRN,VIP,VPS13A,YIPF1,ZBTB2,ZBTB9,ZNF326,ZNF330,ZNF35,ZNF644,ZRANB2* |

**Table S6. STOP genes that are subject to copy-number loss (copy number < 0.7 times the average plody) in each tumor.**

| Sample | Number of STOP genes subject to copy-number loss | STOP genes subject to copy-number loss |
|---|---|---|
| 990069 | 271 | *ACSL1,ACTN1,ADAMTS6,ADH7,ADNP2,ADRBK2,AGXT2L1,ALB,ALDH3A2,ALPK1,ANKRD55,ANXA10,AP3S1,ARG2,ARHGAP11A,ARIH2,ARL2BP,ASB5,ATP6V1E1,ATRNL1,ATXN3,AXIN1,BAHD1,BAZ1A,BDP1,BPIL2,BPTF,BST1,BTC,C10orf28,C10orf79,C10orf96,C14orf109,C14orf28,C14orf37,C14orf45,C15orf24,C15orf42,C16orf63,C16orf75,C17orf68,C19orf42,C19orf43,C3orf23,C4orf14,C4orf23,C5orf41,CARTPT,CASP3,CASP6,CCDC99,CCL22,CCL7,CCNB2,CCNG2,CD2AP,CD38,CDH2,CDKL2,CDKN1A,CDKN3,CENPH,CH25H,CHD1,CHUK,CLTC,COCH,COIL,COX10,CPB2,CPD,CRMP1,CTDSPL,CTNS,CX3CR1,CXCL6,CYP2C19,DAAM1,DCTD,DIS3L,DOCK2,EFCAB6,EGR1,ELMOD2,ENPP5,ENPP6,ENTHD1,EP300,EPHA5,ERAP2,ERI1,FA2H,FAM175A,FAM81B,FBXO15,FBXO22,FBXO25,FBXO34,FBXW7,FER,FGF2,FGF7,FOS,FOXF1,G3BP1,GAB1,GABRB1,GCSH,GDF9,GNPDA2,GPR110,GPR125,GRK4,HERC3,HERC5,HMHB1,HNRNPH1,HTN1,HTR7,HYAL2,IL31RA,IL8,INA,IQCK,IQGAP1,ISL2,JUB,KAT2B,KCNN2,KDSR,KIF20B,KIF6,KLHDC1,KLHL36,KPNA3,LIPJ,LNX1,LRAT,LRFN5,LRRFIP2,LYRM7,MAFG,MAP2K6,MAP3K1,MAPK10,MCHR1,MEGF11,MEIS2,METTL3,MIA2,MIPOL1,MLH1,MPP5,MTHFD1,MTUS1,MUDENG,NAE1,NAIP,NDRG2,NF2,NHLRC2,NPY1R,NR1D2,NR2F2,NR3C2,NUDT12,OAT,OLFM4,OMG,ORC6L,PACSIN2,PCDH9,PCGF3,PDHA2,PHF7,PIGW,PIK3C3,PJA2,PLCE1,PLCL2,PMAIP1,PPARG,PPAT,PPP2CB,PRDM8,PRKAR1A,PRKCA,PRKD1,PRTG,PTGER2,PTRH2,PTTG1,RAB2B,RAB33B,RAD18,RAD51C,RAD51L1,RAF1,RB1,RCBTB2,RIOK3,RNF111,RNF125,RNF135,RNF138,RNF180,RNF44,RNFT1,ROCK1,RTN1,SEC31A,SEPSECS,SERPINB7,SLC12A2,SLC25A17,SLC25A18,SLC25A31,SLC6A6,SLFN13,SMAD2,SMAD4,SNX6,SORCS1,SOS2,SOX7,SPATA18,SPATA4,SPG11,SQRDL,SRD5A3,SRFBP1,ST6GALNAC1,ST8SIA5,STAMBPL1,STRN3,STXBP4,SUGT1,TAOK1,TEP1,THRB,TIGD2,TLR10,TLR3,TM9SF3,TMEM144,TMEM165,TNKS2,TP53,TPT1,TUSC3,TXK,UBE2E1,UBE2K,UBR2,UGT8,USP32,USP53,VAPA,ZBTB1,ZBTB25,ZBTB9,ZFP1,ZKSCAN2,ZNF253,ZNF254,ZNF330,ZNF35,ZNF43,ZNF626,ZNF675,ZNF85* |
| 2000433 | 264 | *ABCD3,ACSL1,ACTN1,ADH7,ADORA3,AGXT2L1,ALB,ALG6,ALOX5AP,ALPK1,ANGPTL3,ANXA10,ARF4,ARG2,ARHGAP11A,ARIH2,ARL2BP,ASB15,ASB17,ASB5,ATP12A,ATXN3,BAHD1,BAI2,BAZ1A,BCL6,BRAF,BRCA2,BRDT,BST1,BTBD8,BTC,C11orf58,C14orf109,C14orf28,C14orf37,C14orf45,C15orf24,C19orf42,C19orf43,C3orf14,C3orf23,C4orf14,C4orf23,CALD1,CAPZA2,CASD1,CASP3,CASP6,CCL22,CCNB2,CCNG2,CD38,CDC14A,CDC42BPB,CDKL2,CDKN3,CHI3L2,CHKA,CLCC1,CNOT4,COCH,CPB2,CPT2,CRMP1,CSF1,CTBS,CTDSPL,CX3CR1,CXCL6,CYP4X1,DAAM1,DCTD,DEPDC1,DFFA,DIS3L,DUSP8,EIF4A2,ELMOD2,ENPP6,EPHA5,ERRFI1,FA2H,FAM175A,FBXO22,FBXO3,FBXO34,FBXW7,FGF2,FGF7,FLNB,FOS,FOXD2,FOXF1,GAB1,GABRB1,GCSH,GIMAP4,GNG5,GNPDA2,GPR125,GPR22,GRK4,GTF2H1,HDAC1,HERC3,HERC5,HES1,HESX1,HTN1,HYAL2,IFNA1,IFNA13,IFNW1,IFRD1,IL8,IPP,ISL2,JUB,KAT2B,KLHDC1,KLHL36,KNG1,KPNA3,LMO4,LNX1,LPXN,LRAT,LRFN5,LRRFIP2,LRRIQ3,LUZP1,MAB21L1,MAPK10,MEGF11,MEIS2,METT5D1,METTL3,MIA2,MICAL2,MIPOL1,MLH1,MPP5,MS4A5,MTF1,MTF2,MTHFD1,MUDENG,N4BP2L2,NAE1,NDRG2,NDUFA5,NPY1R,NR0B2,NR1D2,NR3C2,NRIP3,OLFM4,OLFML1,OLFML3,ORC6L,PAK7,PCGF3,PDHA2,PDYN,PHACTR4,PHF7,PIGK,PLCL2,PNRC2,PON2,PPARG,PPAT,PPP1R13B,PRDM8,PRKACB,PRKD1,PRNP,PRTG,PTGER2,PTN,RAB2B,RAB33B,RABGGTB,RAD18,RAD51L1,RAF1,RAG2,RB1,RBP7,RCAN3,RCBTB2,RLN2,RNF111,RNF133,RNF24,RPE65,RSBN1,RTN1,S1PR1,SACS,SAMD13,SEC31A,SENP2,SEPSECS,SLC25A31,SLC6A6,SNX6,SOS2,SPATA18,SPATA4,SPG11,SQRDL,SRD5A3,SSX2IP,ST7L,STOML3,STRN3,SUGT1,TARDBP,TAS1R1,TEK,TEP1,TESK2,THAP3,THRB,TIGD2,TLR10,TLR3,TMEM144,TMEM165,TMEM56,TMEM59,TPT1,TPTE2,TRIM24,TSGA14,TTC9C,TXK,UBE2E1,UBE2K,UBE2U,UBQLNL,UGT8,USP12,USP33,USP53,YIPF1,ZBTB1,ZBTB25,ZFP1,ZNF214,ZNF253,ZNF254,ZNF326,ZNF330,ZNF35,ZNF43,ZNF626,ZNF644,ZNF675,ZNF800,ZNF85,ZRANB2* |

**Table S6. STOP genes that are subject to copy-number loss (copy number < 0.7 times the average plody) in each tumor.**

| Sample | Number of STOP genes subject to copy-number loss | STOP genes subject to copy-number loss |
|---|---|---|
| 57701999 | 235 | *ACACB,ACYP2,ADAMTS6,ADNP2,ADRBK2,AIG1,AKAP12,AKAP7,ALB,ALDH3A2,ANKRD55,APAF1,ARF4,ARHGAP11A,ARIH2,AVPR1A,BAHD1,BAI2,BCL11A,BDP1,BMI1,BPIL2,BRWD1,BTC,C11orf58,C12orf29,C13orf27,C14orf45,C15orf24,C15orf42,C17orf68,C19orf42,C19orf43,C3orf14,C3orf23,C4orf14,C5orf41,C6orf170,C9orf64,CADM2,CARTPT,CCDC28A,CCDC99,CCNB2,CCNG2,CDC123,CDC42BPB,CDH2,CDKL2,CDKN1A,CENPH,COX10,CTDSPL,CTNS,CX3CR1,CXCL6,CYP26B1,DCN,DDX1,DIABLO,DIS3L,DNAH10,DNMT3L,DOCK2,DYRK2,EFCAB6,EIF2AK2,ENPP1,ENTHD1,EP300,EPB41L2,EPHA5,ESCO1,ESR1,FAM175A,FAM84A,FAM8A1,FBXO15,FBXO22,FGF7,FLNB,FOS,FRK,FSHR,GCNT2,GEN1,GJA1,GMCL1,GPC5,GPR158,GPR180,GRIK1,GTF2H1,HCFC2,HERC3,HERC5,HESX1,HMGN1,HMHB1,HNRNPUL1,HNRPLL,HS6ST3,HTN1,HTR1F,HYAL2,IFNA1,IFNA13,IFNAR1,IFNW1,IFT81,IGF1,IL31RA,IL8,IQGAP1,IRAK3,IRAK4,ISL2,KAT2B,KDSR,KIF6,LAIR1,LIG4,LNX1,LRRFIP2,LUZP1,MAFG,MAP2K6,MAP3K1,MAPK10,MCFD2,MCHR1,MEGF11,MEIS2,MELK,METTL7A,MITF,MKX,MLH1,MSH6,MTHFD1,MYB,MYLIP,NAIP,NF2,NLRP9,NR0B2,NR1D2,NR2F2,NRSN1,NSUN6,NT5DC3,NTRK2,NUDT5,PACSIN2,PAPOLG,PDZRN4,PHACTR4,PHF7,PIK3C3,PLCL2,PLN,PLXNC1,PMAIP1,PNRC2,PPAT,PPP1R13B,PRDM8,PRKAR1A,PRTG,PTPLA,PTPRK,PTTG1,PTTG1IP,RAP2A,RCAN3,RIOK3,RLN2,RNF111,RNF125,RNF138,RNF180,ROCK1,SEC31A,SERAC1,SERINC1,SERPINB7,SLC25A17,SLC38A2,SLC41A2,SMAD2,SMAD4,SMU1,SNRNP48,SNX9,SOCS2,SOCS5,SOX1,SPATA18,SPG11,SQRDL,SRD5A3,SSR1,ST6GALNAC1,ST8SIA5,STON1-GTF2A1L,SUPT7L,SYNJ1,TEK,THRB,TIGD2,TMEM165,TMPO,TP53,TRAPPC10,TRIM39,UBE2E1,UHRF1BP1L,USP34,UTRN,VIP,VRK2,ZBTB1,ZBTB2,ZBTB25,ZBTB9,ZIC2,ZNF253,ZNF254,ZNF35,ZNF43,ZNF571,ZNF626,ZNF664,ZNF675,ZNF85* |
| 990090 | 221 | *ACSL1,ACTN1,ADAMTS6,ADH7,ADNP2,ADRBK2,AGXT2L1,AIG1,AKAP12,AKAP7,ALB,ALPK1,ANXA10,ARG2,ARHGAP11A,ARL2BP,ASB5,ATP5J,ATP6V1E1,AUH,BAHD1,BDP1,BPIL2,BRWD1,BTC,BTG3,C10orf28,C10orf79,C14orf37,C14orf45,C15orf24,C15orf42,C4orf14,C4orf23,C6orf170,C8orf4,C9orf6,C9orf64,CARTPT,CASP3,CASP6,CCDC28A,CCL22,CCNB2,CCNG2,CD109,CDH2,CDKL2,CDKN3,CENPH,CH25H,CHUK,CLTC,COCH,COIL,CRMP1,CTSL1,CXCL6,CYP2C19,DAAM1,DCTD,DNMT3L,ECM2,EFCAB6,ELMOD2,ENPP1,ENPP6,ENTHD1,EP300,EPB41L2,EPHA5,ERI1,ESR1,FA2H,FAM175A,FAM8A1,FBXO22,FBXO25,FBXO34,FBXW7,FGF2,FGF7,FOS,FOXF1,GAB1,GCNT2,GCSH,GIMAP4,GJA1,GPR125,GPR21,GRIK1,GRK4,HERC3,HERC5,HMGN1,HTN1,HTR7,IFNA1,IFNA13,IFNAR1,IFNW1,IL8,INA,INVS,IQGAP1,ISL2,JAM2,KIF20B,KLHL36,LIPI,LIPJ,LRAT,MAFG,MAPK10,MCHR1,MEGF11,MEIS2,METTL3,MPP5,MTHFD1,MTUS1,MUDENG,MYB,MYLIP,NAE1,NAIP,NDRG2,NF2,NPY1R,NR2F2,NR3C2,NRIP1,NTRK2,NUDT12,OGN,PACSIN2,PCGF3,PDHA2,PIGW,PIK3C3,PLCE1,PLN,PPAT,PPP2CB,PRDM8,PRKD1,PRTG,PTGER2,PTGR1,PTP4A1,PTPRK,PTRH2,PTTG1IP,RAB2B,RAB33B,RAD51C,RAD51L1,RLN2,RNF111,RNF125,RNF138,RNF180,RNFT1,RTN1,SEC31A,SECISBP2,SEPSECS,SERAC1,SERINC1,SLC25A17,SLC25A18,SLC25A31,SLC46A2,SLFN13,SMAD2,SMAD4,SNRNP48,SNX9,SOX7,SPATA4,SPG11,SPTLC1,SQRDL,SRD5A3,SSR1,ST8SIA5,STAMBPL1,STRN3,STXBP4,SYNJ1,TEK,TEP1,TGFBR1,TIGD2,TLR10,TLR3,TLR4,TM9SF3,TMEM144,TMEM165,TNKS2,TPTE,TRAPPC10,TUSC3,UBE2K,UGT8,USP32,USP53,UTRN,VIP,VPS13A,VPS26A,WHSC1L1,ZBTB1,ZBTB2,ZBTB25,ZBTB43,ZBTB6,ZFP1,ZNF330* |

**Table S6. STOP genes that are subject to copy-number loss (copy number < 0.7 times the average plody) in each tumor.**

| Sample | Number of STOP genes subject to copy-number loss | STOP genes subject to copy-number loss |
|---|---|---|
| 2000403 | 196 | *ABCD3,ACSL1,ACVR2A,ADH7,ADORA3,AFF3,AGXT2L1,ALB,ALG6,ALPK1,ANGPTL3,ANKRD44,ANXA10,ARL6IP6,ASB17,ASB5,ATP5J,AXIN1,B3GALT1,BAI2,BMI1,BRDT,BRWD1,BST1,BTBD8,BTC,BTG3,C13orf27,C16orf63,C16orf75,C2orf29,C2orf47,C4orf14,C4orf23,CASP3,CASP6,CCDC74A,CCDC74B,CCNG2,CD302,CD38,CDC14A,CDKL2,CHI3L2,CLCC1,COBLL1,COPS8,COQ10B,CPT2,CRMP1,CSF1,CTBS,CTLA4,CXCL6,CYP4X1,DCTD,DEPDC1,DFFA,DNMT3L,EIF4E2,ELMOD2,ENPP6,EPHA5,ERRFI1,FAM175A,FBXL3,FBXW7,FGF2,FOXD2,FRZB,GAB1,GABRB1,GAD1,GCA,GNG5,GNPDA2,GPC5,GPD2,GPR125,GPR180,GRB14,GRIK1,GRK4,GTDC1,HAT1,HDAC1,HERC3,HERC5,HMGN1,HS6ST3,HTN1,IFNA1,IFNA13,IFNAR1,IFNW1,IL1R1,IL8,INPP1,IPP,IQCK,ITGA6,JAM2,KCNJ3,KCTD18,LIG4,LIPI,LIPT1,LMO4,LNX1,LRAT,LRRIQ3,LUZP1,MAPK10,MARS2,MFF,MMADHC,MTF1,MTF2,MYCBP2,MYO1B,NPY1R,NR0B2,NR3C2,NRIP1,NSUN6,OLFML3,ORC4L,PCGF3,PDHA2,PGAP1,PHACTR4,PIGK,PKP4,PNRC2,POLR2D,PPAT,PRDM8,PRKACB,PTTG1IP,RAB33B,RABGGTB,RAP2A,RBP7,RCAN3,RPE65,RSBN1,S1PR1,SAMD13,SCN1A,SEC31A,SEPSECS,SLC19A3,SLC20A1,SLC25A31,SOX1,SPATA18,SPATA4,SRD5A3,SSX2IP,ST7L,STK17B,STK39,STRADB,SYNJ1,TANK,TARDBP,TAS1R1,TBC1D4,TEK,TESK2,THAP3,TIGD2,TLR10,TLR3,TMEM144,TMEM165,TMEM56,TMEM59,TPTE,TRAPPC10,TXK,UBE2K,UBE2U,UCHL3,UGT8,USP33,USP53,YIPF1,ZIC2,ZKSCAN2,ZNF107,ZNF326,ZNF330,ZNF644,ZNF804A,ZRANB2* |
| 990203 | 183 | *ABCD3,ACTN1,ADNP2,ADORA3,AHRR,ALDH3A2,ALG6,ANGPTL3,AP3M2,ARF4,ARG2,ARHGAP11A,ARIH2,ARL2BP,ASB17,BAHD1,BAI2,BRDT,BST1,BTBD8,C14orf45,C15orf24,C17orf68,C19orf43,C3orf14,C3orf23,C4orf23,C8orf4,CADM2,CCL22,CD109,CD2AP,CD38,CDC14A,CDH2,CDKN1A,CHI3L2,CLCC1,COX10,CPT2,CRMP1,CSF1,CTBS,CTDSPL,CTNS,CX3CR1,CYP4X1,DAP,DEPDC1,DFFA,DNAH5,EIF2AK2,ENPP5,ERI1,ERRFI1,FA2H,FAM8A1,FBXO15,FBXO25,FLNB,FOS,FOXD2,FOXF1,FSHR,GABRB1,GCNT2,GCSH,GNG5,GNPDA2,GPR110,GPR125,GRK4,GSTA3,HDAC1,HESX1,HNRPLL,HTR1F,HYAL2,IFNA1,IFNA13,IFNW1,IPP,KAT2B,KDSR,KIF6,KLHL36,LMO4,LPXN,LRRFIP2,LRRIQ3,LUZP1,MARCH6,MCFD2,MEIS2,MELK,MITF,MLH1,MS4A5,MSH6,MTF1,MTF2,MTUS1,MYLIP,NAE1,NR0B2,NR1D2,NRSN1,OLFML3,ORC6L,PCGF3,PHACTR4,PHF7,PIGK,PIK3C3,PLCL2,PMAIP1,PNRC2,PPARG,PPP2CB,PRKACB,PTP4A1,RABGGTB,RAD18,RAD51L1,RAF1,RBP7,RCAN3,RLN2,RNF125,RNF138,RNF170,ROCK1,RPE65,RSBN1,S1PR1,SAMD13,SEPSECS,SERPINB7,SLC20A2,SLC6A6,SMAD2,SMAD4,SMU1,SNRNP48,SOCS5,SORCS1,SOX7,SPG11,SQRDL,SSR1,SSX2IP,ST7L,ST8SIA5,STON1-GTF2A1L,TARDBP,TAS1R1,TAS2R1,TEK,TESK2,THAP3,THRB,TLR10,TMEM56,TMEM59,TP53,TRIM39,TTC9C,TUSC3,TXK,UBE2E1,UBE2K,UBE2U,UBR2,USP33,VAPA,WHSC1L1,YIPF1,ZBTB9,ZFP1,ZNF326,ZNF35,ZNF644,ZRANB2* |
| 980390 | 181 | *ACSL1,ACTN1,ADAMTS6,ADH7,ADNP2,ADRBK2,AGXT2L1,ALB,ALPK1,ANKRD55,ANXA10,AP3S1,ARG2,ARL2BP,ASB5,ATP5J,ATP6V1E1,ATXN3,BAZ1A,BDP1,BPIL2,BST1,BTC,BTG3,C14orf109,C14orf28,C14orf37,C14orf45,C4orf14,C4orf23,C5orf41,CARTPT,CASP3,CASP6,CCDC99,CCL22,CCNG2,CD38,CDH2,CDKL2,CDKN3,CENPH,CHD1,COCH,CRMP1,CXCL6,DAAM1,DCTD,DOCK2,DUSP8,EGR1,ELMOD2,ENPP6,EPHA5,ERAP2,ESCO1,FA2H,FAM175A,FAM81B,FAM8A1,FBXO15,FBXO34,FBXW7,FER,FGF2,FOS,FOXF1,G3BP1,GAB1,GABRB1,GCNT2,GCSH,GDF9,GNPDA2,GPR125,GRIK1,GRK4,HERC3,HERC5,HMHB1,HNRNPH1,HTN1,IFNA1,IFNA13,IFNAR1,IFNW1,IL31RA,IL8,JAM2,JUB,KCNN2,KDSR,KLHDC1,KLHL36,LIPI,LNX1,LRAT,LRFN5,LYRM7,MAP3K1,MAPK10,METTL3,MIA2,MIPOL1,MPP5,MTHFD1,MUDENG,MYLIP,NAE1,NAIP,NDRG2,NF2,NPY1R,NR3C2,NRIP1,NRIP3,NRSN1,NUDT12,OLFML1,ORC6L,PDHA2,PIK3C3,PJA2,PMAIP1,PPAT,PRDM8,PRKD1,PTGER2,PTTG1,RAB2B,RAB33B,RAD51L1,RGS2,RIOK3,RLN2,RNF125,RNF138,RNF180,RNF44,ROCK1,RTN1,SEC31A,SEPSECS,SERPINB7,SLC12A2,SLC25A18,SLC25A31,SMAD2,SMAD4,SMU1,SNRNP48,SNX6,SOS2,SPATA18,SPATA4,SRD5A3,SRFBP1,SSR1,ST8SIA5,STRN3,SYNJ1,TEK,TEP1,TIGD2,TLR10,TLR3,TMEM144,TMEM165,TPTE,TPTE2,TXK,UBE2K,UBQLNL,UGT8,USP53,VAPA,ZBTB1,ZBTB25,ZFP1,ZNF214,ZNF330* |

**Table S6. STOP genes that are subject to copy-number loss (copy number < 0.7 times the average plody) in each tumor.**

| Sample | Number of STOP genes subject to copy-number loss | STOP genes subject to copy-number loss |
|---|---|---|
| 96141474 | 167 | *ACSL1,ADAMTS6,ADH7,ADNP2,AGXT2L1,AHRR,AIG1,AKAP12,AKAP7,ALB,ALPK1,AMD1,ANKRD55,ANXA10,AP3S1,ASB5,ATG5,ATP5J,AUH,BDP1,BRWD1,BTC,BTG3,C17orf68,C3orf14,C4orf14,C6orf170,C9orf6,C9orf64,CADM2,CARTPT,CASP3,CASP6,CCDC28A,CCNG2,CD109,CDH10,CDH9,CDKL2,CENPH,CGA,CHD1,COX10,CTNS,CTSL1,CXCL6,DAP,DCTD,DDX1,DNAH5,ECM2,EGR1,ELMOD2,ENPP1,ENPP6,EPB41L2,EPHA5,ERAP2,ESR1,FAM175A,FAM81B,FAM84A,FBXO15,FBXW7,FER,FGF2,FRK,GAB1,GDF9,GEN1,GJA1,GRIK1,GTF3C6,HERC3,HERC5,HMGN1,HMHB1,HTN1,HTR1F,IBTK,IFNA1,IFNA13,IFNAR1,IFNW1,IL31RA,IL8,INVS,JAM2,KCNN2,KDSR,LIFR,LMBRD2,LNX1,LRAT,LYRM7,MAP3K1,MAPK10,MARCH6,MITF,MYB,NAIP,NPY1R,NR3C2,NRIP1,NTRK2,NUDT12,OGN,PDHA2,PIK3C3,PJA2,PLN,PMAIP1,PNRC1,PPAT,PRDM1,PRDM8,PTGR1,PTP4A1,PTPRK,RAB33B,RAD18,RAI14,RARS2,REV3L,RLN2,RNF125,RNF138,RNF180,ROCK1,SEC31A,SEC63,SECISBP2,SERAC1,SERINC1,SERPINB7,SLC12A2,SLC25A31,SLC46A2,SMAD2,SMAD4,SNX9,SPATA18,SPATA4,SPTLC1,SRD5A3,SRFBP1,ST8SIA5,SYNJ1,TAS2R1,TEK,TGFBR1,TIGD2,TLR3,TLR4,TMEM144,TMEM165,TP53,TRAF3IP2,UGT8,USP45,USP53,UTRN,VAPA,VIP,VPS13A,ZBTB2,ZNF330* |
| 38877042 | 157 | *ACSL1,ADAMTS6,ADH7,ADNP2,AGXT2L1,ALB,ALDH3A2,ALPK1,ANKRD55,ANXA10,AP3S1,ASB5,ATP5J,BDP1,BPTF,BRWD1,BST1,BTC,BTG3,C17orf68,C4orf14,C4orf23,C5orf41,C9orf6,CARTPT,CASP3,CASP6,CCDC99,CCL7,CCNG2,CD38,CDKL2,CENPH,CHD1,CLTC,COIL,COX10,CPD,CRMP1,CTNS,CXCL6,DCTD,DNMT3L,DOCK2,DUXA,EGR1,ELMOD2,ENPP6,EPHA5,ERAP2,FAM175A,FAM81B,FAM8A1,FBXW7,FER,FGF2,GAB1,GABRB1,GCNT2,GNPDA2,GPR125,GPR21,GRIK1,GRK4,HERC3,HERC5,HMGN1,HMHB1,HNRNPH1,HNRNPUL1,HTN1,IFNAR1,IL31RA,IL8,INVS,JAM2,KCNN2,KDSR,LAIR1,LIPI,LNX1,LRAT,MAFG,MAP2K6,MAP3K1,MAPK10,MYLIP,NAIP,NLRP9,NPY1R,NR3C2,NRIP1,NRSN1,NUDT12,OMG,PCGF3,PDHA2,PIGW,PIK3C3,PJA2,PMAIP1,PPAT,PRDM8,PRKAR1A,PRKCA,PTGR1,PTRH2,PTTG1,PTTG1IP,RAB33B,RAD51C,RNF135,RNF138,RNF180,RNF44,RNFT1,SEC31A,SEPSECS,SERPINB7,SLC25A31,SLC46A2,SLFN13,SMAD2,SMAD4,SNRNP48,SPATA18,SPATA4,SRD5A3,SRFBP1,SSR1,ST6GALNAC1,ST8SIA5,STXBP4,SYNJ1,TAOK1,TGFBR1,TIGD2,TLR10,TLR3,TLR4,TMEM144,TMEM165,TP53,TPTE,TRAPPC10,TXK,UBE2K,UGT8,USP32,USP53,ZBTB43,ZBTB6,ZNF330,ZNF417,ZNF549,ZNF571,ZNF587* |
| 980156 | 155 | *ACSL1,ADAMTS6,ADH7,AGXT2L1,ALB,ALOX5AP,ALPK1,ANKRD55,ANXA10,AP3M2,AP3S1,ARL2BP,ASB5,ATP12A,ATP5J,ATRNL1,AXIN1,BDP1,BRCA2,BST1,BTC,C10orf28,C10orf79,C10orf96,C13orf27,C16orf75,C4orf23,C8orf4,C9orf6,CADM2,CARTPT,CASP3,CASP6,CCL22,CCNG2,CD38,CDKL2,CENPH,CH25H,CHD1,CHKA,CHUK,CRMP1,CXCL12,CXCL6,CYP26B1,CYP2C19,DCTD,EGR1,ELMOD2,ENPP6,EPHA5,ERAP2,FA2H,FAM175A,FAM81B,FBXO25,FBXW7,FER,FGF2,FOXF1,GAB1,GABRB1,GCSH,GDF9,GMCL1,GNPDA2,GPC5,GPR125,GPR180,GPR21,GRIK1,GRK4,HERC3,HERC5,HS6ST3,HTN1,HTR1F,HTR7,IFNA1,IFNA13,IFNW1,IL31RA,IL8,INA,JAM2,KCNN2,KIF20B,KLHL36,LIG4,LIPJ,LPXN,LRAT,LYRM7,MAB21L1,MAP3K1,MAPK10,MITF,MS4A5,N4BP2L2,NAE1,NAIP,NHLRC2,NPY1R,NR3C2,NUDT12,OAT,ORC6L,PCGF3,PDHA2,PHACTR4,PJA2,PLCE1,PPP2CB,PPP3CB,PRDM8,PTGR1,RAB33B,RAD51L1,RAP2A,RLN2,RNF170,RNF180,SACS,SEC31A,SEPSECS,SLC12A2,SLC20A2,SLC46A2,SMU1,SORCS1,SOX1,SPATA4,SRFBP1,STAMBPL1,STOML3,TIGD2,TLR10,TLR3,TLR4,TM9SF3,TMEM144,TNKS2,TPTE2,TTC9C,UBE2K,UGT8,USP12,USP53,VPS26A,WHSC1L1,ZBTB6,ZFP1,ZIC2,ZNF330* |
| 990046 | 145 | *AADACL2,ACSL1,ADH7,ADNP2,AGTR1,AGXT2L1,ALB,ALPK1,ANXA10,ARF4,ARIH2,ARL6,ASB5,ASTE1,ATG3,ATP5J,BCL6,BRWD1,BTC,BTG3,C12orf60,C3orf14,C3orf15,C3orf23,C4orf14,CADM2,CASP3,CASP6,CCNG2,CCRL1,CDKL2,CDKN1B,CEP70,CEP97,CLEC12A,CLEC2B,CLEC4A,CP,CREBL2,CSTA,CTDSPL,CX3CR1,CXCL6,DCTD,DNMT3L,EIF4A2,ELMOD2,EMP1,ENPP6,EPHA5,FAM175A,FBXL14,FBXO15,FBXW7,FGF2,FKBP4,FLNB,GAB1,GPR15,GRIK1,GSK3B,GTPBP8,HERC3,HERC5,HES1,HESX1,HMGN1,HTN1,HTR1F,HYAL2,IFNAR1,IL8,JAM2,KAT2B,KDSR,KLHL6,KLRB1,KLRF1,KNG1,KPNA4,LEKR1,LIPI,LNX1,LRAT,LRRFIP2,MAPK10,MFAP5,MGP,MITF,MLH1,MME,NDUFB4,NMNAT3,NPY1R,NR1D2,NR3C2,NRIP1,P2RY13,PARP15,PDHA2,PFN2,PHF7,PIK3C3,PIK3R4,PLCL2,PLSCR4,PMAIP1,PPARG,PPAT,PRDM8,PTTG1IP,RAB33B,RAD18,RAF1,RNF13,RNF7,RSRC1,SEC31A,SENP2,SERPINB7,SKIL,SLC25A31,SLC6A6,SLCO2A1,SMAD2,SMAD4,SPATA18,SPATA4,SRD5A3,ST8SIA5,STRAP,SYNJ1,THPO,THRB,TIGD2,TLR3,TMEM144,TMEM165,TPTE,TRAPPC10,UBE2E1,UGT8,USP53,ZNF330,ZNF35* |

**Table S6. STOP genes that are subject to copy-number loss (copy number < 0.7 times the average plody) in each tumor.**

| Sample | Number of STOP genes subject to copy-number loss | STOP genes subject to copy-number loss |
|---|---|---|
| 2000175 | 142 | *ACSL1,ADAMTS6,ADH7,ADNP2,AGXT2L1,ALDH3A2,ALPK1,ANKRD55,ANXA10,ARF4,ARIH2,ASB5,ATP5J,BCL6,BDP1,BST1,BTG3,C12orf60,C17orf68,C19orf42,C19orf43,C3orf14,C3orf23,C4orf23,CADM2,CARTPT,CASP3,CASP6,CD38,CDH2,CDKN1B,CENPH,CLEC12A,CLEC2B,COX10,CREBL2,CRMP1,CTDSPL,CTNS,CX3CR1,DCTD,EIF4A2,ELMOD2,EMP1,ENPP6,FAM175A,FBXO15,FBXW7,FGF2,FLNB,G3BP1,GAB1,GABRB1,GNPDA2,GPR21,GRIK1,GRK4,HERC3,HERC5,HES1,HESX1,HTR1F,HYAL2,IFNA1,IFNA13,IFNW1,IL31RA,JAM2,KAT2B,KDSR,KLRF1,KNG1,LIPI,LRAT,LRRFIP2,MAP3K1,MAPK10,MELK,MGP,MITF,MLH1,NAIP,NPY1R,NR1D2,NR3C2,NRIP1,OAT,PCGF3,PDHA2,PHF7,PIK3C3,PLCL2,PMAIP1,PPARG,PRDM8,PTGR1,PTTG1,RAB33B,RAD18,RAF1,RIOK3,RLN2,RNF125,RNF138,RNF180,ROCK1,SEC31A,SENP2,SERPINB7,SLC25A31,SLC46A2,SLC6A6,SMAD2,SMAD4,SMU1,SPATA4,ST8SIA5,SYNJ1,TEK,THRB,TIGD2,TLR10,TLR3,TLR4,TMEM144,TP53,TPTE,TXK,UBE2E1,UBE2K,UGT8,USP53,ZBTB43,ZBTB6,ZNF253,ZNF254,ZNF330,ZNF35,ZNF43,ZNF626,ZNF675,ZNF85* |
| 980417 | 136 | *ACSL1,ADAMTS6,ADH7,AGXT2L1,AHRR,ALB,ALPK1,ANKRD55,ANXA10,AP3S1,ASB5,ATP5J,AUH,BDP1,BRWD1,BTC,BTG3,C11orf58,C19orf42,C19orf43,C4orf14,C5orf41,C9orf6,C9orf64,CARTPT,CASP3,CASP6,CCDC99,CCNG2,CDH10,CDH9,CDKL2,CENPH,CHD1,CTSL1,CXCL6,DAP,DCTD,DNAH5,DNMT3L,DOCK2,DUSP8,ECM2,EGR1,ELMOD2,ENPP6,EPHA5,ERAP2,FAM175A,FAM81B,FBXW7,FER,FGF2,G3BP1,GAB1,GDF9,GPR21,GRIK1,GTF2H1,HERC3,HERC5,HMGN1,HMHB1,HNRNPH1,HTN1,IFNAR1,IL31RA,IL8,INVS,JAM2,KCNN2,LIFR,LIPI,LMBRD2,LNX1,LRAT,LRRC6,LYRM7,MAP3K1,MAPK10,MARCH6,MICAL2,NAIP,NPY1R,NR3C2,NRIP1,NRIP3,NTRK2,NUDT12,OGN,OLFML1,PDHA2,PJA2,PPAT,PRDM8,PTGR1,PTTG1,PTTG1IP,RAB33B,RAI14,RNF180,RNF44,SEC31A,SECISBP2,SLC12A2,SLC25A31,SLC46A2,SPATA18,SPATA4,SPTLC1,SRD5A3,SRFBP1,SYNJ1,TAS2R1,TGFBR1,TIGD2,TLR3,TLR4,TMEM144,TMEM165,TPTE,TRAPPC10,UBQLNL,UGT8,USP53,VPS13A,ZBTB43,ZBTB6,ZNF214,ZNF253,ZNF254,ZNF330,ZNF43,ZNF626,ZNF675,ZNF85* |
| 990396 | 124 | *ACSL1,ADH7,ADNP2,AGXT2L1,AIG1,AKAP12,AKAP7,ALOX5AP,ALPK1,AMD1,ARF4,ARHGAP11A,ARIH2,ASB5,ATG5,ATP12A,BRCA2,C13orf34,C15orf24,C1orf49,C3orf23,C4orf14,C6orf170,CASP3,CASP6,CCDC28A,CD109,CD55,CDH2,CGA,CPB2,CTDSPL,CX3CR1,DCTD,ENPP1,ENPP6,EPB41L2,EPHA5,ESR1,FBXL3,FBXO15,FLNB,FRK,GIMAP4,GJA1,GTF3C6,HESX1,HYAL2,IBTK,IER5,JUB,KAT2B,KDSR,KIFAP3,KPNA3,LNX1,LRRFIP2,LYST,MAB21L1,MEIS2,METTL3,MLH1,MYB,MYCBP2,N4BP2L2,NDRG2,NR1D2,OLFM4,OPN3,PCDH9,PHF7,PIK3C3,PLN,PMAIP1,PNRC1,PPAT,PRDM1,PTPRK,RAB2B,RARS2,RB1,RCBTB2,REV3L,RNF125,RNF138,SACS,SCYL3,SEC63,SERAC1,SERINC1,SERPINB7,SERPINC1,SLC19A2,SMAD2,SMAD4,SNX9,SPATA18,SPATA4,SRD5A3,ST8SIA5,STOML3,SUGT1,SYT14,TBC1D4,TEP1,THRB,TLR3,TMEM165,TNFSF4,TOR1AIP1,TPT1,TPTE2,TRAF3IP2,TRIM58,UBE2E1,UCHL3,UGT8,USP12,USP45,UTRN,VIP,YOD1,ZBTB2,ZNF35* |
| 990475 | 113 | *ACTN1,ADAMTS6,ADRBK2,ALDH3A2,ANKRD55,ARF4,ARG2,ARIH2,ATG3,ATP5J,ATP6V1E1,ATRNL1,ATXN3,BPIL2,BRWD1,BTG3,C10orf28,C10orf79,C10orf96,C14orf109,C14orf37,C14orf45,C17orf68,C3orf15,C3orf23,CDC42BPB,CENPH,CH25H,CHUK,COX10,CSTA,CTDSPL,CTNS,CX3CR1,CYP2C19,DAAM1,DNMT3L,EFCAB6,ENTHD1,EP300,FAM8A1,FLNB,FOS,GCNT2,GRIK1,GSK3B,GSTA3,GTPBP8,HESX1,HMGN1,HTR7,HYAL2,IFNA1,IFNA13,IFNAR1,IFNW1,IL31RA,INA,JAM2,KAT2B,KIF20B,LIPI,LIPJ,LRRFIP2,MAP3K1,MCHR1,MELK,MLH1,MPP5,MTHFD1,MUDENG,MYLIP,NAIP,NDUFB4,NF2,NHLRC2,NR1D2,NRIP1,NRSN1,PACSIN2,PARP15,PHF7,PLCE1,PLCL2,PPARG,PPP1R13B,PTTG1IP,RAD18,RAD51L1,RAF1,RLN2,RNF180,RTN1,SLC25A17,SLC25A18,SLC6A6,SMU1,SNRNP48,SORCS1,SSR1,STAMBPL1,SYNJ1,TEK,THRB,TM9SF3,TNKS2,TP53,TPTE,TRAPPC10,UBE2E1,ZBTB1,ZBTB25,ZNF35* |

**Table S6. STOP genes that are subject to copy-number loss (copy number < 0.7 times the average plody) in each tumor.**

| Sample | Number of STOP genes subject to copy-number loss | STOP genes subject to copy-number loss |
|---|---|---|
| 2000892 | 102 | *ACTN1,ADNP2,ALDH3A2,ARG2,ARHGAP20,ATXN3,AXIN1,BACE1,BAHD1,C14orf109,C14orf28,C14orf37,C14orf45,C17orf68,C5orf41,CCDC99,CCNB2,CDC42BPB,CDH2,CDKN3,COCH,COIL,COPS8,COX10,CTLA4,CTNS,DAAM1,DOCK2,EIF4E2,ERI1,FAM55A,FBXO15,FBXO25,FBXO34,FOS,FXYD2,G3BP1,GDF9,HMHB1,HNRNPH1,IFNA1,IFNA13,IFNW1,INPP1,JUB,KBTBD3,KDSR,KLHDC1,LRFN5,MEIS2,METTL3,MFF,MMP20,MPP5,MTHFD1,MTUS1,MUDENG,MYO1B,NDRG2,NR2F2,PGAP1,PIGW,PIK3C3,PMAIP1,POU2AF1,PPP1R13B,PPP2CB,PRKD1,PRTG,PTGER2,PTTG1,RAB2B,RAD51L1,RDX,RIOK3,RLN2,RNF111,RNF125,RNF138,RNF44,RTN1,SERPINB7,SLC19A3,SLFN13,SMAD2,SMAD4,SOS2,SOX7,SPG11,SQRDL,ST8SIA5,STK17B,STRADB,STRN3,STXBP4,TEK,TEP1,TP53,TPTE2,TUSC3,ZBTB1,ZBTB25* |
| 20020448 | 92 | *ADNP2,ADRBK2,AHRR,AKAP12,ALDH3A2,ARHGAP11A,ATP6V1E1,AXIN1,BAHD1,BAI2,BDP1,BPIL2,BPTF,C15orf24,C17orf68,C4orf23,CARTPT,CCL7,CCNB2,CDC42BPB,CHKA,CLTC,COIL,COX10,CPD,CRMP1,CTNS,DIS3L,DNMT3L,DUSP8,EFCAB6,ENTHD1,EP300,ESR1,FBXO22,FBXO25,FGF19,FGF7,FOXF1,GIMAP4,GRK4,HDAC1,HNRNPH1,ISL2,MAFG,MAP2K6,MCHR1,MEGF11,MEIS2,MELK,MYEOV,NAE1,NAIP,NCOA6,NF2,NR0B2,OMG,PACSIN2,PCGF3,PHACTR4,PIGW,PPP1R13B,PPP2CB,PRKAR1A,PRKCA,PRTG,PTRH2,PTTG1IP,RAD51C,RBL1,RNF111,RNF135,RNF44,RNFT1,SERAC1,SLC25A17,SLC25A18,SLFN13,SNX9,SOX1,SPG11,SQRDL,ST6GALNAC1,STXBP4,TAOK1,TP53,TRAPPC10,TRIM58,TTC9C,USP32,VIP,ZBTB2* |
| 990108 | 82 | *ACSL1,ADNP2,AHRR,ALDH3A2,ARIH2,ATP6V1E1,AXIN1,C17orf68,C19orf42,C19orf43,C4orf23,CASP3,CCL7,CDC42BPB,CHKA,CPD,CRMP1,CTNS,DIABLO,DNAH10,DNMT3L,DUSP8,DUXA,EFCAB6,EGR1,ENPP6,ENTHD1,EP300,FBXO25,FGF19,FOXF1,GRK4,HNRNPH1,HNRNPUL1,HYAL2,KLHL36,LAIR1,LRRC32,MAFG,MCHR1,MELK,METTL7A,MYEOV,NLRP9,OMG,PACSIN2,PCGF3,PHF7,PIGW,PPARG,PPP1R13B,PTTG1IP,RAF1,RNF135,RNF44,SLC25A17,SLC25A18,SLC6A6,SLFN13,SMU1,SNRNP48,SOX1,SSR1,ST6GALNAC1,TAOK1,TLR3,TP53,TRAPPC10,TTC9C,VPS26A,ZBTB43,ZNF253,ZNF254,ZNF417,ZNF43,ZNF549,ZNF571,ZNF587,ZNF626,ZNF664,ZNF675,ZNF85* |
| 970005 | 78 | *ADAMTS6,ADH7,ADNP2,AGXT2L1,ALB,ALPK1,ANKRD55,AP3S1,BDP1,BST1,BTC,C4orf14,C4orf23,CARTPT,CASP6,CCNG2,CD38,CDKL2,CENPH,CRMP1,CXCL6,EGR1,EPHA5,ERAP2,FAM175A,FAM81B,FBXO15,FER,FGF2,GABRB1,GDF9,GNPDA2,GPR125,GRK4,HERC3,HERC5,HTN1,IFNA1,IFNA13,IFNW1,IL31RA,IL8,KCNN2,KDSR,LNX1,LYRM7,MAP3K1,MAPK10,NAIP,PCGF3,PDHA2,PIK3C3,PJA2,PMAIP1,PPAT,PRDM8,RLN2,RNF125,RNF138,RNF180,SEC31A,SEPSECS,SERPINB7,SLC12A2,SMAD2,SMAD4,SPATA18,SRD5A3,SRFBP1,ST8SIA5,TEK,TIGD2,TLR10,TMEM165,TXK,UBE2K,UGT8,USP53* |
| 990041 | 74 | *ACTN1,ALDH3A2,AMD1,ARG2,ATXN3,BAI2,BAZ1A,BPTF,C14orf109,C14orf28,C14orf37,C14orf45,C17orf68,CCL7,CDC42BPB,CDKN3,CLTC,COCH,COIL,COX10,CPD,CTNS,DAAM1,FBXO34,FOS,GTF3C6,HDAC1,JUB,KLHDC1,LRFN5,LUZP1,MAFG,MAP2K6,METTL3,MIA2,MIPOL1,MPP5,MTF1,MTHFD1,MUDENG,NDRG2,NR0B2,OMG,PHACTR4,PIGW,PNRC2,PPP1R13B,PRKAR1A,PRKCA,PRKD1,PTGER2,PTRH2,RAB2B,RAD51C,RAD51L1,RCAN3,REV3L,RNF135,RNFT1,RTN1,SEC63,SLFN13,SNX6,SOS2,ST6GALNAC1,STRN3,STXBP4,TAOK1,TEP1,TP53,TRAF3IP2,USP32,ZBTB1,ZBTB25* |
| 990275 | 68 | *AFF3,ALDH3A2,ATXN3,AUH,BRAF,BRWD1,C14orf109,C17orf68,C19orf42,C19orf43,C2orf29,C5orf41,C9orf6,C9orf64,CCDC99,CDC42BPB,COX10,CTNS,CTSL1,DNAH10,DNMT3L,DOCK2,ECM2,ERI1,FBXO25,FOS,GIMAP4,HMGN1,IL1R1,INVS,LIPT1,LNX1,MELK,METTL3,MTUS1,NDRG2,NTRK2,OAT,OGN,PPP1R13B,PPP2CB,PTGR1,PTTG1IP,RAB2B,RAG2,RGS2,SECISBP2,SLC46A2,SMU1,SOX7,SPATA18,SPTLC1,TEP1,TGFBR1,TLR4,TP53,TRAPPC10,TUSC3,VPS13A,VPS26A,ZNF253,ZNF254,ZNF43,ZNF571,ZNF626,ZNF664,ZNF675,ZNF85* |
| 2000201 | 62 | *AIG1,AKAP12,AKAP7,ALDH3A2,AMD1,ARHGAP11A,ATG5,BAHD1,C15orf24,C15orf42,C17orf68,C6orf170,CCDC28A,CCNB2,CD109,CGA,COX10,CTNS,DIS3L,ENPP1,EPB41L2,ESR1,FBXO22,FGF7,FRK,GJA1,GTF3C6,IBTK,IFNA1,IFNA13,IFNW1,IQGAP1,ISL2,MEGF11,MEIS2,MELK,MYB,NR2F2,PLN,PNRC1,PRDM1,PRTG,PTP4A1,PTPRK,RARS2,REV3L,RLN2,RNF111,SEC63,SERAC1,SERINC1,SMU1,SNX9,SPG11,SQRDL,TEK,TP53,TRAF3IP2,USP45,UTRN,VIP,ZBTB2* |

**Table S6. STOP genes that are subject to copy-number loss (copy number < 0.7 times the average plody) in each tumor.**

| Sample | Number of STOP genes subject to copy-number loss | STOP genes subject to copy-number loss |
|---|---|---|
| 2000068 | 53 | *ADRBK2,ARHGAP20,ATP5J,ATP6V1E1,BACE1,BPIL2,BRWD1,BTG3,DIABLO,DNMT3L,EFCAB6,ENTHD1,EP300,ERRFI1,FAM55A,FEZ1,FXYD2,GRIK1,HMGN1,IFNA1,IFNA13,IFNAR1,IFNW1,JAM2,JRKL,KBTBD3,LIPI,MCHR1,MELK,METTL7A,MMP20,MRE11A,NF2,NRIP1,PACSIN2,POU2AF1,PTTG1IP,RDX,RLN2,ROCK1,SLC25A17,SLC25A18,SLC37A2,SLC38A2,SMU1,SYNJ1,TAF1D,TAS1R1,TEK,THAP3,TPTE,TRAPPC10,VAPA* |
| 66811693 | 52 | *ABCD3,ADORA3,ALG6,ANGPTL3,AP3S1,ASB17,BRDT,BTBD8,CDC14A,CHD1,CHI3L2,CLCC1,COPS8,CSF1,CTBS,DEPDC1,EGR1,EIF4E2,ERAP2,FAM81B,FER,GDF9,GNG5,HMHB1,KCNN2,KDSR,LMO4,LRRIQ3,LYRM7,MTF2,NUDT12,OLFML3,PIGK,PJA2,PMAIP1,PRKACB,RABGGTB,RPE65,RSBN1,S1PR1,SAMD13,SERPINB7,SLC12A2,SRFBP1,SSX2IP,ST7L,TMEM56,UBE2U,USP33,ZNF326,ZNF644,ZRANB2* |
| 990355 | 49 | *AP3S1,ARF4,ARIH2,BDP1,C3orf14,C3orf23,C5orf41,CADM2,CARTPT,CCDC99,CHD1,CTDSPL,CX3CR1,DOCK2,EGR1,ERAP2,FAM81B,FER,FLNB,G3BP1,GDF9,HESX1,HMHB1,HNRNPH1,HTR1F,HYAL2,KAT2B,KCNN2,LRRFIP2,LYRM7,MITF,MLH1,NAIP,NR1D2,NUDT12,PHF7,PJA2,PLCL2,PPARG,PTTG1,RAD18,RAF1,RNF44,SLC12A2,SLC6A6,SRFBP1,THRB,UBE2E1,ZNF35* |
| 990071 | 48 | *ACSL1,ADNP2,ALDH3A2,ASB5,AXIN1,BST1,C17orf68,C4orf23,CASP3,CD38,CDH2,CHD1,COX10,CRMP1,CTNS,DCTD,ENPP6,ERAP2,ERI1,FAM81B,FBXO15,FBXO25,FER,FEZ1,GPR125,GRK4,KDSR,LUZP1,NUDT12,PCGF3,PIK3C3,PJA2,PMAIP1,RIOK3,RNF125,RNF138,SEPSECS,SERPINB7,SLC25A31,SLC37A2,SMAD2,SMAD4,SOX7,SPATA4,ST8SIA5,TLR3,TP53,TUSC3* |
| 990412 | 40 | *AP3M2,ARHGAP20,BACE1,C19orf42,C19orf43,C8orf4,C9orf64,ERI1,FAM55A,FBXO25,FEZ1,FXYD2,IFNA1,IFNA13,IFNW1,KBTBD3,LPXN,MELK,MS4A5,MTUS1,NTRK2,POU2AF1,PPP2CB,RDX,RLN2,RNF170,SLC20A2,SLC37A2,SMU1,SOX7,TEK,TUSC3,VPS13A,WHSC1L1,ZNF253,ZNF254,ZNF43,ZNF626,ZNF675,ZNF85* |
| 990044 | 40 | *ADNP2,ALG10,ARHGAP11A,BAHD1,C12orf60,C15orf24,CDH2,CDKN1B,CLEC12A,CLEC2B,CLEC4A,CREBL2,EMP1,ERGIC2,FBXL14,FBXO15,FGF7,FGFR1OP2,FKBP4,KDSR,KLRB1,KLRF1,KRAS,LRFN5,MEIS2,MFAP5,MGP,PIK3C3,PMAIP1,PPFIBP1,RIOK3,RNF125,RNF138,SERPINB7,SMAD2,SMAD4,SPG11,SQRDL,ST8SIA5,STRAP* |
| 2000040 | 30 | *C14orf28,C4orf23,CRMP1,DFFA,ERRFI1,FAM8A1,GCNT2,GRK4,KLHDC1,LRFN5,LUZP1,METTL3,MIA2,MYLIP,NDRG2,NR0B2,NRSN1,PCGF3,PNRC2,RAB2B,RBP7,RCAN3,SLCO2A1,SNRNP48,SOS2,SSR1,TARDBP,TAS1R1,TEP1,THAP3* |
| 990339 | 30 | *ADNP2,ARL2BP,BST1,C4orf23,CCL22,CD38,CDH2,CRMP1,FA2H,FBXO15,FOXF1,GCNT2,GCSH,GPR125,GRK4,KDSR,KLHL36,NAE1,ORC6L,PCGF3,PMAIP1,RIOK3,RNF125,RNF138,SEPSECS,SERPINB7,SMAD4,SNRNP48,SSR1,ZFP1* |
| 990228 | 25 | *AUH,C9orf6,C9orf64,CTSL1,ECM2,GPR21,IFNA1,IFNA13,IFNW1,INVS,MELK,NTRK2,OGN,PTGR1,RLN2,SECISBP2,SLC46A2,SMU1,SPTLC1,TEK,TGFBR1,TLR4,VPS13A,ZBTB43,ZBTB6* |
| 20020011 | 22 | *AGXT2L1,ALPK1,ARF4,CADM2,CASP6,FBXO15,FGF2,FLNB,GABRB1,GNPDA2,HESX1,HTR1F,KDSR,PHF7,PMAIP1,SERPINB7,SLC25A31,SMAD4,TXK,UBE2K,UGT8,USP53* |
| 980029 | 22 | *BAZ1A,C12orf60,CDC123,CDKN1B,CLEC12A,CLEC2B,CLEC4A,CREBL2,EMP1,ERGIC2,FBXL14,FGFR1OP2,FKBP4,KLRB1,KLRF1,MFAP5,MGP,MIPOL1,NUDT5,PPFIBP1,SNX6,STRAP* |
| 2000088 | 20 | *ADCY1,AP3M2,C8orf4,CHCHD7,ERI1,FBXO25,IMPAD1,MTFR1,MTUS1,PDE7A,PENK,PPP2CB,RNF170,SDCBP,SLC20A2,SNAI2,SOX7,TTPA,TUSC3,WHSC1L1* |
| 990489 | 16 | *ATXN3,C14orf109,C19orf42,C19orf43,CDC42BPB,CXCL12,DUSP8,MAFG,PPP1R13B,ST6GALNAC1,ZNF253,ZNF254,ZNF43,ZNF626,ZNF675,ZNF85* |
| 990060 | 16 | *ADH7,ALDH3A2,C17orf68,COPS8,COX10,CTNS,EIF4E2,FAM175A,HERC3,HERC5,MAPK10,PDHA2,PRDM8,SEC31A,TIGD2,TP53* |
| 980447 | 14 | *ATP5J,ATXN3,BRWD1,BTG3,C14orf109,DNMT3L,GRIK1,HMGN1,JAM2,LIPI,NRIP1,PTTG1IP,TPTE,TRAPPC10* |

**Table S6. STOP genes that are subject to copy-number loss (copy number < 0.7 times the average plody) in each tumor.**

| Sample | Number of STOP genes subject to copy-number loss | STOP genes subject to copy-number loss |
|---|---|---|
| 990300 | 14 | *C12orf60,CDKN1B,CLEC12A,CLEC2B,CLEC4A,CREBL2,EMP1,FBXL14,FKBP4,KLRB1,KLRF1,MFAP5,MGP,STRAP* |
| 87622942 | 13 | *ARL2BP,BTG3,CCL22,FA2H,FOXF1,GCSH,KLHL36,LIPI,NAE1,NRIP1,ORC6L,TPTE,ZFP1* |
| 990005 | 13 | *ADNP2,FBXL14,FBXO15,FKBP4,KDSR,PIK3C3,PMAIP1,RNF125,RNF138,SERPINB7,SMAD2,SMAD4,ST8SIA5* |
| 2000362 | 12 | *BACE1,FAM55A,FEZ1,FXYD2,IFNA1,IFNA13,IFNW1,MELK,RLN2,SLC37A2,SMU1,TEK* |
| 2000303 | 12 | *AP3M2,C8orf4,ERI1,FBXO25,FOXF1,MTUS1,PPP2CB,RNF170,SLC20A2,SOX7,TUSC3,WHSC1L1* |
| 2000877 | 8 | *ATG3,BMI1,FAM81B,GPR15,GTPBP8,LPXN,LRFN5,RLN2* |
| 990097 | 6 | *DFFA,ERRFI1,RBP7,TARDBP,TAS1R1,THAP3* |
| 980437 | 6 | *ATP5J,BTG3,JAM2,LIPI,NRIP1,TPTE* |
| 980369 | 5 | *ALDH3A2,C17orf68,COX10,CTNS,TP53* |
| 91228050 | 2 | *ADNP2,FBXO15* |
| 2000441 | 1 | *GIMAP4* |
| 2000169 | 1 | *DCTD* |
| 46404174 | 1 | *SKIL* |
| 990170 | 1 | *PDHA2* |
| 980021 | 0 | |
| 61669256 | 0 | |
| 990111 | 0 | |
| 2000242 | 0 | |
| 990474 | 0 | |
| 32226415 | 0 | |
| 990010 | 0 | |
| 58947266 | 0 | |
| 980401 | 0 | |
| 47492137 | 0 | |
| 980097 | 0 | |
| 2000286 | 0 | |
| 2000085 | 0 | |
| 20263644 | 0 | |

**Avg** 91.11

**Table S7. CYCLOPS genes that are subject to copy-number loss (copy number < 0.7 times the average ploidy) in each tumor.**

| Sample | Number of CYCLOPS genes subject to copy-number loss | CYCLOPS genes subject to copy-number loss |
|---|---|---|
| 76629543 | 39 | *ALDH2,BMP8A,CEBPG,CPT1B,CSF3R,EEF1A1,EEF2,EIF2B2,ENC1,EPHB2,ETFDH,FBXO6,GRIK5,HPGD,LSM4,OBP2A,PABPN1,PAFAH1B1,PARK7,PGF,PMPCB,POLG,PSMA4,PSMC2,PSMC4,RBM17,RPS11,RPS15,RPS9,SF3A2,SMC2,SMU1,SNRNP70,SRP68,TIE1,TIMM50,UBA52,UBE2L6,ZNF583* |
| 990195 | 29 | *ADSL,ASCL3,EEF2,EIF2B2,ENC1,ETFDH,GRIK5,HPGD,LSM4,NUTF2,OBP2A,PABPN1,PDGFB,PGF,PHF5A,POLR2F,PSMC4,RBM17,RPS11,RPS15,RPS9,SBK1,SF3A2,SMC2,SMU1,SNRNP70,TIMM50,UBA52,ZNF583* |
| 990172 | 28 | *ALDH2,ASCL3,BMP8A,CSF3R,EEF1A1,EEF2,EIF2B2,ENC1,EPHB2,ETFDH,FBXO6,HPGD,LSM4,PABPN1,PAFAH1B1,PARK7,PGF,POLG,PSMA4,RPS15,SBK1,SF3A2,SMC2,SMU1,SRP68,TIE1,UBA52,UBE2L6* |
| 57701999 | 27 | *ADSL,ALDH2,CEBPG,CPT1B,EEF2,EIF2B2,EPHB2,GRIK5,LSM4,PAFAH1B1,PDGFB,PGF,PHF5A,POLG,POLR2F,PSMA4,PSMC4,RBM17,RPS11,RPS15,RPS9,SF3A2,SMU1,SNRNP70,SRP68,TIMM50,UBA52* |
| 2000433 | 26 | *ASCL3,BMP8A,CSF3R,EEF2,EIF2B2,EPHB2,ETFDH,FBXO6,HPGD,LSM4,NUPL1,NUTF2,PABPN1,PARK7,PCNA,PGF,PMPCB,PSMA4,PSMC2,RPS15,SF3A2,SNRPB,TIE1,UBA52,UBE2L6,USPL1* |
| 990515 | 24 | *ADSL,BMP8A,CPT1B,CSF3R,EEF2,EIF2B2,ENC1,EPHB2,ETFDH,FBXO6,HPGD,LSM4,PARK7,PDGFB,PGF,PHF5A,POLR2F,RPS15,SBK1,SF3A2,SMU1,TIE1,UBA52,UBE2L6* |
| 57689477 | 23 | *ADSL,ALDH2,CPT1B,EEF2,EIF2B2,ENC1,ETFDH,HPGD,NUTF2,OBP2A,PABPN1,PAFAH1B1,PDGFB,PGF,PHF5A,PMPCB,POLR2F,PSMC2,RPS15,SF3A2,SMC2,SMU1,SRP68* |
| 990069 | 23 | *ADSL,CPT1B,EEF2,EIF2B2,ENC1,ETFDH,HPGD,LSM4,NUTF2,PABPN1,PAFAH1B1,PDGFB,PGF,PHF5A,POLG,POLR2F,PSMA4,RPS15,SBK1,SF3A2,SRP68,TIAF1,UBA52* |
| 73291145 | 22 | *ADSL,CPT1B,EEF2,EIF2B2,ENC1,ETFDH,HPGD,LSM4,NUPL1,NUTF2,PABPN1,PDGFB,PGF,PHF5A,POLG,POLR2F,PSMA4,RPS15,SF3A2,SMU1,SRP68,UBA52* |
| 990108 | 21 | *ADSL,CEBPG,CPT1B,GRIK5,LSM4,OBP2A,PAFAH1B1,PDGFB,PHF5A,POLR2F,PSMC4,RPS11,RPS9,SBK1,SMU1,SNRNP70,SRP68,TIAF1,TIMM50,UBA52,ZNF583* |
| 38877042 | 16 | *CEBPG,ENC1,ETFDH,GRIK5,HPGD,OBP2A,PAFAH1B1,PSMC4,RPS11,RPS9,SMC2,SNRNP70,SRP68,TIAF1,TIMM50,ZNF583* |
| 990203 | 16 | *BMP8A,CSF3R,EEF1A1,EEF2,EIF2B2,EPHB2,FBXO6,NUTF2,PAFAH1B1,PARK7,PGF,RPS15,SF3A2,SMU1,TIE1,UBE2L6* |
| 990090 | 16 | *ADSL,CPT1B,EEF1A1,EIF2B2,ENC1,ETFDH,HPGD,NUTF2,OBP2A,PDGFB,PGF,PHF5A,POLG,POLR2F,PSMA4,SMC2* |

**Table S7. CYCLOPS genes that are subject to copy-number loss (copy number < 0.7 times the average ploidy) in each tumor.**

| Sample | Number of CYCLOPS genes subject to copy-number loss | CYCLOPS genes subject to copy-number loss |
|---|---|---|
| 20020720 | 14 | *BMP8A,CSF3R,EIF2B2,ENC1,EPHB2,ETFDH,FBXO6,HPGD,PARK7,PGF,PMPCB,PSMC2,SMU1,TIE1* |
| 980011 | 12 | *BMP8A,CSF3R,EEF1A1,EPHB2,ETFDH,FBXO6,HPGD,PABPN1,PAFAH1B1,PARK7,SMC2,TIE1* |
| 990275 | 12 | *CPT1B,EEF2,EIF2B2,LSM4,OBP2A,PAFAH1B1,PGF,RPS15,SF3A2,SMC2,SMU1,UBA52* |
| 20020448 | 11 | *ADSL,CPT1B,ENC1,NUTF2,OBP2A,PAFAH1B1,PDGFB,PHF5A,POLR2F,SRP68,TIAF1* |
| 980417 | 11 | *ASCL3,EEF2,ENC1,ETFDH,HPGD,LSM4,OBP2A,RPS15,SF3A2,SMC2,UBA52* |
| 2000403 | 10 | *BMP8A,CSF3R,EPHB2,ETFDH,FBXO6,HPGD,PARK7,RBM17,SBK1,TIE1* |
| 2000175 | 10 | *EEF2,ENC1,ETFDH,HPGD,LSM4,PAFAH1B1,RPS15,SF3A2,SMU1,UBA52* |
| 990098 | 9 | *EEF1A1,EIF2B2,ETFDH,HPGD,NUTF2,PABPN1,PGF,RBM17,SMC2* |
| 990475 | 9 | *ADSL,CPT1B,EIF2B2,PAFAH1B1,PDGFB,PGF,PHF5A,POLR2F,SMU1* |
| 980390 | 9 | *ASCL3,EIF2B2,ENC1,ETFDH,HPGD,NUTF2,PABPN1,PGF,SMU1* |
| 980156 | 8 | *ENC1,ETFDH,HPGD,NUPL1,NUTF2,SMC2,SMU1,USPL1* |
| 2000068 | 8 | *ADSL,CPT1B,FBXO6,PARK7,PDGFB,PHF5A,POLR2F,SMU1* |
| 990041 | 8 | *CSF3R,EIF2B2,EPHB2,PABPN1,PAFAH1B1,PGF,SRP68,TIAF1* |
| 990412 | 7 | *EEF2,LSM4,RPS15,SF3A2,SMU1,UBA52,UBE2L6* |
| 990489 | 6 | *EEF2,LSM4,RPS15,SF3A2,SRP68,UBA52* |
| 2000892 | 6 | *CPT1B,EIF2B2,PABPN1,PAFAH1B1,PGF,PSMA4* |
| 96141474 | 6 | *EEF1A1,ENC1,ETFDH,HPGD,PAFAH1B1,SMC2* |
| 2000362 | 5 | *EEF2,OBP2A,RPS15,SF3A2,SMU1* |
| 2000201 | 5 | *EEF1A1,PAFAH1B1,POLG,PSMA4,SMU1* |
| 990396 | 5 | *EEF1A1,HPGD,NUPL1,PABPN1,USPL1* |
| 2000040 | 3 | *EPHB2,FBXO6,PARK7* |
| 990228 | 3 | *OBP2A,SMC2,SMU1* |
| 990071 | 3 | *EPHB2,HPGD,PAFAH1B1* |
| 2000242 | 2 | *RPS15,SF3A2* |
| 990097 | 2 | *FBXO6,PARK7* |
| 990046 | 2 | *ETFDH,HPGD* |
| 87622942 | 1 | *NUTF2* |
| 980369 | 1 | *PAFAH1B1* |
| 970005 | 1 | *ENC1* |
| 980029 | 1 | *RBM17* |
| 2000877 | 1 | *UBE2L6* |
| 990060 | 1 | *PAFAH1B1* |
| 990339 | 1 | *NUTF2* |
| 990355 | 1 | *ENC1* |

**Table S7. CYCLOPS genes that are subject to copy-number loss (copy number < 0.7 times the average ploidy) in each tumor.**

| Sample | Number of CYCLOPS genes subject to copy-number loss | CYCLOPS genes subject to copy-number loss |
|---|---|---|
| 980021 | 0 | |
| 61669256 | 0 | |
| 2000441 | 0 | |
| 2000169 | 0 | |
| 20020011 | 0 | |
| 990111 | 0 | |
| 980447 | 0 | |
| 2000088 | 0 | |
| 990474 | 0 | |
| 66811693 | 0 | |
| 990300 | 0 | |
| 32226415 | 0 | |
| 46404174 | 0 | |
| 990010 | 0 | |
| 58947266 | 0 | |
| 91228050 | 0 | |
| 980401 | 0 | |
| 990170 | 0 | |
| 47492137 | 0 | |
| 980097 | 0 | |
| 2000286 | 0 | |
| 980437 | 0 | |
| 2000303 | 0 | |
| 990005 | 0 | |
| 2000085 | 0 | |
| 20263644 | 0 | |
| 990044 | 0 | |

**Avg** 6.81

**Table S8. CYCLOPS genes subject to copy-number loss in 74 gastric adenocarcinomas. Copy-number loss is defined as a region where genomic copy number is < 0.7 times the average ploidy.**

| Gene | Gene name | Number of tumors where gene is subject to loss | Median mRNA level (RMA) | | Differ-ence | Percent reduction in tumors with copy-number loss | P-value[b] | FDR[b] |
|---|---|---|---|---|---|---|---|---|
| | | | Copy-number loss | No copy-number loss or gain[a] | | | | |
| *SMU1* | smu-1 suppressor of mec-8 and unc-52 homolog (C. elegans) | 20 | 6.26 | 6.47 | -0.21 | 14 | 0.001 | 0.027 |
| *RBM17* | RNA binding motif protein 17 | 6 | 7.34 | 7.63 | -0.29 | 18 | 0.002 | 0.040 |
| *EEF2** | eukaryotic translation elongation factor 2 | 16 | 12.56 | 12.88 | -0.32 | 20 | 0.005 | 0.070 |
| *SRP68* | signal recognition particle 68kDa | 11 | 9.37 | 9.59 | -0.22 | 14 | 0.006 | 0.070 |
| *ETFDH** | electron-transferring-flavoprotein dehydrogenase | 20 | 6.51 | 7.37 | -0.86 | 45 | 0.007 | 0.070 |
| *PABPN1* | poly(A) binding protein, nuclear 1 | 13 | 8.06 | 8.37 | -0.31 | 19 | 0.009 | 0.073 |
| *PMPCB* | peptidase (mitochondrial processing) beta | 4 | 6.96 | 7.10 | -0.15 | 10 | 0.015 | 0.108 |
| *POLG* | polymerase (DNA directed), gamma | 7 | 5.98 | 6.15 | -0.18 | 12 | 0.022 | 0.138 |
| *ENC1** | ectodermal-neural cortex 1 (with BTB domain) | 18 | 9.27 | 9.90 | -0.62 | 35 | 0.028 | 0.154 |
| *TIMM50* | translocase of inner mitochondrial membrane 50 homolog (S. cerevisiae) | 5 | 6.96 | 7.32 | -0.36 | 22 | 0.065 | 0.271 |
| *SF3A2* | splicing factor 3a, subunit 2, 66kDa | 17 | 7.24 | 7.78 | -0.54 | 31 | 0.065 | 0.271 |
| *ALDH2* | aldehyde dehydrogenase 2 family (mitochondrial) | 4 | 10.87 | 11.22 | -0.35 | 21 | 0.066 | 0.271 |
| *EEF1A1* | eukaryotic translation elongation factor 1 alpha 1 | 9 | 11.78 | 11.90 | -0.11 | 8 | 0.074 | 0.279 |
| *PSMA4* | proteasome (prosome, macropain) subunit, alpha type, 4 | 9 | 10.77 | 10.83 | -0.06 | 4 | 0.085 | 0.296 |
| *PAFAH1B1* | platelet-activating factor acetylhydrolase 1b, regulatory subunit 1 (45kDa) | 20 | 7.86 | 8.13 | -0.27 | 17 | 0.091 | 0.298 |
| *SNRNP70* | small nuclear ribonucleoprotein 70kDa (U1) | 5 | 7.38 | 7.44 | -0.06 | 4 | 0.097 | 0.298 |
| *NUPL1* | nucleoporin like 1 | 4 | 6.05 | 6.34 | -0.29 | 18 | 0.113 | 0.325 |
| *POLR2F* | polymerase (RNA) II (DNA directed) polypeptide F | 11 | 7.00 | 7.03 | -0.02 | 2 | 0.121 | 0.330 |
| *RPS15* | ribosomal protein S15 | 17 | 8.84 | 8.90 | -0.07 | 5 | 0.144 | 0.371 |
| *CPT1B* | carnitine palmitoyltransferase 1B (muscle) | 13 | 6.09 | 6.27 | -0.18 | 12 | 0.174 | 0.417 |
| *HPGD* | hydroxyprostaglandin dehydrogenase 15-(NAD) | 22 | 7.76 | 7.97 | -0.21 | 14 | 0.179 | 0.417 |
| *UBA52* | ubiquitin A-52 residue ribosomal protein fusion product 1 | 14 | 12.54 | 12.71 | -0.16 | 11 | 0.187 | 0.417 |
| *USPL1* | ubiquitin specific peptidase like 1 | 3 | 5.40 | 5.65 | -0.25 | 16 | 0.205 | 0.437 |
| *TIE1* | tyrosine kinase with immunoglobulin-like and EGF-like domains 1 | 8 | 4.87 | 5.11 | -0.24 | 16 | 0.241 | 0.477 |
| *PGF* | placental growth factor | 18 | 7.69 | 7.76 | -0.07 | 4 | 0.243 | 0.477 |
| *PARK7* | parkinson protein 7 | 11 | 12.17 | 12.24 | -0.07 | 5 | 0.302 | 0.557 |
| *PSMC2* | proteasome (prosome, macropain) 26S subunit, ATPase, 2 | 4 | 8.45 | 9.07 | -0.62 | 35 | 0.316 | 0.557 |
| *NUTF2* | nuclear transport factor 2 | 13 | 8.55 | 8.59 | -0.04 | 3 | 0.347 | 0.557 |
| *GRIK5* | glutamate receptor, ionotropic, kainate 5 | 5 | 5.77 | 5.86 | -0.09 | 6 | 0.361 | 0.557 |
| *ADSL* | adenylosuccinate lyase | 11 | 9.44 | 9.48 | -0.03 | 2 | 0.374 | 0.557 |
| *PHF5A* | PHD finger protein 5A | 11 | 8.65 | 8.63 | 0.03 | -2 | 0.374 | 0.557 |

**Table S8. CYCLOPS genes subject to copy-number loss in 74 gastric adenocarcinomas. Copy-number loss is defined as a region where genomic copy number is < 0.7 times the average ploidy.**

| Gene | Gene name | Number of tumors where gene is subject to loss | Median mRNA level (RMA) | | Differ-ence | Percent reduction in tumors with copy-number loss | P-value[b] | FDR[b] |
|---|---|---|---|---|---|---|---|---|
| | | | Copy-number loss | No copy-number loss or gain[a] | | | | |
| *CEBPG* | CCAAT/enhancer binding protein (C/EBP), gamma | 4 | 8.04 | 8.36 | -0.32 | 20 | 0.385 | 0.557 |
| *BMP8A* | bone morphogenetic protein 8a | 8 | 5.41 | 5.52 | -0.11 | 7 | 0.387 | 0.557 |
| *UBE2L6* | ubiquitin-conjugating enzyme E2L 6 | 7 | 9.03 | 9.50 | -0.46 | 27 | 0.387 | 0.557 |
| *EPHB2* | EPH receptor B2 | 12 | 6.86 | 7.16 | -0.30 | 19 | 0.405 | 0.563 |
| *TIAF1* | TGFB1-induced anti-apoptotic factor 1 | 5 | 6.48 | 6.38 | 0.10 | -7 | 0.413 | 0.563 |
| *PDGFB* | platelet-derived growth factor beta polypeptide | 11 | 5.55 | 5.64 | -0.09 | 6 | 0.449 | 0.592 |
| *OBP2A* | odorant binding protein 2A | 11 | 5.34 | 5.43 | -0.09 | 6 | 0.459 | 0.592 |
| *ZNF583* | zinc finger protein 583 | 4 | 4.10 | 4.03 | 0.07 | -5 | 0.476 | 0.599 |
| *PSMC4* | proteasome (prosome, macropain) 26S subunit, ATPase, 4 | 5 | 9.43 | 9.40 | 0.03 | -2 | 0.520 | 0.637 |
| *SMC2* | structural maintenance of chromosomes 2 | 13 | 6.86 | 6.53 | 0.33 | -26 | 0.577 | 0.689 |
| *RPS11* | ribosomal protein S11 | 5 | 10.64 | 10.63 | 0.01 | -1 | 0.601 | 0.701 |
| *EIF2B2* | eukaryotic translation initiation factor 2B, subunit 2 beta, 39kDa | 18 | 8.43 | 8.30 | 0.14 | -10 | 0.726 | 0.810 |
| *CSF3R* | colony stimulating factor 3 receptor (granulocyte) | 9 | 7.12 | 6.95 | 0.18 | -13 | 0.728 | 0.810 |
| *RPS9* | ribosomal protein S9 | 5 | 9.60 | 9.58 | 0.03 | -2 | 0.809 | 0.881 |
| *FBXO6* | F-box protein 6 | 11 | 8.52 | 7.89 | 0.63 | -55 | 0.859 | 0.915 |
| *ASCL3* | achaete-scute complex homolog 3 (Drosophila) | 5 | 5.26 | 5.21 | 0.05 | -3 | 0.903 | 0.941 |
| *SBK1* | SH3-binding domain kinase 1 | 6 | 6.34 | 5.56 | 0.78 | -71 | 0.979 | 0.980 |
| *LSM4* | LSM4 homolog, U6 small nuclear RNA associated (S. cerevisiae) | 14 | 7.93 | 7.60 | 0.34 | -26 | 0.980 | 0.980 |
| *SNPRB* | small nuclear ribonucleoprotein polypeptides B and B1 | 1 | 10.63 | 10.55 | 0.08 | -6 | ** | ** |
| *PCNA* | proliferating cell nuclear antigen | 1 | 10.12 | 10.48 | -0.36 | 22 | ** | ** |
| *ETV2* | ets variant 2 | 0 | | | | | | |
| *LSM7* | LSM7 homolog, U6 small nuclear RNA associated (S. cerevisiae) | 0 | | | | | | |
| *MTHFD2L* | methylenetetrahydrofolate dehydrogenase (NADP+ dependent) 2-like | 0 | | | | | | |
| *PUF60* | poly-U binding splicing factor 60KDa | 0 | | | | | | |

* Subject to copy-number loss in ≥ 10% of tumors and median expression reduction ≥ 20% and FDR ≤ 0.25

** We computed the p value only when we had at least 2 samples where the gene is subject to copy-number loss

Highlighted green cell indicate median expression reduction ≥ 10% and FDR ≤ 0.25

[a] copy-number equal to average ploidy

[b] p values by one-sided Wilcoxon rank-sum test; FDR (false discovery rate) by the R p.adjust function with the 'fdr' method (Benjamini and Hochberg, 1995)

RMA = Robust Multiarray Average

**Table S9. Multivariate Cox proportional hazards analysis of overall survival.**

| Variable | Hazard ratio | 95% confidence interval | p |
|---|---|---|---|
| Copy-number loss in 3p as in Table 1 | 3.57 | 1.24 - 10.31 | 0.018 |
| Copy-number loss in 5q as in Table 1 | 4.57 | 1.05 - 19.86 | 0.042 |
| Copy-number loss in 17p as in Table 1 | 0.09 | 0.03 - 0.29 | 0.00009 |
| Copy-number loss in 18q as in Table 1 | 2.05 | 0.84 - 5.02 | 0.117 |
| Copy-number loss in 21q as in Table 1 | 0.28 | 0.08 - 1.08 | 0.064 |
| Age | 1.06 | 1.01 - 1.10 | 0.01 |
| Stage 2 | 0.63 | 0.13 - 3.16 | 0.573 |
| Stage 3 | 15.78 | 3.65 - 68.18 | 0.0002 |
| Stage 4 | 20.90 | 3.85 - 113.28 | 0.0004 |

Calculated using the coxph function in the R package survival.

This table shows the final model after applying the R function step to a starting model that included the following additional variables: copy-number loss in 4, 9p, 14q, 19p, gender, tumor grade, Lauren classification, G-INT/G-DIF subtype.

**Table S10. Univariate tests of association between copy-number loss of specific chromosomal arms as in Table 1 and clinical characteristics; none are significant after taking multiple hypothesis testing into account.**

| Clinical characteristic | Statistical test | Copy-number loss | | | | | | | | |
|---|---|---|---|---|---|---|---|---|---|---|
| | | 3p | 4 | 5q | 9p | 14q | 17p | 18q | 19p | 21q |
| **Gender** | Fisher's exact test | 1.0000 | 0.2509 | 0.7009 | 1.0000 | 0.3172 | 0.7616 | 0.7328 | 0.2057 | 0.7101 |
| **Lauren classification** | Fisher's exact test | 1.0000 | 0.5800 | 0.3705 | 0.0192 | 0.7566 | 0.6688 | 0.3525 | 0.2768 | 0.6208 |
| **TNM stage** | Cochran-Armitage test[a] | 0.2792 | 0.1257 | 0.1257 | 0.5170 | 0.8224 | 0.2882 | 0.6738 | 0.9299 | 0.5788 |
| **Tumor grade[b]** | Fisher's exact test | 1.0000 | 1.0000 | 0.0576 | 0.0023 | 0.6426 | 1.0000 | 0.0421 | 0.3595 | 0.1478 |
| **G-INT/G-DIF subtype** | Fisher's exact test | 0.7132 | 1.0000 | 0.6985 | 0.5123 | 0.3449 | 0.1365 | 1.0000 | 0.1998 | 1.0000 |
| **Adjuvant treatment** | Fisher's exact test | 0.1124 | 0.6348 | 1.0000 | 0.6697 | 0.5725 | 0.2585 | 0.1863 | 1.0000 | 0.1124 |

[a] As implemented in the function independence_test in the R "coin" package (http://cran.r-project.org/web/packages/coin/index.html); we used the quadratic test statistic.

[b] We combined undifferentiated and poorly differentiated because of the small number of undifferentiated tumors; we combined moderately differentiated and well differentiated because of the small number of well-differentiated tumors.

**Table S11. Comparisons of current results to Yustein et al. [3]**

| Chromosome arm | Yustein | | Current results | | P value* | FDR* |
|---|---|---|---|---|---|---|
| | LOH | No LOH | LOH | No LOH | | |
| 1p | 7 | 11 | 21 | 53 | 0.40 | 0.76 |
| 1q | 4 | 14 | 6 | 68 | 0.10 | 0.49 |
| 2p | 6 | 12 | 20 | 54 | 0.57 | 0.82 |
| 2q | 7 | 11 | 19 | 55 | 0.38 | 0.76 |
| 3p | 13 | 5 | 27 | 47 | 0.01 | 0.26 |
| 3q | 4 | 14 | 13 | 61 | 0.74 | 0.87 |
| 4p | 10 | 8 | 26 | 48 | 0.18 | 0.63 |
| 4q | 6 | 12 | 24 | 50 | 1.00 | 1.00 |
| 5p | 6 | 12 | 17 | 57 | 0.37 | 0.76 |
| 5q | 11 | 7 | 26 | 48 | 0.06 | 0.44 |
| 6p | 7 | 11 | 18 | 56 | 0.24 | 0.63 |
| 6q | 4 | 14 | 16 | 58 | 1.00 | 1.00 |
| 7p | 4 | 14 | 8 | 66 | 0.24 | 0.63 |
| 7q | 6 | 12 | 17 | 57 | 0.37 | 0.76 |
| 8p | 8 | 10 | 22 | 52 | 0.27 | 0.65 |
| 8q | 7 | 11 | 9 | 65 | 0.01 | 0.26 |
| 9p | 9 | 9 | 36 | 38 | 1.00 | 1.00 |
| 9q | 7 | 11 | 24 | 50 | 0.59 | 0.82 |
| 10p | 6 | 12 | 14 | 60 | 0.21 | 0.63 |
| 10q | 8 | 10 | 17 | 57 | 0.08 | 0.45 |
| 11p | 7 | 11 | 18 | 56 | 0.24 | 0.63 |
| 11q | 5 | 13 | 24 | 50 | 0.78 | 0.87 |
| 12p | 5 | 13 | 15 | 59 | 0.53 | 0.82 |
| 12q | 7 | 11 | 14 | 60 | 0.11 | 0.49 |
| 13q | 10 | 8 | 18 | 56 | 0.02 | 0.26 |
| 14q | 4 | 14 | 22 | 52 | 0.77 | 0.87 |
| 15q | 5 | 13 | 16 | 58 | 0.55 | 0.82 |
| 16p | 3 | 15 | 10 | 64 | 0.71 | 0.87 |
| 16q | 7 | 11 | 17 | 57 | 0.23 | 0.63 |
| 17p | 12 | 6 | 40 | 34 | 0.43 | 0.76 |
| 17q | 5 | 13 | 23 | 51 | 1.00 | 1.00 |
| 18p | 6 | 12 | 20 | 54 | 0.57 | 0.82 |
| 18q | 11 | 7 | 26 | 48 | 0.06 | 0.44 |
| 19p | 6 | 12 | 20 | 54 | 0.57 | 0.82 |
| 19q | 5 | 13 | 17 | 57 | 0.76 | 0.87 |
| 20p | 6 | 12 | 9 | 65 | 0.07 | 0.44 |
| 20q | 3 | 15 | 9 | 65 | 0.70 | 0.87 |
| 21q | 4 | 14 | 25 | 49 | 0.41 | 0.76 |
| 22q | 6 | 12 | 22 | 52 | 0.78 | 0.87 |

* P values by two-sided Fisher's exact tests; FDR (false discovery rate) by the R p.adjust function with the 'fdr' method [4].

We were unable to locate marker D21S413 (Table 2 in [3]) in the literature; therefore 21q is represented by only one locus in our data.

**Table S12. Top ranked CYCLOPS genes and their frequency of loss in reference [5] (3131 samples) and in our 74 samples.**

| Gene | Frequency of loss in reference | Number of our samples where it is subject to copy-number loss | Frequency of loss in our samples | P value* | FDR* |
|---|---|---|---|---|---|
| *PSMC2* | 0.1 | 4 | 0.05 | 0.2382 | 0.372 |
| *EIF2B2* | 0.17 | 18 | 0.24 | 0.1169 | 0.254 |
| *EEF2* | 0.27 | 16 | 0.22 | 0.3538 | 0.520 |
| *PHF5A* | 0.23 | 11 | 0.15 | 0.1221 | 0.254 |
| *HPGD* | 0.26 | 22 | 0.30 | 0.5030 | 0.635 |
| *RPS15* | 0.28 | 17 | 0.23 | 0.4311 | 0.599 |
| *SNRPB* | 0.13 | 1 | 0.01 | 0.0011 | 0.009 |
| *POLR2F* | 0.22 | 11 | 0.15 | 0.1562 | 0.300 |
| *USPL1* | 0.27 | 3 | 0.04 | 6.82E-07 | 1.70E-05 |
| *SMC2* | 0.21 | 13 | 0.18 | 0.5637 | 0.671 |
| *SMU1* | 0.25 | 20 | 0.27 | 0.6848 | 0.778 |
| *PUF60* | 0.08 | 0 | 0.00 | 0.0037 | 0.018 |
| *RPS11* | 0.19 | 5 | 0.07 | 0.0060 | 0.021 |
| *POLG* | 0.17 | 7 | 0.09 | 0.1137 | 0.254 |
| *ZNF583* | 0.2 | 4 | 0.05 | 0.0009 | 0.009 |
| *CPT1B* | 0.25 | 13 | 0.18 | 0.1730 | 0.309 |
| *BMP8A* | 0.12 | 8 | 0.11 | 0.8579 | 0.933 |
| *TIE1* | 0.11 | 8 | 0.11 | 1.0000 | 1.000 |
| *SF3A2* | 0.27 | 17 | 0.23 | 0.5082 | 0.635 |
| *SNRNP70* | 0.19 | 5 | 0.07 | 0.0060 | 0.021 |
| *RBM17* | 0.2 | 6 | 0.08 | 0.0110 | 0.034 |
| *PCNA* | 0.12 | 1 | 0.01 | 0.0016 | 0.010 |
| *PSMA4* | 0.18 | 9 | 0.12 | 0.2215 | 0.369 |
| *LSM4* | 0.2 | 14 | 0.19 | 1.0000 | 1.000 |
| *EEF1A1* | 0.2 | 9 | 0.12 | 0.1049 | 0.254 |

* P values for frequency of loss in gastric cancer versus the cancers in reference [5] by two-sided Fisher's exact tests; FDR (false discovery rate) by the R p.adjust function with the 'fdr' method [4].

**Supplementary References**

1. Tan IB, Ivanova T, Lim KH, Ong CW, Deng N, Lee J, et al. Intrinsic subtypes of gastric cancer, based on gene expression pattern, predict survival and respond differently to chemotherapy. Gastroenterology. 2011;141(2):476-85, 85 e1-11.

2. Therneau TM, Grambsch PM. Modeling Survival Data: Extending the Cox Model. New York: Springer; 2000.

3. Yustein AS, Harper JC, Petroni GR, Cummings OW, Moskaluk CA, Powell SM. Allelotype of gastric adenocarcinoma. Cancer Res. 1999;59(7):1437-41.

4. Benjamini Y, Hochberg Y. Controlling the false discovery rate: a practical and powerful approach to multiple testing. J R Statist Soc B. 1995;57(1):289-300.

5. Nijhawan D, Zack TI, Ren Y, Strickland MR, Lamothe R, Schumacher SE, et al. Cancer vulnerabilities unveiled by genomic loss. Cell. 2012;150(4):842-54.
