# Supplementary material for: Abundant copy-number loss of CYCLOPS and STOP genes in gastric adenocarcinoma
Source: Gastric Cancer. 2015 Jul 24;19:453–65. doi: 10.1007/s10120-015-0514-z (PMC4824836; doi:10.1007/s10120-015-0514-z)
Supplement: Supplementary file 3 — Supplementary material 3 (PDF 816 kb) [file 10120_2015_514_MOESM3_ESM.pdf]

Original article

Abundant copy-number loss of CYCLOPS and STOP genes in gastric adenocarcinoma

Ioana Cutcutache*[,1,2], Alice Yingting Wu*[,1, 2,3], Yuka Suzuki[1,2], John Richard McPherson[1,2], Zhengdeng Lei[1,2], Niantao Deng[1,4], Shenli Zhang[1], Wai Keong Wong[5], Khee Chee Soo[5,6], Weng Hoong Chan[5], London Lucien Ooi[5,6], Roy Welsch[3,7], Patrick Tan[1,3,8,9], Steven G. Rozen[1, 2,3]

* These authors contributed equally to this manuscript.

[1] Program in Cancer and Stem Cell Biology, Duke-NUS Graduate Medical School, Singapore

[2] Centre for Computational Biology, Duke-NUS Graduate Medical School, Singapore

[3] Computation and Systems Biology, Singapore-MIT Alliance

[4] NUS Graduate School for Integrative Science and Engineering, National University of Singapore, Singapore

[5] Department of General Surgery, Singapore General Hospital

[6] Division of Surgical Oncology, National Cancer Centre Singapore

[7] Engineering Systems Division and Sloan School of Management, Massachusetts Institute of Technology, MA, USA

[8] Duke-NUS Genome Biology Facility, Duke-NUS Graduate Medical School, Singapore

[9] Genome Institute of Singapore, A*STAR, Singapore

Correspondence: Patrick Tan (gmstanp@duke-nus.edu.sg), Steven Rozen (steve.rozen@duke-nus.edu.sg, +65 9857 3213)

Table S2. One ordered list of genes used as input to GSEA.

Gene	Average relative CN	Correlation coeficient between avg relative CN and expression
FHIT	0.730966716	0.428807973
TYRP1	0.778609875	0.262172988
KLHL9	0.778815541	0.499186139
PTPLAD2	0.778815541	0.222228208
PLAA	0.785472444	0.349004037
UHRF2	0.786522989	0.581443816
CDC37L1	0.786522989	0.455354853
RFX3	0.786522989	0.418717492
KIAA1432	0.786522989	0.364592973
JAK2	0.786522989	0.286530701
AK3	0.786522989	0.283103823
GLIS3	0.786522989	0.254445713
NFIB	0.787802741	0.266960539
CNTLN	0.788296981	0.282079119
IFT74	0.788705342	0.481526796
RANBP6	0.79031892	0.48979162
C9orf123	0.79167536	0.319791579
ADAMTSL1	0.79167536	0.264272791
SMARCA2	0.794009701	0.476319285
KANK1	0.794009701	0.327989209
MLLT3	0.795159551	0.389135469
PSIP1	0.797489848	0.543563372
SNAPC3	0.797489848	0.430862752
RRAGA	0.80620602	0.385406219
DENND4C	0.80620602	0.341175971
RPS6	0.80620602	0.318783674
TET2	0.808678632	0.417944765
INTS12	0.81191153	0.252228322
PPA2	0.81191153	0.247524763
ZDHHC21	0.812020508	0.436624611
HNRNPD	0.813680456	0.305731299
COPS4	0.813680456	0.283439792
SEC31A	0.813680456	0.279256984
NUDT9	0.813680456	0.258460536
HELQ	0.813680456	0.236790571
ARHGAP24	0.813680456	0.232691756
LARP7	0.814528638	0.421643966
CAMK2D	0.814528638	0.382032951
PLA2G12A	0.814528638	0.359590069
RPL34	0.814528638	0.279679298
ALPK1	0.814528638	0.260411345
C4orf32	0.814528638	0.255102999
CASP6	0.814528638	0.255102999
SEC24B	0.814528638	0.240992204
C4orf21	0.814528638	0.224361626
UBE2D3	0.815555739	0.404724406
DNAJB14	0.815555739	0.37581432
ADH1A	0.815555739	0.371665109
ADH6	0.815555739	0.30689039
MANBA	0.815555739	0.289705603
EIF4E	0.815555739	0.252194725
NFKB1	0.815555739	0.234892349
ADH1B	0.815555739	0.234052428
METTL14	0.819372192	0.283576565
SEC24D	0.819372192	0.270708865
MRPL1	0.819936713	0.241645315

Table S2. One ordered list of genes used as input to GSEA.

Gene	Average relative CN	Correlation coeficient between avg relative CN and expression
ELOVL6	0.822015349	0.264275015
GTF2H2C	0.822712809	0.316602543
SERF1B	0.822712809	0.283979731
NAIP	0.822712809	0.261587244
SMN1	0.822712809	0.250013034
SMN2	0.822712809	0.250013034
GALNT7	0.824578618	0.352853845
EXOSC9	0.825686917	0.313645995
BBS7	0.825686917	0.241311377
ANXA5	0.825686917	0.232088965
TOPORS	0.828002391	0.35397637
NDEL1	0.828807297	0.372279247
ANKRD37	0.829122471	0.23507911
SPRY1	0.830945094	0.235331088
PHF17	0.831268384	0.409666589
ACOX3	0.831537531	0.28196665
STX8	0.831926487	0.34650581
KIF1C	0.832299161	0.505159365
SAT2	0.832299161	0.461638356
ZBTB4	0.832299161	0.381027104
RABEP1	0.832299161	0.380842409
ACADVL	0.832299161	0.346992735
MNT	0.832299161	0.331209653
SPAG7	0.832299161	0.312034888
SRR	0.832299161	0.301356122
PER1	0.832299161	0.293246304
SGSM2	0.832299161	0.286647633
PFN1	0.832299161	0.28525402
NEURL4	0.832299161	0.284649562
CHD3	0.832299161	0.277799032
CAMTA2	0.832299161	0.276119981
LSMD1	0.832299161	0.272879412
ZNF232	0.832299161	0.262603618
VAMP2	0.832299161	0.260471223
DHX33	0.832299161	0.247341042
DPH1	0.832299161	0.241581897
PLSCR3	0.832299161	0.239264806
GPS2	0.832299161	0.236208932
GABARAP	0.832299161	0.235083968
TSR1	0.832299161	0.23417728
TMEM88	0.832299161	0.226184996
RPAIN	0.832299161	0.222608617
MED11	0.832299161	0.219351258
ZNF594	0.832299161	0.218847542
RNF167	0.832299161	0.216295384
ING2	0.832510452	0.348402225
BDP1	0.832927733	0.392616046
PTCD2	0.832927733	0.357927011
MAP1B	0.832927733	0.306120239
MRPS27	0.832927733	0.247728429
LCORL	0.83315273	0.219875461
POLR2B	0.833451029	0.320600601
GPM6A	0.834265724	0.294361243
USP16	0.834590354	0.326308607
NTN1	0.835151669	0.21941842
TBC1D14	0.835910837	0.520779764

Table S2. One ordered list of genes used as input to GSEA.

Gene	Average relative CN	Correlation coeficient between avg relative CN and expression
KIAA0232	0.835910837	0.432861358
MRFAP1	0.835910837	0.348669161
GRPEL1	0.835910837	0.304844235
MRFAP1L1	0.835910837	0.214122779
NUP54	0.836772778	0.417461123
SDAD1	0.836772778	0.355222442
THAP6	0.836772778	0.349813304
SLC4A4	0.836772778	0.277629873
RUFY3	0.836772778	0.261822424
SCARB2	0.836772778	0.225789504
RCHY1	0.836772778	0.223740079
UBA6	0.836772778	0.216516696
VCAN	0.837568971	0.260848108
NDUFC1	0.8378222	0.295385956
ELF2	0.8378222	0.243293608
PGRMC2	0.838755095	0.437401019
MFSD8	0.838755095	0.30365085
C2CD2	0.838795511	0.275714837
C17orf85	0.838956064	0.432355702
UBE2G1	0.838956064	0.396306471
ZZEF1	0.838956064	0.393133064
ITGAE	0.838956064	0.374713872
PAFAH1B1	0.838956064	0.33208276
CYB5D2	0.838956064	0.278907206
ANKFY1	0.838956064	0.256710148
ASPA	0.838956064	0.231272522
STK32B	0.839083023	0.264879797
LYSMD3	0.839083644	0.427204291
RASA1	0.839083644	0.392128888
CCNH	0.839083644	0.335534525
ARRDC3	0.839083644	0.303516461
CETN3	0.839083644	0.296024301
FAM172A	0.839083644	0.288599336
COX7C	0.839083644	0.277260305
TMEM161B	0.839083644	0.234625549
SOD1	0.839444713	0.239243675
NXN	0.839898664	0.431319453
RNMTL1	0.839898664	0.362537948
PPID	0.840529209	0.374893561
NAF1	0.840529209	0.266861994
ETFDH	0.840529209	0.256430085
TMEM192	0.840529209	0.24596458
RAPGEF2	0.840529209	0.240975406
PAPD4	0.840889245	0.469402284
NARS	0.840974282	0.437904976
TXNL1	0.840974282	0.415915655
C18orf54	0.840974282	0.314872291
ATP8B1	0.840974282	0.303314878
WDR7	0.840974282	0.279225737
CCDC68	0.840974282	0.247442853
FECH	0.840974282	0.227922921
POLK	0.841117741	0.438660911
ANKRA2	0.841117741	0.423609398
COL4A3BP	0.841117741	0.402493602
HEXB	0.841117741	0.312335707
F2R	0.841117741	0.291270308

Table S2. One ordered list of genes used as input to GSEA.

Gene	Average relative CN	Correlation coeficient between avg relative CN and expression
ENC1	0.841117741	0.289741638
BTF3	0.841117741	0.256530877
UTP15	0.841117741	0.244183932
IQGAP2	0.841117741	0.244150335
NDUFV3	0.841752516	0.426935513
USP25	0.842666456	0.361453929
YTHDC1	0.843000203	0.232357742
SGTB	0.84344497	0.374211127
IPO11	0.84344497	0.366160142
NDUFAF2	0.84344497	0.361924551
PPWD1	0.84344497	0.33429236
RNF180	0.84344497	0.329754227
ERBB2IP	0.84344497	0.321249428
TRIM23	0.84344497	0.302323731
ADAMTS6	0.84344497	0.215342839
RGS7BP	0.84344497	0.214250325
ATP5J	0.843560418	0.298710032
GABPA	0.843560418	0.228301731
TMEM184C	0.844272565	0.35266906
SMARCA5	0.844272565	0.341565209
FBXW7	0.844272565	0.335685712
OTUD4	0.844272565	0.327823984
SMAD1	0.844272565	0.327706394
PLRG1	0.844272565	0.286012357
LRBA	0.844272565	0.28152714
ABCE1	0.844272565	0.279796888
SLC10A7	0.844272565	0.258546704
USP38	0.844272565	0.246367745
RPS3A	0.844272565	0.234894326
ARFIP1	0.844272565	0.233264865
TDO2	0.844272565	0.23192098
LSM6	0.844272565	0.22548713
FAM174A	0.84434182	0.331066107
NUDT12	0.84434182	0.304809951
GIN1	0.84434182	0.27205275
PAM	0.84434182	0.226562238
KIAA1468	0.844937189	0.309765527
CBR4	0.845125642	0.478779912
SH3RF1	0.845125642	0.329590606
CLCN3	0.845125642	0.303871788
ANXA10	0.845125642	0.237063335
LDB2	0.845355584	0.230285754
TTC3	0.84570097	0.308492231
DYRK1A	0.84570097	0.226957099
SMAD4	0.845715866	0.474929012
BRWD1	0.845772666	0.321854513
HMGN1	0.845772666	0.234335768
MYO1C	0.845812674	0.594897918
CRK	0.845812674	0.387445648
PRPF8	0.845812674	0.279221357
INPP5K	0.845812674	0.278482854
SYNJ1	0.845834081	0.226637749
ANKRD17	0.845965644	0.393422378
COX18	0.845965644	0.241966521
FLII	0.846125222	0.350585905
SMCR7	0.846125222	0.285539459

Table S2. One ordered list of genes used as input to GSEA.

Gene	Average relative CN	Correlation coeficient between avg relative CN and expression
ALKBH5	0.846125222	0.258557105
STX18	0.846569734	0.394118894
TMEM128	0.846569734	0.271171792
LYAR	0.846569734	0.21412098
RAB28	0.846895697	0.317617349
MBP	0.846955122	0.23062749
TBCA	0.847031751	0.383141656
LHFPL2	0.847031751	0.296897827
ZBED3	0.847031751	0.283005414
ARSB	0.847031751	0.259991381
AP3B1	0.847031751	0.24900512
AGGF1	0.847031751	0.248265983
WDR41	0.847031751	0.234558354
LRPAP1	0.847102468	0.37291996
HTT	0.847102468	0.223553918
ZSWIM7	0.847195679	0.337119914
CDRT4	0.847195679	0.317558966
TTC19	0.847195679	0.255652346
NCOR1	0.847195679	0.232296743
HS3ST1	0.847329299	0.272281821
STATH	0.847843757	0.232240152
TRIM2	0.848068495	0.297620165
TAF9	0.848288523	0.333183039
RAD17	0.848288523	0.305365961
SLC30A5	0.848288523	0.297886445
ELL2	0.849211073	0.36730061
RFESD	0.849211073	0.33640805
TTC37	0.849211073	0.325556178
MCTP1	0.849211073	0.310991822
ARSK	0.849211073	0.301551029
GLRX	0.849211073	0.23472634
PMP22	0.850367865	0.231591542
JMY	0.850827682	0.294613222
PIK3R1	0.850931681	0.33348839
PJA2	0.850972366	0.375330324
EFNA5	0.850972366	0.37373446
SLC25A46	0.850972366	0.360564386
WDR36	0.850972366	0.358750141
MAN2A1	0.850972366	0.338003914
FER	0.850972366	0.292194229
TSLP	0.850972366	0.273934189
SCFD2	0.851319722	0.354752082
SPATA18	0.851319722	0.235515872
FIP1L1	0.851319722	0.223420906
C18orf25	0.851863018	0.289633765
ATP5A1	0.851863018	0.261060332
GPR125	0.851921498	0.348744812
DHX29	0.852507316	0.540727011
GPBP1	0.852507316	0.480655569
IL6ST	0.852507316	0.431458841
SNX18	0.852507316	0.393187248
GPX8	0.852507316	0.389035696
PPAP2A	0.852507316	0.380967904
GAPT	0.852507316	0.296995626
SLC38A9	0.852507316	0.289196759
NDUFS4	0.852507316	0.239983223

Table S2. One ordered list of genes used as input to GSEA.

Gene	Average relative CN	Correlation coeficient between avg relative CN and expression
GZMK	0.852507316	0.234050033
PLK2	0.852507316	0.229629555
VPS4B	0.852529051	0.31043747
SERPINB3	0.852529051	0.226696626
SERPINB8	0.852529051	0.217927776
SERPINB4	0.852529051	0.216365509
ME2	0.852592972	0.293218941
CNDP2	0.852911939	0.331200495
MPRIP	0.853661475	0.293817182
REEP5	0.853881974	0.468763939
APC	0.853881974	0.374171223
SRP19	0.853881974	0.224092849
SRP72	0.854644609	0.417914684
AASDH	0.854644609	0.397521227
CLOCK	0.854644609	0.34799906
SEC11C	0.854953779	0.364344063
ATP5O	0.855323236	0.31931652
MRPS6	0.855323236	0.299298308
ITSN1	0.855323236	0.240352997
SLC5A3	0.855323236	0.214536059
ZNF236	0.855701733	0.302928511
NOP14	0.855963788	0.372684976
MXD4	0.855963788	0.297171118
TNIP2	0.855963788	0.22001237
WHSC1	0.855963788	0.21470844
PIK3C3	0.856397754	0.340007047
NRIP1	0.856645953	0.234470231
EPN2	0.856882785	0.359115485
ATG12	0.857114872	0.492718692
CEP120	0.857114872	0.46558901
YTHDC2	0.857114872	0.418989793
AP3S1	0.857114872	0.414487777
CDO1	0.857114872	0.35712068
DMXL1	0.857114872	0.355592011
SNX2	0.857114872	0.31522506
DCP2	0.857114872	0.27872178
SNX24	0.857114872	0.276924334
LOX	0.857114872	0.269835339
MCC	0.857114872	0.265467713
SRFBP1	0.857114872	0.251608897
CSNK1G3	0.857114872	0.216684682
KIAA1737	0.857120726	0.318248802
PRMT2	0.858131743	0.291085523
PCNT	0.858131743	0.228863641
RRP1B	0.858407636	0.273598218
COPS3	0.858505028	0.235772379
FLCN	0.858505028	0.225664491
ELAC2	0.8585541	0.309381987
MYOCD	0.8585541	0.236024237
CTDP1	0.85862867	0.35055833
TXNL4A	0.85862867	0.349614664
ADNP2	0.85862867	0.256801815
PQLC1	0.85862867	0.243043675
LNPEP	0.85889818	0.407617164
ERAP1	0.85889818	0.359522875
CAST	0.85889818	0.354063341

Table S2. One ordered list of genes used as input to GSEA.

Gene	Average relative CN	Correlation coeficient between avg relative CN and expression
C21orf91	0.85933113	0.29279365
BTG3	0.85933113	0.231747351
DYM	0.859349729	0.457695976
IER3IP1	0.859349729	0.384749681
HDHD2	0.859349729	0.367437543
CXXC1	0.859349729	0.364328081
MBD1	0.859349729	0.352192776
ACAA2	0.859349729	0.326527109
C18orf32	0.859349729	0.297113281
SMAD2	0.859349729	0.290490968
RBPJ	0.85940821	0.325190061
ANAPC4	0.85940821	0.291675352
STIM2	0.85940821	0.265145673
LETM1	0.860337093	0.361892486
TMEM129	0.860337093	0.355363282
SLBP	0.860337093	0.25557891
UBLCP1	0.860447154	0.508089378
SLU7	0.860447154	0.388181225
EBF1	0.860447154	0.355272838
ITK	0.860447154	0.310891031
TTC1	0.860447154	0.291253509
MED7	0.860447154	0.28445009
THG1L	0.860447154	0.266710807
C1QTNF2	0.860447154	0.249811451
HAVCR2	0.860447154	0.224344827
LSM11	0.860447154	0.219154071
ATG10	0.860810887	0.406911625
TMEM167A	0.860810887	0.267265159
YIPF5	0.861308852	0.430144039
LIAS	0.861330217	0.380151512
UBE2K	0.861330217	0.366796653
RFC1	0.861330217	0.30704416
RPL9	0.861330217	0.274891707
UGDH	0.861330217	0.266442029
FAM114A1	0.861330217	0.256094114
AKAP10	0.862682577	0.476783398
ULK2	0.862682577	0.285186858
SLC47A1	0.862682577	0.248398845
ALDH3A2	0.862682577	0.23251502
MAPK7	0.862682577	0.218075179
NEK9	0.863034736	0.431958284
TMED8	0.863034736	0.330175783
U2AF1	0.863075115	0.224831986
FLRT2	0.86379172	0.2585971
PTTG1IP	0.864036059	0.395236623
PIGG	0.864203735	0.477252954
ATP5I	0.864203735	0.343597286
GAK	0.864203735	0.309860266
PCGF3	0.864203735	0.271977452
GUF1	0.864253391	0.261488653
GNPDA2	0.864253391	0.23635779
PHAX	0.864302911	0.362109854
MAT2B	0.864535763	0.277092319
CCNG1	0.864535763	0.232979289
TMED7	0.864601583	0.49648157
CCDC112	0.864601583	0.489510166

Table S2. One ordered list of genes used as input to GSEA.

Gene	Average relative CN	Correlation coeficient between avg relative CN and expression
FEM1C	0.864601583	0.410338532
PGGT1B	0.864601583	0.352215499
TICAM2	0.864601583	0.286499515
SON	0.8646429	0.377707171
GART	0.8646429	0.260707366
CRYZL1	0.8646429	0.21517476
SPOCK1	0.865016509	0.297216999
FAM13B	0.865016509	0.289926422
HNRNPA0	0.865016509	0.244855874
MYOT	0.865016509	0.241479363
RBM27	0.865104783	0.39164173
LARS	0.865104783	0.336861611
MOCS2	0.865821122	0.378712454
NRXN3	0.866206923	0.334341827
RIOK2	0.866384891	0.49928693
TRAPPC10	0.867268957	0.31031988
UBE2G2	0.867268957	0.289052897
CSTB	0.867268957	0.274286959
SUMO3	0.867268957	0.242319291
TRPM2	0.867268957	0.222765762
ETF1	0.867418748	0.332208409
HSPA9	0.867418748	0.277915449
EGR1	0.867418748	0.273682211
ARIH2	0.8675347	0.458500016
NCKIPSD	0.8675347	0.270641671
SLC26A6	0.8675347	0.265014152
PRKAR2A	0.8675347	0.258294726
IP6K2	0.8675347	0.240505046
UQCRC1	0.8675347	0.236355801
PFKFB4	0.8675347	0.21448407
FNIP1	0.867831243	0.399940221
RAPGEF6	0.867831243	0.321944486
LYRM7	0.867831243	0.287742609
CDC42SE2	0.867831243	0.280216852
CHSY3	0.867831243	0.274018182
HINT1	0.867831243	0.238035657
WDR6	0.868086486	0.431807097
USP19	0.868086486	0.388030038
IMPDH2	0.868086486	0.358094997
USP4	0.868086486	0.301467036
QRICH1	0.868086486	0.296209086
QARS	0.868086486	0.280216852
C3orf62	0.868086486	0.26163764
NICN1	0.868086486	0.243679975
TBC1D1	0.868207323	0.270356095
NR3C1	0.868274581	0.279561708
CTBP1	0.86857704	0.377065753
CRIPAK	0.86857704	0.309205667
MAEA	0.86857704	0.283021711
APBB2	0.869256032	0.442289401
SLC30A9	0.869256032	0.314200348
TMEM33	0.869256032	0.255808538
HSPA13	0.869275404	0.332678801
AFF4	0.869612125	0.284668472
RAD54L2	0.870561943	0.44452361
HEMK1	0.870561943	0.438963286

Table S2. One ordered list of genes used as input to GSEA.

Gene	Average relative CN	Correlation coeficient between avg relative CN and expression
RBM15B	0.870561943	0.285928364
TEX264	0.870561943	0.281930306
C3orf18	0.870561943	0.222144215
PCNX	0.870626598	0.390096262
SIPA1L1	0.870626598	0.274673326
LIN52	0.870626598	0.270624872
ABCD4	0.870626598	0.221052309
PARP8	0.870897418	0.294404729
ISL1	0.870897418	0.222770055
SMU1	0.870977693	0.404690809
PTPN23	0.871026564	0.422819865
DHX30	0.871026564	0.387139714
SCAP	0.871026564	0.383309642
ZNF589	0.871026564	0.355541615
SMARCC1	0.871026564	0.277478686
ATRIP	0.871026564	0.276201995
CDC25A	0.871026564	0.232139361
TMX3	0.871250131	0.308808009
TYMP	0.87268153	0.361336274
ODF3B	0.87268153	0.240890849
TUBGCP6	0.87268153	0.239966415
TUSC2	0.874000496	0.317324881
RASSF1	0.874000496	0.303079698
NAT6	0.874000496	0.275194082
TMEM115	0.874000496	0.240471449
PELO	0.87401113	0.463726106
ITGA1	0.87401113	0.415655911
WDR82	0.874357874	0.372793741
RPL29	0.874357874	0.363117767
BAP1	0.874357874	0.360413199
PCBP4	0.874357874	0.297754553
ABHD14A	0.874357874	0.295419553
ABHD14B	0.874357874	0.219910006
KIAA1644	0.87534558	0.338359872
PARVG	0.87534558	0.336897585
SETD2	0.875591941	0.396143745
CCDC12	0.875591941	0.384636728
KIF9	0.875591941	0.240622636
MYL3	0.875591941	0.219372452
KLHL18	0.875591941	0.216096732
DPYSL3	0.87568493	0.235650261
SOCS6	0.875846466	0.244150335
TTC8	0.876807691	0.214652056
ELP2	0.877245682	0.292944922
FAM161B	0.877267145	0.473181961
ZNF410	0.877267145	0.432680623
ZFYVE1	0.877267145	0.386047808
RBM25	0.877267145	0.37455759
ALDH6A1	0.877267145	0.294277251
PNMA1	0.877267145	0.292160632
NUMB	0.877267145	0.257874762
ACOT1	0.877267145	0.246888501
ACOT2	0.877267145	0.246888501
ENTPD5	0.877267145	0.238774794
RPS6KA5	0.877598412	0.370257157
C14orf159	0.877598412	0.33711359

Table S2. One ordered list of genes used as input to GSEA.

Gene	Average relative CN	Correlation coeficient between avg relative CN and expression
PSMC1	0.877598412	0.331956431
CALM1	0.877598412	0.221304287
PBRM1	0.877849738	0.507971788
GNL3	0.877849738	0.314301139
NEK4	0.877849738	0.258059546
SPCS1	0.877849738	0.255019006
TMEM110	0.877849738	0.249257098
GLT8D1	0.877849738	0.248685947
ITIH4	0.877849738	0.223219323
41890	0.878358736	0.307699304
MON1A	0.878421461	0.359405285
IP6K1	0.878421461	0.357255068
RNF123	0.878421461	0.334761791
CAMKV	0.878421461	0.255774941
FBXO38	0.87878436	0.365839135
ADRB2	0.87878436	0.269062606
SYNJ2BP	0.878816606	0.497623872
VTI1B	0.878816606	0.480472538
KIAA0247	0.878816606	0.290783149
ZFYVE26	0.878816606	0.239026772
COX16	0.878816606	0.216667883
UBE2B	0.879170277	0.401048926
PPP2CA	0.879170277	0.310672649
TXNDC15	0.879170277	0.293504517
SAR1B	0.879170277	0.27474052
PHF15	0.879170277	0.266139655
PCBD2	0.879170277	0.261721633
C5orf24	0.879170277	0.221909035
CAMLG	0.879170277	0.221892237
SLIT3	0.879465488	0.248400371
TIMM44	0.879739258	0.360428647
41700	0.879739258	0.322694908
SNAPC2	0.879739258	0.302441386
NDUFA7	0.879739258	0.277700155
ADAMTS10	0.879739258	0.271447615
MAP2K7	0.879739258	0.252891691
TRAPPC5	0.879739258	0.248790563
KANK3	0.879739258	0.237428422
ELAVL1	0.879739258	0.234772773
FCER2	0.879739258	0.223007242
ZNF397	0.880055246	0.446737224
ZNF24	0.880055246	0.350058172
ZNF396	0.880055246	0.319148441
GALNT1	0.880055246	0.316038979
RNF138	0.880210773	0.234407167
SIL1	0.880654418	0.374288813
CTNNA1	0.880654418	0.220430762
FOXN3	0.880770599	0.429875262
KIAA1143	0.881936313	0.391927305
ZNF35	0.881936313	0.381025037
LIMD1	0.881936313	0.379597159
SACM1L	0.881936313	0.376590216
NKTR	0.881936313	0.362042659
ZNF445	0.881936313	0.306892972
LARS2	0.881936313	0.237313319
TNIP1	0.882011271	0.442087819

Table S2. One ordered list of genes used as input to GSEA.

Gene	Average relative CN	Correlation coeficient between avg relative CN and expression
CCDC69	0.882011271	0.370189963
LARP1	0.882011271	0.254162279
ARHGAP26	0.882650659	0.257387603
FGF1	0.882650659	0.239917096
PCDHB15	0.882831185	0.282383867
PCDHB14	0.882831185	0.265635698
PCDHB4	0.882831185	0.232105764
DUSP1	0.882957352	0.462044513
ATP6V0E1	0.882957352	0.433369364
STK10	0.882957352	0.402728782
BOD1	0.882957352	0.333905064
RPL26L1	0.882957352	0.251138537
DOCK2	0.882957352	0.217071049
ULK4	0.883526836	0.290900739
PPP2R5C	0.883534126	0.488955813
TECPR2	0.883534126	0.475903329
WDR20	0.883534126	0.360295609
EVL	0.883534126	0.297401784
YY1	0.883534126	0.229384396
ZFP36L1	0.883614098	0.259974582
CHD1	0.883970892	0.436040335
C18orf21	0.884589983	0.35664687
PRKD1	0.885031581	0.235751052
NDFIP1	0.885052897	0.420199289
MOAP1	0.885085123	0.444708395
BTBD7	0.885085123	0.415764468
ATXN3	0.885085123	0.411833604
TRIP11	0.885085123	0.395119033
NDUFB1	0.885085123	0.358783738
PRIMA1	0.885085123	0.35325701
FBLN5	0.885085123	0.322045278
UBR7	0.885085123	0.247526846
SMEK1	0.885085123	0.242890442
ASB2	0.885085123	0.220716337
ATG2B	0.885135063	0.336004885
PAPOLA	0.885135063	0.279057751
TCL1A	0.885135063	0.217121444
DICER1	0.885135063	0.21448407
PPP2R5E	0.885200694	0.427338679
UBE2D2	0.885912595	0.37853885
PURA	0.885912595	0.293454121
TMEM173	0.885912595	0.281443147
PAIP2	0.885912595	0.247627637
MATR3	0.885912595	0.236221412
SH3GL1	0.885977483	0.253160618
MPP5	0.886282636	0.248047602
EIF2S1	0.886282636	0.219036481
FUT8	0.886282636	0.216499898
PACSIN2	0.886477746	0.249849461
STXBP6	0.88664267	0.295705129
NGLY1	0.886745393	0.302928511
OXSM	0.886745393	0.239345945
CPEB4	0.886753282	0.305666677
LRFN5	0.887082406	0.241218589
ACTR8	0.887366297	0.384922304
CHDH	0.887366297	0.33617287

Table S2. One ordered list of genes used as input to GSEA.

Gene	Average relative CN	Correlation coeficient between avg relative CN and expression
RPP14	0.887470373	0.379865936
PDHB	0.887470373	0.254767027
ZNF354A	0.887806128	0.410993676
AGXT2L2	0.887806128	0.232139361
TOX4	0.887818901	0.365253235
METTL3	0.887818901	0.272214657
PCYOX1L	0.888267528	0.315006679
HDAC3	0.888745195	0.331150099
TAF7	0.888745195	0.276823542
HECTD1	0.888827512	0.474139479
AP4S1	0.888827512	0.344404167
SCFD1	0.888827512	0.325186609
COCH	0.888827512	0.229686771
STRN3	0.888827512	0.218129358
SLC25A23	0.889222426	0.511346798
RANBP3	0.889222426	0.467730293
C19orf70	0.889222426	0.456149649
RPL36	0.889222426	0.425811386
GTF2F1	0.889222426	0.411507861
SH2D3A	0.889222426	0.411339782
ALKBH7	0.889222426	0.386531319
NDUFA11	0.889222426	0.385455613
GPR108	0.889222426	0.380614938
CLPP	0.889222426	0.368681327
ARHGEF18	0.889222426	0.362899409
MLLT1	0.889222426	0.330561006
PNPLA6	0.889222426	0.279128826
CRB3	0.889222426	0.275884901
LONP1	0.889222426	0.245765141
FUT3	0.889222426	0.23912602
FUT5	0.889222426	0.216317697
FAM114A2	0.890201279	0.365435969
GM2A	0.890201279	0.359422083
SAP30L	0.890201279	0.309311966
G3BP1	0.890201279	0.278536996
GALNT10	0.890201279	0.236473391
ZNF621	0.891118698	0.220665942
GABRA2	0.89114554	0.276691482
CYB5R3	0.891286826	0.256286888
RHOJ	0.89142812	0.268340267
RPL15	0.891588946	0.39866353
PPARGC1B	0.89162943	0.387324499
CD74	0.89162943	0.267819512
TIGD6	0.89162943	0.2351967
RBM22	0.89162943	0.214349681
HSPA2	0.891849379	0.304725958
ZBTB1	0.891849379	0.229401195
PDE12	0.891901033	0.416369216
APPL1	0.891901033	0.375783885
CCDC66	0.891901033	0.277058722
DNASE1L3	0.891901033	0.245007061
TMED9	0.892179433	0.343009886
RAB24	0.892179433	0.329537437
PDLIM7	0.892179433	0.301198259
ZNF354B	0.892179433	0.288095379
DDX41	0.892179433	0.262595158

Table S2. One ordered list of genes used as input to GSEA.

Gene	Average relative CN	Correlation coeficient between avg relative CN and expression
KCTD6	0.892313926	0.342069166
CCNB1IP1	0.892627981	0.300037238
APEX1	0.892627981	0.299130528
TTC5	0.892627981	0.259671828
OSGEP	0.892627981	0.259201682
TMEM55B	0.892627981	0.257606543
NDRG2	0.892662454	0.355951056
EEF2	0.89273424	0.425524434
ZBTB7A	0.89273424	0.399419465
MRPL54	0.89273424	0.373650467
CCDC94	0.89273424	0.228090907
PPP1R13B	0.892808339	0.406760438
C14orf2	0.892808339	0.28468527
ACTN1	0.893312792	0.276185197
DDX24	0.893325071	0.483160308
CHMP4A	0.893365811	0.586706659
TINF2	0.893365811	0.489073403
DHRS4	0.893365811	0.361857875
DHRS4L2	0.893365811	0.361857875
GMPR2	0.893365811	0.305599483
TM9SF1	0.893365811	0.265098144
NEDD8	0.893365811	0.243646378
PSME1	0.893365811	0.23472634
DHRS1	0.893365811	0.22758695
NFATC4	0.893365811	0.219036481
CANX	0.893595733	0.369652409
MAML1	0.893595733	0.334341827
SQSTM1	0.893595733	0.323489954
LTC4S	0.893595733	0.287221854
EIF3G	0.893718755	0.436770138
ZNF562	0.893718755	0.390582023
MRPL4	0.893718755	0.385169879
UBL5	0.893718755	0.366395453
CDC37	0.893718755	0.353167634
ZNF561	0.893718755	0.34758741
ZNF846	0.893718755	0.322829371
PIN1	0.893718755	0.283767807
ZNF317	0.893718755	0.26235454
TYK2	0.893718755	0.262186461
ICAM3	0.893718755	0.260169513
KEAP1	0.893718755	0.249630958
ZNF266	0.893718755	0.235260202
OCIAD1	0.894378438	0.411081128
COMMD8	0.894378438	0.38833568
FRYL	0.894378438	0.353394372
NFXL1	0.894378438	0.312304738
ATP10D	0.894378438	0.246722591
TIMP3	0.894518573	0.315796212
ANKHD1	0.894659206	0.358918126
ANKHD1-EIF4	0.894659206	0.356566327
PFDN1	0.894659206	0.34658798
EIF4EBP3	0.894659206	0.28948966
HARS	0.894659206	0.277109118
ZMAT2	0.894659206	0.236406196
EML1	0.894764192	0.359657263
FAF2	0.894818886	0.315040276

Table S2. One ordered list of genes used as input to GSEA.

Gene	Average relative CN	Correlation coeficient between avg relative CN and expression
RASD2	0.895205877	0.316182579
HMOX1	0.895205877	0.282921421
ARHGAP5	0.895926961	0.314721103
CHURC1	0.896243456	0.505334413
MAX	0.896243456	0.246031774
GPX2	0.896243456	0.222832956
LGALS1	0.896348216	0.394379896
CSF2RB	0.896348216	0.356482335
NCF4	0.896348216	0.325976142
CYTH4	0.896348216	0.265837281
CLK4	0.896552738	0.337768734
UIMC1	0.896552738	0.317425672
TBC1D9B	0.896552738	0.287053868
HNRNPAB	0.896552738	0.24092501
RUFY1	0.896552738	0.231853785
WASF2	0.896570933	0.242746564
CECR1	0.896790467	0.318937543
RNASE4	0.897471534	0.353079806
ANG	0.897471534	0.332611655
PITPNB	0.897718417	0.538897945
HSCB	0.897718417	0.413782238
MN1	0.897718417	0.365368775
ZNRF3	0.897718417	0.245107853
TPST2	0.897718417	0.226142274
APOBEC3C	0.898043583	0.390281046
ST13	0.898043583	0.302794123
EP300	0.898043583	0.300677504
CBX7	0.898043583	0.27778106
SYNGR1	0.898043583	0.272254333
CBX6	0.898043583	0.265081346
APOBEC3G	0.898043583	0.257152423
POLR3H	0.898043583	0.243713572
XPNPEP3	0.898043583	0.22091792
APOBEC3F	0.898043583	0.217239034
RCOR1	0.898064785	0.236910153
B4GALT6	0.898128047	0.301500633
TTR	0.898128047	0.24151296
DSG2	0.898128047	0.214517667
FBXO7	0.898141499	0.271783973
ALDH1B1	0.899076494	0.296139399
CNTNAP3	0.899076494	0.253706583
ZNF658	0.899076494	0.230541557
PIP5K1C	0.899490996	0.438327389
NFIC	0.899490996	0.385150494
DOHH	0.899490996	0.240093596
RPL14	0.899865309	0.508492544
UBP1	0.899865309	0.425625225
STT3B	0.899865309	0.339717367
EIF1B	0.899865309	0.324817041
OSBPL10	0.899865309	0.305901857
ZNF619	0.899865309	0.266895591
CMTM8	0.899865309	0.220262776
ATP6V1E1	0.90001284	0.376942986
THTPA	0.900122568	0.371278059
EFS	0.900122568	0.353060047
PABPN1	0.900122568	0.33116485

Table S2. One ordered list of genes used as input to GSEA.

Gene	Average relative CN	Correlation coeficient between avg relative CN and expression
ELOF1	0.900359302	0.459881003
ZNF700	0.900359302	0.314912849
KANK2	0.900359302	0.308122457
CARM1	0.900359302	0.303987713
ECSIT	0.900359302	0.302340539
ZNF441	0.900359302	0.30008828
DNM2	0.900359302	0.274842811
ZNF653	0.900359302	0.264388296
QTRT1	0.900359302	0.240134494
TRIM52	0.900983399	0.470426997
GFPT2	0.900983399	0.459726311
MGAT1	0.900983399	0.407717956
DSC2	0.901227476	0.25824433
NEFH	0.901461772	0.332510783
MARK3	0.902495446	0.39023065
CKB	0.902495446	0.360026832
KLC1	0.902495446	0.320197435
BAG5	0.902495446	0.31312524
EIF5	0.902495446	0.218868495
COX4I1	0.902522427	0.416947385
FBXO31	0.902522427	0.349281629
FOXF1	0.902522427	0.26125567
MAP1LC3B	0.902522427	0.240912266
ZCCHC14	0.902522427	0.214017312
APOL3	0.902692588	0.401620077
APOL2	0.902692588	0.265938073
APOL1	0.902692588	0.254363862
NFX1	0.903306673	0.525992221
CHMP5	0.903306673	0.268489195
BAG1	0.903306673	0.25788939
KIAA1191	0.903689901	0.451108648
C22orf39	0.903932941	0.404324646
MRPL40	0.903932941	0.227284576
KLHL28	0.904369699	0.504063904
FAM179B	0.904369699	0.489422208
C14orf28	0.904369699	0.44067812
LRRFIP2	0.904525141	0.409481805
SLC25A38	0.904525141	0.397622019
PLCD1	0.904525141	0.344387368
GORASP1	0.904525141	0.337785532
GOLGA4	0.904525141	0.332527582
WDR48	0.904525141	0.237951664
CTDSPL	0.904525141	0.22068274
SMTN	0.904782046	0.306170634
TCN2	0.904782046	0.2983761
PISD	0.904782046	0.270003325
RNF185	0.904782046	0.268222677
EIF4ENIF1	0.904782046	0.241731341
PIK3IP1	0.904782046	0.234440764
MAPK9	0.905414059	0.395824572
CIRBP	0.906247753	0.343438636
ATP5D	0.906247753	0.336040051
SF3A2	0.906247753	0.330856008
C19orf21	0.906247753	0.284434499
BTBD2	0.906247753	0.279116242
CFD	0.906247753	0.277572773

Table S2. One ordered list of genes used as input to GSEA.

Gene	Average relative CN	Correlation coeficient between avg relative CN and expression
MKNK2	0.906247753	0.274620049
GNG7	0.906247753	0.260913373
REXO1	0.906247753	0.246049095
NUDT7	0.906699958	0.233434815
BCL2L13	0.906889946	0.293538114
KLHL36	0.907654081	0.258013493
RPSA	0.908898446	0.420333678
ACVR2B	0.908898446	0.337382367
XYLB	0.908898446	0.266509224
ACAA1	0.908898446	0.260041776
OXSR1	0.908898446	0.234743139
MAGI1	0.909189391	0.322700422
SUCLG2	0.909189391	0.308841606
TMF1	0.909189391	0.286381925
LRIG1	0.909189391	0.27989768
C19orf43	0.90984247	0.365790368
PRDX2	0.90984247	0.350226251
ZNF791	0.90984247	0.32708177
ZNF564	0.90984247	0.319148441
RAD23A	0.90984247	0.301752262
TRMT1	0.90984247	0.293667661
NACC1	0.90984247	0.291617097
HOOK2	0.90984247	0.260102281
IER2	0.90984247	0.247714858
ZNF563	0.90984247	0.242857374
ZBTB40	0.909965224	0.285311587
C15orf52	0.910395087	0.345903706
C15orf57	0.910395087	0.217425187
RAB5A	0.910534435	0.332594776
KCNH8	0.910534435	0.2761516
ZFYVE16	0.91061499	0.426969111
ZCCHC9	0.91061499	0.350250067
MSH3	0.91061499	0.276588362
ACOT12	0.91061499	0.242957636
APRT	0.910826086	0.319060503
TRAF3	0.911356766	0.247930012
ABHD4	0.911885102	0.28894667
DAD1	0.911885102	0.230167045
TJP1	0.911979625	0.410327851
HERC2	0.911979625	0.28299226
GRHPR	0.912008086	0.537650327
ZCCHC7	0.912008086	0.434692792
ZBTB5	0.912008086	0.368221432
POLR1E	0.912008086	0.257301445
EXOSC3	0.912008086	0.249775752
TOMM5	0.912008086	0.239226342
SHB	0.912008086	0.216229301
PSKH1	0.912174259	0.22259965
ZDHHC7	0.91246316	0.24173541
KIAA0513	0.91246316	0.218284219
GNA11	0.91300451	0.323365657
ZNF57	0.91300451	0.26243464
AES	0.91300451	0.216941942
GKAP1	0.913132033	0.418015476
C9orf64	0.913132033	0.342623519
FAM98B	0.913352092	0.400949121

Table S2. One ordered list of genes used as input to GSEA.

Gene	Average relative CN	Correlation coeficient between avg relative CN and expression
SLC30A4	0.913352092	0.330776722
SRP14	0.913352092	0.327314323
VPS39	0.913352092	0.319717216
CAPN3	0.913352092	0.234081677
EIF2AK4	0.913352092	0.233846369
SERF2	0.913352092	0.227341765
TTBK2	0.913352092	0.224047444
AQR	0.913760508	0.246082418
ZNF770	0.913760508	0.219089155
RPL11	0.913761155	0.232201856
GLG1	0.914889967	0.312791901
ZFHX3	0.914889967	0.270946325
RAD23B	0.91511324	0.346265894
KLF4	0.91511324	0.323487232
SLC44A1	0.91511324	0.3184981
IKBKAP	0.91511324	0.283506985
NIPSNAP3A	0.91511324	0.26875797
TPPP3	0.915131264	0.226127378
RPL13	0.915669639	0.446126982
SPG7	0.915669639	0.28457247
SHQ1	0.91700067	0.401552883
PROK2	0.91700067	0.282316673
RPAP1	0.917125189	0.265848344
NDUFAF1	0.917125189	0.22761069
TMEM39B	0.917283677	0.295839517
ZNF510	0.91810355	0.314600866
CDC14B	0.91810355	0.290075169
SLC35D2	0.91810355	0.254580101
PTCH1	0.91810355	0.247457569
HABP4	0.91810355	0.230591953
KLF13	0.918945354	0.268940972
ZCCHC6	0.918956823	0.427221089
ISCA1	0.918956823	0.319693479
UGCG	0.919234228	0.301094934
HSDL2	0.919234228	0.26257615
SLC35E2	0.919430006	0.384603131
MIB2	0.919430006	0.330142186
MMP23B	0.919430006	0.314956283
GNB1	0.919430006	0.219624431
MRPL20	0.919430006	0.216298315
SNTB2	0.919699046	0.314975732
DDX19A	0.91973352	0.227454476
DDX19B	0.91973352	0.216098551
OXA1L	0.919810916	0.408587208
LRP10	0.919810916	0.398583594
MRPL52	0.919810916	0.226289806
PNRC2	0.919903661	0.229364188
INO80	0.920082194	0.257511695
VPS18	0.920082194	0.241006474
TLE1	0.920618744	0.3348654
DYNC1LI2	0.921045275	0.279597658
TUBGCP5	0.921724594	0.227778767
CYFIP1	0.921724594	0.217273917
RNF20	0.921836381	0.308603829
ZNF189	0.921836381	0.257486228
FTSJD1	0.921918795	0.278656931

Table S2. One ordered list of genes used as input to GSEA.

Gene	Average relative CN	Correlation coeficient between avg relative CN and expression
MTSS1L	0.922966418	0.287073082
FBXO33	0.922967441	0.463379466
MAPKBP1	0.923039199	0.36778741
C3orf38	0.924773189	0.362395429
ERP44	0.925069279	0.514166132
ANP32B	0.925069279	0.434138444
STX17	0.925069279	0.41267006
SEC61B	0.925069279	0.412384487
ALG2	0.925069279	0.35461471
NANS	0.925069279	0.353573208
PTPDC1	0.925069279	0.343712533
XPA	0.925069279	0.331668064
GALNT12	0.925069279	0.298776752
TEX10	0.925069279	0.289621611
NR4A3	0.925069279	0.288882481
HIATL1	0.925069279	0.267598879
ANKS6	0.925069279	0.256999074
INVS	0.925069279	0.244534644
TDRD7	0.925069279	0.217791554
THOC7	0.925475609	0.34331226
DNAJC25	0.926199956	0.441479355
ALAD	0.926199956	0.437010975
CDC26	0.926199956	0.407160177
GNG10	0.926199956	0.371429931
SLC31A1	0.926199956	0.345425972
C9orf43	0.926199956	0.291906197
PTGR1	0.926199956	0.283221412
LPAR1	0.926199956	0.269597891
BSPRY	0.926199956	0.246987214
SLC31A2	0.926199956	0.22358701
ARHGEF10L	0.926256631	0.268654979
AGTPBP1	0.926443534	0.375820645
LSM3	0.927501257	0.354029744
CHCHD4	0.927501257	0.342690713
ZBTB48	0.927637983	0.224563209
ACTC1	0.927691965	0.262100214
GOLGA8A	0.927691965	0.230820972
AMFR	0.927769593	0.297149805
UBE3A	0.928690323	0.300724447
ATP10A	0.928690323	0.241846862
MAFG	0.928863483	0.279514015
RNF213	0.928863483	0.237520589
ATL1	0.929634971	0.288753729
SAV1	0.929634971	0.266119547
FBXO42	0.929695184	0.287108218
ZNF280D	0.930792938	0.384914518
PYGO1	0.930792938	0.264184376
NEDD4	0.930792938	0.256419191
TCF12	0.930792938	0.217576457
GMDS	0.930965375	0.215734113
RERE	0.931433914	0.349426937
ILVBL	0.932751016	0.21404863
PSMD6	0.93281991	0.289775235
TATDN2	0.933263147	0.401452091
SEC13	0.933263147	0.271280016
OXNAD1	0.933845629	0.436611487

Table S2. One ordered list of genes used as input to GSEA.

Gene	Average relative CN	Correlation coeficient between avg relative CN and expression
MRPS25	0.933845629	0.408238711
CAPN7	0.933845629	0.35115719
HACL1	0.933845629	0.346453592
BTD	0.933845629	0.273043865
ZFYVE20	0.933845629	0.261301669
EAF1	0.933845629	0.2574212
NR2C2	0.933845629	0.2457294
DPH3	0.933845629	0.237750082
SETMAR	0.933918386	0.391104176
THUMPD3	0.933918386	0.355642406
RAD18	0.933918386	0.259470625
SUMF1	0.933918386	0.257790769
ARL8B	0.933918386	0.220901121
CAV3	0.933918386	0.217910977
ARID4A	0.934445509	0.425171664
ACTR10	0.934445509	0.395639788
TIMM9	0.934445509	0.361185933
MUL1	0.934504263	0.29845889
HP1BP3	0.934504263	0.26239196
DMXL2	0.934525517	0.257747004
TMOD2	0.934525517	0.253477833
ADRBK2	0.935255954	0.2796625
LRP5L	0.935255954	0.222345798
ZRANB2	0.935289417	0.251227709
TPM1	0.93545277	0.369955611
SLTM	0.93545277	0.354492472
RNF111	0.93545277	0.277697815
RAB8B	0.93545277	0.275882577
MYO1E	0.93545277	0.221274164
FAM63B	0.93545277	0.219912735
OSTF1	0.93549861	0.295825208
C9orf41	0.93549861	0.25036791
RBL2	0.935914959	0.42650234
AKTIP	0.935914959	0.228613586
FTO	0.935914959	0.21403231
RPL32	0.936362577	0.482874733
RAF1	0.936362577	0.455106706
MKRN2	0.936362577	0.349662117
TSEN2	0.936362577	0.345210498
ATG7	0.936362577	0.245242241
GGT5	0.93710159	0.325707365
SLC2A11	0.93710159	0.29545315
ZNF70	0.93710159	0.268995411
ZNF280B	0.93710159	0.264862964
SUSD2	0.93710159	0.246401342
KLHDC1	0.937560785	0.483830826
KLHDC2	0.937560785	0.430788258
RPS29	0.937560785	0.367771875
RPL36AL	0.937560785	0.263466579
SOS2	0.937560785	0.245953633
RPUSD3	0.937693808	0.462750053
BRPF1	0.937693808	0.38169698
MTMR14	0.937693808	0.373919244
JAGN1	0.937693808	0.331973229
ARPC4	0.937693808	0.286919479
OGG1	0.937693808	0.283072608

Table S2. One ordered list of genes used as input to GSEA.

Gene	Average relative CN	Correlation coeficient between avg relative CN and expression
IL17RC	0.937693808	0.265770087
SGSH	0.938550589	0.248434514
DAAM1	0.938980245	0.525190316
PPM1A	0.938980245	0.484621784
LPCAT2	0.939658314	0.237867671
BEND5	0.940031001	0.256236464
CACHD1	0.94013297	0.265144652
PGM1	0.94013297	0.257060051
JAK1	0.94013297	0.253748894
TIMP2	0.940752204	0.457528527
USP36	0.940752204	0.382222444
CYTH1	0.940752204	0.250449393
SOCS3	0.940752204	0.243313365
RNASET2	0.940779743	0.327838126
MYEOV2	0.940823065	0.312795054
DUSP28	0.940823065	0.227881533
COPS2	0.941215375	0.408478998
USP8	0.941215375	0.355400091
SECISBP2L	0.941215375	0.295278732
GLDN	0.941215375	0.224938256
TRPM7	0.941215375	0.215542718
UBA52	0.94121898	0.383435435
SLC35E1	0.94121898	0.377838451
ARRDC2	0.94121898	0.371216193
MED26	0.94121898	0.370543883
SLC27A1	0.94121898	0.367989103
TPM4	0.94121898	0.349584606
PLVAP	0.94121898	0.34482801
TMEM161A	0.94121898	0.326658821
SIN3B	0.94121898	0.317952401
JUND	0.94121898	0.301262295
MAP1S	0.94121898	0.300489138
KLHL26	0.94121898	0.286992507
RAB8A	0.94121898	0.28606808
RAB3A	0.94121898	0.282624858
FAM32A	0.94121898	0.272806757
C19orf60	0.94121898	0.259915205
WIZ	0.94121898	0.252116404
GATAD2A	0.94121898	0.247208538
CHERP	0.94121898	0.243275522
UPF1	0.94121898	0.242048556
EPS15L1	0.94121898	0.234569102
PDE4C	0.94121898	0.233224481
RPL18A	0.94121898	0.221257356
COPE	0.94121898	0.21927404
RFXANK	0.94121898	0.218870654
PGLS	0.94121898	0.216937762
TRNT1	0.941405097	0.345395282
CRBN	0.941405097	0.257689977
ATP6V1G1	0.942406265	0.398341005
ASTN2	0.942406265	0.311593948
AKNA	0.942406265	0.298911139
COL27A1	0.942406265	0.23359887
MAP3K4	0.942516382	0.464179915
TAF4B	0.942906571	0.351257981
ATP1A1	0.943453244	0.310391524

Table S2. One ordered list of genes used as input to GSEA.

Gene	Average relative CN	Correlation coeficient between avg relative CN and expression
CSDE1	0.943453244	0.22406614
FGFR1OP	0.943736748	0.343318203
SEC23A	0.943864627	0.400312709
TRAPPC6B	0.943864627	0.388861291
PNN	0.943864627	0.375630033
CTAGE5	0.943864627	0.232907076
PHF2	0.944237383	0.298625566
SSBP3	0.944874554	0.333485581
ZCCHC11	0.944874554	0.250757088
RNF11	0.944874554	0.221141565
C1orf123	0.944874554	0.215157952
MIPOL1	0.945192703	0.253572195
GSTM3	0.945488379	0.328073436
AMIGO1	0.945488379	0.285381366
AHCYL1	0.945488379	0.268321345
C1orf194	0.945488379	0.268052419
CYB561D1	0.945488379	0.26748095
TMEM167B	0.945488379	0.244000311
KCNC4	0.945488379	0.240100878
GSTM4	0.945488379	0.223225745
GNAQ	0.945533397	0.477469613
VPS13A	0.945533397	0.428064619
PER2	0.945795581	0.298993247
TRAF3IP1	0.945795581	0.240939243
DDHD1	0.945874895	0.433621342
KTN1	0.945874895	0.415294108
C14orf166	0.945874895	0.390180255
FERMT2	0.945874895	0.301685418
PTGER2	0.945874895	0.29557074
STYX	0.945874895	0.286969875
MAPK1IP1L	0.945874895	0.273329441
FBXO34	0.945874895	0.268054692
SOCS4	0.945874895	0.240286665
PELI2	0.945874895	0.216348711
TXNDC16	0.945874895	0.215911948
TGFBR3	0.946100654	0.285919219
ACADM	0.946100654	0.28117939
SH3GLB1	0.946100654	0.277061454
USP33	0.946100654	0.26811965
ARHGAP29	0.946170597	0.293499582
FANCD2	0.946440418	0.399688242
CRELD1	0.946440418	0.299921568
PHKB	0.946925474	0.444943574
ITFG1	0.946925474	0.437367422
LONP2	0.946925474	0.329772617
NDUFB7	0.94721398	0.437358414
C19orf53	0.94721398	0.408869021
MRI1	0.94721398	0.406784841
CCDC130	0.94721398	0.397422839
CD97	0.94721398	0.373152229
CC2D1A	0.94721398	0.317787
GIPC1	0.94721398	0.296155231
UBE4B	0.94784585	0.235011916
PI4KA	0.948170839	0.407398783
UBE2F	0.948908986	0.307903954
SH3BP4	0.949031652	0.340340338

Table S2. One ordered list of genes used as input to GSEA.

Gene	Average relative CN	Correlation coeficient between avg relative CN and expression
SNX7	0.949231735	0.22221727
GDAP2	0.949278034	0.2288732
SNX1	0.949384227	0.291933988
SMC5	0.949493419	0.359811478
PIP5K1B	0.949493419	0.351244138
C9orf85	0.949493419	0.328196314
TJP2	0.949493419	0.315311707
FAM122A	0.949493419	0.237164128
FXN	0.949493419	0.236525777
TMEM2	0.949493419	0.229907087
AKIRIN1	0.94978805	0.403624955
OVGP1	0.94991904	0.237781388
ATP5F1	0.94991904	0.225040998
MOV10	0.94991904	0.221948344
CDK5RAP2	0.950314774	0.258981288
RFK	0.95037695	0.356199756
GCNT1	0.95037695	0.249830352
JMJD6	0.950439311	0.377319573
MXRA7	0.950439311	0.338868976
GAA	0.950439311	0.303575022
EIF4A3	0.950439311	0.28067257
CLN8	0.950505865	0.333053943
KBTBD11	0.950505865	0.262834762
MYOM2	0.950505865	0.222299144
HYI	0.950526856	0.291717945
CLCN6	0.950774151	0.341800389
MTHFR	0.950774151	0.245914184
TMEM181	0.951709249	0.49130764
WTAP	0.951709249	0.418698116
ARID1B	0.951709249	0.408478998
MRPL18	0.951709249	0.351315805
TCP1	0.951709249	0.313145382
ACAT2	0.951709249	0.290975946
DYNLT1	0.951709249	0.257108309
SLC22A18	0.951847213	0.27997158
PPM1F	0.951910267	0.319340709
MAPK1	0.951910267	0.309429556
SECISBP2	0.952262307	0.413392392
SEMA4D	0.952262307	0.375797521
GADD45G	0.952262307	0.32809
41884	0.952788232	0.343956904
MTERFD2	0.952788232	0.28022134
CGRRF1	0.953361606	0.445850697
CNIH	0.953361606	0.339616576
ROCK1	0.953689378	0.347116789
RIMKLA	0.954322786	0.363084296
FBXW2	0.95468808	0.443209593
PSMD5	0.95468808	0.284800464
ERICH1	0.95487917	0.530373201
VHL	0.955301739	0.344756937
SLC43A3	0.955981737	0.239159636
TULP4	0.956082554	0.524318081
AKAP8	0.956151039	0.445170338
BRD4	0.956151039	0.392511625
OSBPL5	0.956220518	0.225345439
HECA	0.956564987	0.337079632

Table S2. One ordered list of genes used as input to GSEA.

Gene	Average relative CN	Correlation coeficient between avg relative CN and expression
ZFP91	0.956627714	0.319299712
ROCK2	0.956977716	0.421239636
KCNF1	0.956977716	0.214075637
ALDH1A1	0.95708528	0.225858599
TBP	0.957753635	0.311652116
C6orf120	0.957753635	0.264774878
FAM120B	0.957753635	0.256337311
C6orf70	0.957753635	0.223242552
FOXA1	0.957822155	0.2684388
VAMP1	0.957949393	0.234270807
MRPL51	0.957949393	0.232288593
TAPBPL	0.957949393	0.218984242
DMAP1	0.958013567	0.311253958
CCDC24	0.958013567	0.252204692
IRAK1BP1	0.958571755	0.502383954
PHIP	0.958571755	0.286303388
HMGN3	0.958571755	0.233342136
FAM120AOS	0.958613462	0.480972448
FAM120A	0.958613462	0.319270828
FGD3	0.958613462	0.263718443
C9orf89	0.958613462	0.249355791
COX7A2	0.958659871	0.261343864
SRP68	0.958679258	0.380257938
GRB2	0.958679258	0.374515534
SAP30BP	0.958679258	0.352049639
EXOC7	0.958679258	0.309602866
TRIM47	0.958679258	0.280202431
LZTR1	0.958787374	0.323271573
UBE2L3	0.958787374	0.314267542
MCPH1	0.959634123	0.58093773
AGPAT5	0.959634123	0.330802897
IARS	0.960075878	0.263382474
IPPK	0.960075878	0.228928909
ZNF195	0.960593823	0.424823298
PFKM	0.960608913	0.360274341
FMNL3	0.960608913	0.327751325
DIP2B	0.960608913	0.315195928
ZNF641	0.960608913	0.284656228
TUBA1A	0.960608913	0.264352453
LMBR1L	0.960608913	0.221223741
ASB8	0.960608913	0.216383106
PDE6D	0.960750375	0.220299314
MBIP	0.960772721	0.405984287
FBXO25	0.961106596	0.525199156
ZNF596	0.961106596	0.385886317
MUTYH	0.961809497	0.289821093
IPP	0.961809497	0.273433251
BEST4	0.961809497	0.261634004
TOE1	0.961809497	0.256709248
GPBP1L1	0.961809497	0.217630547
TNKS1BP1	0.961895747	0.307819915
ZDHHC5	0.961895747	0.273834337
YPEL4	0.961895747	0.231142266
NDUFA8	0.962279941	0.379459577
RAB14	0.962279941	0.379392384
PTGS1	0.962279941	0.34310779

Table S2. One ordered list of genes used as input to GSEA.

Gene	Average relative CN	Correlation coeficient between avg relative CN and expression
PDCL	0.962279941	0.324075177
RBM18	0.962279941	0.321874583
RC3H2	0.962279941	0.285825168
TTLL11	0.962279941	0.281625562
ZBTB6	0.962279941	0.255218441
USP32	0.962359785	0.473147813
RPS6KB1	0.962359785	0.404087273
HEATR6	0.962359785	0.342515427
PTRH2	0.962359785	0.282085356
RNFT1	0.962359785	0.221050816
PPP6C	0.962401364	0.525320284
RPL35	0.962401364	0.267430894
MAPKAP1	0.962401364	0.256125556
HSPA5	0.962401364	0.231230293
GAPVD1	0.962401364	0.229332071
GOLGA1	0.962401364	0.228643336
SCN3A	0.962761047	0.227143899
RHOC	0.96321102	0.223309784
SH3BGRL2	0.963415308	0.246233688
TTK	0.963415308	0.228770425
BCKDHB	0.963415308	0.222383476
ING4	0.963863403	0.355017872
SCNN1A	0.963863403	0.326897312
ATF1	0.963929186	0.325936087
BIN2	0.963929186	0.280218979
POU6F1	0.963929186	0.271294058
TFCP2	0.963929186	0.232686633
CSRNP2	0.963929186	0.224131483
AIG1	0.964051698	0.306287815
LTV1	0.964051698	0.298236898
PHACTR2	0.964051698	0.297631819
VTA1	0.964051698	0.282857798
TSG101	0.964312679	0.301183331
USH1C	0.964312679	0.255431836
UEVLD	0.964312679	0.242590491
HPS5	0.964312679	0.216336327
MTO1	0.964485822	0.356475787
RNGTT	0.964875326	0.365854518
CGA	0.964875326	0.314977427
GABRR1	0.964875326	0.293429879
RARS2	0.964875326	0.28253845
SNX14	0.964875326	0.280101325
PGM3	0.964875326	0.256469614
NUP43	0.964907964	0.29154741
KATNA1	0.964907964	0.291026369
PCMT1	0.964907964	0.262150637
C6orf211	0.964907964	0.246872383
PPIL4	0.964907964	0.236165839
CNKSR3	0.964907964	0.228602347
PPP1R14C	0.964907964	0.215737816
ULBP3	0.964907964	0.215693987
MYO6	0.964916127	0.282051025
SENP6	0.964916127	0.224585293
COX11	0.965032039	0.32313885
MMD	0.965032039	0.301193281
STXBP4	0.965032039	0.290934105

Table S2. One ordered list of genes used as input to GSEA.

Gene	Average relative CN	Correlation coeficient between avg relative CN and expression
PCTP	0.965032039	0.289087118
HLF	0.965032039	0.244893745
FASTK	0.965394756	0.391849218
TMUB1	0.965394756	0.34061787
ZNF775	0.965394756	0.336836029
CUL1	0.965394756	0.321557388
ZNF212	0.965394756	0.288344415
ZNF398	0.965394756	0.269720946
ABCF2	0.965394756	0.257854367
ZNF282	0.965394756	0.215581782
SOX6	0.965629626	0.392182078
C11orf58	0.965629626	0.387795179
RPS13	0.965629626	0.320327692
PIK3C2A	0.965629626	0.271853295
NUCB2	0.965629626	0.216437176
SNAP29	0.965630159	0.227670943
SNRNP48	0.965686717	0.606611769
RIOK1	0.965686717	0.560017754
EEF1E1	0.965686717	0.557399359
SSR1	0.965686717	0.495178894
DSP	0.965686717	0.494356449
TXNDC5	0.965686717	0.335725315
RREB1	0.965686717	0.24053992
NAT9	0.965733609	0.219537799
ANXA2	0.965968115	0.248586774
SNF8	0.96600298	0.381512195
NFE2L1	0.96600298	0.314250744
MRPL10	0.96600298	0.296444265
KPNB1	0.96600298	0.282955018
CBX1	0.96600298	0.277680269
HOXB4	0.96600298	0.241210586
RNF32	0.966010809	0.235112893
TUBB2B	0.966117079	0.319595326
NPTN	0.966383853	0.394108363
UACA	0.966383853	0.361904694
MYO9A	0.966383853	0.356543019
ARIH1	0.966383853	0.325045276
AUH	0.966541674	0.308788612
SYK	0.966541674	0.255084053
CREBL2	0.96656021	0.399063337
CDKN1B	0.96656021	0.246298478
APOLD1	0.96656021	0.241746106
RXRA	0.966631688	0.293955604
PRKAR1A	0.966715948	0.399113842
AMZ2	0.966715948	0.337374608
ABCA9	0.966715948	0.235556083
PTEN	0.966992255	0.540852361
PCGF5	0.966992255	0.540448979
BTAF1	0.966992255	0.473050428
HECTD2	0.966992255	0.39264278
PLCE1	0.966992255	0.387550071
FAS	0.966992255	0.385281043
PDLIM1	0.966992255	0.365733782
ATAD1	0.966992255	0.365313592
TBC1D12	0.966992255	0.31267213
RPP30	0.966992255	0.291057533

Table S2. One ordered list of genes used as input to GSEA.

Gene	Average relative CN	Correlation coeficient between avg relative CN and expression
LIPA	0.966992255	0.290351613
IFIT5	0.966992255	0.281040192
CPEB3	0.966992255	0.268215979
MYOF	0.966992255	0.26806471
TNKS2	0.966992255	0.267947057
LGI1	0.966992255	0.254954768
ACTA2	0.966992255	0.250920939
EXOC6	0.966992255	0.245122311
ANKRD22	0.966992255	0.235995774
NOC3L	0.966992255	0.230398837
41703	0.966992255	0.214095447
CD69	0.967383432	0.241779702
ZNF684	0.967603881	0.319400559
TM9SF3	0.967783635	0.337950789
BLNK	0.967783635	0.277157632
LMBRD1	0.968080164	0.257242771
EAPP	0.968238382	0.443092004
FAM177A1	0.968238382	0.430224412
PPP2R3C	0.968238382	0.409881522
PSMA6	0.968238382	0.392713533
SNX6	0.968238382	0.379778747
KIAA0391	0.968238382	0.367835069
BAZ1A	0.968238382	0.26939631
CFL2	0.968238382	0.249842945
BRMS1L	0.968238382	0.244618636
TCF7L2	0.968619472	0.316153971
VTI1A	0.968619472	0.260335399
CUEDC1	0.968825581	0.331702155
VEZF1	0.968825581	0.292059088
SUPT4H1	0.968825581	0.260777837
RAD51C	0.968825581	0.242123264
DHX40	0.968825581	0.226642151
MRPS23	0.968825581	0.224341812
TRIM37	0.968825581	0.216937071
STIM1	0.968879703	0.333387542
COBLL1	0.968988473	0.216991841
SPAG9	0.969031773	0.473484335
ANKRD40	0.969031773	0.357725428
TOB1	0.969031773	0.26199041
EME1	0.969031773	0.236842959
NME1	0.969031773	0.221136301
RIPK1	0.969074084	0.429819711
BPHL	0.969074084	0.371745039
TUBB2A	0.969074084	0.347508095
COQ9	0.969109699	0.453830015
CRAT	0.969118339	0.396593969
ENDOG	0.969118339	0.387825192
LRRC8A	0.969118339	0.370086057
ZDHHC12	0.969118339	0.320144346
PPP2R4	0.969118339	0.316196716
DOLK	0.969118339	0.306453631
ZER1	0.969118339	0.284430898
FAM73B	0.969118339	0.274099868
TBC1D13	0.969118339	0.259586031
SET	0.969118339	0.258494133
DOLPP1	0.969118339	0.255050457

Table S2. One ordered list of genes used as input to GSEA.

Gene	Average relative CN	Correlation coeficient between avg relative CN and expression
SH3GLB2	0.969118339	0.221050448
NAT1	0.96927045	0.352792588
DPP8	0.969553651	0.458902279
ZNF609	0.969553651	0.370880038
SPG21	0.969553651	0.310842718
RBPMS2	0.969553651	0.273142912
CLPX	0.969553651	0.268907356
PDCD7	0.969553651	0.257360425
PARP16	0.969553651	0.250284358
SURF4	0.969588693	0.401297527
RPL7A	0.969588693	0.352834077
SETX	0.969588693	0.326090988
SURF1	0.969588693	0.284279713
MED27	0.969588693	0.256948678
RALGDS	0.969588693	0.253891365
MED22	0.969588693	0.219286614
TMEM80	0.969700416	0.338174745
SIRT3	0.969700416	0.285880374
CD151	0.969700416	0.283259776
IFITM2	0.969700416	0.249561572
PDDC1	0.969700416	0.235769835
IFITM3	0.969700416	0.222666844
GALNT11	0.969768061	0.225061598
PRKAG2	0.969768061	0.2248599
IWS1	0.969906328	0.221496397
SLC38A1	0.970125472	0.397335453
COPS7A	0.970329199	0.293132482
C1RL	0.970329199	0.276334059
C12orf57	0.970329199	0.27258801
CD163L1	0.970329199	0.255201642
ENO2	0.970329199	0.228424956
PTPN6	0.970329199	0.226308355
PTPRN2	0.970384114	0.280545417
CHMP7	0.970493051	0.626345014
INTS10	0.970493051	0.480497424
TNFRSF10A	0.970493051	0.4531489
C8orf58	0.970493051	0.452678533
HR	0.970493051	0.445875
PIWIL2	0.970493051	0.420038373
SLC39A14	0.970493051	0.373203187
EPB49	0.970493051	0.322823445
TNFRSF10B	0.970493051	0.303051202
RHOBTB2	0.970493051	0.301992874
ATP6V1B2	0.970493051	0.296029284
POLR3D	0.970493051	0.293358267
PPP3CC	0.970493051	0.264615439
BMP1	0.970493051	0.261608446
EGR3	0.970493051	0.255779246
TNFRSF10C	0.970493051	0.255342476
REEP4	0.970493051	0.248169368
LOXL2	0.970493051	0.231706498
CDC27	0.970662812	0.315880204
WRNIP1	0.970747366	0.49717626
SERPINB1	0.970747366	0.234178562
XIRP2	0.970837498	0.246727338
SLC25A28	0.971526991	0.513170213

Table S2. One ordered list of genes used as input to GSEA.

Gene	Average relative CN	Correlation coeficient between avg relative CN and expression
CUTC	0.971526991	0.404492151
ENTPD1	0.971526991	0.271308581
TCTN3	0.971526991	0.260803819
AVPI1	0.971526991	0.252870623
SORBS1	0.971526991	0.242920513
GOLGA7B	0.971526991	0.216851896
NKX2-3	0.971526991	0.216784666
PI4K2A	0.971526991	0.214818174
RNF34	0.971555946	0.337718327
RNF10	0.971555946	0.277681007
TMEM120B	0.971555946	0.25446949
ANAPC5	0.971555946	0.237930654
CAMKK2	0.971555946	0.227274534
MRPL45	0.971582733	0.287406638
ZBTB43	0.971594231	0.334473401
RALGPS1	0.971594231	0.321219445
LRSAM1	0.971594231	0.295249083
FAM129B	0.971594231	0.268086033
UBA3	0.971679627	0.307615311
MDN1	0.971769976	0.283731801
RPP40	0.972031089	0.488817535
FARS2	0.972031089	0.467366833
CDYL	0.972031089	0.350411828
ODF2	0.972075344	0.322882489
GLE1	0.972075344	0.32126984
SPOP	0.972145486	0.352081111
PHB	0.972145486	0.246317349
SLC35B1	0.972145486	0.241949723
HEYL	0.972461731	0.22230131
KCTD2	0.972610715	0.332992247
GGA3	0.972610715	0.255016451
ATP5H	0.972610715	0.22830252
WNK1	0.9726541	0.285942757
KIAA1467	0.972787635	0.238302429
CCDC115	0.973139226	0.263600546
INSIG1	0.973206614	0.352854229
ASB6	0.973226489	0.243493142
C15orf39	0.973349581	0.381754659
CLRN3	0.973480345	0.223358326
PTPRE	0.973480345	0.218433652
SCPEP1	0.973509433	0.390218076
DGKE	0.973509433	0.253876821
COIL	0.973509433	0.219757925
STYK1	0.973525938	0.25948524
KLRC4	0.973525938	0.222931872
LRRK2	0.9737159	0.371636387
CNOT7	0.973805186	0.419181632
EGLN3	0.974088388	0.279914478
URM1	0.974193423	0.380853847
COQ4	0.974193423	0.219101831
SH2D4A	0.974288982	0.377352502
C16orf91	0.974839944	0.258371067
TMEM204	0.974839944	0.232991136
PSMD12	0.974905956	0.479289214
BPTF	0.974905956	0.408466236
HELZ	0.974905956	0.319895537

Table S2. One ordered list of genes used as input to GSEA.

Gene	Average relative CN	Correlation coeficient between avg relative CN and expression
ARHGAP15	0.974918222	0.222538495
ENTPD4	0.975336604	0.417585741
NKX3-1	0.975336604	0.409908668
EIF3A	0.975342613	0.541831768
RGS10	0.975342613	0.488584783
BAG3	0.975342613	0.441724748
FAM160B1	0.975342613	0.376140867
ABLIM1	0.975342613	0.334054235
GRK5	0.975342613	0.31743136
MXI1	0.975342613	0.306035699
TIAL1	0.975342613	0.287110161
GFRA1	0.975342613	0.239392929
INPP5F	0.975342613	0.233846369
AFAP1L2	0.975342613	0.231409243
ADRA2A	0.975342613	0.228938503
RAB11FIP2	0.975342613	0.2226524
CLEC14A	0.975597681	0.219776564
TRIM68	0.975602844	0.357691973
CNRIP1	0.975817603	0.233480532
PRPF4B	0.97582702	0.501892729
SLFN12	0.975955732	0.327336825
PIGW	0.975955732	0.291001531
SLFN5	0.975955732	0.26187282
GGNBP2	0.975955732	0.252129653
PEX12	0.975955732	0.232828102
SLFN11	0.975955732	0.227889324
TAF15	0.975955732	0.224630403
SNX17	0.976057421	0.405020011
MPV17	0.976057421	0.340191934
EIF2B4	0.976057421	0.33388897
KCNK3	0.976057421	0.265245499
ATP5G2	0.976097006	0.277714623
PFDN5	0.976097006	0.247208538
PCBP2	0.976097006	0.232014323
TOR1A	0.976183494	0.468575212
TOR1B	0.976183494	0.383037642
USP20	0.976183494	0.346232297
C9orf78	0.976183494	0.331432886
GPR107	0.976183494	0.310821221
ABL1	0.976183494	0.240587014
ASS1	0.976183494	0.217018827
STXBP1	0.97640331	0.378636455
TOR2A	0.97640331	0.33459099
CDK9	0.97640331	0.291519833
ST6GALNAC4	0.97640331	0.273377536
AK1	0.97640331	0.267145321
FPGS	0.97640331	0.234623574
DPM2	0.97640331	0.234170017
CNTN1	0.97681533	0.41829473
ARID2	0.97681533	0.369199262
ZCRB1	0.97681533	0.364812437
YAF2	0.97681533	0.343231272
NELL2	0.97681533	0.240619896
PPHLN1	0.97681533	0.231610936
PRICKLE1	0.97681533	0.216988185
PPP2R2D	0.976918898	0.276588503

Table S2. One ordered list of genes used as input to GSEA.

Gene	Average relative CN	Correlation coeficient between avg relative CN and expression
FOXJ2	0.977294928	0.587188876
NECAP1	0.977294928	0.256327137
INPP5A	0.977350069	0.287530355
CHFR	0.977690499	0.322776228
SMPDL3A	0.977860664	0.264570954
SIRT4	0.978245805	0.242972982
SLC35B3	0.978375462	0.477555077
FUT9	0.978482656	0.349366105
MANEA	0.978482656	0.249006968
PSMA4	0.978716295	0.237880231
MORF4L1	0.978716295	0.235392683
ILK	0.978716557	0.381273658
PRKCDBP	0.978716557	0.336144061
TRIM5	0.978716557	0.328294705
TAF10	0.978716557	0.296947704
TPP1	0.978716557	0.268491686
SBF2	0.97892577	0.256305855
NHLRC2	0.979138543	0.233745522
ARHGEF5	0.979357326	0.396955562
GSTK1	0.979357326	0.35797771
COPB1	0.979597082	0.441664958
PDE3B	0.979597082	0.398905295
PSMA1	0.979597082	0.253835072
RRAS2	0.979597082	0.21734481
COG1	0.979665066	0.332790759
C17orf80	0.979665066	0.248552049
GNPTG	0.979683497	0.33469575
PHC1	0.979895945	0.270706587
RIMKLB	0.979895945	0.224006971
APPBP2	0.980082426	0.42917272
BIN1	0.980105206	0.316108873
PID1	0.980190272	0.233495373
CNPY2	0.980282682	0.23361106
GLCE	0.98031531	0.227694728
NDUFB10	0.98068995	0.430211432
FAHD1	0.98068995	0.222417092
TSC2	0.98068995	0.220971624
SPSB3	0.98068995	0.219828697
BCLAF1	0.980830053	0.229056157
CIZ1	0.98083397	0.308973394
SLC25A25	0.98083397	0.293821217
C9orf16	0.98083397	0.290159161
GOLGA2	0.98083397	0.251421997
LCN2	0.98083397	0.222864678
SLC8A1	0.980855818	0.237178301
H2AFJ	0.981027583	0.315642368
MCM9	0.981081974	0.261209402
OSR1	0.981270695	0.291499442
RDH14	0.981270695	0.230436334
OSBP	0.981392908	0.469579162
MS4A6E	0.981392908	0.261194795
MS4A1	0.981392908	0.225713314
RFX6	0.981500281	0.25604942
GPRC6A	0.981500281	0.220736316
EXOC2	0.981864214	0.441950515
DUSP22	0.981864214	0.220681419

Table S2. One ordered list of genes used as input to GSEA.

Gene	Average relative CN	Correlation coeficient between avg relative CN and expression
NAP1L1	0.981919833	0.461658751
CSRP2	0.981919833	0.362593812
OSBPL8	0.981919833	0.302892648
BBS10	0.981919833	0.278420549
VPS37A	0.982045134	0.586061379
MTUS1	0.982045134	0.381115444
ASAH1	0.982045134	0.372598429
NLE1	0.982183157	0.218078963
GTF3C6	0.982273719	0.372840999
SLC16A10	0.982273719	0.271983267
KIAA1919	0.982273719	0.259199667
DCTN6	0.982321862	0.500769418
GSR	0.982321862	0.500450242
GTF2E2	0.982321862	0.454724165
TMEM66	0.982321862	0.439806915
LEPROTL1	0.982321862	0.417380643
PPP2CB	0.982321862	0.37432556
ZFAND6	0.982512226	0.361501308
KCTD9	0.982551797	0.409807875
DOCK5	0.982551797	0.376109388
GNRH1	0.982551797	0.313180906
ADAM28	0.982551797	0.264833824
FAM50B	0.982583777	0.311368503
SMAP1	0.983053587	0.421101626
FAM135A	0.983053587	0.301379949
ETV6	0.983144098	0.363013921
MANSC1	0.983144098	0.280819238
MGRN1	0.983238391	0.45498607
ZNF500	0.983238391	0.418395576
HCFC1R1	0.983238391	0.349903953
NAGPA	0.983238391	0.316422895
NMRAL1	0.983238391	0.289110284
CREBBP	0.983238391	0.284740267
ALG1	0.983238391	0.277311237
HMOX2	0.983238391	0.254435875
UBN1	0.983238391	0.253528256
ZNF213	0.983238391	0.246771536
ADCY9	0.983238391	0.225795451
DNAJA3	0.983238391	0.219929543
ZNF205	0.983238391	0.218584922
VASN	0.983238391	0.215475487
TEAD1	0.983393013	0.33022763
STK33	0.983491146	0.270895236
EIF3F	0.983491146	0.22052153
NCOR2	0.98360451	0.256536845
TMEM198	0.98386802	0.273366019
REPS1	0.983957244	0.335398856
HEBP2	0.983957244	0.295934235
BNIP2	0.984127327	0.395671484
GCNT3	0.984127327	0.266150884
TMEM109	0.984152182	0.309551129
DDB1	0.984152182	0.305987853
TMEM138	0.984152182	0.289633765
KIF3C	0.984297368	0.304105368
DNMT3A	0.984297368	0.270624028
DNAJC27	0.984297368	0.247143389

Table S2. One ordered list of genes used as input to GSEA.

Gene	Average relative CN	Correlation coeficient between avg relative CN and expression
C11orf84	0.984573118	0.303416244
TAOK3	0.984590177	0.246401765
PEBP1	0.984590177	0.241393053
CSPG4	0.984949164	0.265159226
UBAC1	0.985042469	0.409763933
CRIM1	0.985452251	0.334494055
SUPT7L	0.98557398	0.323888269
ZNF512	0.98557398	0.293583622
MRPL33	0.98557398	0.226284783
HDAC2	0.98558893	0.336003935
MPHOSPH10	0.985633732	0.278610128
PCYOX1	0.985633732	0.241195424
SLK	0.985736843	0.477794201
FBXW4	0.985736843	0.476247887
PPRC1	0.985736843	0.415487834
TRIM8	0.985736843	0.397923724
USMG5	0.985736843	0.391284659
C10orf76	0.985736843	0.388393724
FAM178A	0.985736843	0.375687057
LZTS2	0.985736843	0.373233124
CALHM2	0.985736843	0.366308327
SH3PXD2A	0.985736843	0.353416775
C10orf32	0.985736843	0.350240108
NT5C2	0.985736843	0.338088098
ITPRIP	0.985736843	0.318960867
CUEDC2	0.985736843	0.317986016
GSTO1	0.985736843	0.28832032
MGEA5	0.985736843	0.286908468
POLL	0.985736843	0.272739526
NFKB2	0.985736843	0.272302524
HPS6	0.985736843	0.265915575
FBXL15	0.985736843	0.25845293
SFXN3	0.985736843	0.251225593
ARL3	0.985736843	0.247729579
OBFC1	0.985736843	0.24036778
GBF1	0.985736843	0.239292083
ACTR1A	0.985736843	0.225223988
MRPL43	0.985736843	0.22475337
EPHA4	0.986040278	0.377323768
ITFG3	0.986440254	0.440861523
DECR2	0.986440254	0.357070284
UBE2I	0.986440254	0.29592351
RAB11FIP3	0.986440254	0.246250155
NARFL	0.986440254	0.243679975
TPSAB1	0.986440254	0.233768822
STUB1	0.986440254	0.218078963
AXIN1	0.986440254	0.215760761
GYPC	0.986449578	0.25291063
NRXN1	0.986482273	0.327202182
MED13L	0.986504239	0.404142596
PLEKHA3	0.986580663	0.276341042
PRKRA	0.986580663	0.270357379
PAK1IP1	0.986615409	0.472620408
TMEM14B	0.986615409	0.464362391
TMEM14C	0.986615409	0.360281166
GCNT2	0.986615409	0.27248099

Table S2. One ordered list of genes used as input to GSEA.

Gene	Average relative CN	Correlation coeficient between avg relative CN and expression
NOL7	0.986649025	0.54617098
DEK	0.986649025	0.439381524
NUP153	0.986649025	0.420189645
RANBP9	0.986649025	0.410400612
TBC1D7	0.986649025	0.361253955
DTNBP1	0.986649025	0.358668172
JARID2	0.986649025	0.329217118
SIRT5	0.986649025	0.258040938
MYLIP	0.986649025	0.252046624
FAM8A1	0.986649025	0.24900749
HIVEP1	0.986649025	0.237556168
TPMT	0.986649025	0.217491168
CEP68	0.986686381	0.384937811
SPRED2	0.986686381	0.25978512
ACTR2	0.986686381	0.214621907
KRT5	0.986728314	0.284807498
USP15	0.986728913	0.366476405
RASSF3	0.986728913	0.317532207
XPOT	0.986728913	0.313145382
DPY19L2	0.986728913	0.312036069
CAND1	0.986728913	0.30902748
LEMD3	0.986728913	0.243746139
GNS	0.986728913	0.228316615
TBK1	0.986728913	0.224215522
DNAJA4	0.986733742	0.234132101
WDR61	0.986733742	0.220450584
NQO2	0.986796725	0.520020083
ACP1	0.986815556	0.2431263
HDDC2	0.986960776	0.27976517
HINT3	0.986960776	0.227358573
PDPK1	0.987034322	0.280538327
ATP6V0C	0.987034322	0.275680884
RNPS1	0.987034322	0.221711166
GOPC	0.987246497	0.320843336
NUS1	0.987246497	0.247527885
MFHAS1	0.987283256	0.367273194
C11orf63	0.987452322	0.324661432
SORL1	0.987452322	0.306693785
ZNF202	0.987452322	0.256118809
PUM2	0.987469555	0.23944537
MED13	0.987687631	0.504193992
TLK2	0.987687631	0.419064662
YWHAQ	0.987840207	0.32318506
IAH1	0.987840207	0.240724798
GOSR2	0.988385453	0.381612987
BMPR1A	0.989665935	0.388491298
FAM35A	0.989665935	0.259862592
WAPAL	0.989665935	0.252080665
MMRN2	0.989665935	0.233995667
LDB3	0.989665935	0.218852003
UBE2Q2	0.989690747	0.328339597
ODF3L1	0.989690747	0.319112136
RCN2	0.989690747	0.304724694
ETFA	0.989690747	0.263932259
IDH3A	0.989690747	0.254183759
TSPAN3	0.989690747	0.228266192

Table S2. One ordered list of genes used as input to GSEA.

Gene	Average relative CN	Correlation coeficient between avg relative CN and expression
HMG20A	0.989690747	0.21470233
NACA	0.989940048	0.340441184
R3HDM2	0.989940048	0.283462877
TAC3	0.989940048	0.269462013
ZBTB39	0.989940048	0.258705046
MARS	0.989940048	0.228266192
SOCS5	0.989970707	0.369625684
EPAS1	0.989970707	0.280845598
RHOQ	0.989970707	0.273836644
ZNF830	0.990108972	0.320449414
LIG3	0.990108972	0.218717308
STOML2	0.990175432	0.292998094
VCP	0.990175432	0.233246104
FADS3	0.990294688	0.23559636
CCNC	0.990371377	0.358828874
COQ3	0.990371377	0.250149896
FBXL4	0.990371377	0.225509719
PRDM13	0.990371377	0.214685522
ISCU	0.990712071	0.24779681
DUSP14	0.990719025	0.389844283
DDX52	0.990719025	0.303348475
AATF	0.990719025	0.241428967
BLOC1S2	0.990763309	0.251290707
FNBP4	0.990809149	0.441489439
APLP2	0.991090264	0.440686379
SNX19	0.991090264	0.298558761
RNASEH1	0.991246216	0.299062998
RIC8B	0.991275104	0.221173317
VEGFB	0.991450224	0.276725296
MACROD1	0.991450224	0.251429404
COX8A	0.991450224	0.219359927
MARK2	0.991450224	0.214905833
BCL11A	0.99149546	0.419831309
UGP2	0.99149546	0.36982738
SERTAD2	0.99149546	0.31399106
MDH1	0.99149546	0.258709405
USP47	0.991583021	0.415007401
CTR9	0.991583021	0.349321567
MICALCL	0.991583021	0.272508809
EIF4G2	0.991583021	0.219445815
CCDC25	0.991744663	0.569929689
PPP2R2A	0.991744663	0.471136501
EPHX2	0.991744663	0.384673563
TRIM35	0.991744663	0.364582311
PTK2B	0.991744663	0.357392464
CLU	0.991744663	0.246101039
ECHS1	0.992438815	0.313565576
PAOX	0.992438815	0.276722965
ACAD8	0.992456621	0.374396014
THYN1	0.992456621	0.351738962
VPS26B	0.992456621	0.338191793
PPFIBP2	0.992684012	0.246422725
GHITM	0.99282741	0.24329233
CTDSP2	0.992897053	0.381435311
41707	0.992897053	0.267394658
CDK4	0.992897053	0.261730443

Table S2. One ordered list of genes used as input to GSEA.

Gene	Average relative CN	Correlation coeficient between avg relative CN and expression
DTX3	0.992897053	0.227240919
EHD3	0.992918281	0.267127984
SPAST	0.992918281	0.240554691
ATP2B1	0.992931821	0.312943688
EEA1	0.992931821	0.293228186
PREPL	0.99308442	0.273819836
PPM1B	0.99308442	0.237850623
DCTN3	0.993122742	0.538456651
GALT	0.993122742	0.373630525
UBAP1	0.993132437	0.419691801
NUDT2	0.993132437	0.315474384
C12orf65	0.993219841	0.290068327
SMURF2	0.993433847	0.462049097
GNA13	0.993433847	0.370169867
PSMC5	0.993433847	0.362093494
FTSJ3	0.993433847	0.322013867
DDX5	0.993433847	0.285880076
DDX42	0.993433847	0.253322724
TEX2	0.993433847	0.239218456
ICAM2	0.993433847	0.226894013
ZFC3H1	0.99348567	0.2322214
RAB21	0.99348567	0.215338985
UBE4A	0.993594828	0.51141403
CEP164	0.993594828	0.476756136
ATP5L	0.993594828	0.409238794
MLL	0.993594828	0.394699959
RPS25	0.993594828	0.388867617
RBM7	0.993594828	0.367908164
CCDC84	0.993594828	0.34631001
ARCN1	0.993594828	0.330191232
BUD13	0.993594828	0.320476265
TMEM25	0.993594828	0.311114263
BCL9L	0.993594828	0.308475423
USP28	0.993594828	0.306324011
BACE1	0.993594828	0.291869216
TRAPPC4	0.993594828	0.280288571
HINFP	0.993594828	0.280120492
ZNF259	0.993594828	0.275128546
PAFAH1B2	0.993594828	0.246302994
APOA1	0.993594828	0.244117967
NLRX1	0.993594828	0.228772352
PTPRZ1	0.993744417	0.260457441
IQUB	0.993744417	0.251952571
EFEMP1	0.993948302	0.314192756
TBRG1	0.99441805	0.351806194
ANO5	0.994426799	0.25912524
CREB3L2	0.994474427	0.228320462
TPCN1	0.994508002	0.368375682
VPS29	0.994508002	0.335600549
TRAFD1	0.994508002	0.315162313
TCTN1	0.994508002	0.302371607
ANKRD13A	0.994508002	0.278739896
GIT2	0.994508002	0.259158856
ACACB	0.994508002	0.249948203
BRAP	0.994508002	0.230501624
CDC42EP3	0.994645118	0.429105734

Table S2. One ordered list of genes used as input to GSEA.

Gene	Average relative CN	Correlation coeficient between avg relative CN and expression
EIF2AK2	0.994645118	0.309483897
MAP4K3	0.994645118	0.285230095
DHX57	0.994645118	0.28149874
SOS1	0.994645118	0.27605298
CYP1B1	0.994645118	0.263968099
ATL2	0.994645118	0.260068665
MERTK	0.994677968	0.261516348
ACTR3	0.994677968	0.249145628
LIMS3	0.994677968	0.227597716
ALPK3	0.994797238	0.493811997
ZNF592	0.994797238	0.483139069
C15orf40	0.994797238	0.316120355
PDE8A	0.994797238	0.282941836
ADAMTSL3	0.994797238	0.224013829
UNC13B	0.994917016	0.266540578
ESCO2	0.994977561	0.291407148
B3GAT3	0.995103768	0.358781473
UBXN1	0.995103768	0.354176108
GANAB	0.995103768	0.286356224
BSCL2	0.995103768	0.270741683
HNRNPUL2	0.995103768	0.233629836
C11orf48	0.995103768	0.23127673
SMAD3	0.995206853	0.353299121
SMAD6	0.995206853	0.278420549
DIS3L	0.995206853	0.224349984
FEM1B	0.995206853	0.214937638
PUS10	0.995868766	0.228774279
COMMD1	0.995868766	0.215445501
SST	0.995908894	0.235966134
FAU	0.995959248	0.356764525
FRMD8	0.995959248	0.327938973
SLC25A45	0.995959248	0.307601412
ZFPL1	0.995959248	0.278977555
ARL2	0.995959248	0.263211743
MRPL49	0.995959248	0.249832653
SYVN1	0.995959248	0.244605396
HNRNPA1	0.996018648	0.338777216
METTL4	0.99665681	0.456698726
DDX10	0.996915101	0.321047733
ABI2	0.997327962	0.250389423
ANXA11	0.997507571	0.422009245
TSPAN14	0.997507571	0.261091748
PPP1CB	0.997659864	0.34967159
YPEL5	0.997659864	0.333166231
CLIP4	0.997659864	0.25731217
LBH	0.997659864	0.241243815
PLXNA4	0.997707325	0.419545573
IGF1	0.9977409	0.260637938
NT5DC3	0.9977409	0.247275769
METAP2	0.9977409	0.229224235
POLR2G	0.998060773	0.262304116
ZBTB3	0.998060773	0.251597483
TMEM179B	0.998060773	0.232083509
PTS	0.998191261	0.290258102
BCO2	0.998191261	0.249649869
SYT1	0.998328495	0.24381337

Table S2. One ordered list of genes used as input to GSEA.

Gene	Average relative CN	Correlation coeficient between avg relative CN and expression
CCDC50	0.999141792	0.303315397
OPA1	0.999141792	0.296810739
ZC3H15	1.000205524	0.231883768
PAPOLG	1.000242071	0.249969222
ORMDL1	1.000584096	0.282425554
ASNSD1	1.000584096	0.256927752
SCARB1	1.000883478	0.290219597
SLC39A13	1.001191872	0.228753617
ZRANB1	1.001220695	0.369098415
CTBP2	1.001220695	0.365467939
FAM175B	1.001220695	0.287143776
METTL10	1.001220695	0.278302894
FAM53B	1.001220695	0.257377233
TMEM218	1.001383779	0.417827632
HYLS1	1.001383779	0.37923669
DCPS	1.001383779	0.362714522
TIRAP	1.001383779	0.319501406
RPUSD4	1.001383779	0.313282483
FOXRED1	1.001383779	0.313181635
FAM118B	1.001383779	0.276960607
PUS3	1.001383779	0.226604133
GAS2	1.001653277	0.314540426
GTPBP8	1.001820416	0.373000958
QTRTD1	1.001820416	0.355655203
C3orf17	1.001820416	0.309736015
ZDHHC23	1.001820416	0.27060722
GRAMD1C	1.001820416	0.249345224
B4GALT4	1.001820416	0.222200463
STAT5B	1.001921728	0.374456798
NKIRAS2	1.001921728	0.309782326
EIF1	1.001921728	0.214752847
RANBP2	1.002164679	0.269653713
NCK2	1.002164679	0.214438825
AKAP13	1.002608518	0.219542965
ASCC3	1.002826228	0.223576827
ALG9	1.003034814	0.395594111
C11orf57	1.003034814	0.394837749
PIH1D2	1.003034814	0.310360522
TIMM8B	1.003034814	0.264777109
DLAT	1.003034814	0.238657409
SIK2	1.003034814	0.221832558
CUL5	1.003057608	0.385909427
NPAT	1.003057608	0.368446017
ALKBH8	1.003057608	0.341452526
ATM	1.003057608	0.339586849
C11orf65	1.003057608	0.297449439
KDELC2	1.003057608	0.229780826
SMARCC2	1.003524597	0.361165153
RNF41	1.003524597	0.23826681
ZC3H10	1.003524597	0.233409367
MYL6B	1.003524597	0.228988926
PRDM11	1.003821978	0.318154094
TSPAN18	1.003821978	0.309380443
KHDRBS2	1.003939954	0.247126581
NFRKB	1.004021856	0.318879514
TMEM45B	1.004021856	0.301752262

Table S2. One ordered list of genes used as input to GSEA.

Gene	Average relative CN	Correlation coeficient between avg relative CN and expression
BARX2	1.004021856	0.256286888
ABTB1	1.004427637	0.319366943
CHCHD6	1.004427637	0.28870933
MGLL	1.004427637	0.281347469
RUVBL1	1.004427637	0.273599026
EEFSEC	1.004427637	0.223108089
RNF38	1.004604122	0.424697731
HINT2	1.004604122	0.361384474
CCDC107	1.004604122	0.358629533
TESK1	1.004604122	0.340588027
CLTA	1.004604122	0.242065276
TLN1	1.004604122	0.227971399
CREB3	1.004604122	0.217270803
NDUFB8	1.004973161	0.473592261
HIF1AN	1.004973161	0.287698432
SEC31B	1.004973161	0.236218243
NF1	1.005150815	0.417175548
UTP6	1.005150815	0.333182726
RNF135	1.005150815	0.235163103
PPP1R2	1.005322397	0.263951291
ACAP2	1.005322397	0.216721087
VAMP5	1.005803362	0.2171749
DCDC2	1.005957768	0.303795854
WRN	1.005977904	0.553550951
EML4	1.006022853	0.367390214
SF3B1	1.006148869	0.241817321
ZMIZ1	1.006174116	0.293228186
ZCCHC24	1.006174116	0.222064129
PARP12	1.006363148	0.249313709
TBXAS1	1.006363148	0.218790302
TTC39C	1.006600471	0.273211851
GJC1	1.007192191	0.324313084
NMT1	1.007192191	0.269583361
PLEKHM1	1.007192191	0.229031627
HEXIM1	1.007192191	0.214383279
ALX1	1.007428007	0.237779384
CCDC59	1.007428007	0.237123882
HSD17B12	1.007617909	0.232720249
ATG3	1.007734426	0.438551775
SLC35A5	1.007734426	0.281666819
ABHD10	1.007734426	0.242302713
PMM2	1.007939641	0.338357022
TMEM186	1.007939641	0.246250496
ERI1	1.008020105	0.537075941
TNKS	1.008020105	0.489770387
PPP1R3B	1.008020105	0.347904123
SIPA1	1.008244261	0.354713961
RNASEH2C	1.008244261	0.253328697
RELA	1.008244261	0.234066841
CFL1	1.008244261	0.22062052
RFC4	1.008250002	0.232839864
EIF4A2	1.008250002	0.214099054
SHOC2	1.008302402	0.231577321
E2F3	1.008761158	0.360045017
MBOAT1	1.008761158	0.242022519
BECN1	1.008887457	0.364545645

Table S2. One ordered list of genes used as input to GSEA.

Gene	Average relative CN	Correlation coeficient between avg relative CN and expression
EZH1	1.008887457	0.286701098
AOC2	1.008887457	0.22384087
ZNF148	1.009355709	0.479394977
SNX4	1.009355709	0.376497002
MUC13	1.009355709	0.326711996
OSBPL11	1.009355709	0.288188285
SULT1A3	1.009716689	0.438215617
BCKDK	1.009716689	0.389220583
GDPD3	1.009716689	0.357067067
KCTD13	1.009716689	0.34141891
CD2BP2	1.009716689	0.338242217
PYCARD	1.009716689	0.326796036
ORAI3	1.009716689	0.325888409
STX4	1.009716689	0.316593639
ALDOA	1.009716689	0.306038277
TMEM219	1.009716689	0.293550006
INO80E	1.009716689	0.286490687
ZNF688	1.009716689	0.284641818
HIRIP3	1.009716689	0.270741683
MAPK3	1.009716689	0.264253833
SETD1A	1.009716689	0.236756106
TBC1D10B	1.009716689	0.232570938
BCL7C	1.009716689	0.230285063
ZNF785	1.009716689	0.227982381
ZNF646	1.009716689	0.22615032
DCTPP1	1.009716689	0.225578851
CHST10	1.01006876	0.262373558
LNP1	1.010255771	0.329014679
TBC1D23	1.010255771	0.237882235
FYTTD1	1.010534628	0.381001519
PIGX	1.010534628	0.302743928
RPL35A	1.010534628	0.2671616
BDH1	1.010534628	0.26620355
KIAA0226	1.010534628	0.245344943
LRCH3	1.010534628	0.23912602
LMLN	1.010534628	0.23191543
PAK2	1.010534628	0.226049472
CWF19L2	1.010649469	0.513128436
CASP4	1.010649469	0.267699453
KBTBD3	1.010649469	0.256942396
AASDHPPT	1.010649469	0.251261325
NSUN3	1.010726019	0.238336048
PIP4K2B	1.010904495	0.309815923
PSMB3	1.010904495	0.284886853
PCGF2	1.010904495	0.27217034
RPL23	1.010904495	0.250399401
MLLT6	1.010904495	0.238203643
LASP1	1.010904495	0.222261805
KRR1	1.011802296	0.358795258
GLIPR1	1.011802296	0.239998009
MBD4	1.011805213	0.320409033
GDF11	1.012395613	0.264033106
RPS26	1.012395613	0.235728838
RHOT1	1.012637526	0.415596483
PSMD11	1.012637526	0.341833986
ZNF207	1.012637526	0.265350123

Table S2. One ordered list of genes used as input to GSEA.

Gene	Average relative CN	Correlation coeficient between avg relative CN and expression
LRRC37B	1.012637526	0.233483246
YES1	1.012661585	0.456172319
ENOSF1	1.012661585	0.450396285
USP14	1.012661585	0.380882394
C16orf58	1.012673694	0.279885182
SLC5A2	1.012673694	0.243462458
MRPS11	1.012687959	0.266537462
ARHGAP17	1.013104291	0.22166261
EMP2	1.013191533	0.450313513
TXNDC11	1.013191533	0.337886405
NUBP1	1.013191533	0.335953512
LITAF	1.013191533	0.302774993
TEKT5	1.013191533	0.274739649
TCF7L1	1.013290073	0.319420058
WDR53	1.013367228	0.334494055
TAOK1	1.01336881	0.472896386
NUFIP2	1.01336881	0.383880793
PIPOX	1.01336881	0.379378778
TNFAIP1	1.01336881	0.328176754
SUPT6H	1.01336881	0.298006532
RPL23A	1.01336881	0.285256422
SDF2	1.01336881	0.271599189
IFT20	1.01336881	0.257169222
FLOT2	1.01336881	0.239665118
TMEM199	1.01336881	0.239396341
FZD2	1.01415792	0.258580301
IFT57	1.014629076	0.311450421
CD47	1.014629076	0.276305099
CCDC54	1.014629076	0.227999189
GOLGB1	1.014686887	0.418735259
KPNA1	1.014686887	0.40767566
FAM162A	1.014686887	0.404885548
RABL3	1.014686887	0.375454912
NDUFB4	1.014686887	0.348410998
GSK3B	1.014686887	0.319165248
WDR5B	1.014686887	0.29215495
ILDR1	1.014686887	0.268691119
GTF2E1	1.014686887	0.215040296
SLC41A3	1.014762218	0.25386655
INTS9	1.014848919	0.533123725
ELP3	1.014848919	0.471321287
ZNF395	1.014848919	0.411081128
DUSP4	1.014848919	0.352050476
FBXO16	1.014848919	0.331203282
HMBOX1	1.014848919	0.251678209
MINA	1.015099324	0.233007943
TMEM39A	1.015101511	0.284994784
RNF121	1.015866378	0.271229112
RPN1	1.016178518	0.358630202
RAB7A	1.016178518	0.333771315
ACAD9	1.016178518	0.316879374
BCCIP	1.016194117	0.368695029
UROS	1.016194117	0.350878803
DHX32	1.016194117	0.213878749
USP7	1.016800961	0.505375736
ANKS4B	1.016890988	0.279429014

Table S2. One ordered list of genes used as input to GSEA.

Gene	Average relative CN	Correlation coeficient between avg relative CN and expression
ERI2	1.016890988	0.264133953
ACSM3	1.016890988	0.218013458
WDR91	1.017104712	0.376853142
C7orf49	1.017104712	0.290409375
FCHSD2	1.01729335	0.23663845
EDF1	1.017323296	0.436087061
FBXW5	1.017323296	0.415005041
PHPT1	1.017323296	0.364223408
TMEM203	1.017323296	0.356563327
SSNA1	1.017323296	0.34960878
NRARP	1.017323296	0.302338017
MRPL41	1.017323296	0.286026749
EHMT1	1.017323296	0.281104811
NPDC1	1.017323296	0.273276746
ANAPC2	1.017323296	0.258141366
MAN1B1	1.017323296	0.256881485
UAP1L1	1.017323296	0.238386421
C9orf142	1.017323296	0.236253021
PMPCA	1.017323296	0.229332071
PNPLA7	1.017323296	0.217421989
ADIPOR2	1.017574366	0.401280729
DCP1B	1.017574366	0.311375569
FBXL14	1.017574366	0.306823196
XYLT1	1.017873786	0.335432472
ABCC1	1.017873786	0.298993247
ABCC6	1.017873786	0.243393176
KALRN	1.018007161	0.265144652
SENP2	1.018210823	0.251210901
MAP3K13	1.018210823	0.224570377
TMEM41A	1.018210823	0.216334505
ATR	1.018250266	0.333919773
PLS1	1.018250266	0.330843953
PCYT1A	1.018442313	0.238739438
HK1	1.018551211	0.374157551
EIF4EBP2	1.018551211	0.363921625
VPS26A	1.018551211	0.313750461
SLC29A3	1.018551211	0.218870654
TSPAN15	1.018551211	0.215475487
SULT1A4	1.018587704	0.434517879
MVP	1.018587704	0.388430612
UQCRC2	1.018954297	0.445052684
COG7	1.018954297	0.407571379
CDR2	1.018954297	0.329465717
ERN2	1.018954297	0.304539808
METTL9	1.018954297	0.276756581
POLR3E	1.018954297	0.242199825
GGA2	1.018954297	0.237813
C3orf37	1.019011119	0.450065188
CNBP	1.019011119	0.373320308
ISY1	1.019011119	0.338645607
STRAP	1.019274593	0.277190778
C16orf72	1.019914674	0.263428027
COX17	1.019945064	0.25201768
PTP4A1	1.020175742	0.295852689
PHF3	1.020175742	0.264321065
GOSR1	1.020334538	0.328428732

Table S2. One ordered list of genes used as input to GSEA.

Gene	Average relative CN	Correlation coeficient between avg relative CN and expression
BLMH	1.020334538	0.267281958
TP53I13	1.020334538	0.218482128
RBBP6	1.020591002	0.306292976
THUMPD1	1.020686919	0.282202295
PLA2G10	1.020783394	0.345130549
PDXDC1	1.020783394	0.259881589
MKL2	1.020783394	0.250923053
NPIP	1.020783394	0.241477092
BFAR	1.020783394	0.222585169
NTAN1	1.020783394	0.218719384
MAK16	1.021274801	0.447248741
FUT10	1.021274801	0.422773027
IMPACT	1.021462472	0.321087759
ALDH5A1	1.021713738	0.390016586
GMNN	1.021713738	0.388169599
MRS2	1.021713738	0.352724231
C6orf62	1.021713738	0.335161059
NSMCE1	1.021850902	0.492068135
KIAA0556	1.021850902	0.290457352
IL21R	1.021850902	0.243395227
IL4R	1.021850902	0.240689155
XPO6	1.021850902	0.232201164
UBXN7	1.022238244	0.241613589
RNF168	1.022238244	0.232251588
KIAA1279	1.022347141	0.41424406
RUFY2	1.022347141	0.325818433
SLC25A16	1.022347141	0.311262912
DDX50	1.022347141	0.243090637
ADCK2	1.022381753	0.280391781
SSBP1	1.022381753	0.233816693
NDUFB2	1.022381753	0.228438119
SULT1A1	1.022383635	0.365622289
SULT1A2	1.022383635	0.343570322
CCDC101	1.022383635	0.28798659
EIF3C	1.022383635	0.274220919
RPS15A	1.022533618	0.394696634
TMC7	1.022533618	0.336777092
C16orf62	1.022533618	0.296236775
ARL6IP1	1.022533618	0.269058626
TMC5	1.022533618	0.221492665
COQ7	1.022533618	0.218820231
IQCK	1.022533618	0.215962912
SAE1	1.022671354	0.406398259
NAPA	1.022671354	0.389388662
AP2S1	1.022671354	0.338746454
SLC1A5	1.022671354	0.317165108
CARD8	1.022671354	0.270691259
KDELR1	1.022671354	0.265178268
TMEM160	1.022671354	0.264522759
GNG8	1.022671354	0.262051998
C5AR1	1.022671354	0.24969819
PPP5C	1.022671354	0.232806249
FKRP	1.022671354	0.215695805
ZBTB11	1.022691186	0.276338714
RPL24	1.022691186	0.258421491
RAB12	1.022892132	0.299249626

Table S2. One ordered list of genes used as input to GSEA.

Gene	Average relative CN	Correlation coeficient between avg relative CN and expression
WSB1	1.023313203	0.231937778
EHHADH	1.023468999	0.327703663
CEP63	1.023624771	0.371248273
SLCO1C1	1.024008876	0.219725222
NDE1	1.024016292	0.452111943
C16orf45	1.024016292	0.214904023
CSGALNACT2	1.024249964	0.306539932
CXCL12	1.024249964	0.28966494
ZNF33B	1.024249964	0.269764552
ZNF32	1.024249964	0.232451324
JMJD1C	1.024271839	0.354862242
SEC22A	1.024373994	0.417558706
HSPBAP1	1.024373994	0.329065103
DIRC2	1.024373994	0.309719208
DTX3L	1.024373994	0.227494952
RNF7	1.02490717	0.371824405
C3orf58	1.02490717	0.324627817
XRN1	1.02490717	0.255496916
TFDP2	1.02490717	0.253597623
ATP1B3	1.02490717	0.233058367
TGIF1	1.024977602	0.217505981
ACADSB	1.024998138	0.354257163
IKZF5	1.024998138	0.305632313
OAT	1.024998138	0.26280614
FAM24A	1.024998138	0.214265328
AIP	1.025136153	0.334677798
CCS	1.025136153	0.332040423
BANF1	1.025136153	0.322498839
BRMS1	1.025136153	0.292681387
DRAP1	1.025136153	0.286902681
RAB1B	1.025136153	0.283929335
POLD4	1.025136153	0.261570446
YIF1A	1.025136153	0.247879616
B3GNT1	1.025136153	0.239429938
CDK2AP2	1.025136153	0.237632492
ZDHHC24	1.025136153	0.225503928
NDUFV1	1.025136153	0.223740079
CORO1B	1.025136153	0.218431733
C11orf68	1.025136153	0.218146157
STAT1	1.025609121	0.221328316
SLC37A3	1.026177684	0.264709876
COL11A2	1.027375933	0.231836987
CCDC14	1.027694267	0.305365961
ZXDC	1.027882134	0.342982045
DNAJC19	1.027978242	0.420920286
FXR1	1.027978242	0.30313051
DNM1L	1.028000709	0.308267861
PIK3R4	1.02800124	0.261228411
NDUFS8	1.028093158	0.292009444
LRP5	1.028093158	0.231400224
CHKA	1.028093158	0.223084935
NR1H2	1.02852136	0.37183808
ALDH16A1	1.02852136	0.36936734
FTL	1.02852136	0.337953636
TBC1D17	1.02852136	0.331600302
IRF3	1.02852136	0.324053618

Table S2. One ordered list of genes used as input to GSEA.

Gene	Average relative CN	Correlation coeficient between avg relative CN and expression
NUCB1	1.02852136	0.302371607
GYS1	1.02852136	0.288068203
AKT1S1	1.02852136	0.288034588
PPP1R15A	1.02852136	0.283277992
FCGRT	1.02852136	0.278470972
LIN7B	1.02852136	0.262738908
NUP62	1.02852136	0.253444217
FAM71E1	1.02852136	0.248132965
BCL2L12	1.02852136	0.244250372
POLD1	1.02852136	0.226013952
JOSD2	1.02852136	0.215290601
RNMT	1.02883248	0.328378336
PSMG2	1.028866096	0.474660235
CEP192	1.028866096	0.381562591
PTPN2	1.028866096	0.35360978
AFG3L2	1.028866096	0.318349593
SEH1L	1.028866096	0.285877968
CEP76	1.028866096	0.242907241
CTTN	1.02888599	0.314341381
CCDC90B	1.0290897	0.269044085
PRCP	1.0290897	0.240823618
NCOA4	1.029540708	0.390797234
FAM21A	1.029540708	0.295463618
C10orf128	1.029540708	0.219324464
TUBG2	1.029784643	0.351442765
MSL2	1.030381528	0.354545882
NCK1	1.030381528	0.342645887
NUDT16	1.030381528	0.25890892
ATP2C1	1.030381528	0.254017821
STAG1	1.030381528	0.235629976
MRPL3	1.030381528	0.234268536
DNAJC13	1.030381528	0.233730683
UBA5	1.030381528	0.219477582
ASTE1	1.030381528	0.218485916
RALBP1	1.030483993	0.491693979
NDUFV2	1.030483993	0.482757143
PPP4R1	1.030483993	0.398411551
ANKRD12	1.030483993	0.395858169
VAPA	1.030483993	0.387845254
TWSG1	1.030483993	0.31698891
RASA2	1.030706961	0.42433229
SERP1	1.030706961	0.424029748
RNF13	1.030706961	0.402179476
CEP70	1.030706961	0.310845337
SIAH2	1.030706961	0.31066045
ARMC8	1.030706961	0.309030084
MRPS22	1.030706961	0.291886024
PLSCR1	1.030706961	0.28798659
DBR1	1.030706961	0.286944501
COPB2	1.030706961	0.275884901
FAIM	1.030706961	0.272103123
MBNL1	1.030706961	0.253765702
PARL	1.030808612	0.424886951
DCUN1D1	1.030808612	0.414281165
MCCC1	1.030808612	0.325115246
LAMP3	1.030808612	0.281397893

Table S2. One ordered list of genes used as input to GSEA.

Gene	Average relative CN	Correlation coeficient between avg relative CN and expression
ATP11B	1.030808612	0.279380945
KLHL24	1.030808612	0.25514395
AP2M1	1.030808612	0.248572061
ALDH3B2	1.031050163	0.244939867
ZSCAN16	1.031580697	0.416789181
ABT1	1.031580697	0.413563856
ZKSCAN4	1.031580697	0.402997559
BTN2A1	1.031580697	0.391322557
ZNF184	1.031580697	0.338575065
ZKSCAN3	1.031580697	0.291320703
HIST1H2BJ	1.031580697	0.270574476
BTN1A1	1.031580697	0.253423142
HMGN4	1.031580697	0.239614722
PRSS16	1.031580697	0.234591951
PGBD1	1.031580697	0.232307347
HIST1H4I	1.031580697	0.217810186
HIST1H4K	1.031580697	0.217810186
HIST1H4L	1.031580697	0.217810186
CCDC6	1.0318637	0.379199879
HERC4	1.0318637	0.309161942
RHOBTB1	1.0318637	0.264470108
FAM13C	1.0318637	0.25409972
NRBF2	1.0318637	0.249965011
CISD1	1.0318637	0.248754852
RABAC1	1.032323987	0.368009011
EXOSC5	1.032323987	0.331855214
HNRNPUL1	1.032323987	0.287952975
ITPKC	1.032323987	0.259245078
ATP5SL	1.032323987	0.254001013
CCDC97	1.032323987	0.241361471
ZNF526	1.032323987	0.23368026
DEDD2	1.032323987	0.226671364
EHMT2	1.032649474	0.264980554
VARS	1.032649474	0.250181019
SGMS1	1.032712895	0.243073829
CCDC81	1.03278048	0.256118809
MYADM	1.033364913	0.298337745
PPP2R1A	1.033364913	0.28929517
ZNF331	1.033364913	0.263545681
FPR3	1.033364913	0.222921324
CRTC3	1.033585145	0.280235787
FURIN	1.033585145	0.280185364
IQGAP1	1.033585145	0.277580161
BLM	1.033585145	0.245779878
FES	1.033585145	0.231224358
RPS10	1.034099074	0.502965816
VPS52	1.034099074	0.445094762
RPS18	1.034099074	0.422131124
ZBTB9	1.034099074	0.403165545
C6orf89	1.034099074	0.396546911
RXRB	1.034099074	0.385795829
LEMD2	1.034099074	0.381562591
STK38	1.034099074	0.373415287
DAXX	1.034099074	0.36426007
MAPK13	1.034099074	0.355676003
TAF11	1.034099074	0.353189816

Table S2. One ordered list of genes used as input to GSEA.

Gene	Average relative CN	Correlation coeficient between avg relative CN and expression
NUDT3	1.034099074	0.352433881
MAPK14	1.034099074	0.34611762
RGL2	1.034099074	0.345176901
ZBTB22	1.034099074	0.336542439
PFDN6	1.034099074	0.332628373
RING1	1.034099074	0.332258805
MTCH1	1.034099074	0.329806214
WDR46	1.034099074	0.329016682
ITPR3	1.034099074	0.316468154
BRPF3	1.034099074	0.287709012
B3GALT4	1.034099074	0.279007356
RPL10A	1.034099074	0.269314584
KIFC1	1.034099074	0.258865877
SNRPC	1.034099074	0.252415228
CUTA	1.034099074	0.24747645
PPIL1	1.034099074	0.243024831
C6orf106	1.034099074	0.24210091
FANCE	1.034099074	0.24092501
KCTD20	1.034099074	0.225957489
ANKS1A	1.034099074	0.221102704
TRIM39	1.034752884	0.503016212
ABCF1	1.034752884	0.400477775
RPP21	1.034752884	0.399033098
DHX16	1.034752884	0.387122916
PPP1R11	1.034752884	0.367233416
ZNRD1	1.034752884	0.36004363
SCAND3	1.034752884	0.341111648
C6orf136	1.034752884	0.317560061
TRIM27	1.034752884	0.301937396
MRPS18B	1.034752884	0.296091496
GNL1	1.034752884	0.282064694
TRIM26	1.034752884	0.275698038
PPFIA1	1.035642747	0.315141068
FADD	1.035642747	0.296175488
PYROXD1	1.035757116	0.332443589
GOLT1B	1.035757116	0.247862817
RECQL	1.035757116	0.245897385
CCNL1	1.035965137	0.388716346
KPNA4	1.035965137	0.369757033
SLC33A1	1.035965137	0.363033872
IFT80	1.035965137	0.347738682
TIPARP	1.035965137	0.345873004
GFM1	1.035965137	0.335721032
NMD3	1.035965137	0.283482073
LXN	1.035965137	0.263631941
PPM1L	1.035965137	0.236890569
RSRC1	1.035965137	0.226889867
RARRES1	1.035965137	0.222503005
MRPL21	1.03618033	0.228578065
ATF6B	1.036445405	0.467588039
DOM3Z	1.036445405	0.412623137
RNF5	1.036445405	0.389978672
STK19	1.036445405	0.37291133
FKBPL	1.036445405	0.357574241
BRD2	1.036445405	0.337365568
SKIV2L	1.036445405	0.3065738

Table S2. One ordered list of genes used as input to GSEA.

Gene	Average relative CN	Correlation coeficient between avg relative CN and expression
AGPAT1	1.036445405	0.257673179
INTS4	1.036513365	0.371437823
ALG8	1.036513365	0.338729646
RSF1	1.036513365	0.258841689
CLNS1A	1.036513365	0.248135055
PAK1	1.036513365	0.23559636
NARS2	1.036513365	0.220099475
C11orf73	1.036576411	0.341116368
TMEM126B	1.036576411	0.335821879
TMEM126A	1.036576411	0.305012995
EED	1.036576411	0.279431368
CREBZF	1.036576411	0.229747211
TMEM135	1.036576411	0.227595799
PICALM	1.036576411	0.22534354
CSTF2T	1.036675802	0.253259332
C2CD3	1.036730249	0.383589936
RAB6A	1.036730249	0.28711258
RNF169	1.036730249	0.247177005
UVRAG	1.037777872	0.342965237
GDPD5	1.037777872	0.258808073
ARRB1	1.037777872	0.238151161
C10orf10	1.037854173	0.328911061
NRG1	1.038166693	0.251679342
SLCO1B1	1.038982298	0.26128487
COG5	1.039426079	0.270928852
GOLGA7	1.040320822	0.290046453
C10orf54	1.04098647	0.339432719
PSAP	1.04098647	0.244452065
HIST1H2BH	1.041097256	0.267853109
HIST1H4A	1.041097256	0.257555589
HIST1H4B	1.041097256	0.257555589
HIST1H4C	1.041097256	0.257555589
HIST1H4D	1.041097256	0.257555589
HIST1H4E	1.041097256	0.257555589
HIST1H4F	1.041097256	0.257555589
HIST1H4H	1.041097256	0.257555589
HIST1H2AC	1.041097256	0.254666236
HIST1H1D	1.041097256	0.248820335
HIST1H2BD	1.041097256	0.232576124
DIP2C	1.041394385	0.238569349
ZFP30	1.041424859	0.215746228
CCHCR1	1.04148039	0.350552441
TCF19	1.04148039	0.257270013
BIRC2	1.041572115	0.423340624
C11orf70	1.041572115	0.340813826
TMEM123	1.041572115	0.302441386
YAP1	1.041572115	0.262657082
TFPT	1.042684578	0.453792719
LENG1	1.042684578	0.36553517
NDUFA3	1.042684578	0.357739369
PRPF31	1.042684578	0.356576634
TTYH1	1.042684578	0.31143099
TSEN34	1.042684578	0.29319457
CNOT3	1.042684578	0.281849332
AARSD1	1.043448873	0.294159661
RUNDC1	1.043448873	0.263451885

Table S2. One ordered list of genes used as input to GSEA.

Gene	Average relative CN	Correlation coeficient between avg relative CN and expression
LMAN2L	1.043669104	0.248910316
ASB7	1.044032281	0.32906233
LRRC28	1.044032281	0.242653635
CCL24	1.044795809	0.218706261
HPS3	1.045134199	0.317097876
KCNE4	1.046034632	0.25201768
CUL3	1.046034632	0.223427439
FAM124B	1.046034632	0.217662329
PDCD10	1.046164015	0.409574953
SI	1.046164015	0.283717383
ZMYND11	1.047308396	0.334945047
MPPE1	1.047548372	0.357305464
IMPA2	1.047548372	0.214332883
CSNK2B	1.047622897	0.464513902
MDC1	1.047622897	0.395992558
NRM	1.047622897	0.394413493
MSH5	1.047622897	0.392028097
C6orf47	1.047622897	0.390902593
NFKBIL1	1.047622897	0.374826367
LY6G5C	1.047622897	0.329587833
FLOT1	1.047622897	0.320970169
LY6G5B	1.047622897	0.283576565
TUBB	1.047622897	0.249677062
GTF2H4	1.047622897	0.23235969
CLIC1	1.047622897	0.231517814
DDR1	1.047622897	0.21658389
STYXL1	1.047752814	0.256137774
POR	1.047752814	0.23761531
RPL27	1.047879533	0.385980614
VAT1	1.047879533	0.227570152
MRPL36	1.048053887	0.363168335
NDUFS6	1.048053887	0.296793931
CLPTM1L	1.048053887	0.291869216
NKD2	1.048053887	0.273565411
MRE11A	1.048711793	0.401759278
ENDOD1	1.048711793	0.252975731
GPATCH8	1.048719335	0.410926482
ATXN7L3	1.048719335	0.368140538
TMUB2	1.048719335	0.322918803
GRN	1.048719335	0.308018476
HDAC5	1.048719335	0.227990116
DHX8	1.048854051	0.323557148
DUSP3	1.048854051	0.273648614
COMMD3	1.049521975	0.313363882
BMI1	1.049521975	0.261478326
MSRB2	1.049521975	0.259074817
DLG5	1.049644693	0.396041255
RPS24	1.049644693	0.26573069
HKR1	1.049664807	0.279212866
ZBTB24	1.050690481	0.393015905
FIG4	1.050690481	0.309796517
CDC40	1.050690481	0.304488216
SMPD2	1.050690481	0.294190782
SNX3	1.050690481	0.27557813
SEC63	1.050690481	0.27216805
FOXO3	1.050690481	0.236891361

Table S2. One ordered list of genes used as input to GSEA.

Gene	Average relative CN	Correlation coeficient between avg relative CN and expression
PLD1	1.051267043	0.278772176
UPK3B	1.051417782	0.33589194
DTX2	1.051417782	0.30308264
NET1	1.051561157	0.357618715
MSRA	1.051689704	0.562622252
HSPBP1	1.052004242	0.406579721
UBE2S	1.052004242	0.379636881
RPL28	1.052004242	0.330877569
ISOC2	1.052004242	0.32208711
ZNF444	1.052004242	0.245107568
U2AF2	1.052004242	0.241544323
ZNF581	1.052004242	0.230451201
ZNF580	1.052004242	0.228820848
RDH13	1.052004242	0.215929296
NLRP11	1.052004242	0.214510839
ZFAND3	1.052484807	0.309345563
BTBD9	1.052484807	0.293672502
RNF8	1.052484807	0.261553647
PGM2L1	1.053210144	0.227141986
LSM12	1.053284712	0.427842636
G6PC3	1.053284712	0.255993322
TMEM101	1.053284712	0.249072314
PRKRIR	1.054240749	0.354428227
DNAJC1	1.054365529	0.358677604
ASB13	1.054518162	0.38024196
AGPAT6	1.054878412	0.35277282
CMAS	1.055062135	0.214517667
PVRL2	1.05529018	0.363033872
TOMM40	1.05529018	0.361806895
SNRPD2	1.05529018	0.359067207
OPA3	1.05529018	0.331233322
ZNF296	1.05529018	0.318257622
VASP	1.05529018	0.316946605
MARK4	1.05529018	0.316694487
CD3EAP	1.05529018	0.30657613
GEMIN7	1.05529018	0.303231357
ERCC1	1.05529018	0.299533619
CLPTM1	1.05529018	0.289700996
PVR	1.05529018	0.285280518
BLOC1S3	1.05529018	0.273027558
ZNF180	1.05529018	0.235428281
KCNMA1	1.055989066	0.218030266
KCNK5	1.056175587	0.312369304
AKR1C2	1.05640471	0.372342313
AKR1C1	1.05640471	0.352408309
AKR1C3	1.05640471	0.323448539
AKR1C4	1.05640471	0.258368891
CEP72	1.056800498	0.269548322
AHRR	1.056800498	0.216485776
TACC2	1.056899541	0.329921028
HTRA1	1.056899541	0.276048485
PLEKHA1	1.056899541	0.228055391
CHSY1	1.05693301	0.304707886
TM2D3	1.05693301	0.297799896
SNRPA1	1.05693301	0.237829808
ABHD11	1.056970794	0.356212865

Table S2. One ordered list of genes used as input to GSEA.

Gene	Average relative CN	Correlation coeficient between avg relative CN and expression
NSUN5	1.056970794	0.282862563
POM121	1.056970794	0.259566615
CLDN4	1.056970794	0.237817007
WBSCR22	1.056970794	0.237800199
MLXIPL	1.056970794	0.228740663
DNAJC30	1.056970794	0.214386594
DNAJB12	1.057007387	0.451557287
DDIT4	1.057007387	0.320624835
ASCC1	1.057007387	0.245208415
PION	1.057762154	0.301805228
MTRR	1.057994958	0.460351624
FASTKD3	1.057994958	0.365555058
MED10	1.057994958	0.27741442
SEMA5A	1.057994958	0.259446773
NSUN2	1.057994958	0.258337452
CD164	1.058282343	0.333213519
LSM1	1.058888761	0.526790613
ASH2L	1.058888761	0.496468949
EIF4EBP1	1.058888761	0.481837277
BRF2	1.058888761	0.401438671
PROSC	1.058888761	0.392115389
ERLIN2	1.058888761	0.392048194
BAG4	1.058888761	0.380692269
RAB11FIP1	1.058888761	0.278001781
NSUN6	1.059209082	0.308237516
ARL5B	1.059209082	0.241628362
RBM17	1.059361715	0.400091925
GDI2	1.059361715	0.398848151
FBXO18	1.059361715	0.277916316
PLAUR	1.060099259	0.310593218
ZNF576	1.060099259	0.295802265
LYPD3	1.060099259	0.225410772
XRCC1	1.060099259	0.22230131
UBE2M	1.06019425	0.425774183
TRIM28	1.06019425	0.387738222
CHMP2A	1.06019425	0.380863848
ZNF584	1.06019425	0.338794024
ZBTB45	1.06019425	0.336961978
RPS5	1.06019425	0.31353196
MZF1	1.06019425	0.302304376
ZNF8	1.06019425	0.274369879
ZNF446	1.06019425	0.248048926
ZNF587	1.06019425	0.236653265
MAGEF1	1.060376361	0.235747631
C11orf74	1.062088962	0.268201431
TRAF6	1.062088962	0.267814852
LDLRAD3	1.062088962	0.224366792
COMMD9	1.062088962	0.222686016
GOLIM4	1.062765383	0.294945062
TMEM147	1.06313778	0.383875671
WDR37	1.063281816	0.348223177
GTPBP4	1.063281816	0.324541043
IDI1	1.063281816	0.262100214
IDI2	1.063281816	0.245611801
WHSC1L1	1.063630344	0.515905052
DDHD2	1.063630344	0.297555475

Table S2. One ordered list of genes used as input to GSEA.

Gene	Average relative CN	Correlation coeficient between avg relative CN and expression
LETM2	1.063630344	0.283461361
ADAM9	1.063630344	0.281747893
TM2D2	1.063630344	0.224145131
GLO1	1.063767449	0.445615517
CEP57	1.06401011	0.458132981
JRKL	1.06401011	0.42641647
FAM76B	1.06401011	0.381976378
CCDC82	1.06401011	0.281364277
EIF3K	1.064187125	0.500320815
ACTN4	1.064187125	0.467898372
NFKBIB	1.064187125	0.4337111
MRPS12	1.064187125	0.408852213
FAM98C	1.064187125	0.40799501
ECH1	1.064187125	0.385119455
SPINT2	1.064187125	0.334443631
FBXO17	1.064187125	0.325199285
SARS2	1.064187125	0.325199285
YIF1B	1.064187125	0.304004521
C19orf33	1.064187125	0.258488723
CAPN12	1.064187125	0.242991837
RPL19	1.064375826	0.340758878
ZNF383	1.06484853	0.249714998
ZNF585B	1.06484853	0.217511058
PLD3	1.065142432	0.405742751
SERTAD3	1.065142432	0.359504213
SHKBP1	1.065142432	0.336242077
AKT2	1.065142432	0.272859479
ZNF780A	1.065142432	0.267178408
BLVRB	1.065142432	0.254942255
TRIM73	1.065246035	0.216756528
THAP1	1.065965701	0.359606867
POLB	1.065965701	0.342085965
VDAC3	1.065965701	0.336912007
SLC20A2	1.065965701	0.336744021
DKK4	1.065965701	0.285306817
AP3M2	1.065965701	0.284248508
FNTA	1.065965701	0.260192964
IKBKB	1.065965701	0.247106882
PMPCB	1.065966341	0.444774446
ATXN7L1	1.065966341	0.312545568
MLL5	1.065966341	0.291602746
PSMC2	1.065966341	0.276391467
FBXL13	1.065966341	0.239901204
PRKRIP1	1.065988839	0.438168885
SPDYE2	1.065988839	0.220403874
EPHB3	1.066632617	0.219612046
TAF3	1.066973013	0.278622242
LRRC17	1.069161025	0.306225744
FOXP4	1.070646049	0.407549603
TBCB	1.070729641	0.545365992
ALKBH6	1.070729641	0.518389309
POLR2I	1.070729641	0.507027167
ZNF146	1.070729641	0.474117296
COX6B1	1.070729641	0.440098102
RBM42	1.070729641	0.415558566
PSENEN	1.070729641	0.400028064

Table S2. One ordered list of genes used as input to GSEA.

Gene	Average relative CN	Correlation coeficient between avg relative CN and expression
U2AF1L4	1.070729641	0.344780491
ZNF567	1.070729641	0.311248727
ZNF565	1.070729641	0.303920481
CAPNS1	1.070729641	0.249496495
THAP8	1.070729641	0.239899183
SPOCK2	1.070986884	0.297799896
CHST3	1.070986884	0.219358079
CENPQ	1.07110028	0.255707747
MUT	1.07110028	0.230022742
NSMCE4A	1.071352497	0.401734286
ATE1	1.071352497	0.276048485
MTPAP	1.071616859	0.234064869
LRRC1	1.072000913	0.364411257
GCLC	1.072000913	0.332510783
PLXNA2	1.072218415	0.216097374
HACE1	1.072537479	0.280655981
UNC5CL	1.072764129	0.294983167
ORAOV1	1.073978956	0.221102704
CD2AP	1.074896211	0.276319585
TNFRSF21	1.074896211	0.264375806
GPR115	1.074896211	0.253641523
G0S2	1.075451313	0.24329233
ZNF451	1.075844959	0.288918509
ZBTB41	1.076076904	0.253713141
RCOR3	1.076297973	0.423824483
TRAF5	1.076297973	0.378359491
LPGAT1	1.076297973	0.302859032
ATP5C1	1.076660119	0.439539739
KIN	1.076660119	0.436110956
ANKH	1.077299977	0.316223866
FAM105A	1.077299977	0.312274009
FAM105B	1.077299977	0.276103404
DNAH5	1.077299977	0.273666258
TROVE2	1.077945366	0.251763441
CDC73	1.077945366	0.230854587
EIF5A2	1.07846876	0.230526699
DYRK3	1.078684211	0.40590741
MAPKAPK2	1.078684211	0.279496245
RASSF5	1.078684211	0.264470108
WBSCR17	1.078900394	0.28392147
KPNA3	1.07921468	0.377930921
KCNRG	1.07921468	0.275225363
TRIM13	1.07921468	0.259350853
MED20	1.079517064	0.521358568
TAF8	1.079517064	0.488834525
MEA1	1.079517064	0.462036191
KLC4	1.079517064	0.456864582
KLHDC3	1.079517064	0.456226527
TFEB	1.079517064	0.447982177
UBR2	1.079517064	0.4229301
RPL7L1	1.079517064	0.416918945
TBCC	1.079517064	0.413745458
PRICKLE4	1.079517064	0.401975012
MRPS10	1.079517064	0.39512431
PPP2R5D	1.079517064	0.388945245
MRPL2	1.079517064	0.37432034

Table S2. One ordered list of genes used as input to GSEA.

Gene	Average relative CN	Correlation coeficient between avg relative CN and expression
CNPY3	1.079517064	0.362734594
C6orf226	1.079517064	0.361139455
CUL7	1.079517064	0.326449381
FRS3	1.079517064	0.301884241
PGC	1.079517064	0.285009351
GNMT	1.079517064	0.268453488
BYSL	1.079517064	0.262123305
CCND3	1.079517064	0.22293326
CUL9	1.079517064	0.214890401
LIFR	1.080093686	0.356041785
DAB2	1.080093686	0.259110615
NFYA	1.08035599	0.366798001
DNAJC15	1.080861701	0.382936851
WAC	1.080936524	0.424765717
NDUFB5	1.081102619	0.312307624
MRPL47	1.081102619	0.272573744
ACTL6A	1.081102619	0.260892253
ICK	1.081344728	0.518806862
TMEM14A	1.081344728	0.456601778
GSTA1	1.081344728	0.280485629
GSTA4	1.081344728	0.229401195
DYRK4	1.081732393	0.276619632
PARP11	1.081732393	0.263684846
NDUFA9	1.081732393	0.225972386
FXYD5	1.081777109	0.379858582
LSR	1.081777109	0.353184442
USF2	1.081777109	0.261497337
GRAMD1A	1.081777109	0.230990995
CAMK2G	1.082020787	0.332407075
FUT11	1.082020787	0.260688362
SEC24C	1.082020787	0.249091007
ANGEL2	1.082122764	0.410361467
NENF	1.082122764	0.38557002
RPS6KC1	1.082122764	0.365518363
VASH2	1.082122764	0.365081361
TMEM206	1.082122764	0.30354815
TATDN3	1.082122764	0.26670554
FLVCR1	1.082122764	0.266201307
INTS7	1.082122764	0.246788344
MED4	1.08364534	0.442554454
SUCLA2	1.08364534	0.360998111
LRCH1	1.08364534	0.334120634
SETDB2	1.08364534	0.31977478
ESD	1.08364534	0.297802443
NUDT15	1.08364534	0.239007963
PAF1	1.084108767	0.506472507
MED29	1.084108767	0.459074223
TIMM50	1.084108767	0.458149789
FBL	1.084108767	0.438955165
SUPT5H	1.084108767	0.437593725
EID2	1.084108767	0.432232004
PSMC4	1.084108767	0.38268231
RPS16	1.084108767	0.3733035
LGALS14	1.084108767	0.304021329
DYRK1B	1.084108767	0.256757509
EID2B	1.084108767	0.240825637

Table S2. One ordered list of genes used as input to GSEA.

Gene	Average relative CN	Correlation coeficient between avg relative CN and expression
PAQR8	1.084577626	0.463589981
MCM3	1.084577626	0.395572594
IL17A	1.084577626	0.305599483
EFHC1	1.084577626	0.28948966
SLC35B4	1.08499557	0.245750403
KIF21B	1.084998937	0.405554447
NUAK2	1.084998937	0.360089456
NUCKS1	1.084998937	0.343113618
TMCC2	1.084998937	0.243527638
ZC3H11A	1.084998937	0.226282876
RBBP5	1.084998937	0.219358079
IPO9	1.084998937	0.219005116
DSTYK	1.084998937	0.215458679
HBS1L	1.085128983	0.31548166
TBPL1	1.085339369	0.283765417
MED23	1.085339369	0.251343247
UBA2	1.085573039	0.529179982
LSM14A	1.085573039	0.461830719
GPI	1.085573039	0.435257427
PDCD2L	1.085573039	0.36785774
KIAA0355	1.085573039	0.347301676
ABCC10	1.085967429	0.428353573
ZNF318	1.085967429	0.413611131
ZNF670	1.086277767	0.333751696
ZC3H13	1.087327497	0.536726414
SUB1	1.087672801	0.532054133
GOLPH3	1.087672801	0.469243004
DNAJC21	1.087672801	0.467478174
C5orf22	1.087672801	0.463914899
MTMR12	1.087672801	0.424315482
RAD1	1.087672801	0.385472421
ZFR	1.087672801	0.3158709
SPEF2	1.087672801	0.228890008
PDSS2	1.087832627	0.321303437
ACBD3	1.088049208	0.217979843
AARS2	1.088468617	0.391212022
PPP3CB	1.088710645	0.397873301
TTC18	1.088710645	0.38587256
ANXA7	1.088710645	0.350038415
ECD	1.088710645	0.295970332
MRPS16	1.088710645	0.230820972
GPATCH2	1.088763311	0.295547657
SPATA17	1.088763311	0.283866263
CENPF	1.088763311	0.266050037
KCTD3	1.088763311	0.262066598
SMYD2	1.088763311	0.217374764
TGFB2	1.088763311	0.215038485
TMEM9	1.088794867	0.305413812
TYW1	1.089086255	0.2923423
VNN3	1.0891353	0.320675258
RPS12	1.0891353	0.301833759
ZNF622	1.089754828	0.481243846
FAM134B	1.089754828	0.329081911
DNAJC24	1.09026836	0.396024447
ELP4	1.09026836	0.274453917
PAIP1	1.090821522	0.417293138

Table S2. One ordered list of genes used as input to GSEA.

Gene	Average relative CN	Correlation coeficient between avg relative CN and expression
C5orf28	1.090821522	0.395589392
ZNF131	1.090821522	0.346016829
NNT	1.090821522	0.290228797
C5orf34	1.090821522	0.256163456
MRPS30	1.090821522	0.240773823
LMBRD2	1.091164665	0.328812984
SMYD3	1.091352852	0.363014006
AHCTF1	1.091352852	0.300657216
SCCPDH	1.091352852	0.263293565
TFB2M	1.091352852	0.261310249
PREP	1.091785885	0.262150637
RNASEH2B	1.092026209	0.48510486
INTS6	1.092026209	0.454884497
WDFY2	1.092026209	0.33064336
DHRS12	1.092026209	0.242955592
TULP3	1.092441477	0.392142387
ITFG2	1.092441477	0.354161152
C4BPA	1.09247351	0.299480672
RCBTB1	1.092506661	0.246668044
C5orf51	1.092723138	0.369118333
FBXO4	1.092723138	0.26299324
PYCR2	1.093873998	0.271260443
RABIF	1.094686043	0.228736809
RHOU	1.094772349	0.34355062
JMJD4	1.094772349	0.329196793
ARF1	1.094772349	0.319549138
SNAP47	1.094772349	0.319364253
PARP1	1.094772349	0.302220337
ZNF678	1.094772349	0.288135434
MRPL55	1.094772349	0.286303388
PSEN2	1.094772349	0.22003039
HIST3H2A	1.094772349	0.214719137
CCDC122	1.094814208	0.290576676
RAB3GAP2	1.095078037	0.521948187
TP53BP2	1.095078037	0.345601167
NVL	1.095078037	0.317851554
FBXO28	1.095078037	0.317784323
MARK1	1.095078037	0.249965011
LYPLAL1	1.095078037	0.244586527
AIDA	1.095078037	0.214719137
ZNF669	1.095964874	0.422513478
ZNF124	1.095964874	0.351635153
ZNF33A	1.09640267	0.260436245
ZNF248	1.09640267	0.221845628
41704	1.096604996	0.391304763
CCT5	1.096604996	0.299718506
FAM173B	1.096604996	0.261161179
DAP	1.096604996	0.220334785
COG3	1.096647161	0.290343943
ASL	1.097947576	0.262323134
GUSB	1.097947576	0.234035197
PAN3	1.098402933	0.384075912
POMP	1.098402933	0.223517936
THSD1	1.098476573	0.303782682
VPS36	1.098476573	0.30242201
UTP14C	1.098476573	0.272436824

Table S2. One ordered list of genes used as input to GSEA.

Gene	Average relative CN	Correlation coeficient between avg relative CN and expression
ATP7B	1.098476573	0.220848867
ATP8A2	1.098645521	0.240752973
CDK8	1.098645521	0.236217437
RNF6	1.098645521	0.229430931
ZNF672	1.098921879	0.373048239
SH3BP5L	1.098921879	0.300942948
ZNF496	1.098921879	0.300152983
MPHOSPH8	1.099020816	0.467563372
ZMYM5	1.099020816	0.360054369
PSPC1	1.099020816	0.326692982
ARHGAP21	1.099463454	0.49821563
KIAA1217	1.099463454	0.27090748
PRTFDC1	1.099463454	0.264732848
DDX59	1.099529596	0.341096687
WDR26	1.100902827	0.495274272
EFCAB2	1.101039959	0.337701519
MFN1	1.101500376	0.371908444
ZNF639	1.101500376	0.318173582
PIK3CA	1.101500376	0.283885462
ZMAT3	1.101500376	0.258791265
CCNY	1.101660846	0.393335206
PARD3	1.101660846	0.392814165
CUL2	1.101660846	0.339449526
CREM	1.101660846	0.25409972
TDRD3	1.101898885	0.420498125
EXOC8	1.102364211	0.417034148
NUP133	1.102364211	0.387603759
TTC13	1.102364211	0.362644235
COG2	1.102364211	0.350542648
C1orf131	1.102364211	0.304590232
ABCB10	1.102364211	0.296354429
URB2	1.102364211	0.272369755
C1orf198	1.102364211	0.263142295
GALNT2	1.102364211	0.217677303
LMO2	1.102853422	0.224165099
LBR	1.103140987	0.232535363
NUPL1	1.103305353	0.243743067
POPDC3	1.103674607	0.224276229
COG6	1.103932143	0.376603846
UFM1	1.103932143	0.249590969
OPN3	1.104272856	0.372947393
SDCCAG8	1.104272856	0.34677771
RGS7	1.104272856	0.238653388
FH	1.104272856	0.221223741
WDR70	1.104311452	0.652902948
NIPBL	1.104311452	0.596579669
NUP155	1.104311452	0.355940938
C5orf42	1.104311452	0.259648468
GJA3	1.106226721	0.220998194
TARS	1.107047013	0.366109718
C1QTNF3	1.107047013	0.26820369
ZNF507	1.107141199	0.376681889
DPY19L3	1.107141199	0.298794071
IFT88	1.107402844	0.468134513
N6AMT2	1.107402844	0.269410841
IL17D	1.107402844	0.242063239

Table S2. One ordered list of genes used as input to GSEA.

Gene	Average relative CN	Correlation coeficient between avg relative CN and expression
ANKRD27	1.107407566	0.431778191
PDCD5	1.107407566	0.413541617
LRP3	1.107407566	0.320812423
NUDT19	1.107407566	0.310425139
GPATCH1	1.107407566	0.304374295
CEBPG	1.107407566	0.280355803
PEPD	1.107407566	0.273548603
C19orf40	1.107407566	0.264186601
RHPN2	1.107407566	0.246487881
LONRF1	1.107974137	0.410105273
VDAC2	1.108203784	0.311246105
COMTD1	1.108203784	0.25994882
FARP1	1.109129347	0.442403268
TGDS	1.109129347	0.373546533
STK24	1.109129347	0.360158189
UBAC2	1.109129347	0.35567301
TM9SF2	1.109129347	0.272436824
RAP2A	1.109129347	0.237244128
DNAJC3	1.109129347	0.234438792
SUGT1	1.110176585	0.333552294
NUFIP1	1.110626658	0.586113777
TPT1	1.110626658	0.507278778
TSC22D1	1.110626658	0.40329654
GTF2F2	1.110626658	0.332944744
KIAA1704	1.110626658	0.26343287
CDC5L	1.111450783	0.277587757
MIPEP	1.112051964	0.216294675
CENPJ	1.112625018	0.427348285
ATP12A	1.112625018	0.340870731
PARP4	1.112625018	0.332706767
SPATA13	1.112625018	0.301797928
CCNA1	1.11296844	0.451894377
EXOSC8	1.11296844	0.303933868
RFXAP	1.11296844	0.282431886
ALG5	1.11296844	0.282112716
AKAP11	1.113480526	0.405883497
KIF5B	1.113991111	0.44380891
ARHGAP12	1.113991111	0.293177762
PIGC	1.11402208	0.388074377
VAMP4	1.11402208	0.295766157
METTL13	1.11402208	0.241090513
PRDX6	1.11402208	0.235543952
TNFSF4	1.11402208	0.230989049
CROT	1.114882171	0.244286046
CARKD	1.115171887	0.434272832
ING1	1.115171887	0.422110773
CARS2	1.115171887	0.382903254
RAB20	1.115171887	0.27663643
PCCA	1.115257925	0.444238035
TMTC4	1.115257925	0.249105911
CLYBL	1.115257925	0.221253891
BCAP29	1.115461193	0.331790778
PNPLA8	1.115461193	0.322008497
DLD	1.115461193	0.273903876
TSNAX	1.115877724	0.483173041
DISC1	1.115877724	0.251405199

Table S2. One ordered list of genes used as input to GSEA.

Gene	Average relative CN	Correlation coeficient between avg relative CN and expression
GORAB	1.117254978	0.404381294
POLR1D	1.11728485	0.351655228
MTIF3	1.11728485	0.320813583
LNX2	1.11728485	0.313523129
RPL21	1.11728485	0.299580555
SAMD8	1.117686951	0.278118009
VCL	1.117686951	0.234871642
AP3M1	1.117686951	0.225812259
SERP2	1.118218519	0.349205618
RABL5	1.118279399	0.344793435
DHTKD1	1.118415293	0.304775117
NUDT5	1.118415293	0.293681995
CDC123	1.118415293	0.261663212
UPF2	1.118415293	0.257360425
RAB18	1.118837667	0.438867428
ABI1	1.118837667	0.31398577
ACBD5	1.118837667	0.311380567
PDSS1	1.118837667	0.270941095
MASTL	1.118837667	0.255124993
HNRNPU	1.120437346	0.231022665
EPC1	1.120747868	0.506573244
BIVM	1.121107931	0.381273807
TPP2	1.121107931	0.375427956
ERCC5	1.121107931	0.317590985
ARHGEF7	1.121314393	0.474471458
ANKRD10	1.121314393	0.314315293
XPO4	1.121382341	0.364757888
MYCBP2	1.121958059	0.352464512
COMMD6	1.121958059	0.285993152
UCHL3	1.121958059	0.279912123
PIBF1	1.121958059	0.269362713
PRPF18	1.122158648	0.294337498
PHYH	1.122158648	0.279815593
SEPHS1	1.122158648	0.267764429
OPTN	1.122158648	0.256704923
LMTK2	1.122295429	0.361908271
BAIAP2L1	1.122295429	0.31371319
BRI3	1.122295429	0.300072755
DGKH	1.122549998	0.234505985
SRI	1.122807985	0.318207198
SLC25A40	1.122807985	0.278288431
GPR158	1.12368122	0.249729702
RFWD2	1.123720774	0.406495682
DARS2	1.123720774	0.354744588
CENPL	1.123720774	0.307111396
SLC19A2	1.123720774	0.306489508
MRPS14	1.123720774	0.300959755
KLHL20	1.123720774	0.257797427
NME7	1.123720774	0.245897533
TBCE	1.124248023	0.333365117
C18orf8	1.124312545	0.419308965
RIOK3	1.124312545	0.380907447
NPC1	1.124312545	0.331133301
ARGLU1	1.124903861	0.405480335
EFNB2	1.124903861	0.272235243
MRPL14	1.125240083	0.413543967

Table S2. One ordered list of genes used as input to GSEA.

Gene	Average relative CN	Correlation coeficient between avg relative CN and expression
TMEM63B	1.125240083	0.368829704
SLC35B2	1.125240083	0.340217949
NFKBIE	1.125240083	0.232890285
HSP90AB1	1.125240083	0.213849363
STEAP2	1.125604092	0.301939693
STEAP1	1.125604092	0.284375287
HEATR1	1.125824205	0.307766898
COL4A4	1.125880638	0.258169373
IGSF9	1.127286289	0.396965682
PIGM	1.127286289	0.36081219
PEX19	1.127286289	0.329869103
COPA	1.127286289	0.314792542
VANGL2	1.127286289	0.313027727
DUSP23	1.127286289	0.310725064
NCSTN	1.127286289	0.282807374
PEA15	1.127286289	0.233728714
YIPF3	1.127335327	0.421586825
POLR1C	1.127335327	0.321932619
POLH	1.127335327	0.228877251
TJAP1	1.127335327	0.223688852
TRIM56	1.127472265	0.380957843
PLOD3	1.127472265	0.348704599
CLDN15	1.127472265	0.23425598
FIS1	1.127472265	0.229149217
ZNHIT1	1.127472265	0.228275691
TIPRL	1.127516705	0.362963583
XCL2	1.127516705	0.265646651
GPR161	1.127516705	0.254519914
XCL1	1.127516705	0.231409243
PPFIBP1	1.127676162	0.245122589
FAM107B	1.128473374	0.394024324
POU4F1	1.128647917	0.258813303
RNF219	1.128647917	0.224494125
GTPBP10	1.129400023	0.25523014
IVNS1ABP	1.130125858	0.271125981
SCYL3	1.13018657	0.457708928
KIFAP3	1.13018657	0.370140497
C1orf112	1.13018657	0.286185734
PIAS3	1.130514456	0.284740267
RBM8A	1.130514456	0.269310743
SAP18	1.130878391	0.426138809
MRP63	1.130878391	0.377759758
CTSB	1.13257786	0.310802842
ABHD13	1.133392943	0.298877542
MYO16	1.133392943	0.246852826
SDHC	1.133428796	0.33499547
UFC1	1.133428796	0.300674023
B4GALT3	1.133428796	0.264100337
DEDD	1.133428796	0.258419314
KLHDC9	1.133428796	0.256318344
USP21	1.133428796	0.251208785
ATF6	1.133428796	0.24704046
OLFML2B	1.133428796	0.243544446
NDUFS2	1.133428796	0.215996527
PFDN2	1.133428796	0.215811642
TMCO1	1.133831431	0.35503032

Table S2. One ordered list of genes used as input to GSEA.

Gene	Average relative CN	Correlation coeficient between avg relative CN and expression
POGK	1.133831431	0.347231519
RXRG	1.133831431	0.265377727
MPZL1	1.133831431	0.249561624
TRDMT1	1.13432338	0.293379455
CDC16	1.134606374	0.614822282
TUBGCP3	1.134606374	0.508622652
UPF3A	1.134606374	0.507547553
LAMP1	1.134606374	0.477444779
CUL4A	1.134606374	0.459252087
DCUN1D2	1.134606374	0.452633508
PCID2	1.134606374	0.416869666
GRTP1	1.134606374	0.347240202
F7	1.134606374	0.269161132
TFDP1	1.134606374	0.26470955
RBM26	1.134962643	0.468457623
NDFIP2	1.134962643	0.347727356
TPR	1.136440584	0.291984411
MRPS31	1.136529495	0.509177
ELF1	1.136529495	0.477814344
WBP4	1.136529495	0.446014929
MTRF1	1.136529495	0.427015913
KBTBD7	1.136529495	0.310787624
SLC25A15	1.136529495	0.300859756
KBTBD6	1.136529495	0.228340964
DIS3	1.136686684	0.311073197
BCL9	1.136903824	0.290757445
GJA5	1.136903824	0.240703935
TARBP1	1.137824677	0.394847904
SPRY2	1.138758574	0.359435857
TSEN15	1.139318725	0.306909702
HSD17B7	1.139894591	0.2681342
UHMK1	1.139894591	0.234165716
UAP1	1.139894591	0.233695099
DCLRE1C	1.140173386	0.500909091
NMT2	1.140173386	0.415323255
FAM171A1	1.140173386	0.387556601
RPP38	1.140173386	0.260774597
C1orf85	1.140217881	0.390931696
ETV3	1.140217881	0.3367939
NES	1.140217881	0.312540302
MEF2D	1.140217881	0.276067462
ARHGEF11	1.140217881	0.26189852
SCNM1	1.140852611	0.403924095
PRUNE	1.140852611	0.339516757
POGZ	1.140852611	0.337634288
ARNT	1.140852611	0.33761748
C1orf56	1.140852611	0.32546547
PI4KB	1.140852611	0.324692313
MLLT11	1.140852611	0.306371854
PSMD4	1.140852611	0.269243512
PIP5K1A	1.140852611	0.263041448
VPS72	1.140852611	0.251679402
PSMB4	1.140852611	0.224820601
FDFT1	1.142612647	0.409198555
ARID4B	1.142633756	0.401890356
GGPS1	1.142633756	0.38730122

Table S2. One ordered list of genes used as input to GSEA.

Gene	Average relative CN	Correlation coeficient between avg relative CN and expression
TDRKH	1.144648541	0.490988293
GON4L	1.144648541	0.468617164
RIT1	1.144648541	0.358761643
CLK2	1.144648541	0.336508168
ASH1L	1.144648541	0.312187339
ADAR	1.144648541	0.31060741
ARHGEF2	1.144648541	0.302741378
MSTO1	1.144648541	0.299480672
YY1AP1	1.144648541	0.29206845
SSR2	1.144648541	0.271512559
GBA	1.144648541	0.264503723
EFNA1	1.144648541	0.253898027
HAX1	1.144648541	0.245342877
UBAP2L	1.144648541	0.236501995
KIAA0907	1.144648541	0.235308644
UBQLN4	1.144648541	0.234266563
SCAMP3	1.144648541	0.22198009
GPR160	1.144874232	0.450671885
PRKCI	1.144874232	0.389743492
SKIL	1.144874232	0.346789563
RGS16	1.145845232	0.423253019
LAMC1	1.145845232	0.321582877
NPL	1.145845232	0.234686757
ABL2	1.14635106	0.322977921
TDRD5	1.14635106	0.293513918
FAM20B	1.14635106	0.239443353
TOR1AIP2	1.14635106	0.232300055
SOAT1	1.14635106	0.218921077
PRKAA1	1.146544378	0.428853616
TTC33	1.146544378	0.339233883
CARD6	1.146544378	0.259732507
PTGER4	1.146544378	0.257816407
FAM134C	1.148202029	0.422567887
STAT5A	1.148202029	0.317660852
STAT3	1.148202029	0.275362067
COASY	1.148202029	0.256110912
HSD17B1	1.148202029	0.242436881
POU2F1	1.148804853	0.438749774
ILDR2	1.148804853	0.262671677
CREG1	1.148804853	0.221660742
ARPC5	1.149017419	0.368510144
SLC25A44	1.149079202	0.376157675
THEM4	1.149079202	0.290253212
SF3B4	1.149835416	0.332911308
MYNN	1.151630988	0.515548774
SEC62	1.151630988	0.439778752
LRRC31	1.151630988	0.307954378
NEIL2	1.151805513	0.59528906
MTMR9	1.151805513	0.468517492
GATA4	1.151805513	0.30920509
RAP1B	1.152360761	0.298222404
CLDN12	1.154897219	0.350229201
FBXL20	1.154930298	0.417041159
XPR1	1.155543926	0.403722402
STX6	1.155543926	0.383653936
ACBD6	1.155543926	0.311800761

Table S2. One ordered list of genes used as input to GSEA.

Gene	Average relative CN	Correlation coeficient between avg relative CN and expression
IER5	1.155543926	0.228871272
MFF	1.156248085	0.231444809
TTYH3	1.157443157	0.261352064
UBE2V2	1.158674105	0.382486512
MCM4	1.158674105	0.327756789
TMEM68	1.159016518	0.439719221
TGS1	1.159016518	0.428010622
RPS20	1.159016518	0.352467478
CHCHD7	1.159016518	0.293202143
LYPLA1	1.159094222	0.426532348
MRPL15	1.159094222	0.42451652
ATP6V1H	1.159094222	0.3662423
TRIM44	1.159670132	0.333902965
GNA12	1.160400162	0.34576485
CARD11	1.163357168	0.300207144
RSU1	1.163573409	0.290841484
PTER	1.163573409	0.283950302
IMPAD1	1.165159024	0.325421789
PCMTD1	1.165603988	0.238959578
KDELR2	1.167299832	0.412891914
DAGLB	1.167299832	0.318282399
RAC1	1.167299832	0.292714984
C7orf26	1.167299832	0.276050808
TPCN2	1.169213936	0.331015711
MED1	1.169306376	0.496212793
OPRK1	1.170482015	0.278738579
PKD1L1	1.170969828	0.272976671
EIF3M	1.171934399	0.408831961
TCP11L1	1.171934399	0.247006845
HMGB1	1.173433855	0.495717292
USPL1	1.173433855	0.269276455
C1GALT1	1.173768609	0.42428134
MIOS	1.173768609	0.407684359
RPA3	1.173768609	0.264526993
PILRB	1.174070588	0.469650311
C7orf43	1.174070588	0.465249124
EPHB4	1.174070588	0.354446726
SLC12A9	1.174070588	0.352229334
ZCWPW1	1.174070588	0.337480318
CNPY4	1.174070588	0.330626562
AGFG2	1.174070588	0.311089996
LRCH4	1.174070588	0.283876551
TSC22D4	1.174070588	0.27781232
GAL3ST4	1.174070588	0.258040576
MOSPD3	1.174070588	0.245508952
TRIP6	1.174070588	0.239091955
GNB2	1.174070588	0.226711517
RB1CC1	1.175291094	0.414874144
APIP	1.175554784	0.263579296
EHF	1.175554784	0.24441845
PDS5B	1.176761602	0.496825978
N4BP2L2	1.176761602	0.447942979
N4BP2L1	1.176761602	0.292323697
MRPS18A	1.176775011	0.376570326
MAD2L1BP	1.176775011	0.332611655
GTPBP2	1.176775011	0.281785491

Table S2. One ordered list of genes used as input to GSEA.

Gene	Average relative CN	Correlation coeficient between avg relative CN and expression
KCNMB4	1.177760854	0.321034662
CNOT2	1.177760854	0.286060345
KRT10	1.177829107	0.273178254
NSMAF	1.178014787	0.380000325
UBXN2B	1.178014787	0.380000325
CYP7A1	1.178014787	0.261419259
SDCBP	1.178014787	0.224580007
RRS1	1.179196626	0.379899533
ARMC1	1.179196626	0.345966433
MTFR1	1.179196626	0.311176606
PDE7A	1.179196626	0.222564179
EIF2AK1	1.179441268	0.415949252
WIPI2	1.179441268	0.378018094
RBAK	1.179441268	0.305784267
PMS2	1.179441268	0.255690948
RNF216	1.179441268	0.235751052
SEC23B	1.179811154	0.38587256
MAFK	1.181132577	0.436745875
NUDT1	1.181132577	0.354046543
FTSJ2	1.181132577	0.348116649
ZFAND2A	1.181132577	0.347679887
C7orf50	1.181132577	0.305801066
SNX8	1.181132577	0.304625166
PSMG3	1.181132577	0.263653468
MAD1L1	1.181132577	0.244536702
TMEM184A	1.181132577	0.242907241
FGFR1OP2	1.181467817	0.380702661
MED21	1.181467817	0.326611739
CABLES1	1.182435311	0.361588393
ICA1	1.183455716	0.393607162
COPS5	1.183569932	0.443683682
ARFGEF1	1.183569932	0.427842636
VCPIP1	1.183569932	0.377698921
C8orf44	1.183569932	0.357339061
SGK3	1.183569932	0.336256863
CSPP1	1.183569932	0.317022507
SNRPB2	1.185446157	0.390595541
RAB2A	1.185814913	0.40808409
HNRNPA2B1	1.188236219	0.459591923
CBX3	1.188236219	0.34307708
NFE2L3	1.188236219	0.244452709
AZGP1	1.188497826	0.312013909
COPS6	1.188497826	0.268338009
TAF6	1.188497826	0.242266857
ZKSCAN1	1.188497826	0.217690764
EIF3B	1.188724439	0.430664795
CHST12	1.188724439	0.315477039
DBNL	1.189799235	0.504553188
PURB	1.189799235	0.41283457
NUDCD3	1.189799235	0.371410007
ZMIZ2	1.189799235	0.363313236
UBE2D4	1.189799235	0.302133894
CCM2	1.189799235	0.272568918
MYO1G	1.189799235	0.221334159
PPIA	1.189799235	0.21448046
TASP1	1.190079101	0.360022225

Table S2. One ordered list of genes used as input to GSEA.

Gene	Average relative CN	Correlation coeficient between avg relative CN and expression
ESF1	1.190079101	0.338861255
STARD3NL	1.191774599	0.307948691
C20orf196	1.191991516	0.438548081
TRMT6	1.191991516	0.436732843
CRLS1	1.191991516	0.365753671
CDS2	1.191991516	0.329028715
MCM8	1.191991516	0.322961114
PCNA	1.191991516	0.293110531
FERMT1	1.191991516	0.265176034
ZNF133	1.193257436	0.344491854
POLR3F	1.193257436	0.298892401
RBBP9	1.193257436	0.242519173
ANKMY2	1.193657139	0.353072226
TSPAN13	1.193657139	0.316955313
BZW2	1.193657139	0.246619724
MKKS	1.19530586	0.328978292
PSMF1	1.195984672	0.449355471
STK35	1.195984672	0.367417639
NSFL1C	1.195984672	0.338121713
FKBP1A	1.195984672	0.220753123
ITPA	1.196420446	0.508367517
CENPB	1.196420446	0.429219774
CDC25B	1.196420446	0.339768874
DDRGK1	1.196420446	0.295379579
ATRN	1.196420446	0.276285963
TMEM106B	1.197260373	0.23355044
FAM110A	1.197781373	0.234533511
TRAM1	1.198543354	0.401653674
LACTB2	1.198543354	0.357691831
MACC1	1.199093633	0.271296815
KBTBD2	1.1991189	0.430745163
AVL9	1.1991189	0.408772826
HERPUD2	1.1991189	0.388312346
AOAH	1.1991189	0.301414104
VPS41	1.1991189	0.281474376
RP9	1.1991189	0.237764879
CRNKL1	1.200033389	0.385401943
SMOX	1.200216376	0.247628732
PTPRA	1.200793751	0.506216124
IDH3B	1.200793751	0.441775171
VPS16	1.200793751	0.347920638
SNRPB	1.200793751	0.33304577
ZNF343	1.200793751	0.32388554
NOP56	1.200793751	0.30415323
TRIM4	1.201127278	0.431299511
SRXN1	1.202202338	0.245441656
OSBPL3	1.203948559	0.244637493
IRF2BP2	1.204791197	0.322356034
NOD1	1.205345711	0.352498109
CHN2	1.205345711	0.334355812
GGCT	1.205345711	0.297785645
PLEKHA8	1.205345711	0.277997102
CPVL	1.205345711	0.234959543
PRR15	1.205345711	0.220529697
SCRN1	1.205345711	0.214599854
NANP	1.20625891	0.371417886

Table S2. One ordered list of genes used as input to GSEA.

Gene	Average relative CN	Correlation coeficient between avg relative CN and expression
ABHD12	1.20625891	0.367199139
GINS1	1.20625891	0.320019755
ENTPD6	1.20625891	0.253713141
RBM34	1.206602502	0.317162436
TOMM20	1.206602502	0.314288309
RBCK1	1.206642693	0.534179737
CSNK2A1	1.206642693	0.388457683
TBC1D20	1.206642693	0.360926803
TRIB3	1.206642693	0.277628245
SOX12	1.206642693	0.263022425
ZCCHC3	1.206642693	0.233205709
C20orf96	1.206642693	0.220902532
XRN2	1.206876174	0.326994976
CCDC126	1.207343914	0.311646966
TRA2A	1.207343914	0.286230738
SP4	1.207343914	0.266374835
FAM126A	1.207343914	0.265282929
STK31	1.207343914	0.215743962
UBOX5	1.209540362	0.397049721
FASTKD5	1.209540362	0.317464976
MRPS26	1.209540362	0.282588873
SNX5	1.211974214	0.355500937
NXT1	1.21213435	0.437892578
GZF1	1.21213435	0.357988486
CST3	1.21213435	0.339651219
NAPB	1.21213435	0.281311484
CPSF6	1.215944	0.302186832
YEATS4	1.215944	0.240150255
PLCB1	1.222541441	0.239661854
PLCB4	1.222541441	0.218198344
RBBP8	1.223635047	0.325888409
GSDMB	1.227919341	0.353391399
CASC3	1.227919341	0.335114561
ORMDL3	1.227919341	0.271397606
WIPF2	1.227919341	0.259033863
MED24	1.227919341	0.257941956
PSMD3	1.227919341	0.254179077
TNS4	1.227919341	0.228208497
OSGIN2	1.229059003	0.405735725
NBN	1.229059003	0.310067901
DECR1	1.229059003	0.296998618
STAM	1.23122084	0.240149279
TNS3	1.232827524	0.343393363
TERF1	1.233891992	0.338393488
KIAA1429	1.23520151	0.403535113
RAD54B	1.23520151	0.394228709
TMEM67	1.23520151	0.291035128
TSNARE1	1.236115275	0.336503178
GLI4	1.236115275	0.309155576
ZFP41	1.236115275	0.284764471
PTDSS1	1.23899744	0.562785504
RPL30	1.23899744	0.527042577
MTERFD1	1.23899744	0.518991646
LAPTM4B	1.23899744	0.462699657
MTDH	1.23899744	0.378152483
HRSP12	1.23899744	0.325337796

Table S2. One ordered list of genes used as input to GSEA.

Gene	Average relative CN	Correlation coeficient between avg relative CN and expression
POP1	1.23899744	0.300559914
C8orf37	1.23899744	0.288397753
UQCRB	1.23899744	0.247526846
HOXA1	1.240643197	0.251186819
PSKH2	1.241111081	0.336794417
CPNE3	1.241111081	0.297418582
RIPK2	1.241688455	0.305330706
UQCRFS1	1.242144031	0.460217161
INTS8	1.242793371	0.49075326
DPY19L4	1.242793371	0.428581773
PLEKHF2	1.242793371	0.36683025
CCNE2	1.242793371	0.297166604
ZNF16	1.244647632	0.592475394
C8orf33	1.244647632	0.426340389
BET1	1.245460943	0.217306229
VPS13B	1.245463236	0.383780002
TOP1MT	1.245802381	0.444852095
SCRIB	1.245802381	0.440618928
ZNF696	1.245802381	0.35770261
ZNF623	1.245802381	0.357097872
ZC3H3	1.245802381	0.335696861
EEF1D	1.245802381	0.283689381
TSTA3	1.245802381	0.283554994
GSDMD	1.245802381	0.246229213
FAM83H	1.245802381	0.245994037
PYCRL	1.245802381	0.219387398
TAX1BP1	1.246870623	0.34566115
SMARCE1	1.251964739	0.351358772
VPS28	1.253394243	0.59506233
RPL8	1.253394243	0.563179191
ZNF7	1.253394243	0.46054169
COMMD5	1.253394243	0.457249227
CPSF1	1.253394243	0.410650793
SLC39A4	1.253394243	0.398119275
PPP1R16A	1.253394243	0.376382299
MAF1	1.253394243	0.370637287
CYHR1	1.253394243	0.360323142
EXOSC4	1.253394243	0.350445752
GPAA1	1.253394243	0.337275899
BOP1	1.253394243	0.335058526
C8orf82	1.253394243	0.307677327
FBXL6	1.253394243	0.306266271
CYC1	1.253394243	0.298558363
SCRT1	1.253394243	0.298236693
GRINA	1.253394243	0.27293848
LRRC14	1.253394243	0.269108472
SHARPIN	1.253394243	0.253822036
MFSD3	1.253394243	0.247169916
PARP10	1.253394243	0.236889368
KIFC2	1.253394243	0.232689798
DGAT1	1.253394243	0.227549523
ADCK5	1.253394243	0.221300563
TMEM70	1.254344877	0.451444619
TCEB1	1.254344877	0.420602455
RPL7	1.254344877	0.415361303
STAU2	1.254344877	0.399621048

Table S2. One ordered list of genes used as input to GSEA.

Gene	Average relative CN	Correlation coeficient between avg relative CN and expression
RDH10	1.254344877	0.23859001
HNF4G	1.254344877	0.221455474
AZIN1	1.259057298	0.472266891
UBR5	1.259057298	0.458458703
SLC25A32	1.259057298	0.449354034
ATP6V1C1	1.259057298	0.346951709
GRHL2	1.259057298	0.314363043
ODF1	1.259057298	0.267025485
FZD6	1.259057298	0.241693677
RRM2B	1.259057298	0.235343926
COX6C	1.260426325	0.446959402
POLR2K	1.260426325	0.331670855
DENND3	1.260756319	0.298169499
SLC45A4	1.260756319	0.245758861
TRAPPC9	1.260933959	0.29054308
FAM135B	1.260933959	0.271762601
ZFAT	1.260933959	0.256375375
PHF20L1	1.262396205	0.420595376
NDRG1	1.262396205	0.403225953
TG	1.262396205	0.266319957
LRRC6	1.262396205	0.249118517
ZNF706	1.263900852	0.36747921
IMPA1	1.264455534	0.399469861
MRPS28	1.264455534	0.393842342
CHMP4C	1.264455534	0.364680034
ZFAND1	1.264455534	0.338591863
SNX16	1.264455534	0.264073432
TPD52	1.264455534	0.261016093
MIB1	1.265075886	0.504068977
ESCO1	1.265075886	0.402750944
SNRPD1	1.265075886	0.334040242
ABHD3	1.265075886	0.240134494
SMURF1	1.266963515	0.347307396
TAF2	1.269657125	0.550681269
MTBP	1.269657125	0.373963346
DSCC1	1.269657125	0.342382577
MRPL13	1.269657125	0.296019319
DERL1	1.269657125	0.291819749
SNTB1	1.269657125	0.263665829
ATAD2	1.269657125	0.256240988
WDR67	1.269657125	0.235881471
C8orf76	1.269657125	0.215790726
PTK2	1.270345634	0.396372254
TES	1.273700871	0.339623326
PABPC1	1.275537193	0.488729207
ANKRD46	1.275537193	0.269108472
SNX31	1.275537193	0.240920956
ZNF789	1.277162393	0.478738257
ZNF394	1.277162393	0.457740229
ARPC1B	1.277162393	0.444116708
ARPC1A	1.277162393	0.359990205
PTCD1	1.277162393	0.307243157
BUD31	1.277162393	0.298440783
CYP3A5	1.277162393	0.255638401
ATP5J2	1.277162393	0.252144329
ZKSCAN5	1.277162393	0.22753464

Table S2. One ordered list of genes used as input to GSEA.

Gene	Average relative CN	Correlation coeficient between avg relative CN and expression
RAB3IP	1.277369062	0.283859752
YWHAZ	1.280380746	0.315034974
UTP23	1.281942138	0.301461963
EIF3H	1.281942138	0.292390891
MED30	1.281942138	0.290946238
RAD21	1.281942138	0.256140199
CHRAC1	1.282800486	0.316042871
FAM91A1	1.284110081	0.385251792
TATDN1	1.284110081	0.371846763
RNF139	1.284110081	0.342180997
NDUFB9	1.284110081	0.310667421
TRMT12	1.284110081	0.267697417
TMEM65	1.284110081	0.255266688
CRY2	1.291343543	0.432598134
GSDMC	1.294872811	0.228994176
SLC27A3	1.307577428	0.42864831
RAB13	1.307577428	0.363484624
CREB3L4	1.307577428	0.287328662
SNAPIN	1.307577428	0.218534499
EIF3E	1.309336694	0.317890682
EBAG9	1.312826937	0.438132782
NUDCD1	1.312826937	0.293465981
BEND3	1.314655194	0.347962534
C6orf203	1.314655194	0.304152247
PLCG1	1.317784289	0.343161151
CHD6	1.317784289	0.265190607
EMILIN3	1.317784289	0.25317316
TOP1	1.317784289	0.247777915
NSMCE2	1.318213212	0.271829794
KIAA0196	1.318213212	0.247942637
QRSL1	1.321411951	0.447657887
RTN4IP1	1.321411951	0.40703353
ANP32E	1.322348355	0.280252595
ASXL1	1.325375068	0.520906106
TM9SF4	1.325375068	0.473659492
COMMD7	1.325375068	0.466650656
KIF3B	1.325375068	0.457994659
PLAGL2	1.325375068	0.451406017
PDRG1	1.325375068	0.449742049
TPX2	1.325375068	0.43765727
HM13	1.325375068	0.429186158
POFUT1	1.325375068	0.424950603
MAPRE1	1.325375068	0.357467445
BCL2L1	1.325375068	0.331213724
DNMT3B	1.325375068	0.326810091
C20orf112	1.325375068	0.214147674
ATG5	1.326008384	0.413302825
YTHDF1	1.327346849	0.598892338
NKAIN4	1.327346849	0.217302572
CSTF1	1.329213769	0.594848957
AURKA	1.329213769	0.517089057
DNAJC5	1.332190403	0.558038917
PRPF6	1.332190403	0.549438197
UCKL1	1.332190403	0.508181617
TPD52L2	1.332190403	0.473106805
ARFRP1	1.332190403	0.403897884

Table S2. One ordered list of genes used as input to GSEA.

Gene	Average relative CN	Correlation coeficient between avg relative CN and expression
ARFGAP1	1.332190403	0.341173101
ZGPAT	1.332190403	0.33589844
GMEB2	1.332190403	0.317017171
SLC2A4RG	1.332190403	0.309189172
TFAP2C	1.334057323	0.391504047
RBL1	1.334284058	0.38057491
RPN2	1.334284058	0.330874781
CTNNBL1	1.336402138	0.449872721
RPRD1B	1.336402138	0.412089195
TGM2	1.336402138	0.248786372
ADRM1	1.336992786	0.60346147
TAF4	1.336992786	0.586192837
OSBPL2	1.336992786	0.563683139
PSMA7	1.336992786	0.527969993
GTPBP5	1.336992786	0.514632157
RPS21	1.336992786	0.481539542
TCFL5	1.336992786	0.439342259
DIDO1	1.336992786	0.435579443
SS18L1	1.336992786	0.422947136
LAMA5	1.336992786	0.378364496
SLC17A9	1.336992786	0.33599923
CABLES2	1.336992786	0.321351128
SLCO4A1	1.336992786	0.289938341
COL9A3	1.336992786	0.282143939
NTSR1	1.336992786	0.250529573
OGFR	1.336992786	0.248614569
RALY	1.339164367	0.511489452
DYNLRB1	1.339164367	0.493169147
CHMP4B	1.339164367	0.478059098
CDK5RAP1	1.339164367	0.472058778
PIGU	1.339164367	0.414761606
EIF2S2	1.339164367	0.397348913
AHCY	1.339164367	0.344051954
CBFA2T2	1.339164367	0.317159764
ITCH	1.339164367	0.304251513
PXMP4	1.339164367	0.293007216
ZNF341	1.339164367	0.262988809
NECAB3	1.339164367	0.24187844
ACTR5	1.340198069	0.473437003
MANBAL	1.340198069	0.407315831
DHX35	1.340198069	0.353228914
BLCAP	1.340198069	0.307680267
SRC	1.340198069	0.236533618
RAE1	1.340848033	0.590531763
PCMTD2	1.341051723	0.369595793
RAB22A	1.347704491	0.558576462
SLMO2	1.347704491	0.400235859
GNAS	1.347704491	0.396237868
PPP1R3D	1.347704491	0.387166796
STX16	1.347704491	0.374366505
PMEPA1	1.347704491	0.290946238
ATP5E	1.347704491	0.227213558
VAPB	1.347704491	0.214446864
IFT52	1.349440994	0.478294405
MYBL2	1.349440994	0.417702939
PEX16	1.352042539	0.257310002

Table S2. One ordered list of genes used as input to GSEA.

Gene	Average relative CN	Correlation coeficient between avg relative CN and expression
MAPK8IP1	1.352042539	0.243090637
MOCS3	1.352115282	0.546313716
DPM1	1.352115282	0.482312263
SLC9A8	1.352115282	0.410717987
ADNP	1.352115282	0.409810879
PARD6B	1.352115282	0.361028669
NCOA6	1.354096426	0.40429046
UBE2V1	1.355858637	0.603024715
TMEM189	1.355858637	0.592139429
TMEM189-UB	1.355858637	0.592139429
RNF114	1.355858637	0.562876822
SPATA2	1.355858637	0.538317734
CEBPB	1.355858637	0.455804575
PTPN1	1.355858637	0.351621632
SNAI1	1.355858637	0.325718681
NCOA3	1.355977598	0.374739221
SULF2	1.355977598	0.268589986
TGIF2	1.357266224	0.507321162
DSN1	1.357266224	0.456007502
C20orf24	1.357266224	0.414576722
NDRG3	1.357266224	0.40400473
CPNE1	1.357329324	0.549373824
EIF6	1.357329324	0.52081768
ROMO1	1.357329324	0.465688691
TRPC4AP	1.357329324	0.464512157
PHF20	1.357329324	0.437166162
ERGIC3	1.357329324	0.434392905
SCAND1	1.357329324	0.423215838
NFS1	1.357329324	0.397853142
EDEM2	1.357329324	0.360288114
UQCC	1.357329324	0.355581981
PROCR	1.357329324	0.301209334
RBM12	1.357329324	0.295528359
RBM39	1.357329324	0.269560588
CEP250	1.357329324	0.216011515
SALL4	1.358342707	0.372989046
ZFP64	1.358342707	0.370435707
CD44	1.359594112	0.214298943
YWHAB	1.359726083	0.560222693
PABPC1L	1.359726083	0.262624335
DDX27	1.361802388	0.611860611
STAU1	1.361802388	0.557299792
CSE1L	1.361802388	0.508618372
ARFGEF2	1.361802388	0.490912983
ZNFX1	1.361802388	0.394994795
B4GALT5	1.361802388	0.382799242
ZNF217	1.362138638	0.406619206
PFDN4	1.362138638	0.343457667
DNTTIP1	1.367317944	0.533513426
UBE2C	1.367317944	0.50492275
ACOT8	1.367317944	0.481405156
TOMM34	1.367317944	0.452646498
ZSWIM3	1.367317944	0.441559632
PCIF1	1.367317944	0.42251038
SYS1	1.367317944	0.391903911
STK4	1.367317944	0.342281787

Table S2. One ordered list of genes used as input to GSEA.

Gene	Average relative CN	Correlation coeficient between avg relative CN and expression
ZSWIM1	1.367317944	0.307912503
DBNDD2	1.367317944	0.307374958
PI3	1.367317944	0.297598358
PLTP	1.367317944	0.295952126
PIGT	1.367317944	0.269209262
SLPI	1.367317944	0.251470276
CTSA	1.367317944	0.243339908
NEURL2	1.367317944	0.226071275
SEMG1	1.367317944	0.219116786
C20orf111	1.371171173	0.587872665
HNF4A	1.371171173	0.245456492
TP53RK	1.372161498	0.531195263
SLC35C2	1.372161498	0.484966392
NCOA5	1.372161498	0.392038297
ELMO2	1.372161498	0.350714524
CD40	1.372161498	0.230539617
TTPAL	1.374699505	0.524543143
SERINC3	1.374699505	0.450227545
AKAP9	1.392487581	0.281772414
MTERF	1.399144484	0.241346696
FAM84B	1.432641636	0.347933004
STARD3	1.486653175	0.404828603
ERBB2	1.486653175	0.295402754
GRB7	1.486653175	0.281375953
KRIT1	1.503974067	0.318186963
CYP51A1	1.503974067	0.283346154
MYC	1.530464431	0.334634918
FGFR2	1.545160574	0.262505082
SAMD9L	1.571674865	0.306271426
POP4	1.583277699	0.572140979
C19orf12	1.601000339	0.474823228
PLEKHF1	1.601000339	0.255412877
CCNE1	1.668810084	0.494101891
KRAS	1.722195049	0.484651302
CASC1	1.722195049	0.339731307
LRMP	1.722195049	0.22444373
SAMD9	1.790950745	0.384871908
CDK6	1.798437456	0.392165785
GATAD1	1.798437456	0.392014597
PEX1	1.798437456	0.285813179
